# Assessing ocular activity during performance of motor skills using electrooculography

**DOI:** 10.1111/psyp.13070

**Published:** 2018-02-09

**Authors:** Germano Gallicchio, Andrew Cooke, Christopher Ring

**Affiliations:** ^1^ School of Sport, Exercise & Rehabilitation Sciences University of Birmingham Birmingham United Kingdom; ^2^ School of Sport, Health & Exercise Sciences Bangor University Bangor United Kingdom

**Keywords:** EOG, eye quietness, golf putting, ocular behavior, quiet eye

## Abstract

Eye‐tracking research has revealed that, compared to novices, experts make longer ocular fixations on the target of an action when performing motor skills; that is, they have a longer *quiet eye*. Remarkably, the reason why a longer quiet eye aids movement has yet to be established. There is a need for interdisciplinary research and new measures to accelerate progress on the mechanistic understanding of the phenomenon. With the aim to provide researchers with new tools, we assessed the utility of electrooculography (EOG) to examine ocular activity while 10 experts and 10 novices putted golf balls. We measured quiet eye durations, distinguishing its pre‐ and postmovement initiation components, and developed a novel time‐varying index of ocular activity, eye quietness, computed as the variability of the EOG in short time intervals: lower values correspond with greater quietness. Finally, we measured movement durations using a combination of infrared and sound sensors. Experts had longer postmovement initiation quiet eye compared to novices; however, total and premovement quiet eye durations did not differ between groups. Eye quietness was inversely correlated with quiet eye duration, and was greatest immediately after movement initiation. Importantly, movement duration correlated positively with postmovement initiation quiet eye and negatively with eye quietness shortly after movement initiation. This study demonstrates the utility of assessing ocular activity during performance of motor skills using EOG. Additionally, these findings provide evidence that expert–novice differences in ocular activity may reflect differences in the kinematics (e.g., movement duration) of how experts and novices execute motor skills.

## 1 INTRODUCTION

The study of eye movements during performance of motor skills can yield important information to understand how individuals control their actions. In a seminal study, Vickers (1992) used camera‐based eye tracking to examine the gaze of 12 experienced golfers—comprising five skilled golfers (mean handicap 6.2) and seven less skilled golfers (mean handicap 14.1)^1^—as they putted balls to a 3‐m distant hole. Vickers found that, compared to the less skilled golfers, skilled golfers made fewer and longer fixations on the ball prior to movement initiation, during movement execution, and even after movement completion. In the intervening 25 years since this influential initial report of visual gaze control in putting, researchers have used camera‐based eye tracking to examine individuals' ocular activity, and especially their *quiet eye*, during performance of motor skills.

The quiet eye is defined as the final ocular fixation on the target location (e.g., the ball in golf putting), with onset occurring prior to initiation of a critical phase of the movement and offset occurring when the gaze deviates from the target location (Vickers, 1996, 2007). A compelling body of literature has reported that experts show longer quiet eye durations than novices for a variety of motor skills, ranging from precision sports to surgery (for reviews, see Gonzalez et al., 2017; Rienhoff, Tirp, Strauß, Baker, & Schorer, 2016; Vickers, 2007; Wilson, Causer, & Vickers, 2015; for meta‐analyses, see Lebeau et al., 2016; Mann, Williams, Ward, & Janelle, 2007). For instance, Walters‐Symons, Wilson, and Vine (2017) tested 18 experienced golfers (mean handicap 5.7) and 21 novices (no formal handicap), as they putted balls to a 10‐ft (i.e., 3‐m) distant hole. They found that the experienced golfers had longer quiet eye durations (*M* = 1.9 s) than the novices (*M* = 1.2 s). As a result of extensive research, long quiet eye is currently considered a feature of expertise and is often cited along classic models of skill acquisition (e.g., Fitts & Posner, 1967), whereby experts have greater movement accuracy, consistency, automaticity, and efficiency than novices.

Despite the robustness of the quiet eye phenomenon in revealing differences between expertise levels, there is no consensus on whether and how the quiet eye influences motor performance (e.g., Causer, 2016; Williams, 2016; Wilson, Wood, & Vine, 2016). A number of possible cognitive mechanisms have been proposed. The dominant hypothesis (Vickers, 1996) contends that movement‐related visual processing is enhanced, and movement parameters, such as force and direction, are programmed during the quiet eye period. Therefore, an extended quiet eye period could lead to improved motor programming and, consequently, to enhanced motor performance. Other hypotheses argue that a longer quiet eye duration allows inhibition of task‐irrelevant processing (Klostermann, Kredel, & Hossner, 2014) or promotes an external focus of attention (Vine, Moore, & Wilson, 2014), which has been associated with improved motor performance (Wulf, 2013). An alternative hypothesis is that the quiet eye reflects psychomotor quiescence. Accordingly, rather than eliciting cognitive benefits, the longer quiet eye of experts could be associated with (or be a consequence of) the cleaner and more consistent movement kinematics of expert compared to novice performers, such as a slower and more stable swing in golf putting (Cooke et al., 2014; Delay, Nougier, Orliaguet, & Coello, 1997; Sim & Kim, 2010).

None of the studies to date have provided unequivocal empirical evidence that a longer quiet eye is directly associated with enhanced visual perception or cognitive processing, while our newly suggested kinematic hypothesis has yet to be tested. To shed light on these fundamental questions of why experts have a longer quiet eye than novices and why a longer quiet eye aids performance, researchers have been encouraged to employ objective psychophysiological measures to simultaneously assess cognitive, physiological, and kinematic variables (for review of research in sport psychophysiology, see Cooke, 2013; Hatfield, Haufler, Hung, & Spalding, 2004). Unfortunately, the simultaneous assessment of eye movements with such psychophysiological and kinematic variables is a challenge for camera‐based eye trackers—the primary and often only technique used to assess ocular activity by previous quiet eye and human performance research. Fortunately, an alternate psychophysiological tool used to record eye movements exists. Electrooculography (EOG) measures time‐varying changes in the electric dipoles of the eyes, by recording voltage differences from electrodes placed close to the eyes (Shackel, 1967; Young & Sheena, 1975). A goal of this study is to apply novel EOG methods to quiet eye research and shed new light on the relationship between ocular activity and performance. Some advantages of EOG for quiet eye researchers are as follows.

First, the eyes move at speeds up to 100 Hz (Krauzlis, Goffart, & Hafed, 2017). Therefore, based on the Nyquist‐Shannon sampling theorem (Shannon, 1948), ocular activity should be sampled at least at 200 Hz (corresponding to one data point every 5 ms) to prevent aliasing and avoid temporal distortions (i.e., key features of the signal are missed or altered). Because typical camera‐based mobile eye‐tracking systems sample data at 30 Hz (i.e., one frame every 33 ms), researchers have expressed the need for tools with greater temporal sensitivity than the ones used to date (e.g., Causer, 2016; Gonzalez et al., 2017; Williams, 2016). Typical systems for psychophysiological recording have a sampling frequency of 512 Hz (i.e., one voltage value every 2 ms) or higher. Accordingly, the EOG offers sufficient temporal precision to fully capture time‐varying ocular activity.^2^

Second, by definition, the quiet eye period can extend beyond movement initiation and even beyond movement completion as long as the eyes are on the target (Vickers, 1996, 2007). Because the preprogramming of movement parameters such as direction and force ends with movement initiation, mechanistic studies of the quiet eye should benefit from distinguishing the pre‐ and postmovement initiation components of the quiet eye period. Surprisingly, only a few recent camera‐based studies have reported these components in a golf putting task (Causer, Hayes, Hooper, & Bennett, 2017; Vine, Lee, Moore, & Wilson, 2013; Walters‐Symons, Wilson, Klostermann, & Vine, 2017). Causer et al. (2017) found that, for novice golfers (no formal handicap), longer quiet eye durations were associated with better performance (lower radial error) in both the pre‐ and postmovement initiation phases of the putt. Vine et al. (2013) found that for experts (mean handicap 3.6) only the postmovement initiation component of the quiet eye distinguished holed from missed putts (longer duration for holed putts). Finally, Walters‐Symons, Wilson, Klostermann, & Vine (2017) tested experienced golfers (mean handicap 6.4) and found that, compared to shorter putts (4 ft, 1.2 m), longer putts (8 ft, 2.4 m) were associated with less accuracy and longer postmovement initiation quiet eye durations. They also found no differences in premovement initiation quiet eye durations between long and short putts. These findings cast doubt on any quiet eye mechanism that concerns what happens before movement initiation, such as improved preprogramming of movement parameters. By exploiting the multimeasure approach favored in psychophysiology, EOG recordings can be supplemented with external transducers (e.g., an infrared sensor) to detect movement initiation, such as the beginning of the backswing in golf putting (e.g., Cooke, Kavussanu, McIntyre, & Ring, 2010), thereby ensuring that both pre‐ and postmovement initiation components of the quiet eye can be easily explored.

Third, the eyes are not completely still during a fixation (e.g., Krauzlis et al., 2017). Therefore, identifying a quiet eye period requires a threshold criterion to be applied. Because the fovea corresponds to less than 2° of the visual field (Guyton & Hall, 2006), most quiet eye studies have defined fixations in terms of when gaze remains within 3° or 1° of visual angle on the target location (Gonzalez et al., 2017; Lebeau et al., 2016; Vickers, 2007). Because the threshold influences the duration of the fixation, whereby stringent thresholds identify shorter fixations, the impact of threshold choice on quiet eye durations has been recommended as a research question to better understand the quiet eye phenomenon (Gonzalez et al., 2017). One of the strengths of data processing in psychophysiology is that the signal can be scored repeatedly and automatically using different settings, such as voltage thresholds in the EOG.

Fourth, the EOG allows researchers to examine the quiet eye phenomenon from a novel perspective that is commonplace in psychophysiology, where signals are measured as a function of time relative to a critical event. Accordingly, instead of defining quietness using a threshold and measuring quiet eye duration (see previous point), researchers could quantify the amount of eye quietness as a function of time relative to movement initiation (e.g., Webb & Obrist, 1970).

To date, only one study has used the EOG to examine the quiet eye in a golf putting task. Mann, Coombes, Mousseau, and Janelle (2011) tested 10 skilled (mean handicap 1.2) and 10 less skilled golfers (mean handicap 11.3) as they putted balls to a 12‐ft (i.e., 3.7‐m) distant hole. They computed the quiet eye by applying a voltage threshold to the EOG signal and found that the more skilled golfers had longer quiet eye durations (around 2.3 s) compared to the less skilled golfers (around 2.1 s). However, they only scored the premovement initiation component of the quiet eye, and not the potentially more important postmovement initiation component (Vine et al., 2013; Walters‐Symons, Wilson, Klostermann, & Vine, 2017). Furthermore, they applied an atypical threshold criterion of 100 µV (corresponding to 5° of visual angle) to the EOG signal; all other golf putting studies have employed a threshold of either 1° or 3° of visual angle (for reviews, see Gonzalez et al., 2017; Lebeau et al., 2016).

With the overarching goal of introducing psychophysiological methods to quiet eye research, this study evaluated the utility of EOG in assessing ocular activity during performance of motor skills. We conducted new analyses on a golf putting data set with known expert–novice and holed–missed differences for several psychophysiological indices (Cooke et al., 2014). Our primary aims were threefold: first, to quantify both pre‐ and postmovement initiation components of the quiet eye using EOG; second, to develop a novel, time‐varying measure of ocular activity in the form of eye quietness. In line with the existing literature, we expected that quiet eye durations would be longer and eye quietness greater in experts compared to novices and on holed putts compared to missed putts. Third, we aimed to evaluate the validity of the eye quietness index by assessing its correlation with quiet eye durations. We expected that the two measures would be highly negatively correlated.

Our secondary aims were threefold: first, to examine the impact of threshold level (e.g., 1° or 3° of visual angle) on quiet eye duration (we expected that more stringent thresholds would generate shorter quiet eye periods); second, to determine the influence of expertise on the consistency of indices of ocular activity and kinematics across putts (we expected greater consistency in experts based on theoretical models arguing for decreased performance variability as a function increased expertise and learning, e.g., Fitts & Posner, 1967). Finally, we examined the relation between ocular activity (i.e., quiet eye durations, eye quietness) and swing duration. This analysis provided the first test of our kinematic hypothesis of the relationship between quiet eye and performance; namely, a longer quiet eye is associated with a cleaner and more consistent technique. We expected that longer swing durations would be associated with longer postmovement initiation quiet eye durations and greater eye quietness during swing execution.

## 2 METHOD

### 2.1 Participants

Twenty right‐handed male golfers took part in this study. Ten were experts (age: *M* = 20.90, *SD* = 0.74 years; experience: *M* = 11.25, *SD* = 3.78 years; handicap: *M* = 1.50, *SD* = 2.32) and 10 were novices (age: *M* = 19.00, *SD* = 0.66 years; experience: *M* = 1.85, *SD* = 2.49 years; no formal handicap). All provided informed consent.

### 2.2 Putting task

Participants putted golf balls (diameter 4.7 cm) on an artificial flat putting surface (Turftiles) to a 2.4‐m distant hole, using a blade‐style putter (length 90 cm). The hole was of regular size for novices (diameter: 10.8 cm) and half‐size for experts (diameter: 5.4 cm). This difference in hole size was chosen so that the two groups holed a similar number of putts, and thereby putting outcome (holed, missed) could be used as a factor in our analyses (cf. Babiloni et al., 2008). Indeed, the performance of the two groups did not differ, *t*(18) = 1.18, *p* = .25, *r* ^2^ = .072, with experts holing 41% (*SD* = 17%) and novices holing 31% (*SD* = 19%) of putts. Participants were instructed to get each ball “ideally in the hole, but if unsuccessful, to make them finish as close to the hole as possible.” Addressing of the ball, movement initiation (i.e., beginning of backswing), and putter–ball impact were detected through the combination of infrared (S51‐PA 2‐C10PK, Datasensor, Monte San Pietro, Italy) and sound (NT1, Rode, Silverwater, Australia) sensors.

### 2.3 EOG signal

**Figure 1:**
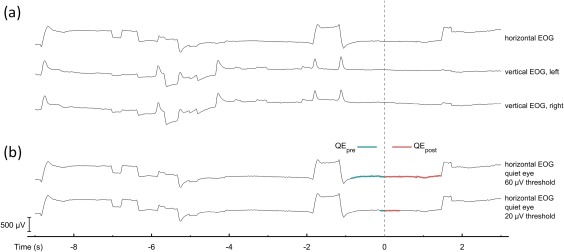
(a) Horizontal and vertical (left and right eye) EOG signals. Voltages (µV) are represented as function of time (s). Voltage increases indicate eye movements to the left or upward; voltage decreases indicate movements to the right or downward. Eyeblinks are evident in the vertical EOG signals. (b) Output of the QE algorithm with 60 and 20 µV thresholds. Thick colored lines indicate the quiet eye period in its premovement initiation (QE_pre_) and postmovement initiation (QE_post_) components

Three pairs of Ag‐AgCl electrodes, each with an integrated preamplifier, were applied to the participant's skin. These were placed below and at the outer canthi of both eyes as well as on the forehead (Fp1 and Fp2 location in the 10–20 system, Jasper, 1958). Common mode sense and driven right leg electrodes were used instead of ground and reference electrodes to enhance the common mode rejection ratio of the signal. Voltages were recorded and digitized at 512 Hz (24‐bit resolution) using the ActiveTwo system (BioSemi, Netherlands). Offline, the electrodes were bipolar‐referenced to obtain one horizontal EOG and two vertical EOG channels: for the horizontal channel, positive and negative voltages indicated eye movements, respectively, to the left and to the right; for the vertical channels, positive and negative voltages indicated, respectively, upward and downward eye movements. The signals were band‐pass filtered 0.1 to 30 Hz (FIR, Order 512) according to guidelines (Marmor et al., 2011). Epochs were extracted from −9 to +3 s relative to movement initiation (i.e., beginning of backswing), unless two contiguous trial epochs overlapped (in this case, the prebackswing portion was cut shorter). All participants' vertical and horizontal EOG signals are presented in the online supporting information (Appendix S1), and examples are shown in Figure 1a. As golf putting is performed in the frontal plane, we focused our analyses on the horizontal signals. Signal processing was performed using MATLAB (MathWorks, Natick, MA).

### 2.4 Procedure

Following instrumentation and task familiarization (20 putts), participants putted 60 balls in each of two counterbalanced pressure conditions. Due to the methodological nature of this study, only the no‐pressure condition was analyzed. It is worth noting that the null effects of pressure on performance and other psychophysiological signals have been reported by Cooke et al. (2014). The mean interputt interval for the no‐pressure condition was 15.44 s (*SD* = 1.90). Light conditions were kept constant throughout testing.

### 2.5 Measures

#### 2.5.1 Quiet eye

The duration of the total quiet eye (QE_total_) was measured as the time (in seconds) between quiet eye onset and quiet eye offset. QE_total_ comprised the sum of the premovement initiation (QE_pre_) and postmovement initiation (QE_post_) components. The onset and offset of the quiet eye were detected using a voltage‐threshold algorithm, which is described in detail in supporting information (Appendix S2). This algorithm was employed twice: once using a 60 µV threshold and once using a 20 µV threshold, corresponding with eye movements of 3° and 1° of visual angle, respectively (Shackel, 1967; cf. Mann et al., 2011). The outputs of both algorithms for all participants are presented in the supporting information (Appendix S3); examples are shown in Figure 1b.

#### 2.5.2 Eye quietness

Eye quietness was operationalized as the standard deviation of the horizontal EOG signal (HEOG‐SD), measured in µV, within each of 12 0.5‐s bins, ranging from −4 to +2 s relative to movement initiation. Lower HEOG‐SD values correspond with less movement of the eyes (i.e., greater quietness). The bin width was chosen following exploratory analyses using a range of widths (0.125, 0.25, 0.5, 1 s): 0.5 s was sufficiently brief to capture variation in eye quietness in the context of golf putting, whereas 1 s was too coarse.

#### 2.5.3 Putting times

Address time was measured as the time, in seconds, between the positioning of the putter head next to the ball and movement initiation (i.e., beginning of backswing). Swing time was measured as the time, in seconds, between movement initiation and putter–ball impact.

#### 2.5.4 Performance

Performance was measured as the percentage of holed putts.

### 2.6 Data reduction and statistical analyses

Putting times, quiet eye, and eye quietness measures were computed for each putt. These were used to compute each participant's (a) arithmetic mean, as an index of the average value, and (b) standard deviation, as an index of variability across putts. Analyses involving quiet eye durations were conducted twice, separately for the two thresholds (60 and 20 µV).

#### 2.6.1 Group, outcome, and time differences

Differences in quiet eye durations and putting times were examined using 2 Group (expert, novice) × 2 Outcome (holed, missed) analyses of variance (ANOVAs), with group as a between‐subjects factor and outcome as a within‐subject factor. Differences in eye quietness were examined using 2 Group (expert, novice) × 2 Outcome (holed, missed) × 12 Time (0.5‐s bins from −4 to +2 s) ANOVA, with group as a between‐subjects factor and outcome and time as within‐subject factors. The multivariate solution was adopted where appropriate (Vasey & Thayer, 1987) and Wilks's lambda (λ) reported. Univariate partial eta‐squared ( $\eta_{p}^{2}$) was reported as a measure of effect size, with values of .02, .13, and .26 reflecting small, medium, and large effects, respectively (Cohen, 1992). Significant interactions were interrogated using post hoc *t* tests (reported for *p* < .05).

#### 2.6.2 Relations between quiet eye and eye quietness

Pearson's correlations were conducted between quiet eye durations and eye quietness (HEOG‐SD) to examine the relationship between the two indices of ocular activity. Only relevant comparisons were considered: QE_pre_ with premovement initiation eye quietness and QE_post_ with postmovement initiation eye quietness.

#### 2.6.3 Impact of threshold on quiet eye durations

We employed 300 different thresholds, ranging from 2 to 600 µV (in 2 µV increments), corresponding to a range of 0.1° to 30° (in 0.1° increments) of visual angle. For each threshold, we evaluated group differences through independent samples *t* tests.

#### 2.6.4 Correlates of performance

Pearson's correlations were conducted between the percentage of holed putts and (a) quiet eye durations, (b) eye quietness, and (c) putting times. These correlations were performed separately for each group due to the different hole sizes (i.e., task difficulties) used for these two groups.

#### 2.6.5 Relations between putting times and ocular activity

Pearson's correlations were conducted to explore the relations between ocular activity (quiet eye and eye quietness) and putting times (address and swing times).

## 3 RESULTS

### 3.1 Group, outcome, and time differences

#### 3.1.1 Quiet eye

The mean (*SD*) quiet eye durations for each group's holed and missed putts are presented in Table 1. It is noteworthy that QE_total_ and QE_pre_ durations did not differ between experts and novices. However, experts had longer QE_post_ (for 60 µV and 20 µV thresholds) than novices. In terms of variability across putts, experts had less variable QE_total_ and QE_pre_ durations (for 60 µV threshold) but more variable QE_post_ duration (for 20 µV threshold) compared to novices.

**Table 1:** Mean (*SD*) of quiet eye durations with the results of the 2 Group (expert, novice) × 2 Outcome (holed, missed) mixed ANOVAs

	Experts (*n* = 10)		Novices (*n* = 10)		Group		Outcome		Group × Outcome	
Measures	Holed	Missed	Holed	Missed	*F*(1, 18)	$\eta_{p}^{2}$	*F*(1, 18)	$\eta_{p}^{2}$	*F*(1, 18)	$\eta_{p}^{2}$
Quiet eye durations (s), 60 µV										
QE_total_	1.983 (0.60)	2.002 (0.52)	2.400 (1.65)	2.557 (1.72)	0.78	.041	0.66	.035	0.40	.022
QE_pre_	1.032 (0.49)	1.061 (0.50)	1.848 (1.59)	2.014 (1.63)	2.88	.138	0.81	.043	0.40	.022
QE_post_	0.952 (0.21)	0.942 (0.23)	0.552 (0.25)	0.543 (0.21)	16.49^**^	.478	0.26	.014	0.00	0.00
*SD* QE_total_	0.57 (0.43)	0.55 (0.35)	1.15 (0.83)	1.19 (0.62)	6.61^*^	.268	0.01	.000	0.07	.003
*SD* QE_pre_	0.46 (0.49)	0.44 (0.38)	1.06 (0.86)	1.11 (0.68)	6.28^*^	.259	0.03	.002	0.07	.004
*SD* QE_post_	0.21 (0.12)	0.22 (0.08)	0.25 (0.06)	0.25 (0.08)	0.73	.039	0.05	.003	0.22	.012
Quiet eye durations (s), 20 µV										
QE_total_	0.705 (0.25)	0.655 (0.21)	0.664 (0.54)	0.627 (0.44)	0.04	.002	4.03	.183	0.08	.004
QE_pre_	0.417 (0.19)	0.381 (0.13)	0.497 (0.51)	0.454 (0.41)	0.25	.014	2.91	.139	0.02	.001
QE_post_	0.288 (0.11)	0.275 (0.11)	0.167 (0.06)	0.173 (0.07)	8.29^*^	.884	0.13	.007	0.89	.047
*SD* QE_total_	0.33 (0.13)	0.32 (0.09)	0.38 (0.29)	0.38 (0.25)	0.38	.020	0.35	.019	0.02	.001
*SD* QE_pre_	0.27 (0.11)	0.25 (0.08)	0.35 (0.30)	0.34 (0.28)	0.82	.044	0.89	.047	0.04	.002
*SD* QE_post_	0.16 (0.07)	0.16 (0.08)	0.08 (0.04)	0.10 (0.05)	8.73^**^	.327	1.02	.054	0.51	.027
Putting times (s)										
Address	2.92 (0.81)	2.99 (0.90)	4.79 (3.3)	4.43 (2.78)	2.79	.134	1.16	.061	2.47	.121
Swing	0.89 (0.15)	0.90 (0.16)	0.71 (0.14)	0.71 (0.15)	8.53^**^	.321	0.44	.024	0.15	.008
*SD* address	0.78 (0.60)	0.79 (0.48)	1.96 (1.47)	2.15 (1.60)	6.19^*^	.256	1.54	.079	1.13	.059
*SD* swing	0.04 (0.02)	0.06 (0.09)	0.07 (0.03)	0.20 (0.44)	1.35	.070	1.18	.062	0.66	.036

*Note*. Values were examined as average (e.g., QE_total_) and standard deviation (e.g., *SD* QE_total_) across putts.

*p ≤ .05. **p < .01.

#### 3.1.2 Eye quietness

**Figure 2:**
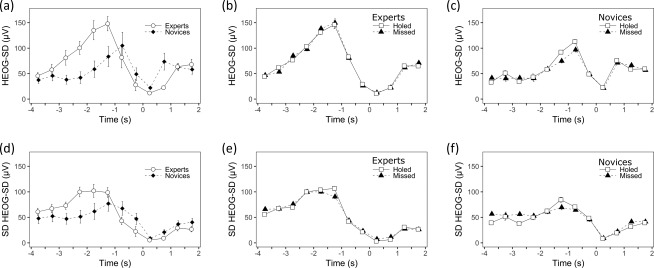
(a,b,c) Eye quietness (i.e., HEOG‐SD, µV) and (d,e,f) its variability across putts (i.e., *SD* HEOG‐SD, µV) as a function of time (s) from −4 to 2 s and either group (expert, novice) or outcome (holed, missed). HEOG‐SD is inversely related to eye quietness: lower values indicate greater quietness. (a,d) Group × Time effects. Error bars indicate between‐subjects *SE*. (b,e) Outcome × Time effects for the experts. (c,f) Outcome × Time effects for the novices. Error bars indicate within‐subject *SE* computed through normalization of the outcome factor (Cousineau, 2005)

The mean (*SE*) HEOG‐SD measures of eye quietness as a function of group, outcome, and time are illustrated in Figure 2. A consistent time‐varying cubic pattern can be seen: ocular activity increased during the movement preparation phase (‐4 to −1 s), peaking just before movement initiation (c. −1 s), before dropping, with a trough around movement execution (0 s), and then increasing again after the ball was struck (c.1 s).

The 2 Group × 2 Outcome × 12 Time ANOVA conducted on the mean HEOG‐SD revealed a main effect for time, *F*(11, 8) = 7.87, *p* = .004, λ = .085, $\eta_{p}^{2}$ = .247, and a Group × Time interaction, *F*(11, 8) = 9.95, *p* = .002, λ = .068, $\eta_{p}^{2}$ = .141. Independent samples *t* tests revealed that, compared to novices, experts had greater HEOG‐SD from −2.5 to −1.5 s and smaller HEOG‐SD from 0 to 1 s. No effects emerged for group, *F*(1, 18) = 0.96, *p* = .34, $\eta_{p}^{2}$ = .051, outcome, *F*(1, 18) = 0.51, *p* = .49, $\eta_{p}^{2}$ = .027, Group × Outcome, *F*(1, 18) = 1.11, *p* = .31, $\eta_{p}^{2}$ = .058, Outcome × Time, *F*(11, 8) = 0.65, *p* = .75, λ = .528, $\eta_{p}^{2}$ = .070, or Group × Outcome × Time, *F*(11, 8) = 0.78, *p* = .65, λ = .481, $\eta_{p}^{2}$ = .044.

The 2 Group × 2 Outcome × 12 Time ANOVA conducted on the variability of HEOG‐SD revealed a main effect for time, *F*(11, 8) = 5.24, *p* = .01, λ = .122, $\eta_{p}^{2}$ = .414, namely, a cubic (increase, decrease, increase) pattern. No effects emerged for group, *F*(1, 18) = 0.27, *p* = .61, $\eta_{p}^{2}$ = .015, outcome, *F*(1, 18) = 1.11, *p* = .31, $\eta_{p}^{2}$ = .058, Group × Outcome, *F*(1, 18) = 0.72, *p* = .41, $\eta_{p}^{2}$ = .038, Outcome × Time, *F*(11, 8) = 3.13, *p* = .06, λ = .189, $\eta_{p}^{2}$ = .115, Group × Time, *F*(11, 8) = 2.84, *p* = .07, λ = .204, $\eta_{p}^{2}$ =.139, or Outcome × Group × Time, *F*(11, 8) = 0.85, *p* = .61, λ = .462, $\eta_{p}^{2}$ = .023.

#### 3.1.3 Putting times

The mean (*SD*) putting times for each group's holed and missed putts are presented in Table 1. Experts had longer swing times and less address time variability (indicative of greater consistency across putts) than novices.

### 3.2 Relation between quiet eye and eye quietness

Quiet eye durations were negatively correlated with HEOG‐SD in both the pre‐ and postmovement initiation phases, most notably and prominently in the second before and the second after the onset of the backswing (see Table 2). As expected, these analyses confirm an inverse association between the quiet eye and eye quietness measures.

**Table 2:** Pearson's correlations between quiet eye durations (QE_pre_ and QE_post_) and eye quietness (HEOG‐SD), computed in different time intervals relative to backswing initiation

	QE_pre_ (s)		QE_post_ (s)	
HEOG‐SD (µV)	60 µV	20 µV	60 µV	20 µV
−4 to −3.5 s	‐.50^*^	‐.34	–	–
−3.5 to −3 s	‐.41	‐.21	–	–
−3 to −2.5 s	‐.38	‐.07	–	–
−2.5 to −2 s	‐.34	‐.02	–	–
−2 to −1.5 s	‐.33	‐.11	–	–
−1.5 to −1 s	‐.64^**^	‐.35	–	–
−1 to −0.5 s	‐.62^**^	‐.50^*^	–	–
−0.5 to 0 s	‐.48^*^	‐.49^*^	–	–
0 to 0.5 s	–	–	‐.91^***^	‐.80^***^
0.5 to 1 s	–	–	‐.53^*^	‐.33
1 to 1.5 s	–	–	.11	‐.18
1.5 to 2 s	–	–	.19	.29

*Note*. Only relevant comparisons (e.g., premovement initiation quiet eye with premovement initiation eye quietness) are shown. Dashes indicate that statistical tests are not meaningful for these comparisons.

*p ≤ .05. **p < .01. ***p < .001.

### 3.3 Impact of threshold level on quiet eye duration

**Figure 3:**
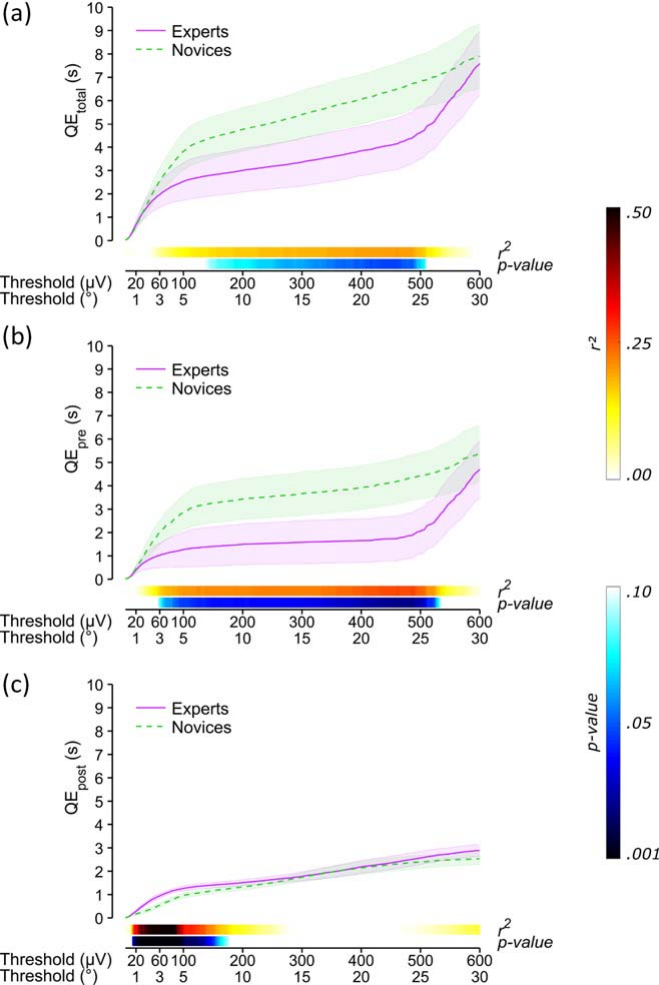
Durations (s) of (a) total (QE_total_), (b) premovement initiation (QE_pre_), and (c) postmovement initiation (QE_post_) quiet eye, as a function of threshold (µV and corresponding degrees of visual angle). The solid and dashed lines represent mean durations, respectively, for experts and novices. The two colored bars above the *x* axis indicate *r^2^* and *p* values associated with the independent samples *t* tests conducted on group differences (*df* = 18). Shaded areas represent the *SE* of each group's means and were computed using pooled estimates, hence corresponding with the independent samples *t* tests (Pfister & Janczyk, 2013)

To further explore the impact of threshold level on expert–novice differences in the quiet eye, we computed their quiet eye durations corresponding to visual angles of 0.1°–30° (2–600 µV). Importantly, experts never exhibited longer durations of QE_total_ (Figure 3a) or QE_pre_ (Figure 3b) than novices. Unexpectedly, compared to experts, novices showed longer QE_total_ durations at extremely high thresholds spanning approximately 400–500 µV (i.e., 20°–25° of visual angle) as well as longer QE_pre_ durations at high to extremely high thresholds spanning 100–500 µV (5°–25° of visual angle). Finally, experts displayed longer QE_post_ durations than novices at thresholds of 20–150 µV, corresponding to 1°–7° of visual angle, which overlap with those used in camera‐based research.

### 3.4 Correlates of performance

For experts, the percentage of holed putts was unrelated to putting times, quiet eye durations, and eye quietness, with two exceptions (see supporting information, Appendix S4). Expert performance was negatively correlated with mean HEOG‐SD in only the −2 to −1.5 s (*r* = ‐.76, *p* = .01) and 1.5 to 2 s (*r* = ‐.71, *p* = .02) bins, indicating that more putts were holed by players whose eyes were quieter within these intervals. For novices, the percentage of holed putts was unrelated to putting times, quiet eye durations, and eye quietness, with three exceptions (see Appendix S4). Novice performance was negatively correlated with the QE_total_ (*r* = ‐.63, *p* = .05) and QE_pre_ (*r* = ‐.63, *p* = .05) with the 60 µV threshold, showing that more putts were holed by players with shorter total and premovement initiation quiet eye durations. Lastly, novices' performance was positively correlated with mean HEOG‐SD in just the −0.5 to 0 s bin (*r* = .73, *p* = .02), indicating that more putts were holed by participants whose eyes were less quiet within this interval.

### 3.5 Relations between putting times and ocular activity

Pearson's correlations were computed between premovement initiation ocular activity and address times as well as between postmovement initiation ocular activity and swing times (Appendix S5). These analyses showed that address times were unrelated to quiet eye and eye quietness. Crucially, ocular activity after backswing initiation was associated with the duration of the swing time. Namely, swing times correlated positively (*r* = .52, *p* = .02) with QE_post_ (60 µV threshold) and negatively (*r* = ‐.63, *p* = .003) with HEOG‐SD measured 0.5 to 1 s after swing initiation. Thus, participants with longer putting strokes were characterized by longer postmovement initiation quiet eye durations and greater quietness around impact with the ball.

## 4 DISCUSSION

This report explored the utility of EOG in the study of ocular activity during performance of a motor skill. Specifically, we conducted the first analysis of the effects of expertise on both pre‐ and postmovement initiation quiet eye components in golf putting. We also developed a new measure of movement‐related ocular activity in the form of eye quietness, inversely related with quiet eye duration. The analyses generated a number of novel findings, shedding light on the mechanisms underpinning the relationship between ocular activity and motor behavior. These effects are discussed below.

### 4.1 Quiet eye

A primary aim was to quantify both pre‐ and postmovement initiation components of the quiet eye using EOG, and a secondary aim was to examine the impact of threshold level on quiet eye duration. We examined quiet eye durations at threshold levels corresponding with 3° and 1° of visual angle (i.e., respectively, 60 µV and 20 µV; Shackel, 1967), typically used in the quiet eye literature. As expected, quiet eye durations were longer with 3° (around 2 s for experts and 2.5 s for novices) than with 1° (around 0.7 s for experts and 0.6 s for novices). However, contrary to expectations, total quiet eye duration (i.e., QE_total_) did not distinguish experts from novices. Interestingly, group differences emerged when the quiet eye period was broken down relative to the moment of movement initiation. Compared to novices, experts showed a shorter, albeit not significant, premovement initiation quiet eye (i.e., QE_pre_), and longer postmovement initiation quiet eye (i.e., QE_post_; Table 1). Further analyses revealed that there was no threshold setting at which experts had a longer total quiet eye (QE_total_) or premovement initiation quiet eye (QE_pre_) than novices. Instead, experts only had shorter durations than novices, although this difference was significant only for threshold levels that were larger than typically used in the literature (Figure 3a,b). These analyses also confirmed that experts showed a longer postmovement initiation quiet eye (QE_post_) than novices (Figure 3c).

That the postmovement initiation component of the quiet eye was more sensitive than the premovement initiation component of the quiet eye in revealing differences in putting performance in experienced golfers is consistent with two previous studies (Vine et al., 2013; Vine, Lee, Walters‐Symons, & Wilson, 2015). First, Vine et al. (2013) tested 50 expert golfers (mean handicap 3.6) as they putted balls to a 5‐ft (i.e., 1.5‐m) distant hole. They examined the quiet eye in different phases of the putt and found that, compared to missed putts, holed putts were characterized by a longer postmovement initiation quiet eye, whereas the premovement initiation quiet eye was not different. Second, Vine et al. (2015) tested 27 experienced golfers (mean handicap 5.8) as they putted balls to a 10‐ft (i.e., 3‐m) distant hole. Participants' view of the ball was occluded either before or after movement initiation, through a liquid crystal glass panel—positioned above the ball—turning opaque. The authors found that, compared to a no‐occlusion condition, performance was impaired by postmovement initiation occlusion but not by premovement initiation occlusion. Taken together, these findings were interpreted as evidence that visual information was actively processed only after movement initiation (i.e., during the execution of the movement), suggesting that postmovement initiation quiet eye was involved in the online control of movement (Vine et al., 2015). However, it has to be noted that this interpretation may not apply to novices (Causer et al., 2017).

To our knowledge, this is the first golf putting study to separately examine quiet eye durations before and after movement initiation in an expert–novice design. The fact that effects of expertise, as well as of performance, emerged only after movement initiation (i.e., when movement preprogramming is completed) raises doubts on the interpretation of the quiet eye as correlate of motor programming (Vickers, 1996). Further mechanistic psychophysiological research is needed to clarify this issue. The EOG methodology developed here offers a promising tool to permit such research.

### 4.2 Eye quietness

Our primary purposes here were to develop a novel time‐based EOG measure of eye quietness, and to evaluate its validity by assessing correlations with our measure of quiet eye. We examined ocular activity as a function of time by computing the variability (standard deviation) of the EOG signal in short intervals (500 ms). This index allowed us to evaluate not only how long the eyes remained “quiet” but also how “quiet” the eyes were for intervals overlapping the quiet eye period. Time‐varying statistical analyses revealed that eye quietness fluctuated over time, decreasing prior to movement initiation, increasing around movement execution, and then finally decreasing after movement completion (Figure 2). It is interesting to note that the eyes were quietest immediately after movement initiation. Group differences emerged in the second after the ball was struck, which were times that roughly overlapped movement execution, when experts kept their eyes quieter compared to novices. Experts also showed more ocular activity than novices around 2 s prior to movement initiation, perhaps indicative of them taking a final look at the hole consistently at that time (Appendix S1).

As expected, these results for eye quietness broadly match those for quiet eye durations (i.e., greater postmovement initiation eye quietness corresponded with longer quiet eye duration), whereas less premovement initiation eye quietness corresponded with shorter quiet eye duration. Indeed, further analyses confirmed our hypothesis that eye quietness would correlate negatively with quiet eye durations, particularly at times immediately preceding and following movement initiation (Table 2), concurrently validating eye quietness as a measure of ocular activity. This new measure of movement‐related ocular activity promises to be especially useful for future multimethod psychophysiological investigations, where it will allow time‐synchronized analyses of ocular activity with other signals of interest such as EEG. This research is needed to shed further light on the mechanisms that underpin motor performance.

### 4.3 Consistency

We hypothesized that consistency of ocular activity across putts would be greater in experts than novices. The analyses of the variability (standard deviation) across putts for quiet eye durations and address times revealed that experts generally showed greater consistency than novices (Table 1), in line with classic models of motor skill acquisition (e.g., Fitts & Posner, 1967). Such group differences are also noticeable from inspection of the individual EOG waveforms (Appendix S1). This consistency effect may reflect the fact that experts have a more consolidated and permanent putting routine than novices, involving address time as well as ocular behavior.

### 4.4 Performance effects

We predicted, based on extant literature, that quiet eye durations would be longer, and eye quietness greater, for holed compared to missed putts. No differences emerged comparing holed and missed putts for all measures of ocular activity and movement times (Table 1). However, performance effects were detected when we considered the variability of participants separately within each group (Appendix S4). Correlation analyses revealed that, among the novices, those with shorter quiet eye durations (total and premovement initiation quiet eye) and less eye quietness immediately before movement initiation holed more putts. This finding is in contrast with the view that longer quiet eye leads to better performance (Vine et al., 2014) but is consistent with the finding of this study that, on average, experts showed shorter total and premovement initiation quiet eye durations than novices. In other words, the novices that showed ocular activity more similar to that of the experts performed better. For the experts, those with greater eye quietness 2 s before and after movement initiation holed more putts. These findings may indicate that better performance was achieved by experts who moved their eyes less before putting (perhaps because they did not need to look at the hole as often, due to superior ability to read the green) and after movement completion (perhaps because the ball ended in the hole more often or was rolling directly to the target and, therefore, there was less need to track it in some other spatial locations).

### 4.5 Kinematic hypothesis

Our final prediction was that longer swing durations would be associated with longer postmovement initiation quiet‐eye durations and greater eye quietness during swing execution. Experts took around 200 ms longer than novices to swing the putter and hit the ball (Table 1). This finding is consistent with studies that have examined expert–novice differences for movement kinematics in golf putting (e.g., Delay et al., 1997). The fact that experts showed less ocular activity (i.e., greater eye quietness) than novices at times overlapping the execution of the swing suggests a connection between ocular activity and movement duration. Further analyses confirmed that swing duration correlated positively with the duration of the postmovement initiation quiet eye (i.e., QE_post_) and negatively with eye quietness 0.5 to 1 s after movement initiation (Appendix S5). These results suggest that group differences for postmovement initiation ocular activity, discussed above, may be explained, at least in part, by the fact that experts took longer to perform the movement compared to novices. This provides promising evidence for the kinematic hypothesis as a mechanism to explain individual differences in gaze behavior. Specifically, keeping a quiet eye during the swing may enhance postural stability and permit a smoother movement execution. Alternatively, a longer and smoother technique may prompt a longer quiet eye and greater eye quietness during the swing. Indeed, compared to novices, experts swing the putter with lower variability in the axis perpendicular to the putting line (Cooke et al., 2014; Sim & Kim, 2010). The hypothesis that quiet eye represents a correlate of stability during the movement execution is worthy of more direct examination by future research. For example, studies could manipulate features of the movement (e.g., by varying putting distance; Delay et al., 1997) and examine their impact on putting kinematics (e.g., swing duration, smoothness, stability) as well as ocular activity to provide more direct tests of the kinematic hypothesis.

### 4.6 Limitations and directions

The findings of this study must be considered in light of some limitations. First, the EOG measures eye movements relative to the head and, therefore, head movements are confounded with eye movements (Young & Sheena, 1975). For example, a shift in gaze to the left with a still head generates an EOG signal that looks similar to a head movement to the right with a still gaze: in both cases, the eyes move to the left but indicate a saccade and a fixation, respectively. In the present study, we were able to observe that all participants rested their head above the ball during the final seconds before and during movement. Nonetheless, it would be better for future studies to directly measure head movements to control for this source of bias. Second, we computed quiet eye durations using the EOG signal from only the horizontal channel. However, to increase reliability and generalizability of this method to a variety of movement tasks, future studies could develop better algorithms that combine information from both the vertical and horizontal EOG channels. Third, we acknowledge that the equivalence of 20 µV on the horizontal EOG signal with 1° of visual angle is an oversimplification (Shackel, 1967). In fact, the corneoretinal potential that generates the electrical activity that is detected by the EOG changes according to ambient luminance (Young & Sheena, 1975). This effect does not bias our findings because light conditions were kept constant throughout testing and adaptations to luminance changes occur over the course of several minutes (Marmor et al., 2011). Nonetheless, we recommend that researchers calibrate the EOG signal to visual stimuli placed at a known distance in visual angles, for each participant, to account for interindividual variability. Fourth, differently from eye tracking, the EOG does not provide spatial information on gaze location. For example, we could not distinguish whether, during the quiet eye period, the gaze was on the target (i.e., the ball) or on a location near the target (e.g., putting surface, putter head). In light of the fact that less skilled golfers make more fixations than more skilled golfers prior to backswing initiation (e.g., Vickers, 1992), this limitation may explain why our finding that novices had longer premovement initiation quiet eye durations than experts departs from what is reported in most other quiet eye studies. Fifth, experts putted to a smaller hole than novices. This ensured that the two groups achieved a similar number of holed and missed putts. However, the novelty of putting to a smaller hole may have affected experts' preparatory processes and their ocular behavior. Finally, EOG can provide complementary information to eye tracking. Therefore, future studies would do well to concurrently record eye tracking and EOG to combine the greater spatial resolution of the former with the greater temporal resolution of the latter.

### 4.7 Conclusion

This study demonstrated the utility of new EOG‐based methods as complementary techniques to camera‐based eye tracking to assess ocular activity during execution of motor skills. By incorporating EOG methods, quiet eye research should benefit from the body of knowledge produced by psychophysiological research about expertise and performance in motor control (for review of studies, see Cooke, 2013; Hatfield et al., 2004). This interdisciplinarity should provide novel viewpoints on pressing issues, such as the efficiency paradox (Mann, Wright, & Janelle, 2016), questioning the function of a longer quiet eye when most psychomotor indices, including those of brain activity, indicate that expertise is associated with quieting of task‐irrelevant activity and enhancement of task‐relevant activity (e.g., Gallicchio, Cooke, & Ring, 2017). The evidence garnered here favors a more parsimonious explanation for previously identified expert–novice differences in quiet eye duration; they could simply reflect experts' better and smoother technique.

## Supporting information

Additional Supporting Information may be found online in the supporting information tab for this article.

**Appendix S1**

**Appendix S2**

**Appendix S3**

**Appendix S4**

**Appendix S5**

## Notes

1 Golf handicap represents the number of strokes taken in relation to the number of strokes expected to be taken to complete a round of golf. It indexes golfers' ability: the lower the value, the better the player.

2 The influence of sampling rate on ocular activity has been investigated by Helsen, Starkes, Elliot, and Ricker (1998). They sampled ocular activity at 60 and 120 Hz using camera‐based eye tracking while participants performed a finger movement task. They found that the two sampling rates produced different results for saccade durations but not for fixation durations.
