# Supplementary material for: Assessing ocular activity during performance of motor skills using electrooculography
Source: Psychophysiology. 2018 Feb 9;55(7):e13070. doi: 10.1111/psyp.13070 (PMC6849535; doi:10.1111/psyp.13070)
Supplement: Supplementary file 1 — Appendix S1 [file PSYP-55-na-s001.pdf]

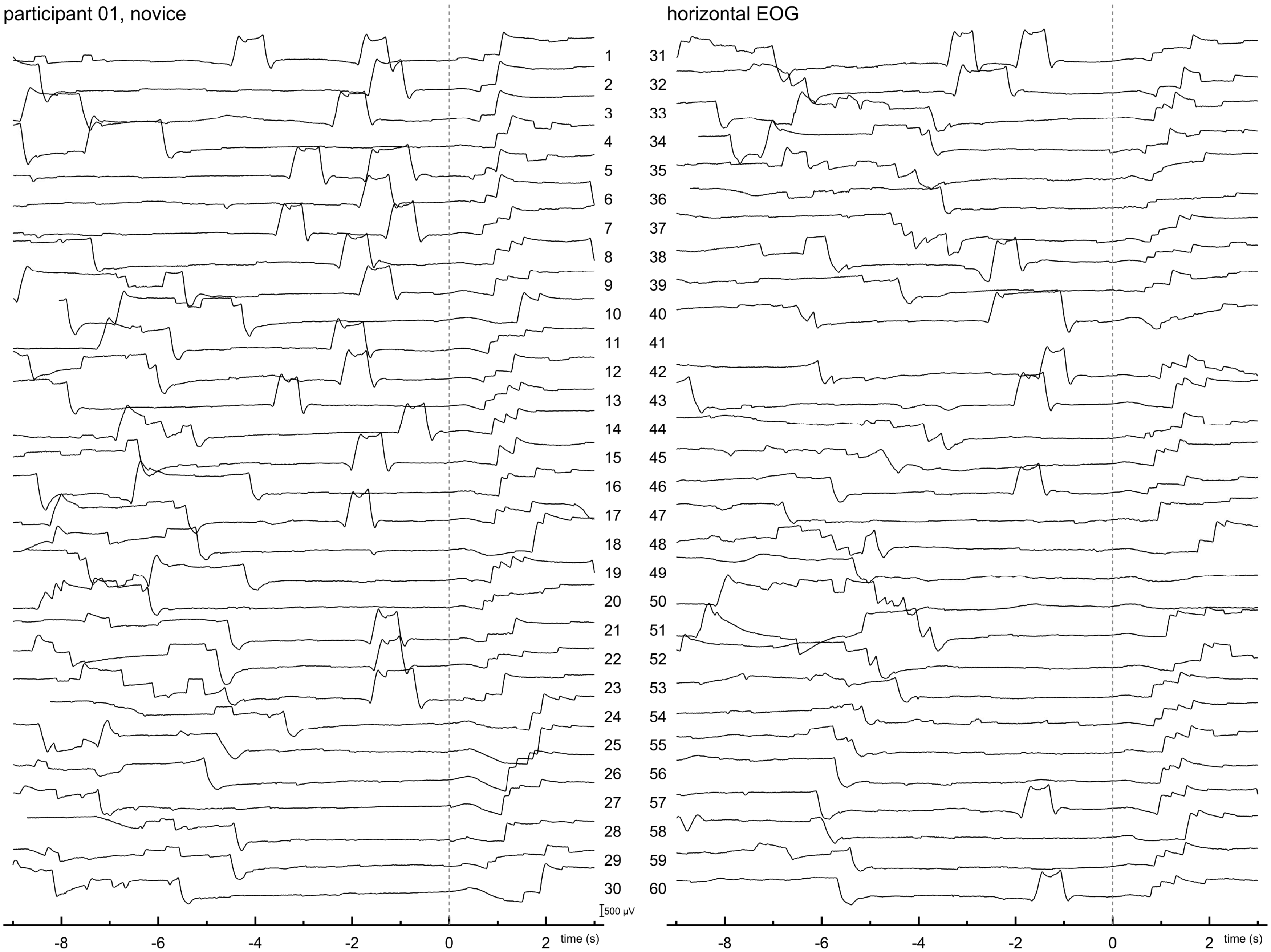

participant 01, novice
horizontal EOG
1 2 3 4 5 6 7 8 9 10 11 12 13 14 15 16 17 18 19 20 21 22 23 24 25 26 27 28 29 30
31 32 33 34 35 36 37 38 39 40 41 42 43 44 45 46 47 48 49 50 51 52 53 54 55 56 57 58 59 60
500 μV
-8 -6 -4 -2 0 2
time (s)

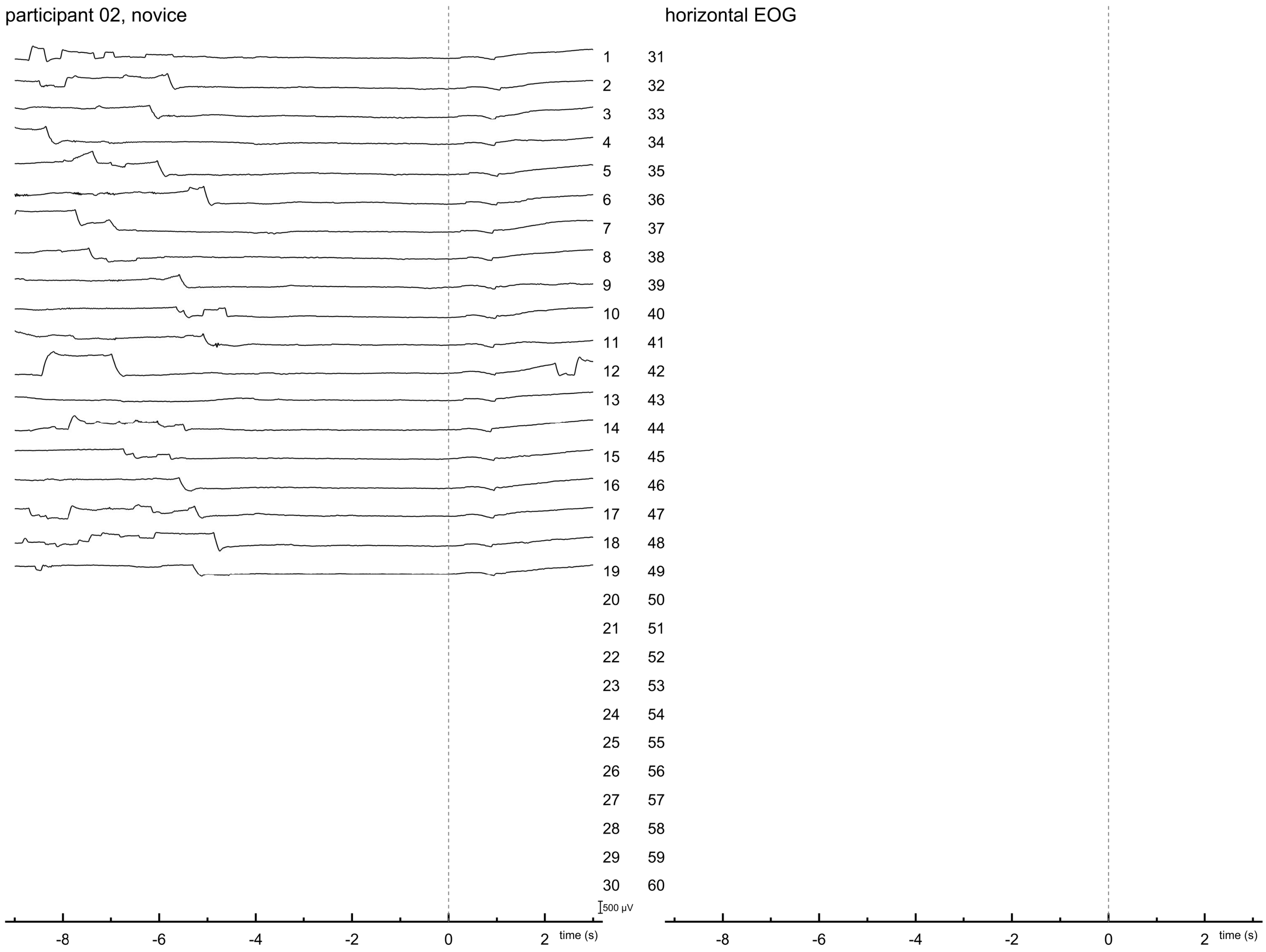
participant 02, novice
horizontal EOG
1
2
3
4
5
6
7
8
9
10
11
12
13
14
15
16
17
18
19
20
21
22
23
24
25
26
27
28
29
30
31
32
33
34
35
36
37
38
39
40
41
42
43
44
45
46
47
48
49
50
51
52
53
54
55
56
57
58
59
60
500 µV
-8
-6
-4
-2
0
2
time (s)
-8
-6
-4
-2
0
2
time (s)

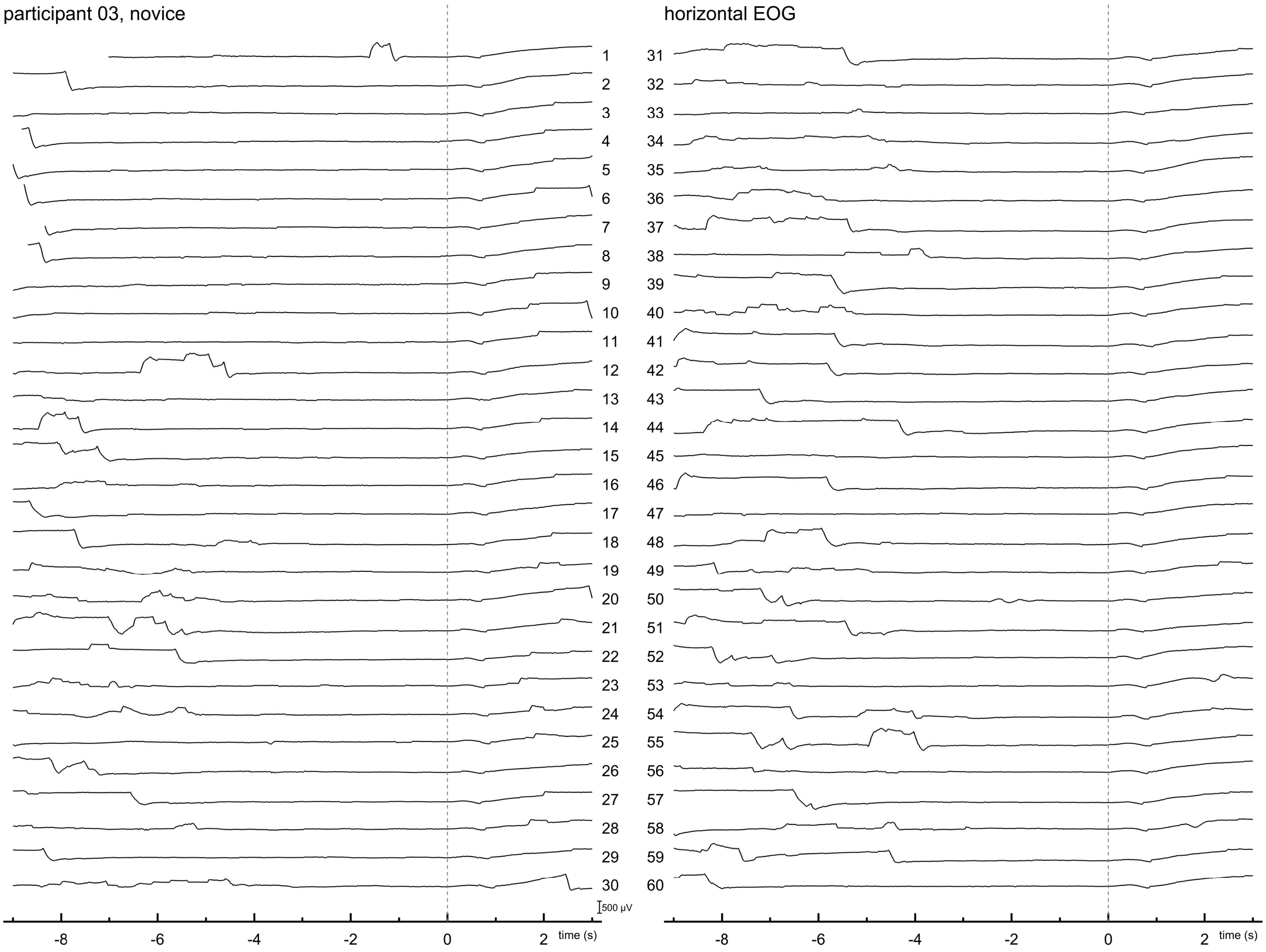
participant 03, novice
horizontal EOG
1
2
3
4
5
6
7
8
9
10
11
12
13
14
15
16
17
18
19
20
21
22
23
24
25
26
27
28
29
30
31
32
33
34
35
36
37
38
39
40
41
42
43
44
45
46
47
48
49
50
51
52
53
54
55
56
57
58
59
60
500 µV
-8
-6
-4
-2
0
2
time (s)
-8
-6
-4
-2
0
2
time (s)

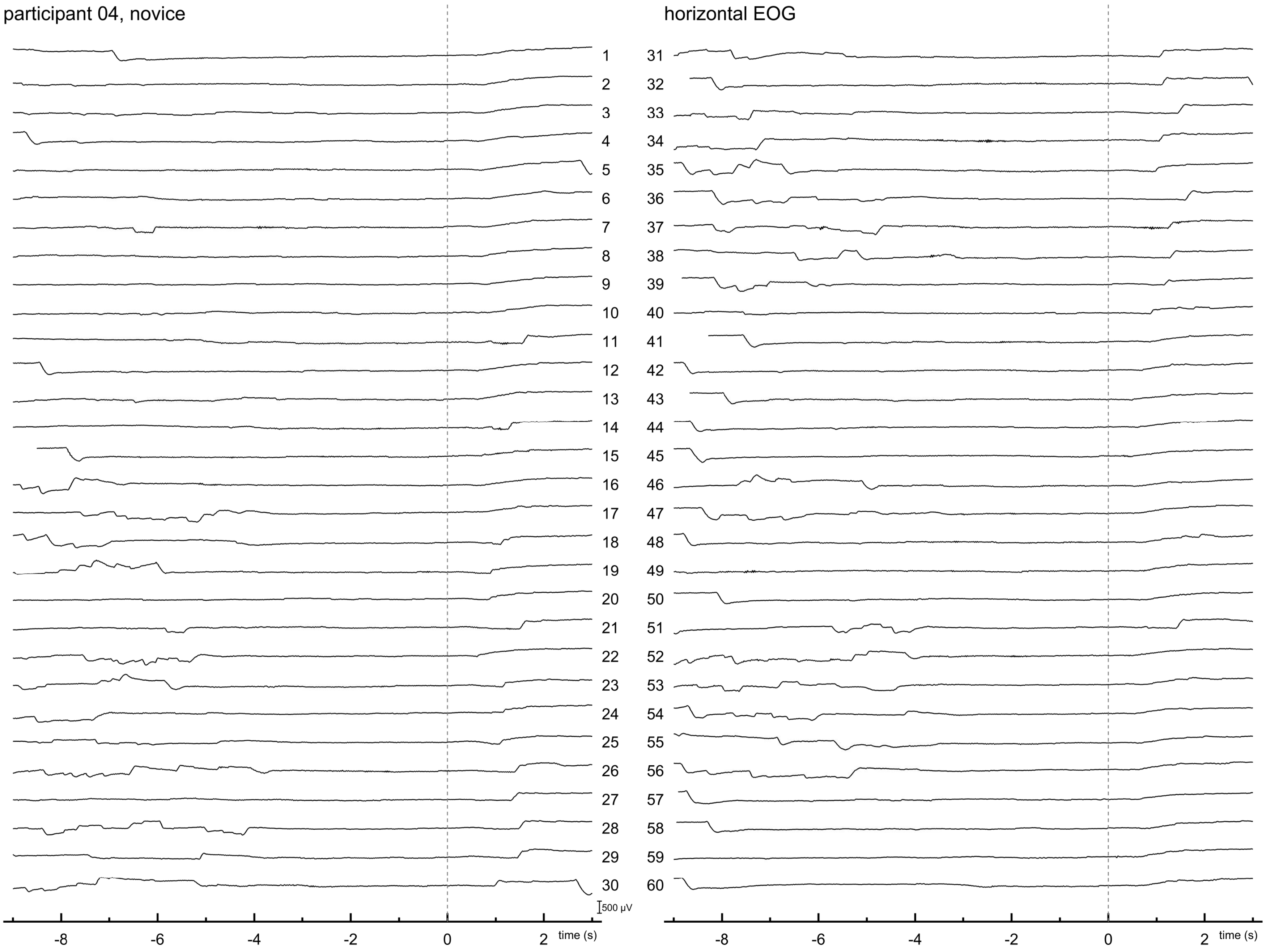

participant 04, novice
horizontal EOG
1 2 3 4 5 6 7 8 9 10 11 12 13 14 15 16 17 18 19 20 21 22 23 24 25 26 27 28 29 30
31 32 33 34 35 36 37 38 39 40 41 42 43 44 45 46 47 48 49 50 51 52 53 54 55 56 57 58 59 60
500 µV
-8 -6 -4 -2 0 2 time (s)
-8 -6 -4 -2 0 2 time (s)

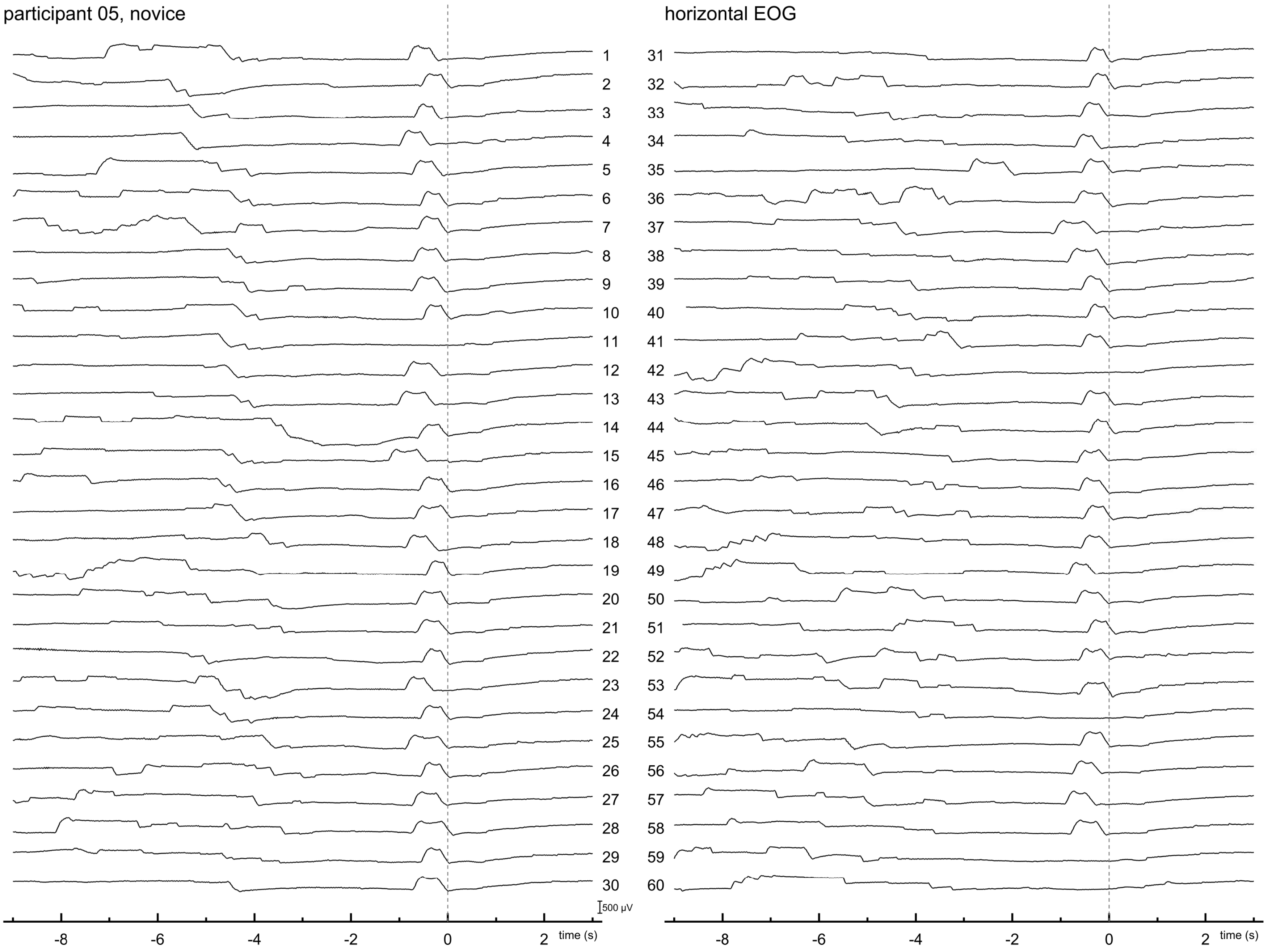
participant 05, novice
horizontal EOG
500 µV
time (s)
-8
-6
-4
-2
0
2

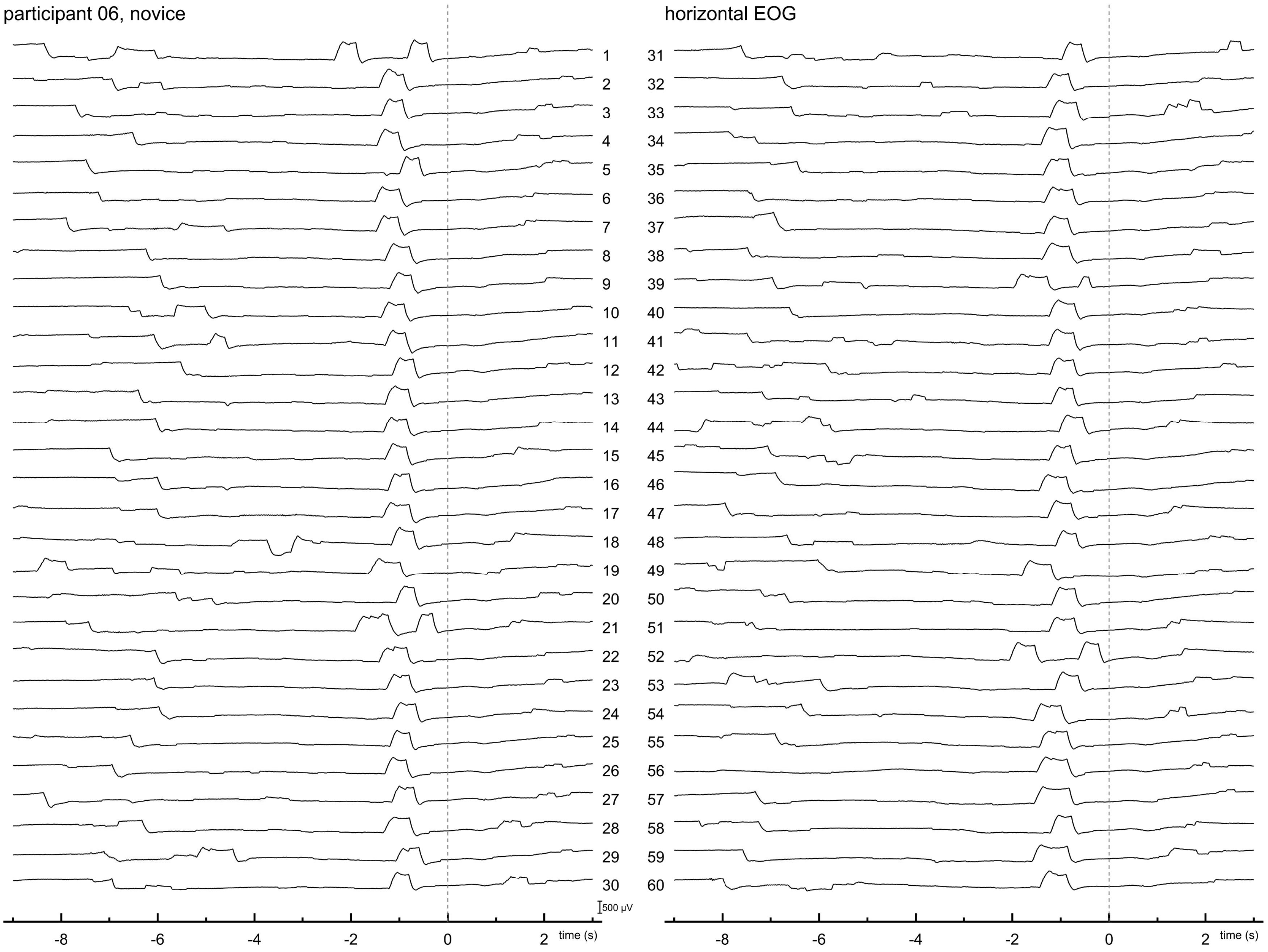

participant 06, novice
horizontal EOG
1
2
3
4
5
6
7
8
9
10
11
12
13
14
15
16
17
18
19
20
21
22
23
24
25
26
27
28
29
30
31
32
33
34
35
36
37
38
39
40
41
42
43
44
45
46
47
48
49
50
51
52
53
54
55
56
57
58
59
60
500 µV
-8
-6
-4
-2
0
2
time (s)

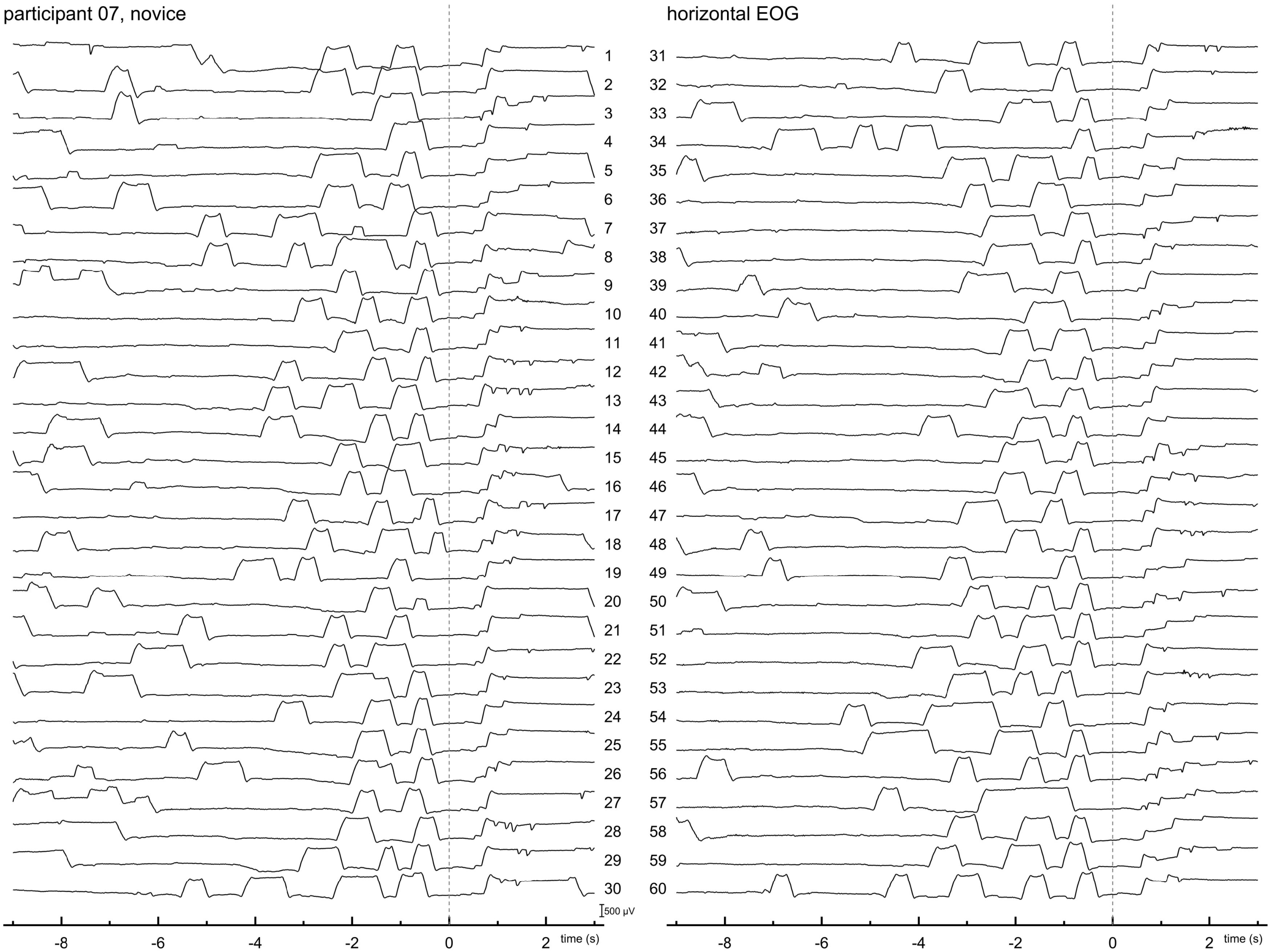
participant 07, novice
horizontal EOG
1
2
3
4
5
6
7
8
9
10
11
12
13
14
15
16
17
18
19
20
21
22
23
24
25
26
27
28
29
30
31
32
33
34
35
36
37
38
39
40
41
42
43
44
45
46
47
48
49
50
51
52
53
54
55
56
57
58
59
60
500 µV
-8
-6
-4
-2
0
2
time (s)

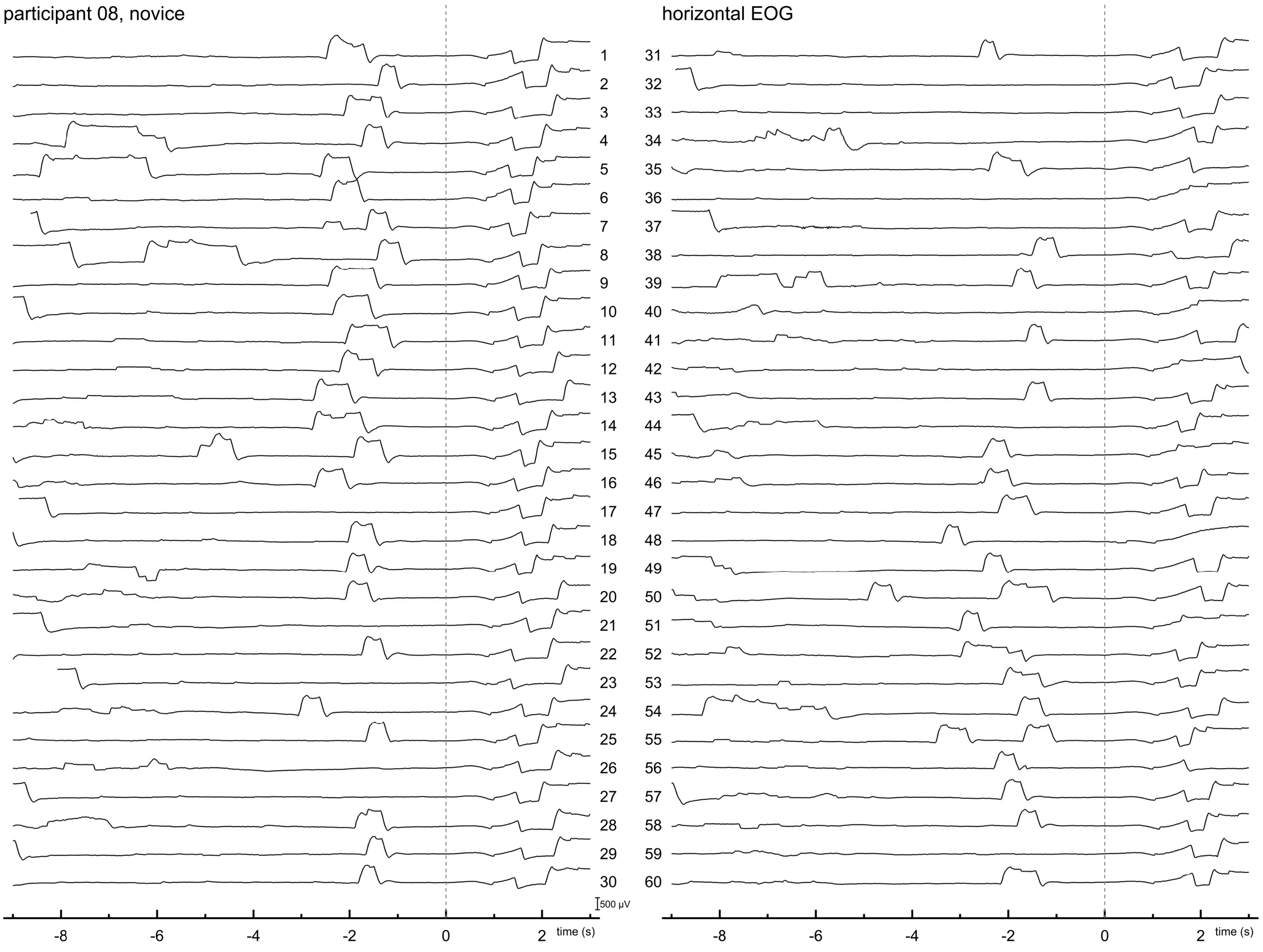
participant 08, novice
horizontal EOG
1 2 3 4 5 6 7 8 9 10 11 12 13 14 15 16 17 18 19 20 21 22 23 24 25 26 27 28 29 30
31 32 33 34 35 36 37 38 39 40 41 42 43 44 45 46 47 48 49 50 51 52 53 54 55 56 57 58 59 60
500 µV
-8 -6 -4 -2 0 2 time (s)
-8 -6 -4 -2 0 2 time (s)

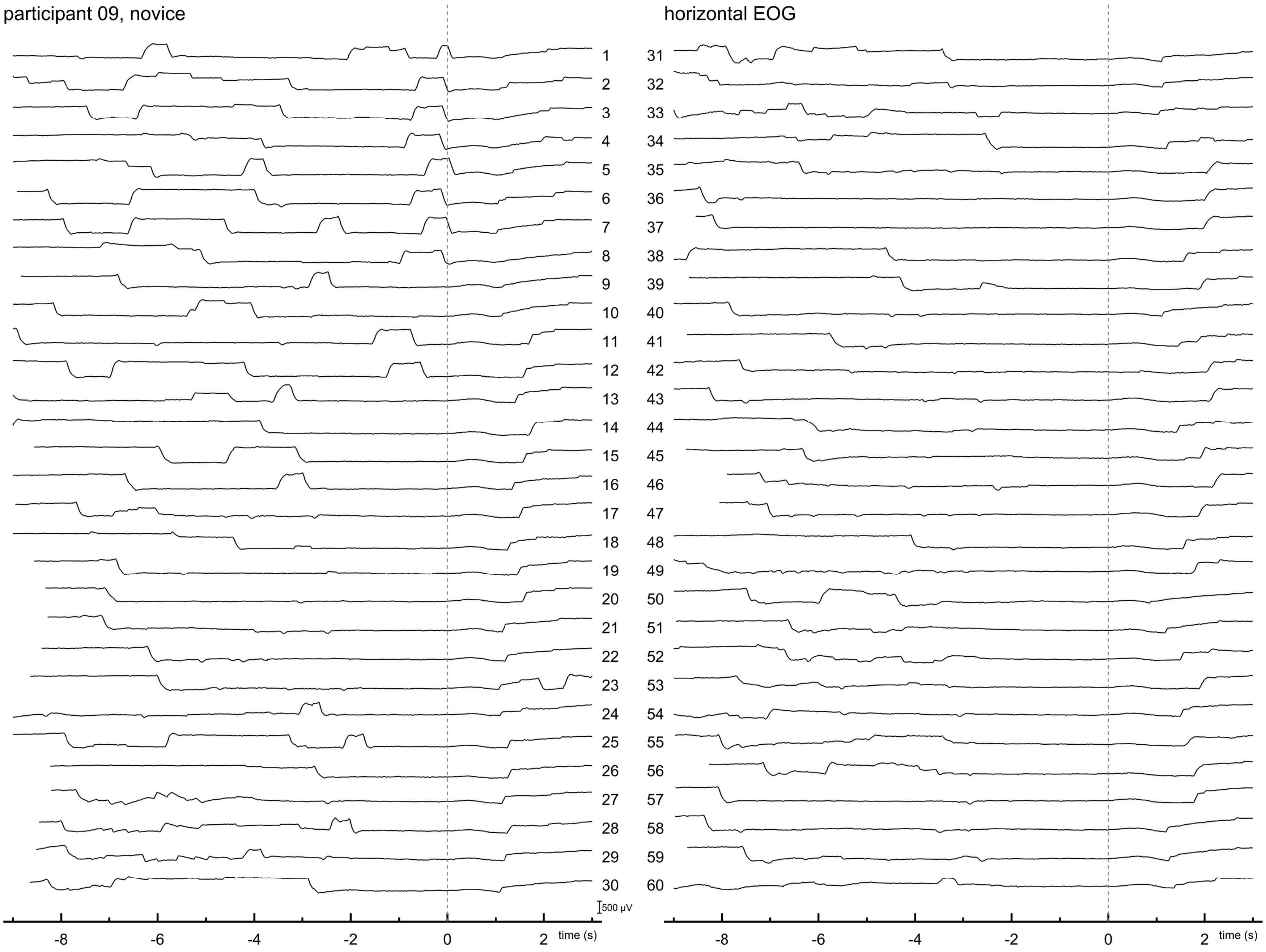
participant 09, novice
horizontal EOG
1
2
3
4
5
6
7
8
9
10
11
12
13
14
15
16
17
18
19
20
21
22
23
24
25
26
27
28
29
30
31
32
33
34
35
36
37
38
39
40
41
42
43
44
45
46
47
48
49
50
51
52
53
54
55
56
57
58
59
60
500 µV
-8
-6
-4
-2
0
2
time (s)

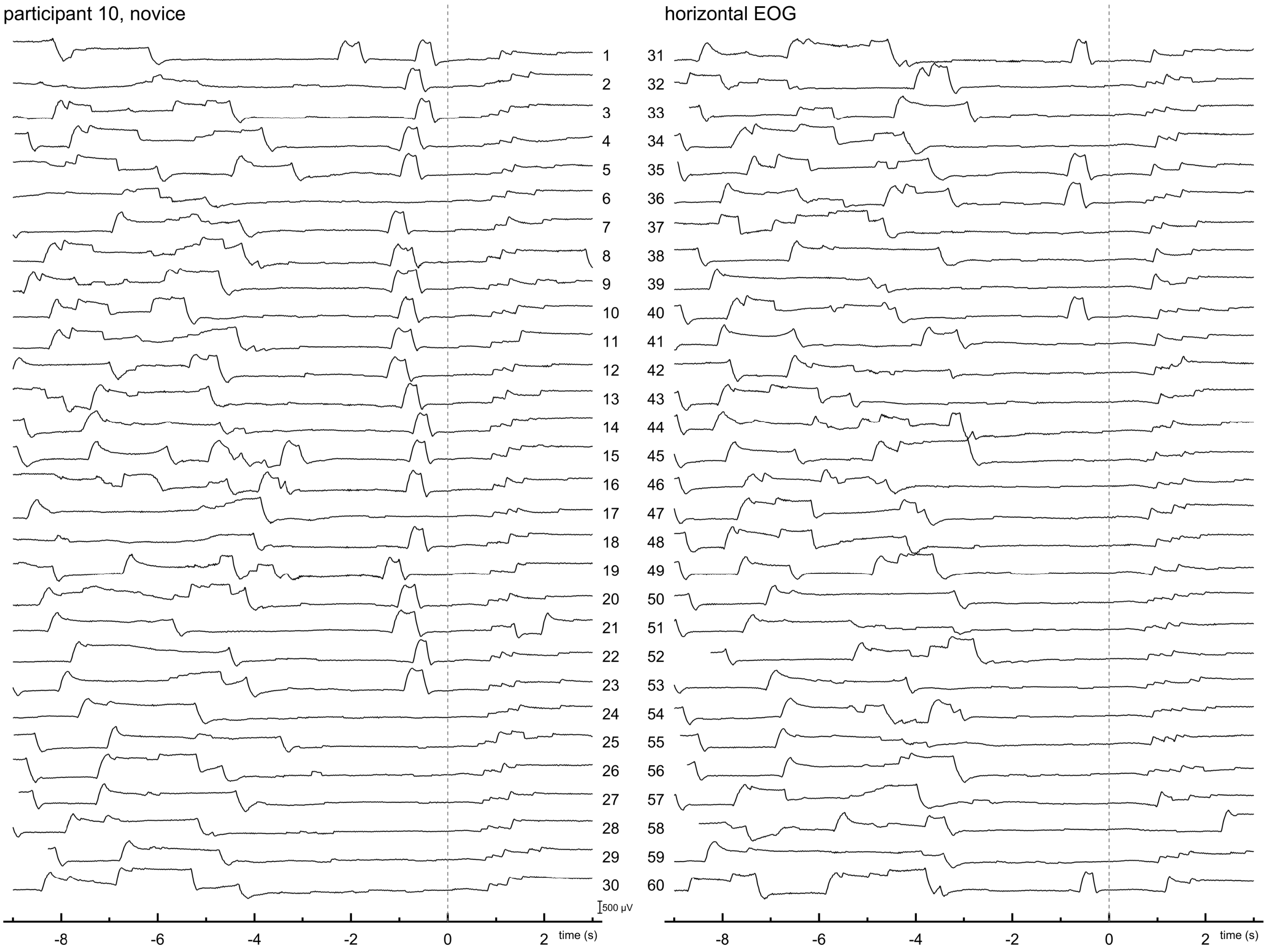
participant 10, novice
horizontal EOG
1
2
3
4
5
6
7
8
9
10
11
12
13
14
15
16
17
18
19
20
21
22
23
24
25
26
27
28
29
30
31
32
33
34
35
36
37
38
39
40
41
42
43
44
45
46
47
48
49
50
51
52
53
54
55
56
57
58
59
60
500 µV
-8
-6
-4
-2
0
2
time (s)

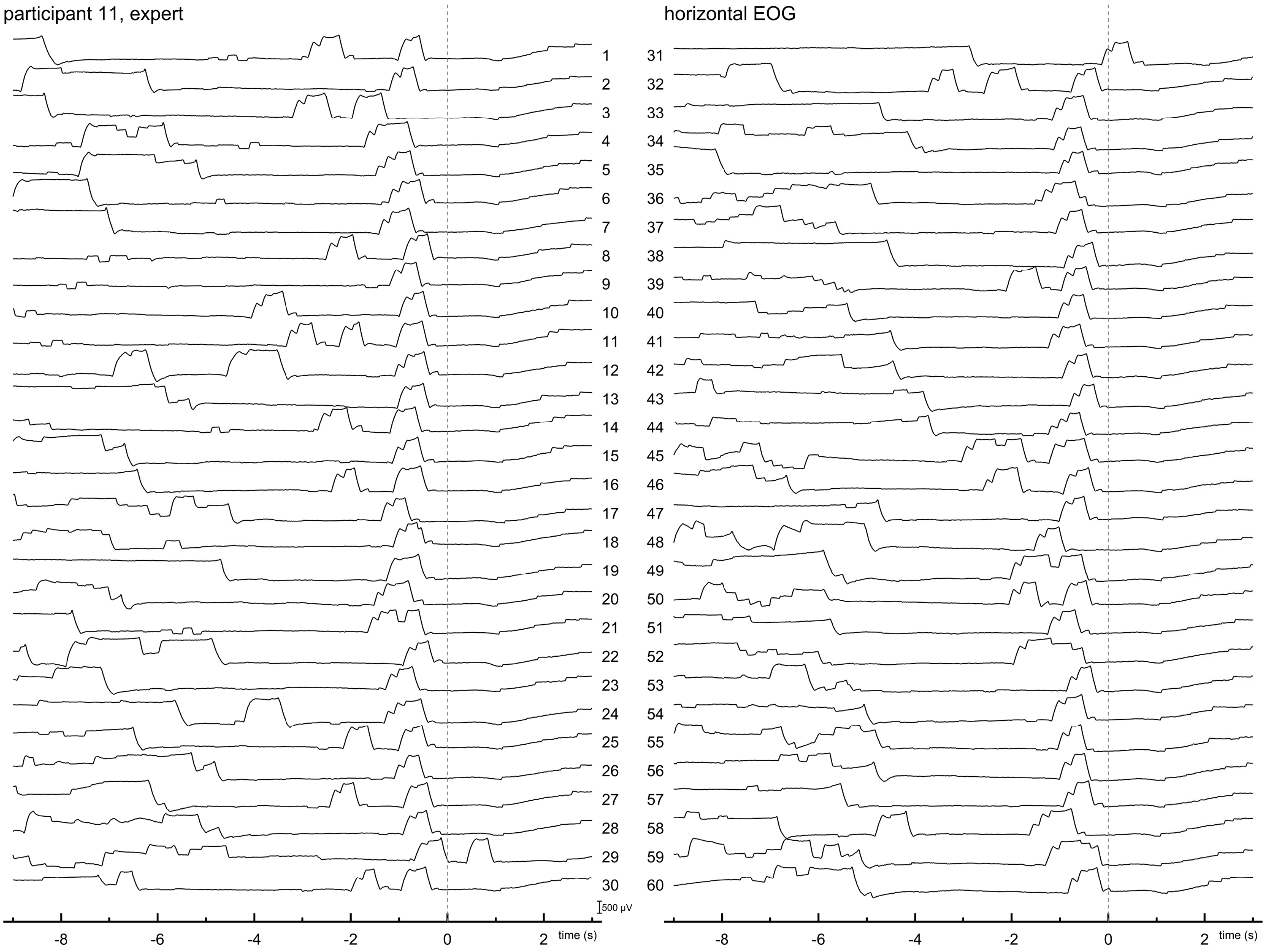
participant 11, expert
horizontal EOG
1
2
3
4
5
6
7
8
9
10
11
12
13
14
15
16
17
18
19
20
21
22
23
24
25
26
27
28
29
30
31
32
33
34
35
36
37
38
39
40
41
42
43
44
45
46
47
48
49
50
51
52
53
54
55
56
57
58
59
60
500 µV
-8
-6
-4
-2
0
2
time (s)
-8
-6
-4
-2
0
2
time (s)

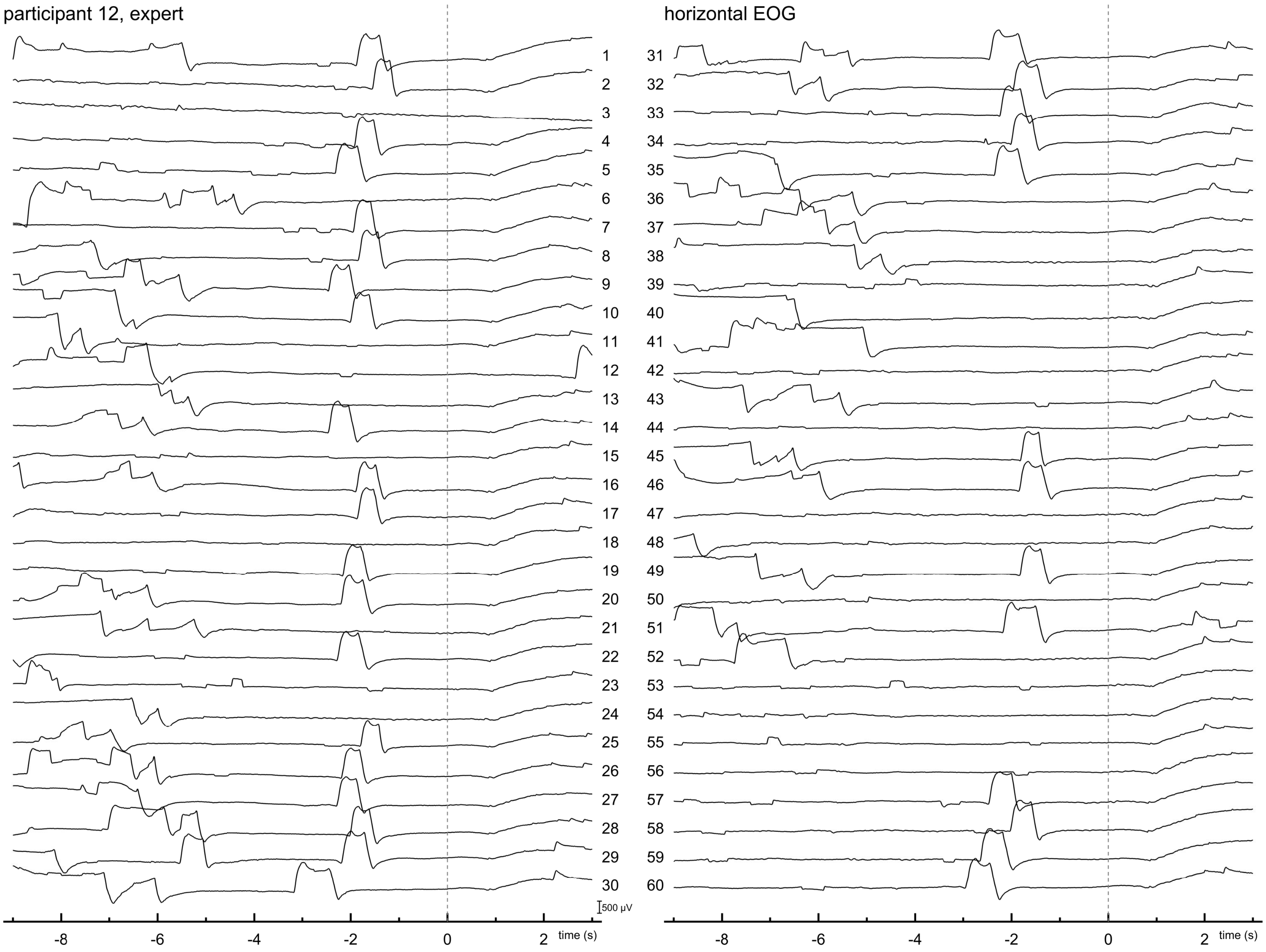

participant 12, expert
horizontal EOG
1
2
3
4
5
6
7
8
9
10
11
12
13
14
15
16
17
18
19
20
21
22
23
24
25
26
27
28
29
30
31
32
33
34
35
36
37
38
39
40
41
42
43
44
45
46
47
48
49
50
51
52
53
54
55
56
57
58
59
60
500 µV
-8
-6
-4
-2
0
2
time (s)

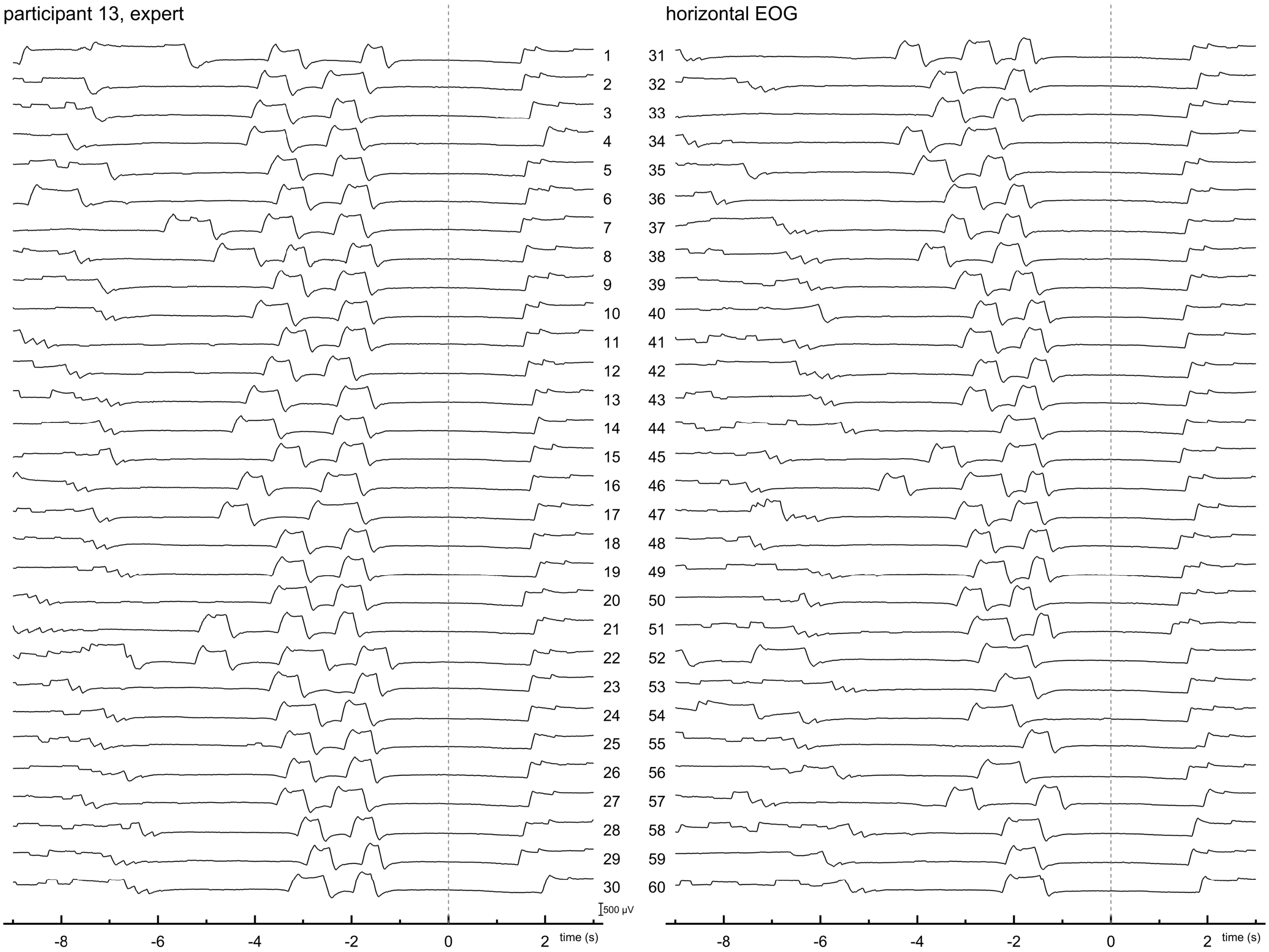
participant 13, expert
horizontal EOG
1
2
3
4
5
6
7
8
9
10
11
12
13
14
15
16
17
18
19
20
21
22
23
24
25
26
27
28
29
30
31
32
33
34
35
36
37
38
39
40
41
42
43
44
45
46
47
48
49
50
51
52
53
54
55
56
57
58
59
60
500 µV
-8
-6
-4
-2
0
2
time (s)
-8
-6
-4
-2
0
2
time (s)

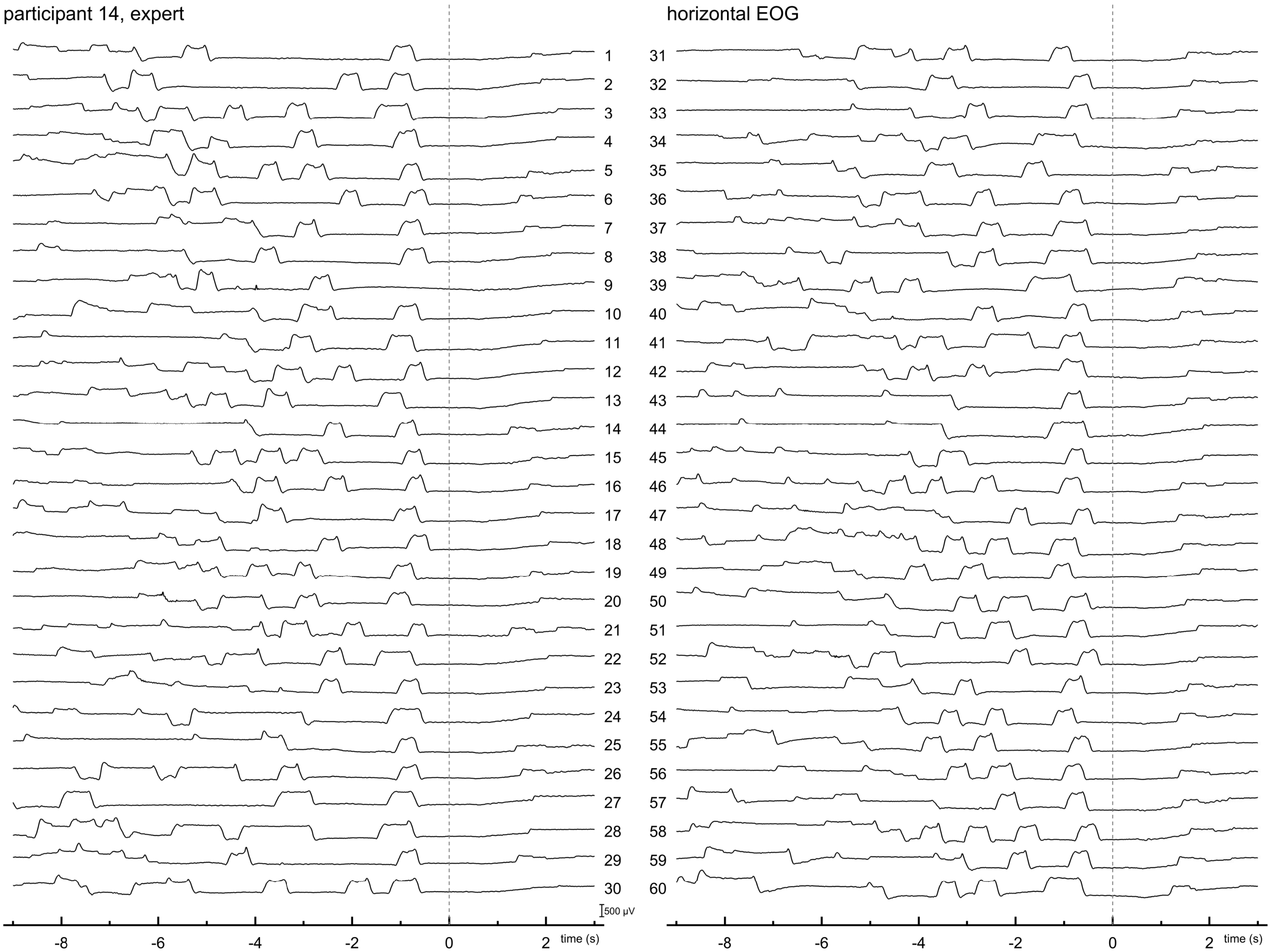
participant 14, expert
horizontal EOG
1 2 3 4 5 6 7 8 9 10 11 12 13 14 15 16 17 18 19 20 21 22 23 24 25 26 27 28 29 30
31 32 33 34 35 36 37 38 39 40 41 42 43 44 45 46 47 48 49 50 51 52 53 54 55 56 57 58 59 60
500 µV
-8 -6 -4 -2 0 2
time (s)

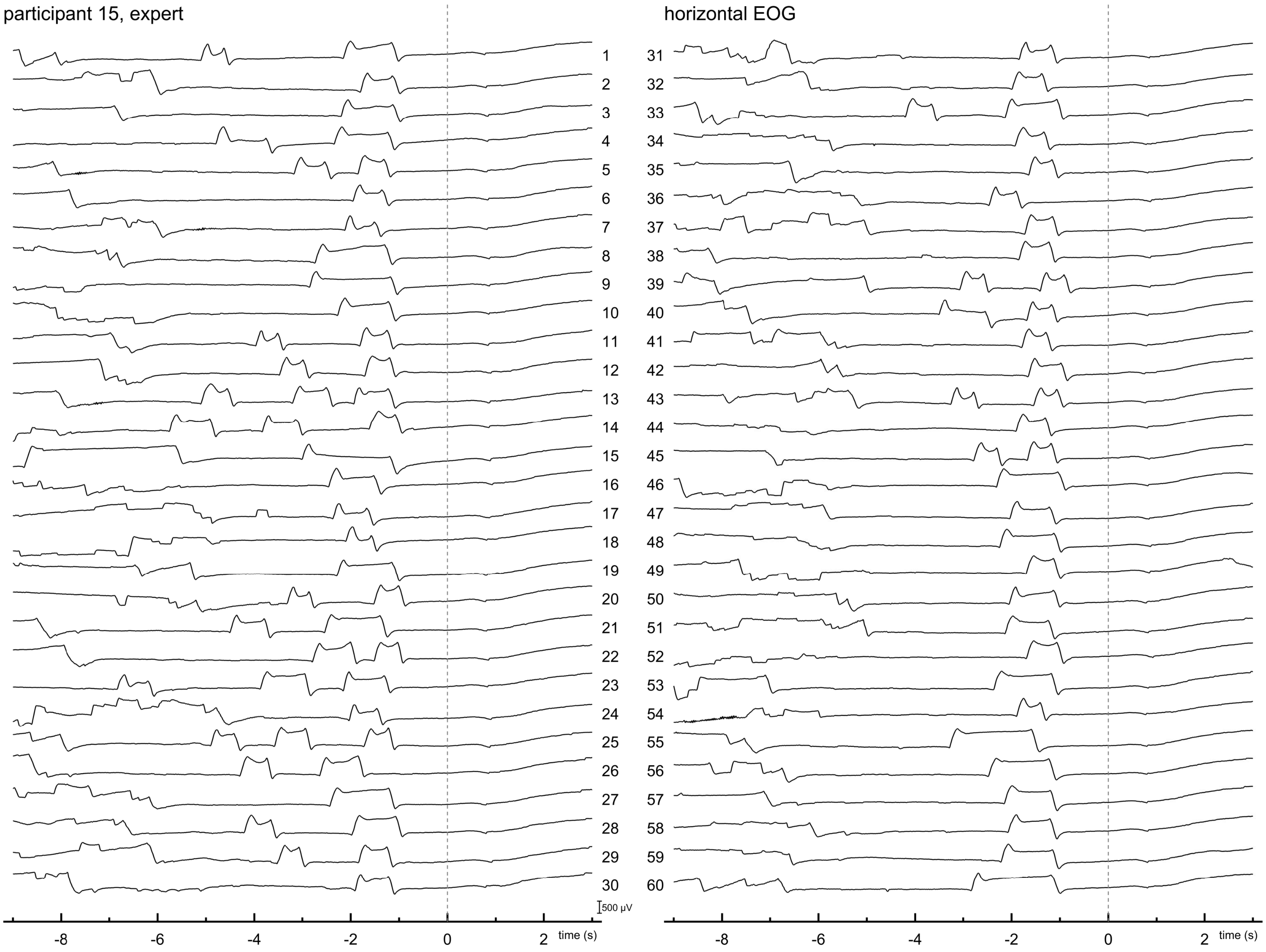
participant 15, expert
horizontal EOG
1 2 3 4 5 6 7 8 9 10 11 12 13 14 15 16 17 18 19 20 21 22 23 24 25 26 27 28 29 30
31 32 33 34 35 36 37 38 39 40 41 42 43 44 45 46 47 48 49 50 51 52 53 54 55 56 57 58 59 60
500 µV
-8 -6 -4 -2 0 2 time (s)
-8 -6 -4 -2 0 2 time (s)

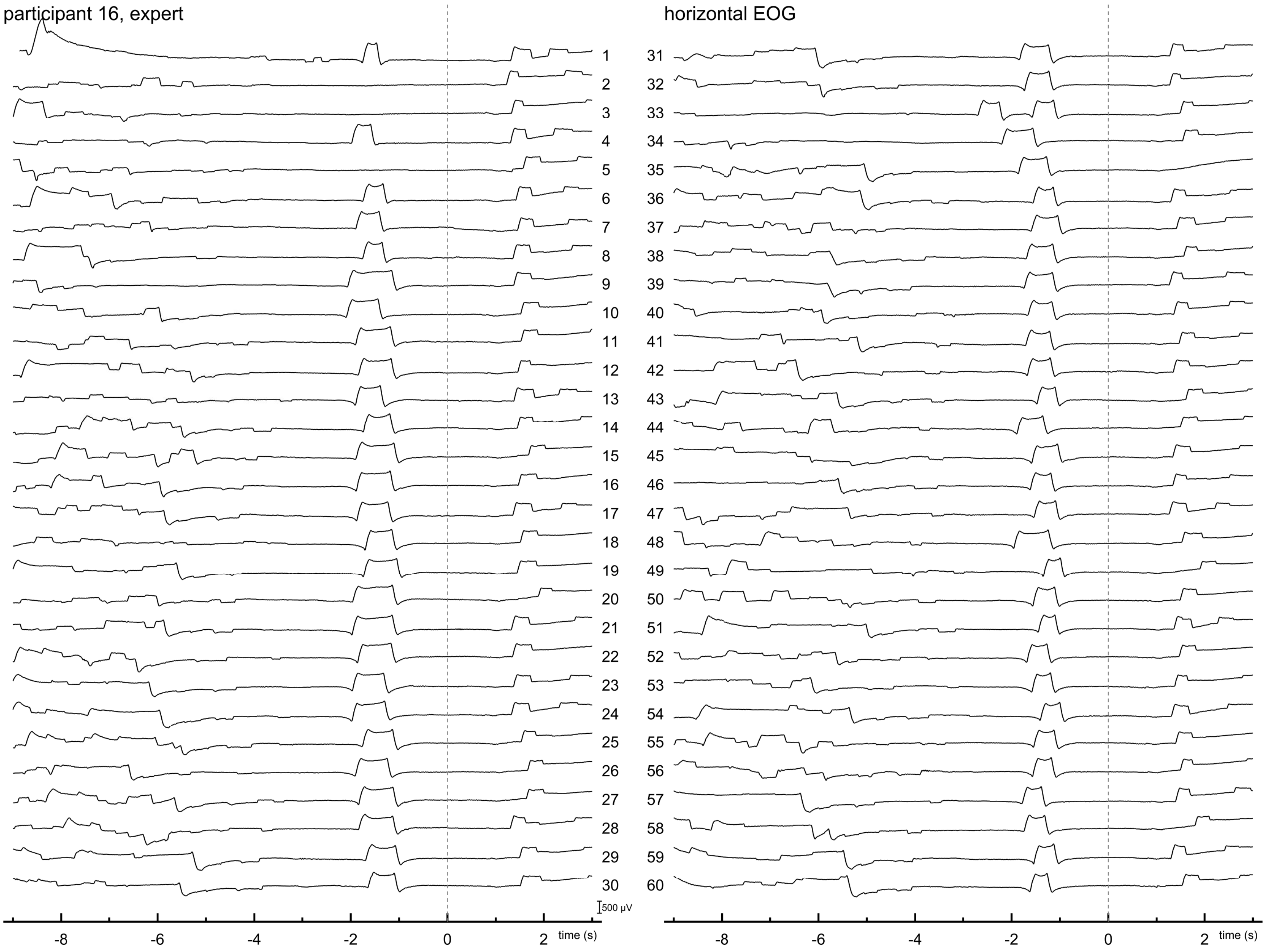
participant 16, expert
horizontal EOG
1
2
3
4
5
6
7
8
9
10
11
12
13
14
15
16
17
18
19
20
21
22
23
24
25
26
27
28
29
30
31
32
33
34
35
36
37
38
39
40
41
42
43
44
45
46
47
48
49
50
51
52
53
54
55
56
57
58
59
60
500 µV
-8
-6
-4
-2
0
2
time (s)
-8
-6
-4
-2
0
2
time (s)

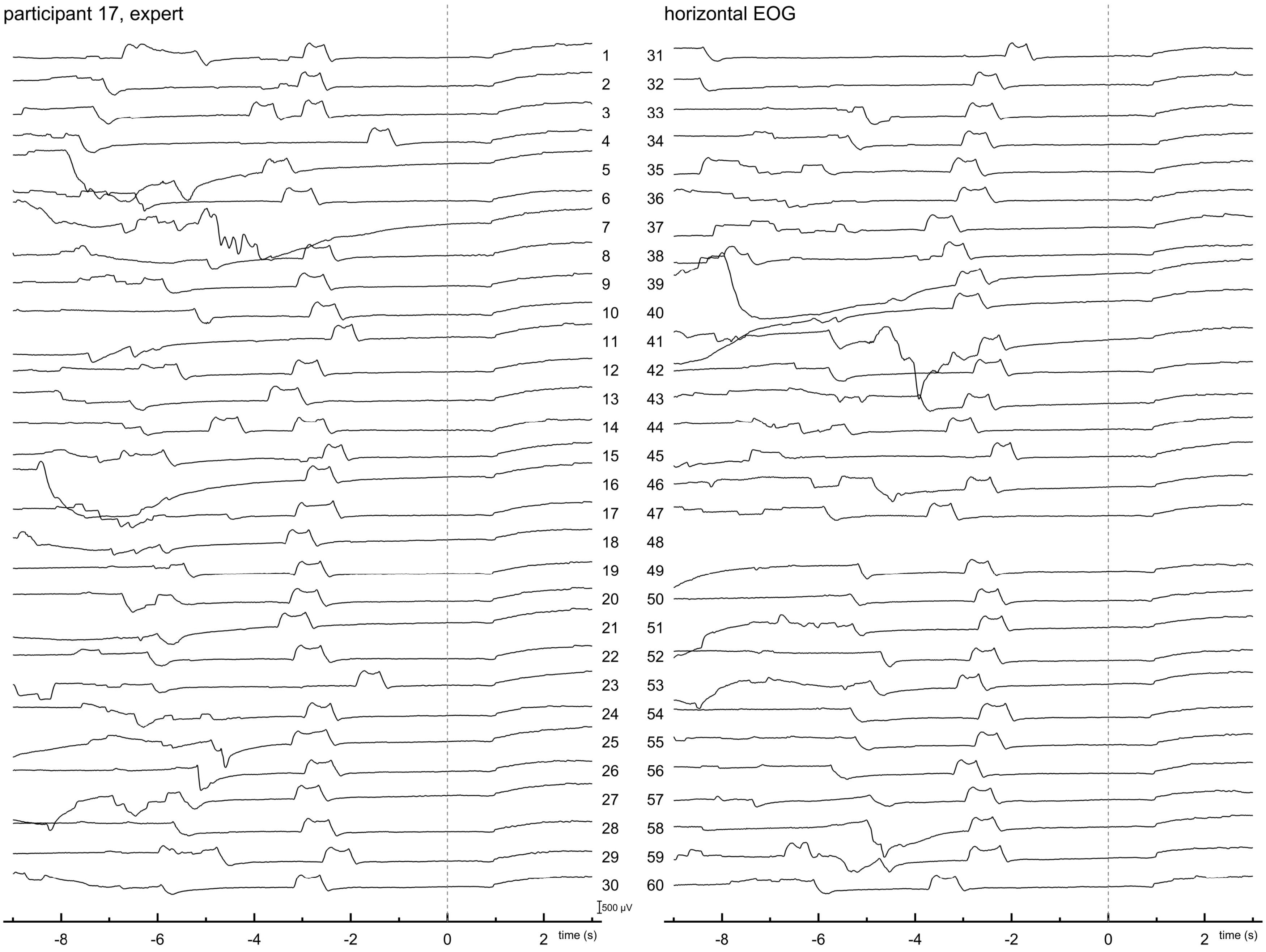
participant 17, expert
horizontal EOG
1
2
3
4
5
6
7
8
9
10
11
12
13
14
15
16
17
18
19
20
21
22
23
24
25
26
27
28
29
30
31
32
33
34
35
36
37
38
39
40
41
42
43
44
45
46
47
48
49
50
51
52
53
54
55
56
57
58
59
60
500 µV
-8
-6
-4
-2
0
2
time (s)

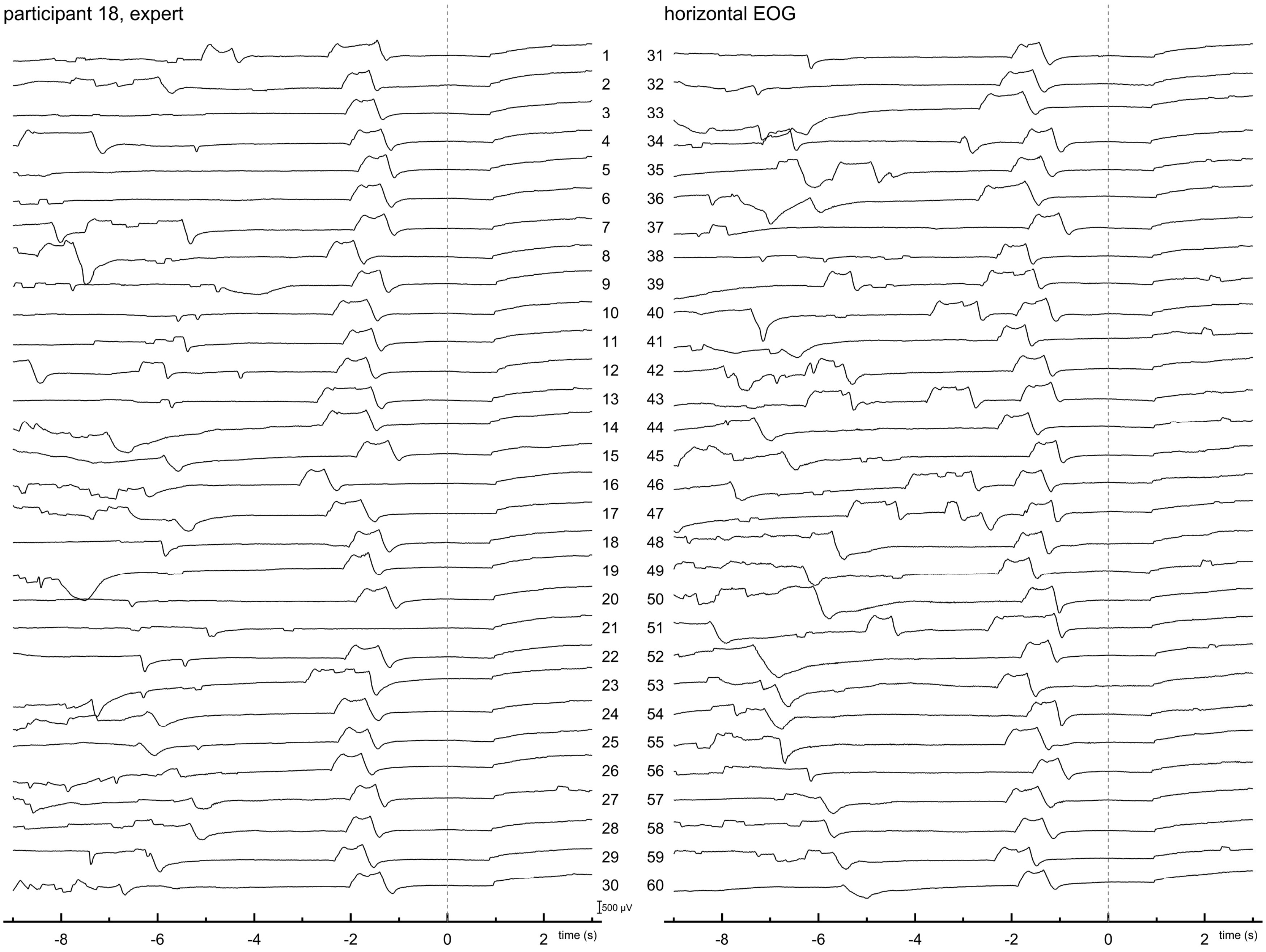
participant 18, expert
horizontal EOG
500 µV
time (s)
-8
-6
-4
-2
0
2

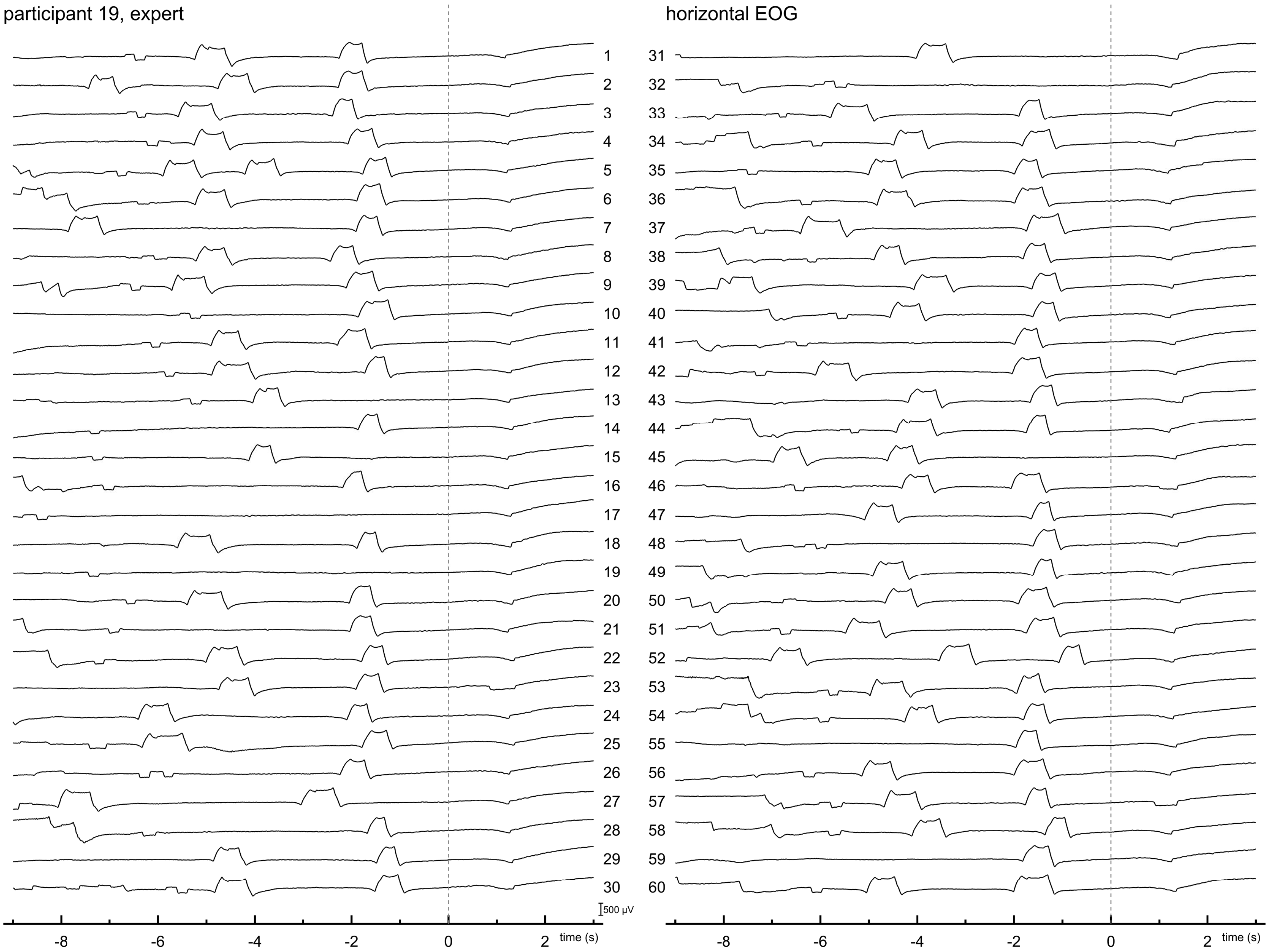
participant 19, expert
horizontal EOG
1
2
3
4
5
6
7
8
9
10
11
12
13
14
15
16
17
18
19
20
21
22
23
24
25
26
27
28
29
30
31
32
33
34
35
36
37
38
39
40
41
42
43
44
45
46
47
48
49
50
51
52
53
54
55
56
57
58
59
60
500 µV
-8
-6
-4
-2
0
2
time (s)
-8
-6
-4
-2
0
2
time (s)

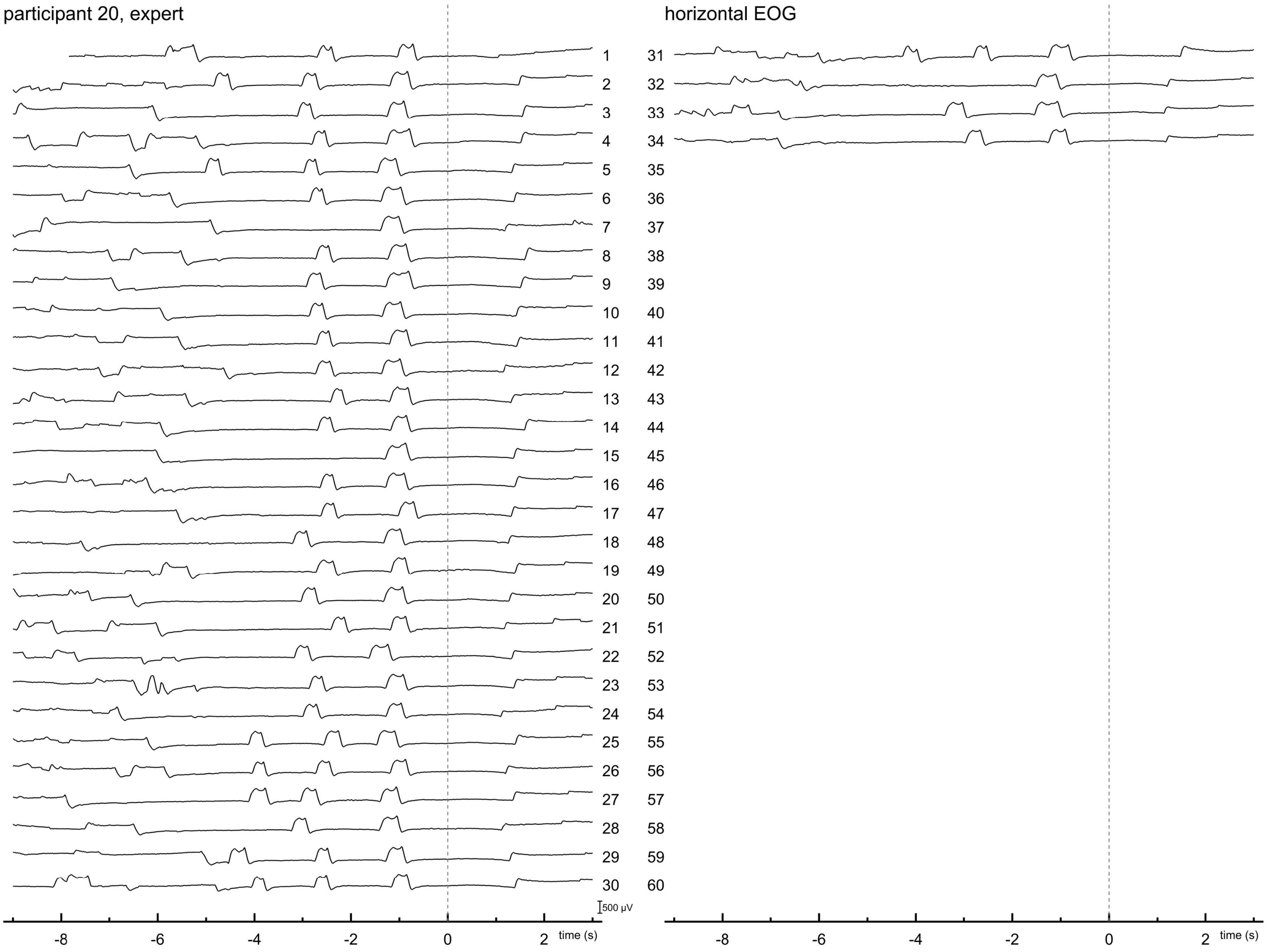

participant 20, expert
horizontal EOG
1
2
3
4
5
6
7
8
9
10
11
12
13
14
15
16
17
18
19
20
21
22
23
24
25
26
27
28
29
30
31
32
33
34
35
36
37
38
39
40
41
42
43
44
45
46
47
48
49
50
51
52
53
54
55
56
57
58
59
60
500 µV
-8
-6
-4
-2
0
2
time (s)

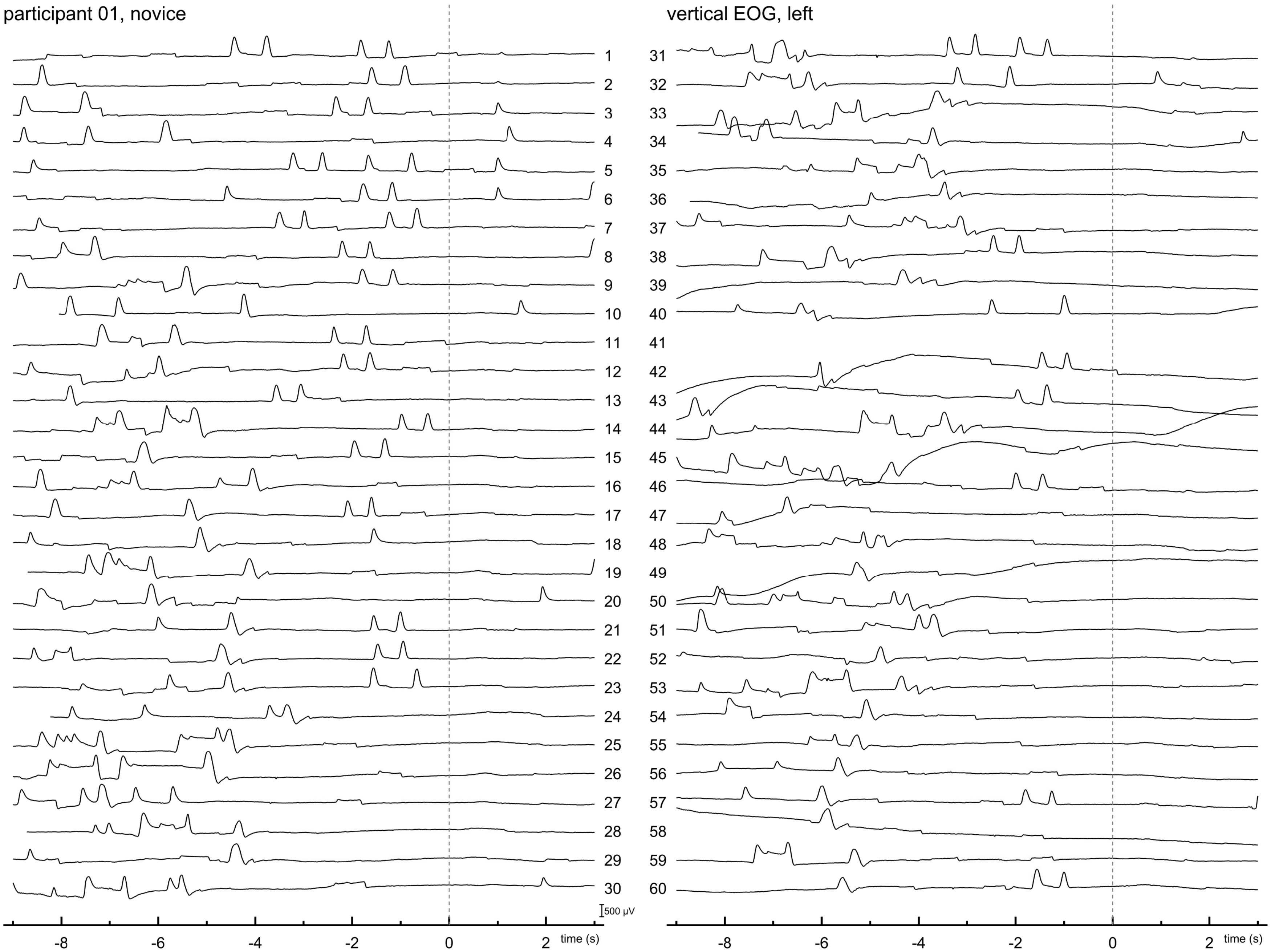

participant 01, novice
vertical EOG, left
1
2
3
4
5
6
7
8
9
10
11
12
13
14
15
16
17
18
19
20
21
22
23
24
25
26
27
28
29
30
31
32
33
34
35
36
37
38
39
40
41
42
43
44
45
46
47
48
49
50
51
52
53
54
55
56
57
58
59
60
500 µV
-8
-6
-4
-2
0
2
time (s)

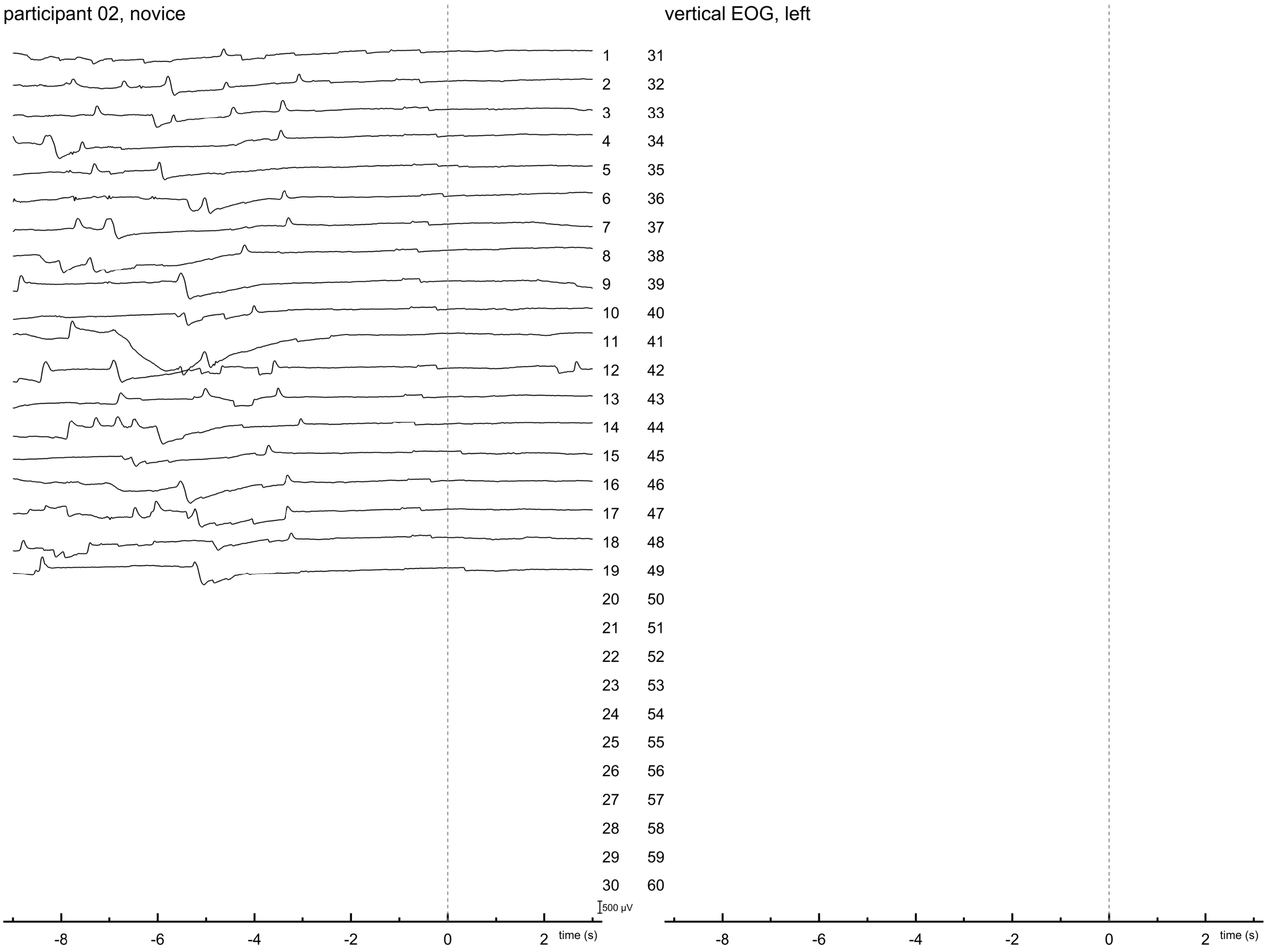
participant 02, novice
vertical EOG, left
1 2 3 4 5 6 7 8 9 10 11 12 13 14 15 16 17 18 19 20 21 22 23 24 25 26 27 28 29 30
31 32 33 34 35 36 37 38 39 40 41 42 43 44 45 46 47 48 49 50 51 52 53 54 55 56 57 58 59 60
500 µV
-8
-6
-4
-2
0
2
time (s)

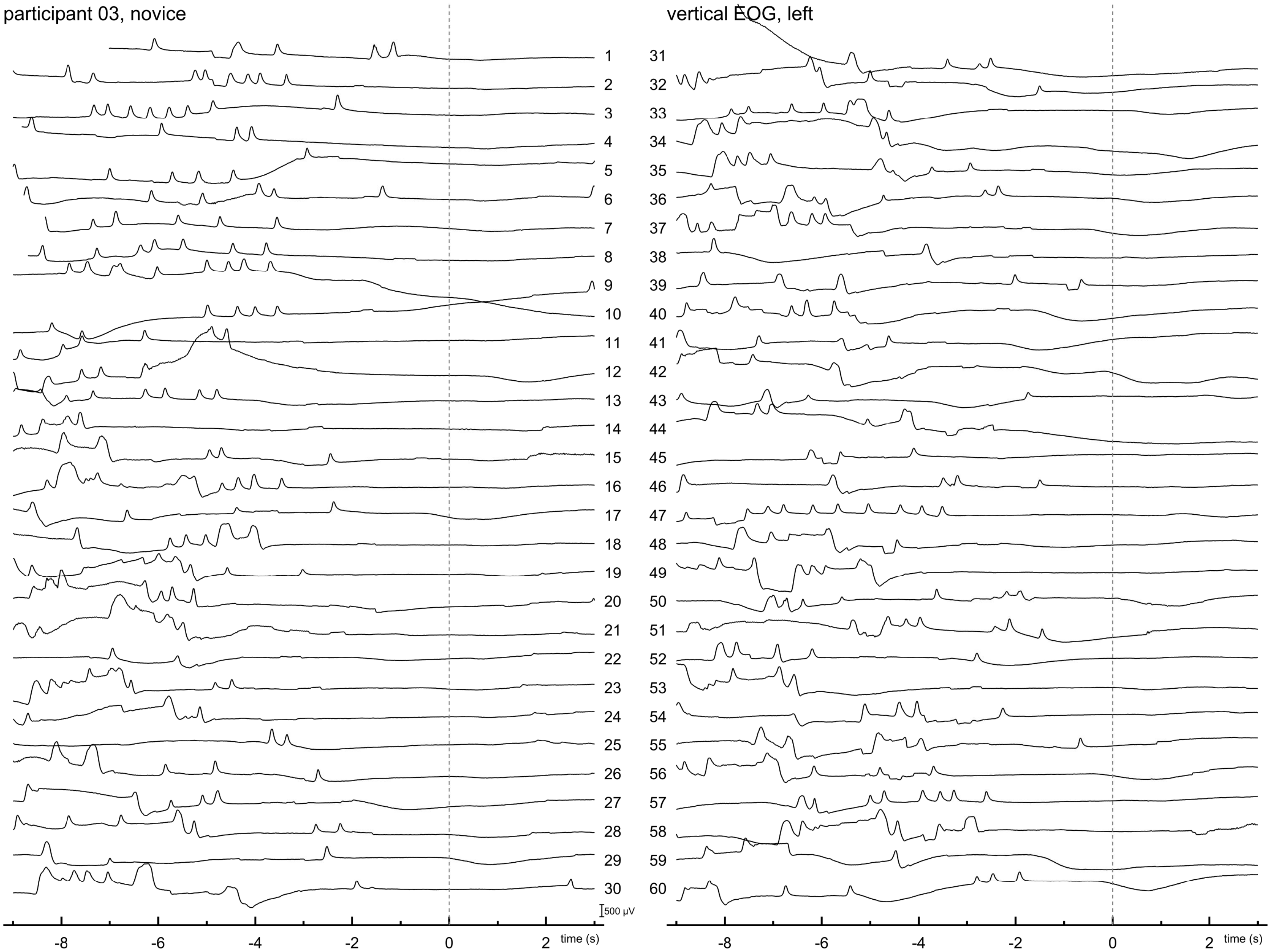

participant 03, novice
vertical EOG, left
1
2
3
4
5
6
7
8
9
10
11
12
13
14
15
16
17
18
19
20
21
22
23
24
25
26
27
28
29
30
31
32
33
34
35
36
37
38
39
40
41
42
43
44
45
46
47
48
49
50
51
52
53
54
55
56
57
58
59
60
500 µV
-8
-6
-4
-2
0
2
time (s)

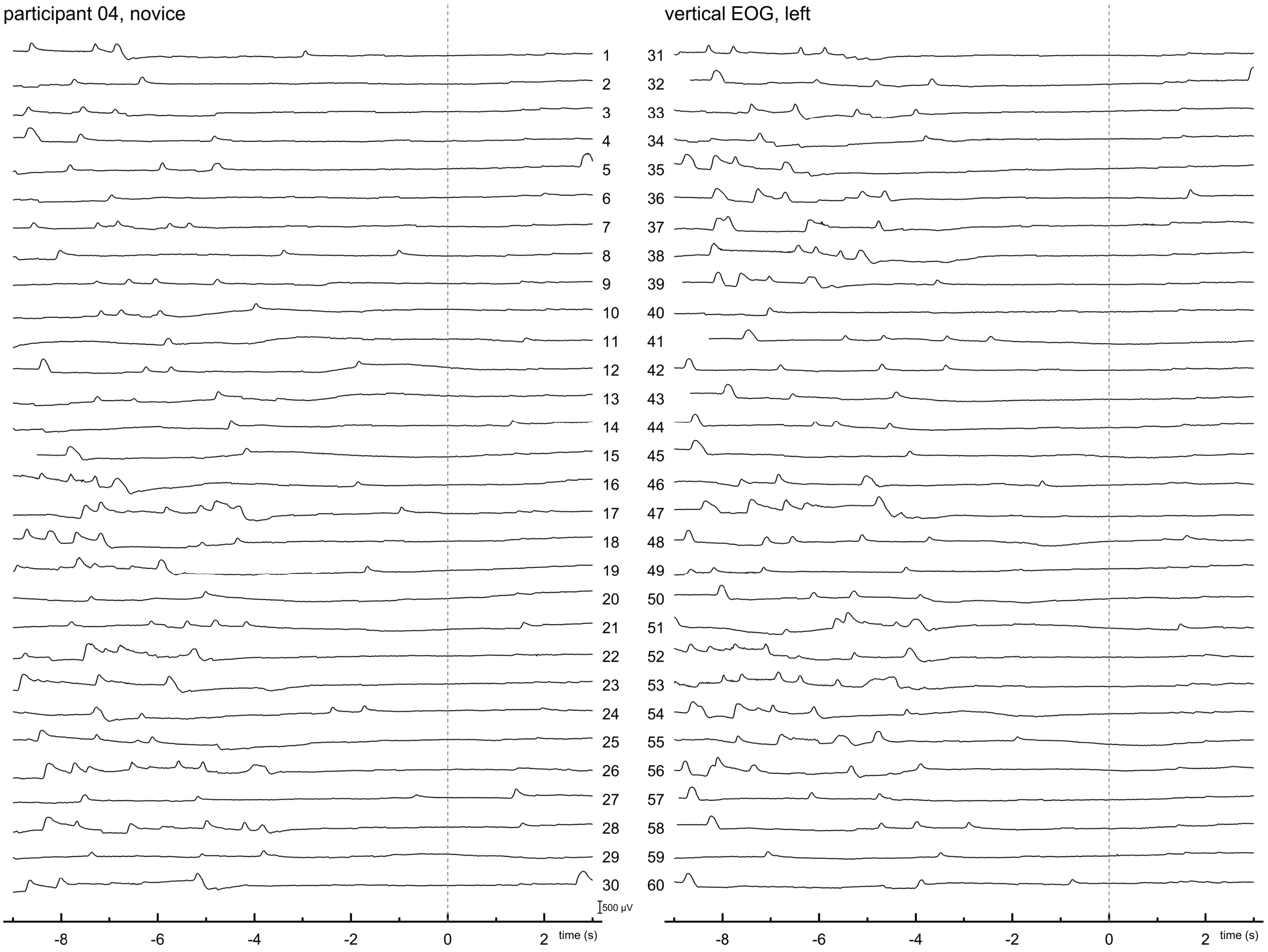
participant 04, novice
vertical EOG, left
1
2
3
4
5
6
7
8
9
10
11
12
13
14
15
16
17
18
19
20
21
22
23
24
25
26
27
28
29
30
31
32
33
34
35
36
37
38
39
40
41
42
43
44
45
46
47
48
49
50
51
52
53
54
55
56
57
58
59
60
500 µV
-8
-6
-4
-2
0
2
time (s)

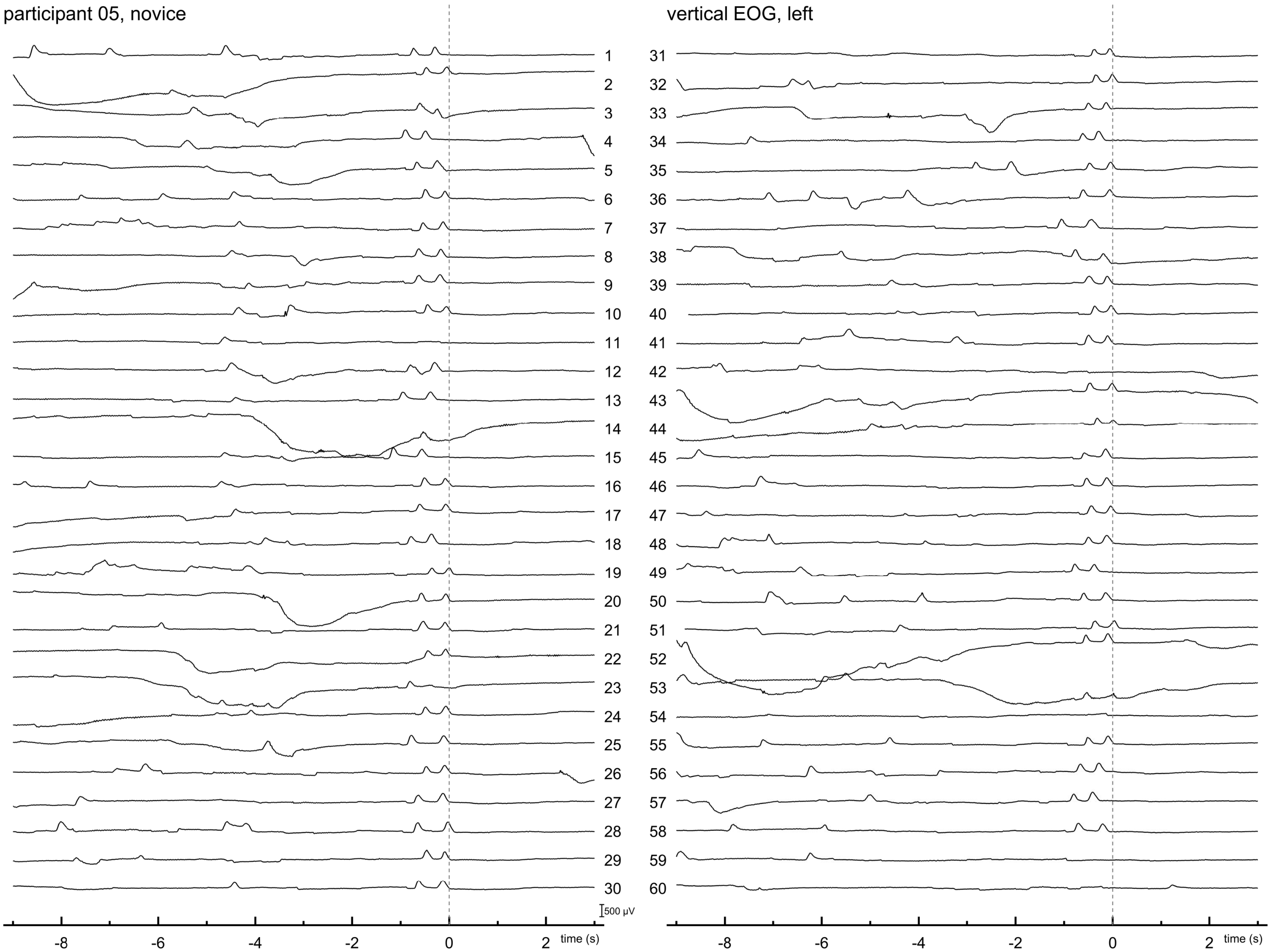

participant 05, novice
vertical EOG, left
500 µV
time (s)
-8
-6
-4
-2
0
2

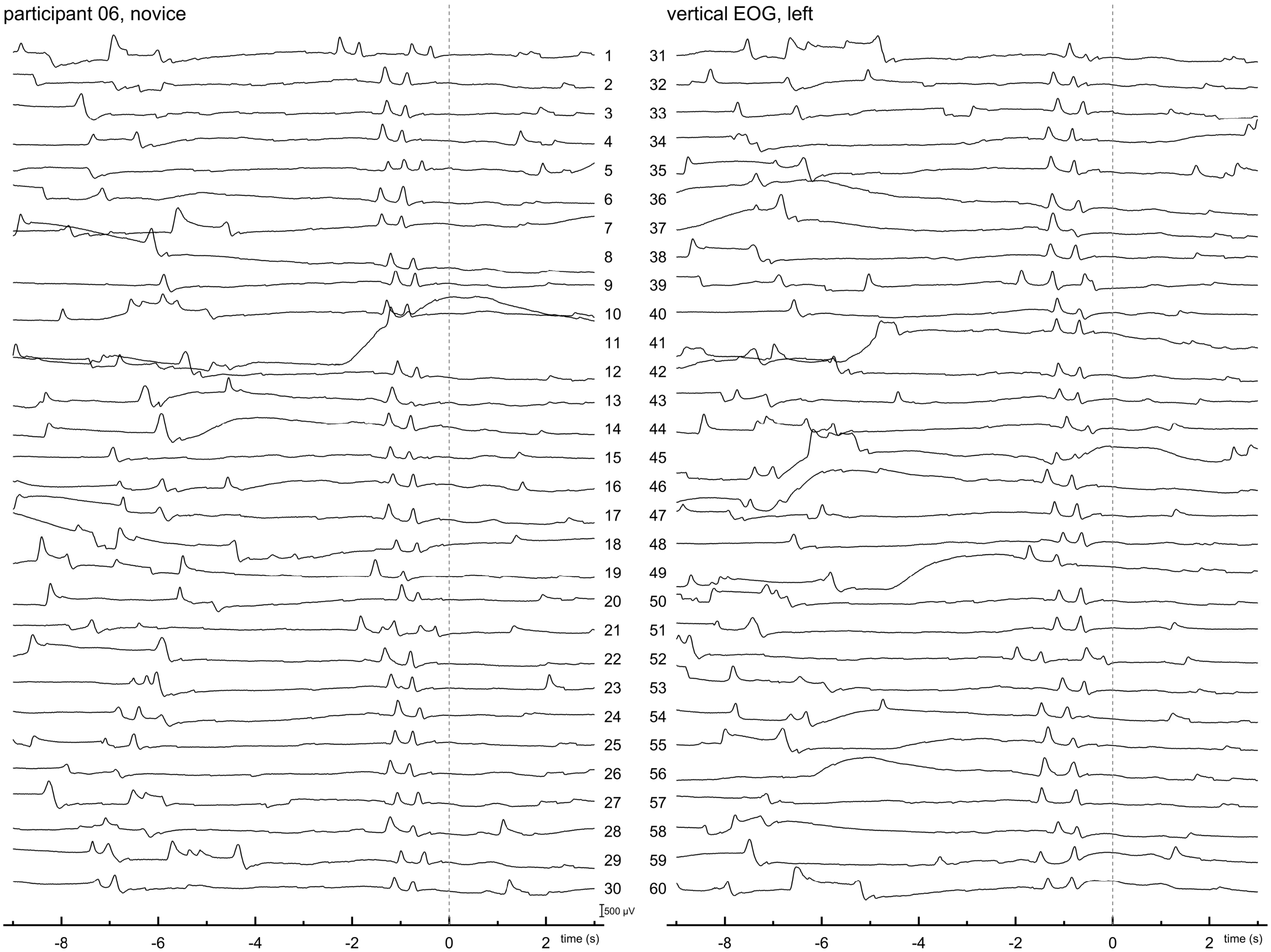

participant 06, novice
vertical EOG, left
1 2 3 4 5 6 7 8 9 10 11 12 13 14 15 16 17 18 19 20 21 22 23 24 25 26 27 28 29 30
31 32 33 34 35 36 37 38 39 40 41 42 43 44 45 46 47 48 49 50 51 52 53 54 55 56 57 58 59 60
500 µV
-8 -6 -4 -2 0 2
time (s)

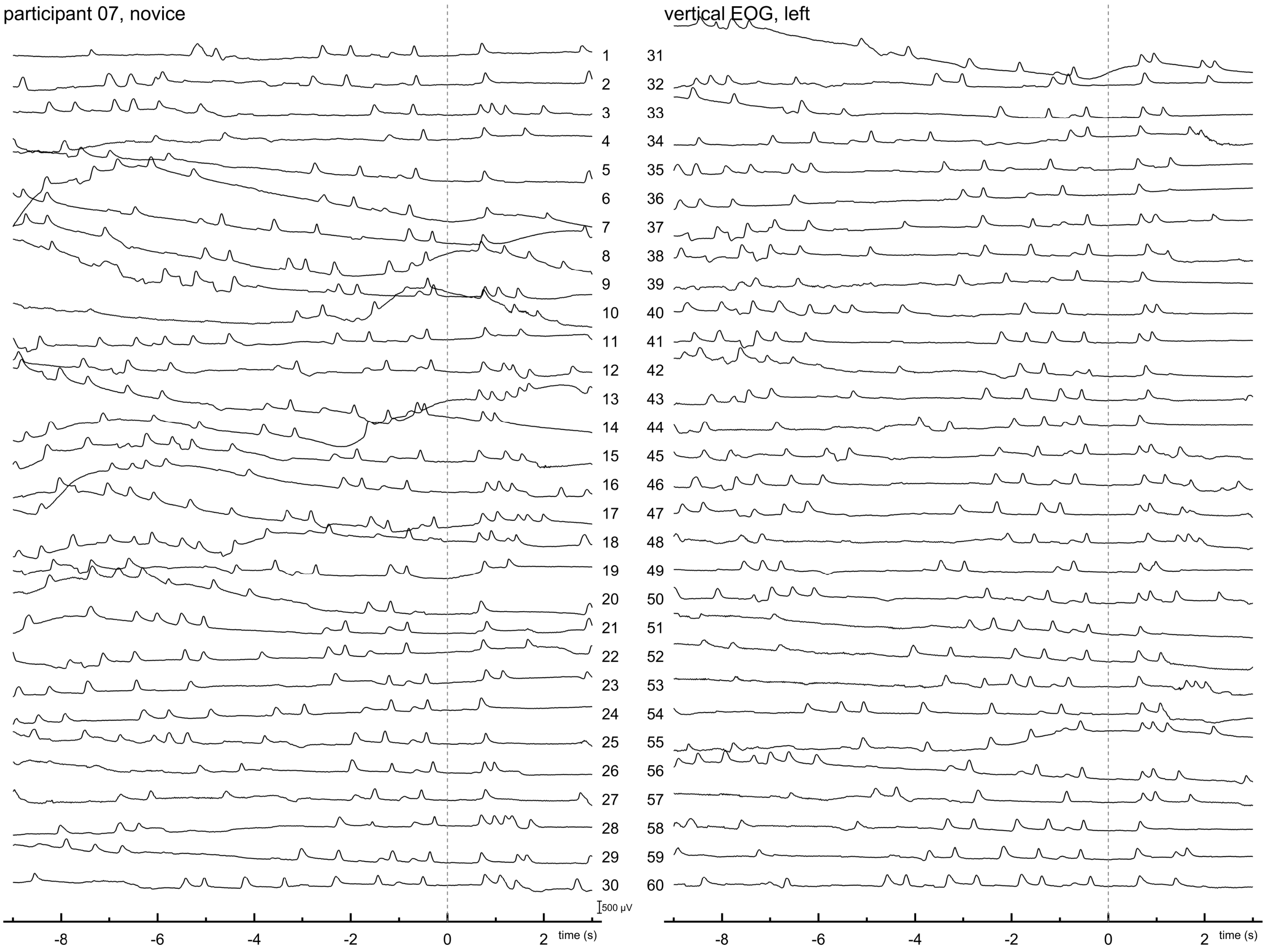
participant 07, novice
vertical EOG, left
1
2
3
4
5
6
7
8
9
10
11
12
13
14
15
16
17
18
19
20
21
22
23
24
25
26
27
28
29
30
31
32
33
34
35
36
37
38
39
40
41
42
43
44
45
46
47
48
49
50
51
52
53
54
55
56
57
58
59
60
500 µV
-8
-6
-4
-2
0
2
time (s)

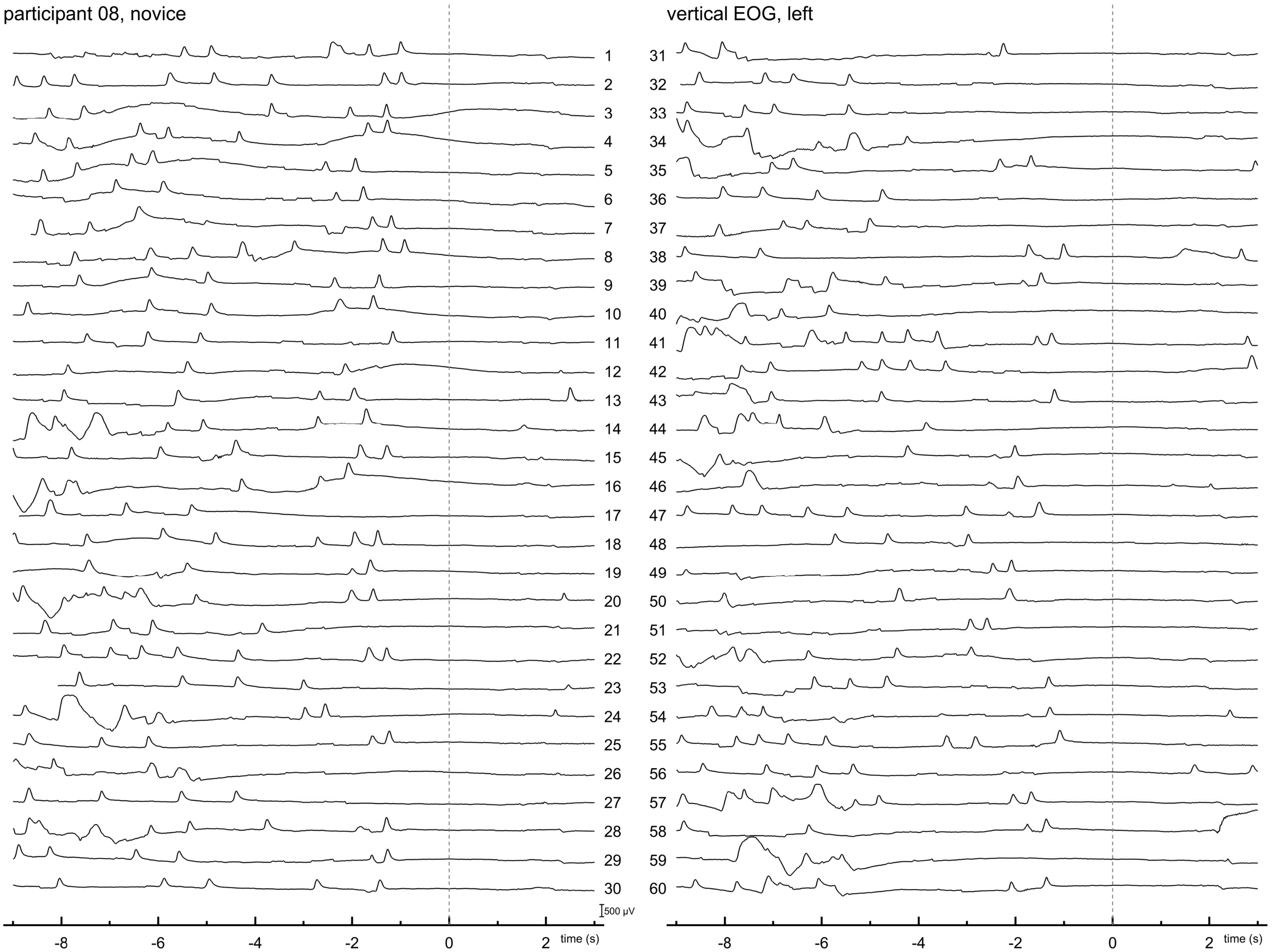
participant 08, novice
vertical EOG, left
500 µV
time (s)
-8
-6
-4
-2
0
2

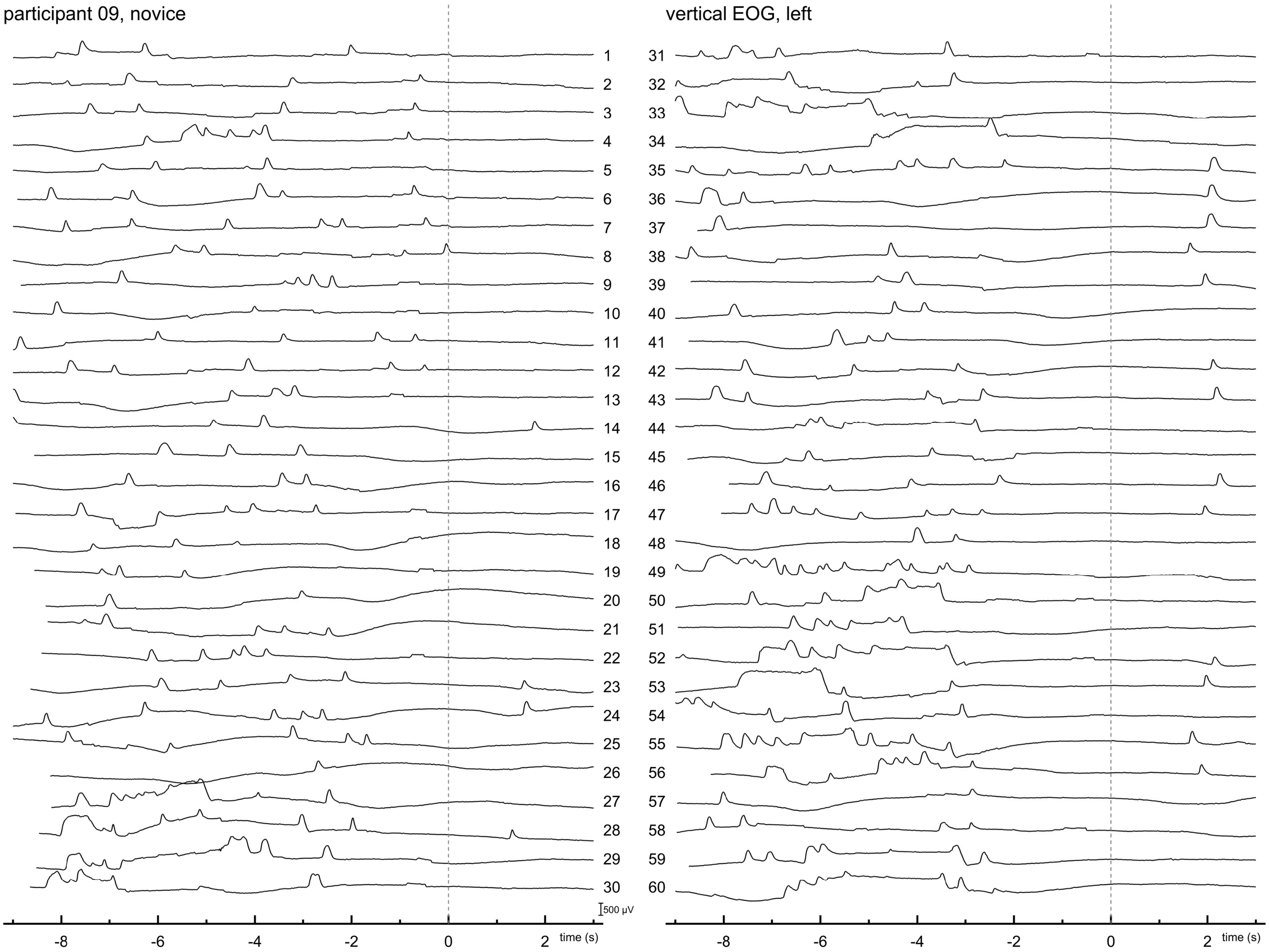
participant 09, novice
vertical EOG, left
1 2 3 4 5 6 7 8 9 10 11 12 13 14 15 16 17 18 19 20 21 22 23 24 25 26 27 28 29 30
31 32 33 34 35 36 37 38 39 40 41 42 43 44 45 46 47 48 49 50 51 52 53 54 55 56 57 58 59 60
500 µV
-8
-6
-4
-2
0
2
time (s)

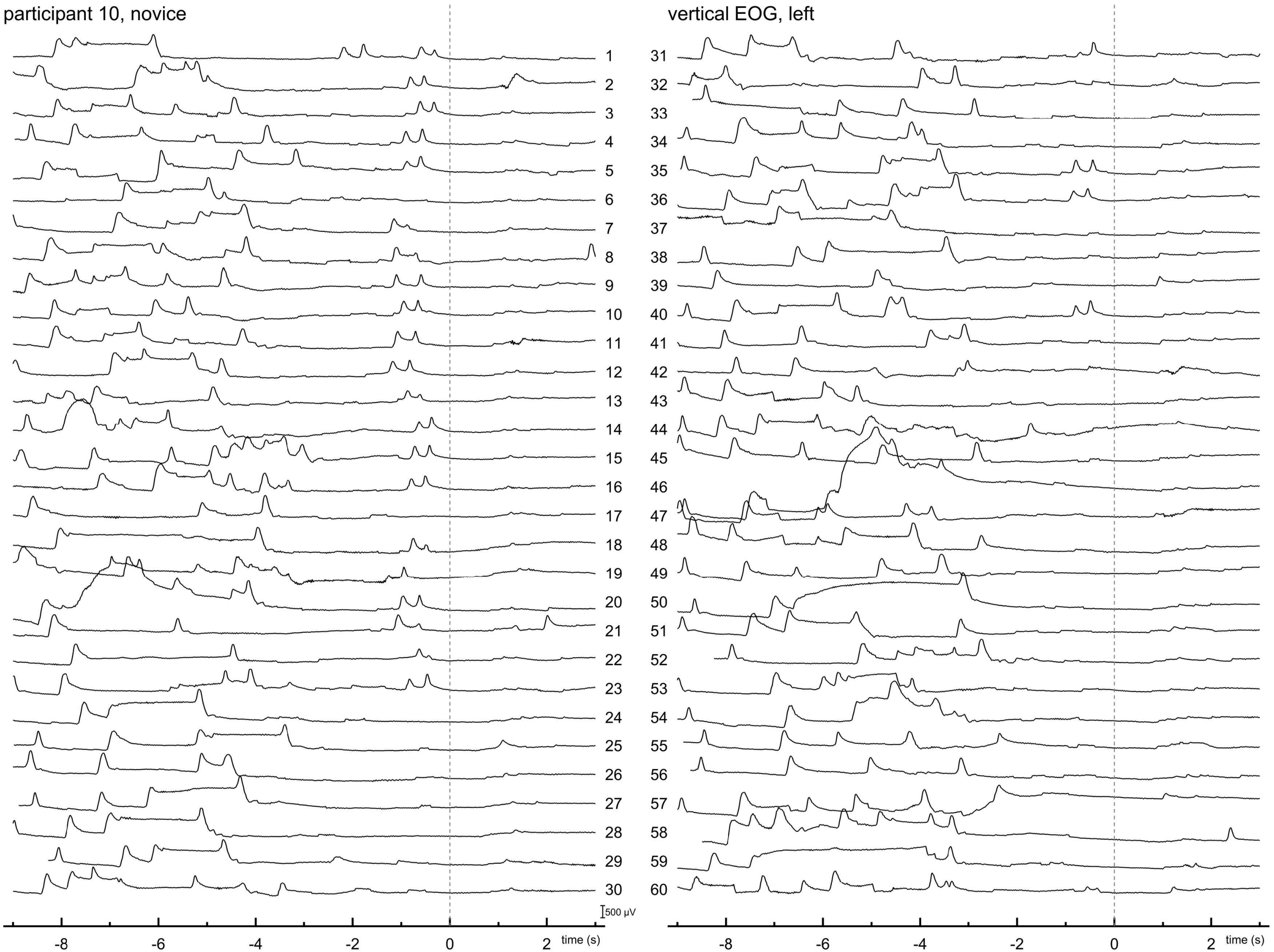
participant 10, novice
vertical EOG, left
500 µV
time (s)
-8
-6
-4
-2
0
2

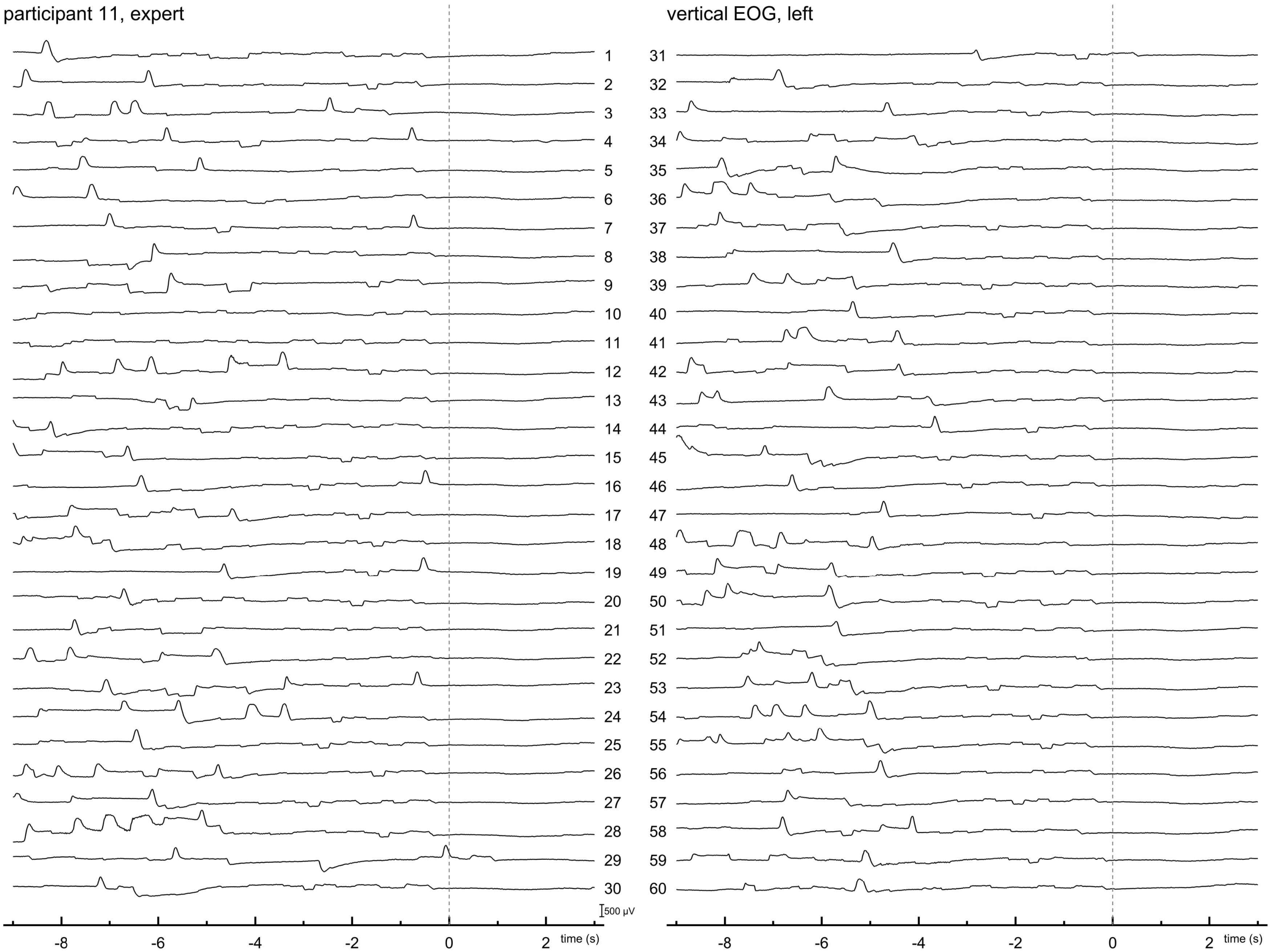

participant 11, expert
vertical EOG, left
1 2 3 4 5 6 7 8 9 10 11 12 13 14 15 16 17 18 19 20 21 22 23 24 25 26 27 28 29 30
31 32 33 34 35 36 37 38 39 40 41 42 43 44 45 46 47 48 49 50 51 52 53 54 55 56 57 58 59 60
500 µV
-8 -6 -4 -2 0 2 time (s)
-8 -6 -4 -2 0 2 time (s)

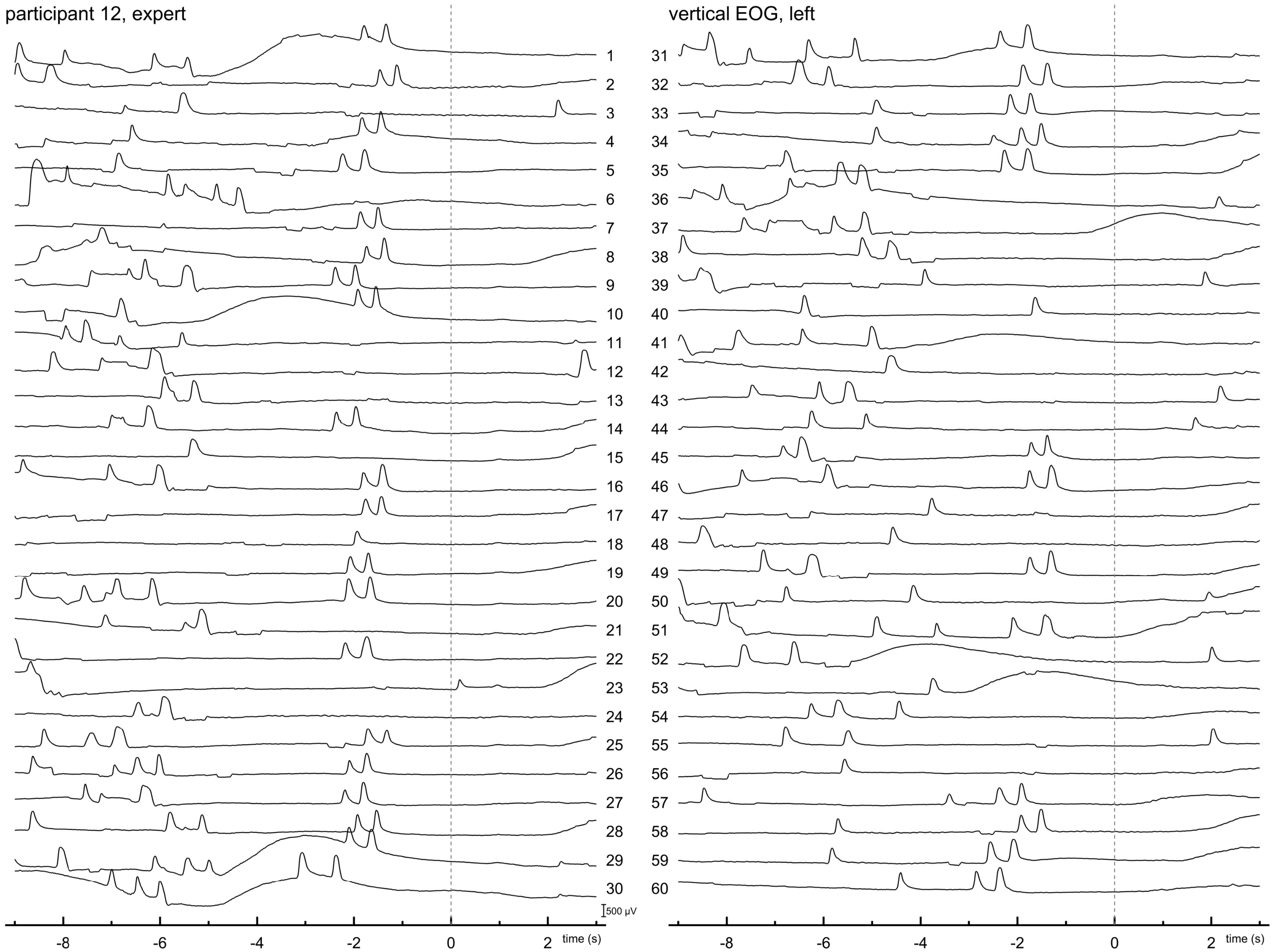

participant 12, expert
vertical EOG, left
500 µV
time (s)
time (s)

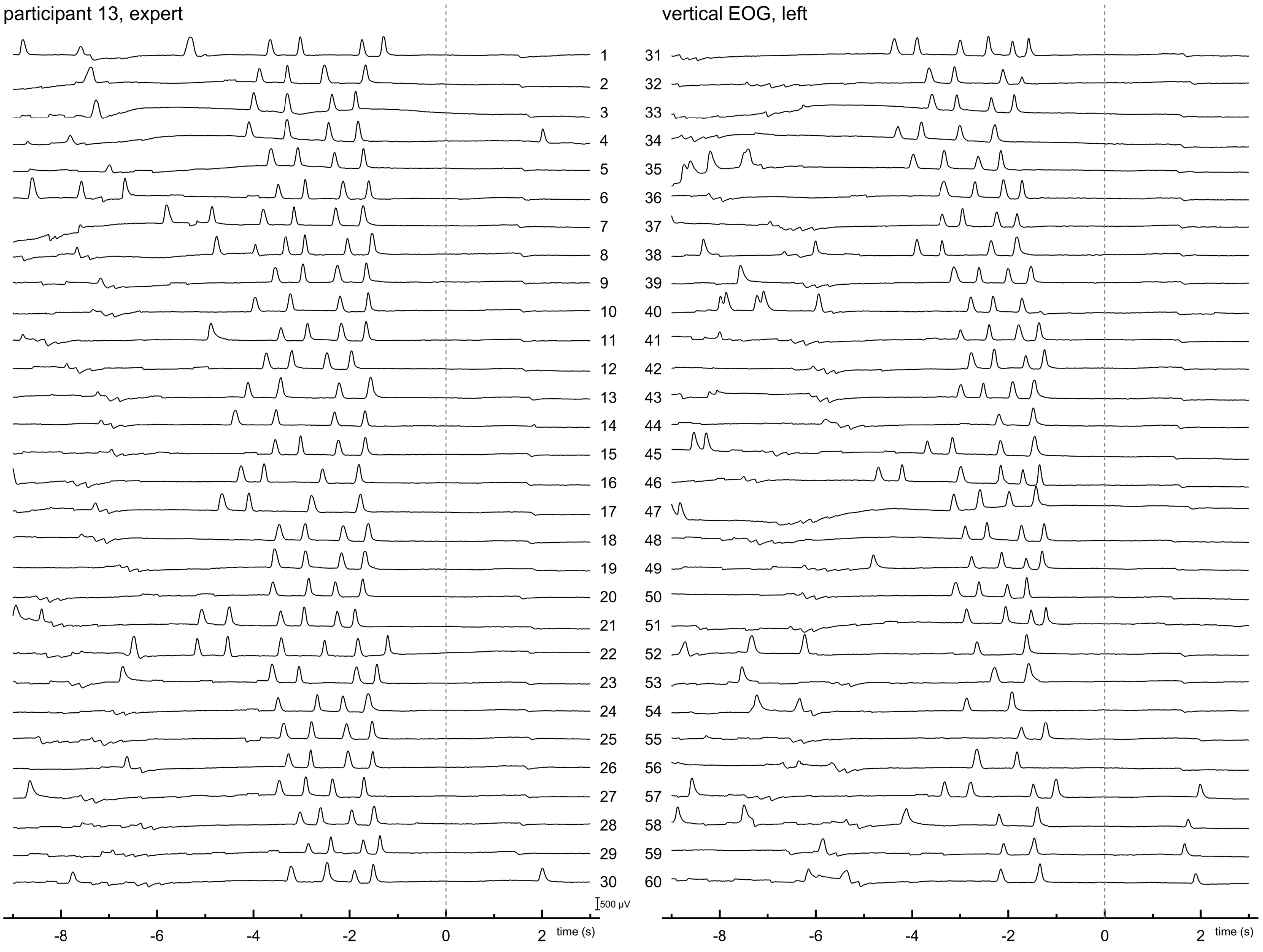
participant 13, expert
vertical EOG, left
500 µV
time (s)
-8
-6
-4
-2
0
2

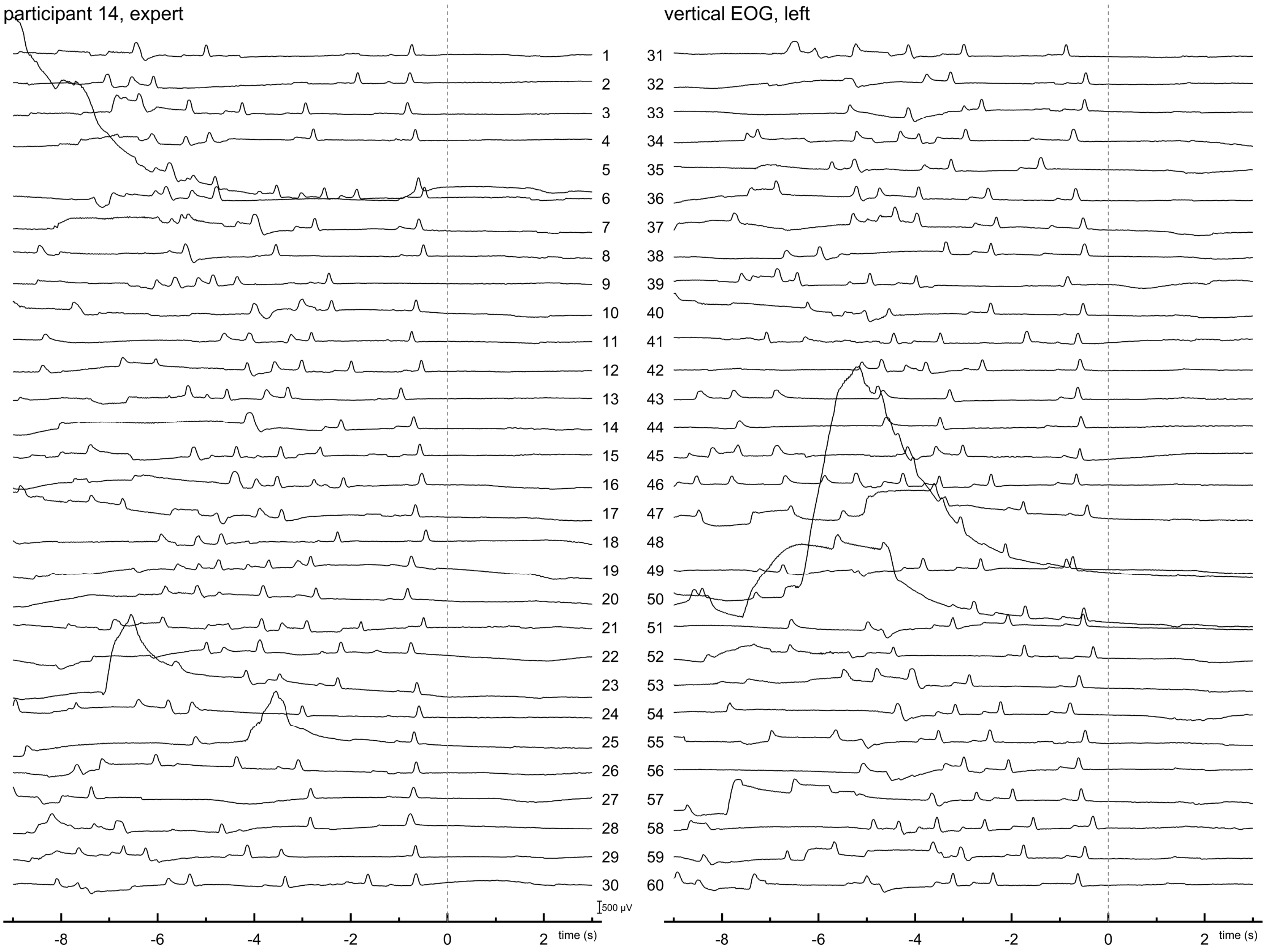
participant 14, expert
vertical EOG, left
1
2
3
4
5
6
7
8
9
10
11
12
13
14
15
16
17
18
19
20
21
22
23
24
25
26
27
28
29
30
31
32
33
34
35
36
37
38
39
40
41
42
43
44
45
46
47
48
49
50
51
52
53
54
55
56
57
58
59
60
500 µV
-8
-6
-4
-2
0
2
time (s)

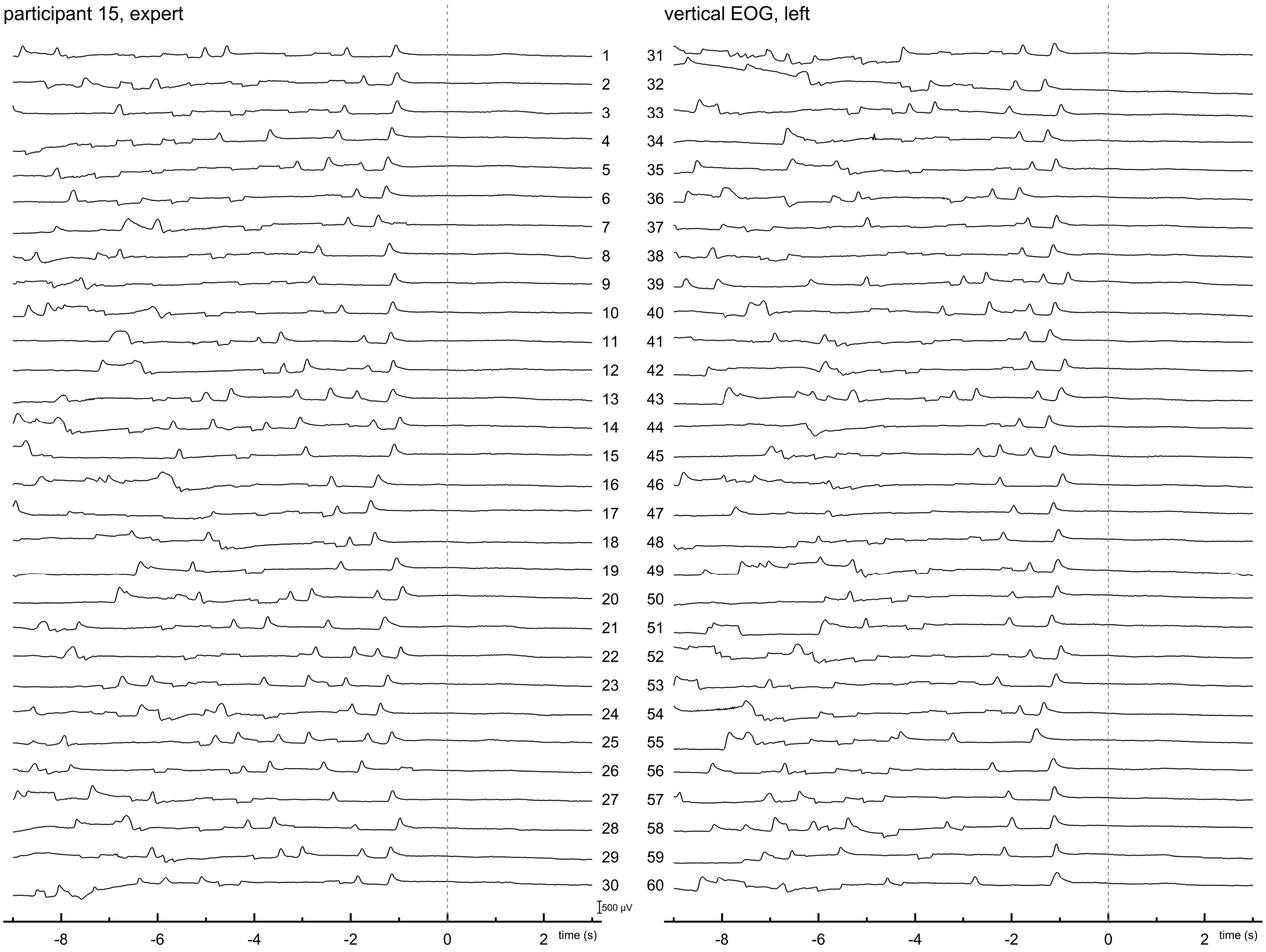
participant 15, expert
vertical EOG, left
1 2 3 4 5 6 7 8 9 10 11 12 13 14 15 16 17 18 19 20 21 22 23 24 25 26 27 28 29 30
31 32 33 34 35 36 37 38 39 40 41 42 43 44 45 46 47 48 49 50 51 52 53 54 55 56 57 58 59 60
500 µV
-8 -6 -4 -2 0 2
time (s)
-8 -6 -4 -2 0 2
time (s)

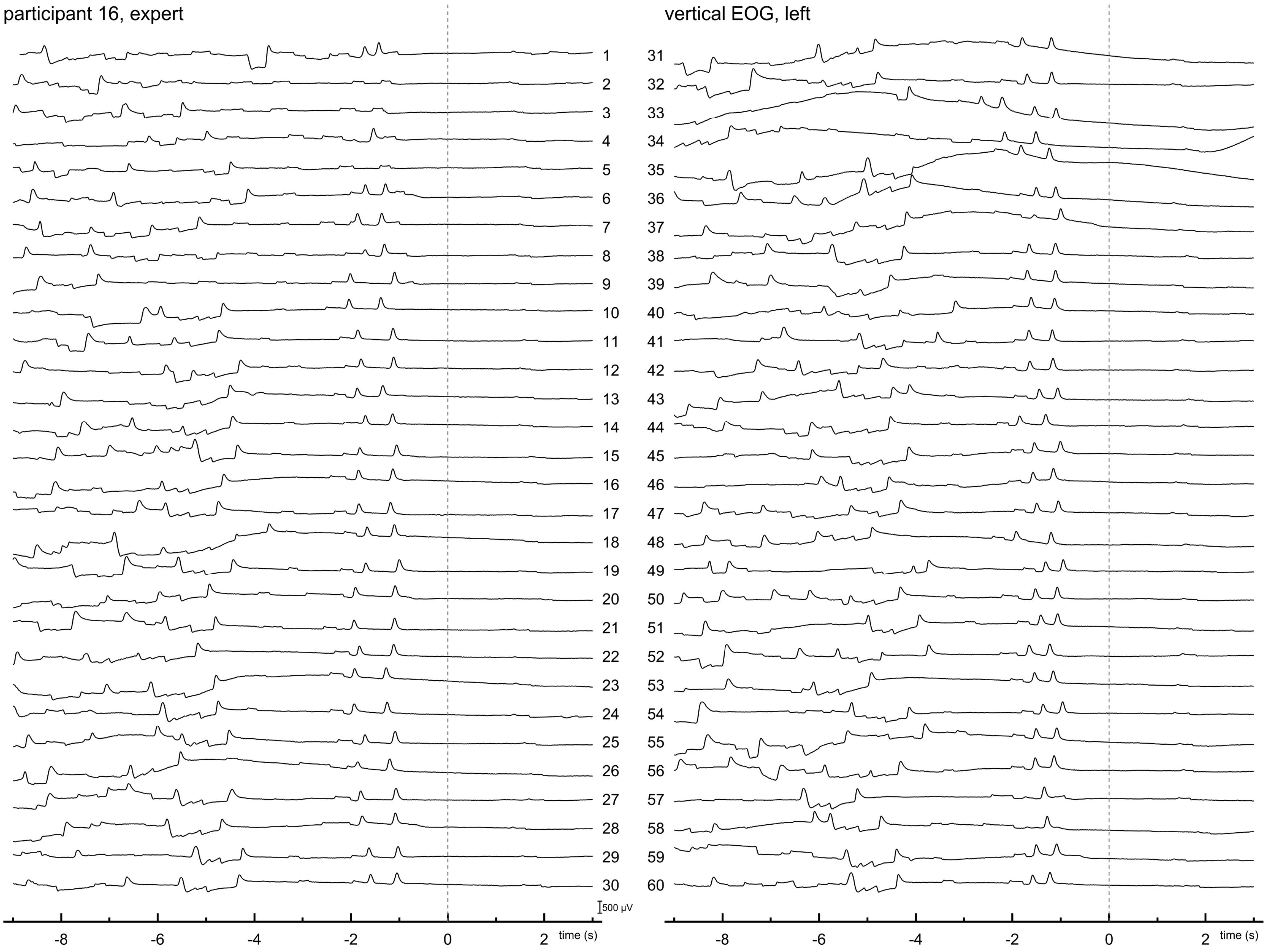
participant 16, expert
vertical EOG, left
1
2
3
4
5
6
7
8
9
10
11
12
13
14
15
16
17
18
19
20
21
22
23
24
25
26
27
28
29
30
31
32
33
34
35
36
37
38
39
40
41
42
43
44
45
46
47
48
49
50
51
52
53
54
55
56
57
58
59
60
500 µV
-8
-6
-4
-2
0
2
time (s)

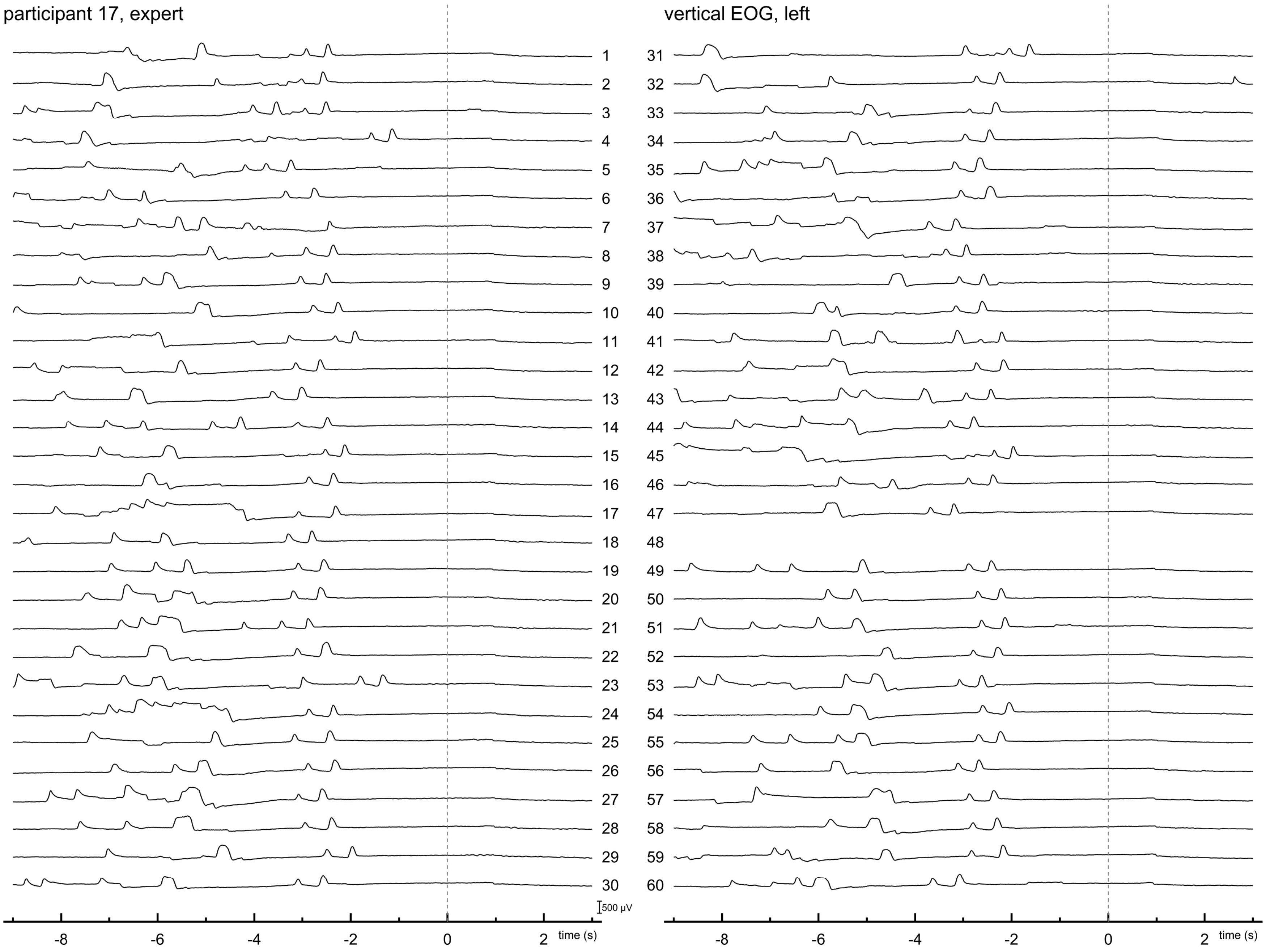
participant 17, expert
vertical EOG, left
1
2
3
4
5
6
7
8
9
10
11
12
13
14
15
16
17
18
19
20
21
22
23
24
25
26
27
28
29
30
31
32
33
34
35
36
37
38
39
40
41
42
43
44
45
46
47
48
49
50
51
52
53
54
55
56
57
58
59
60
500 µV
-8
-6
-4
-2
0
2
time (s)

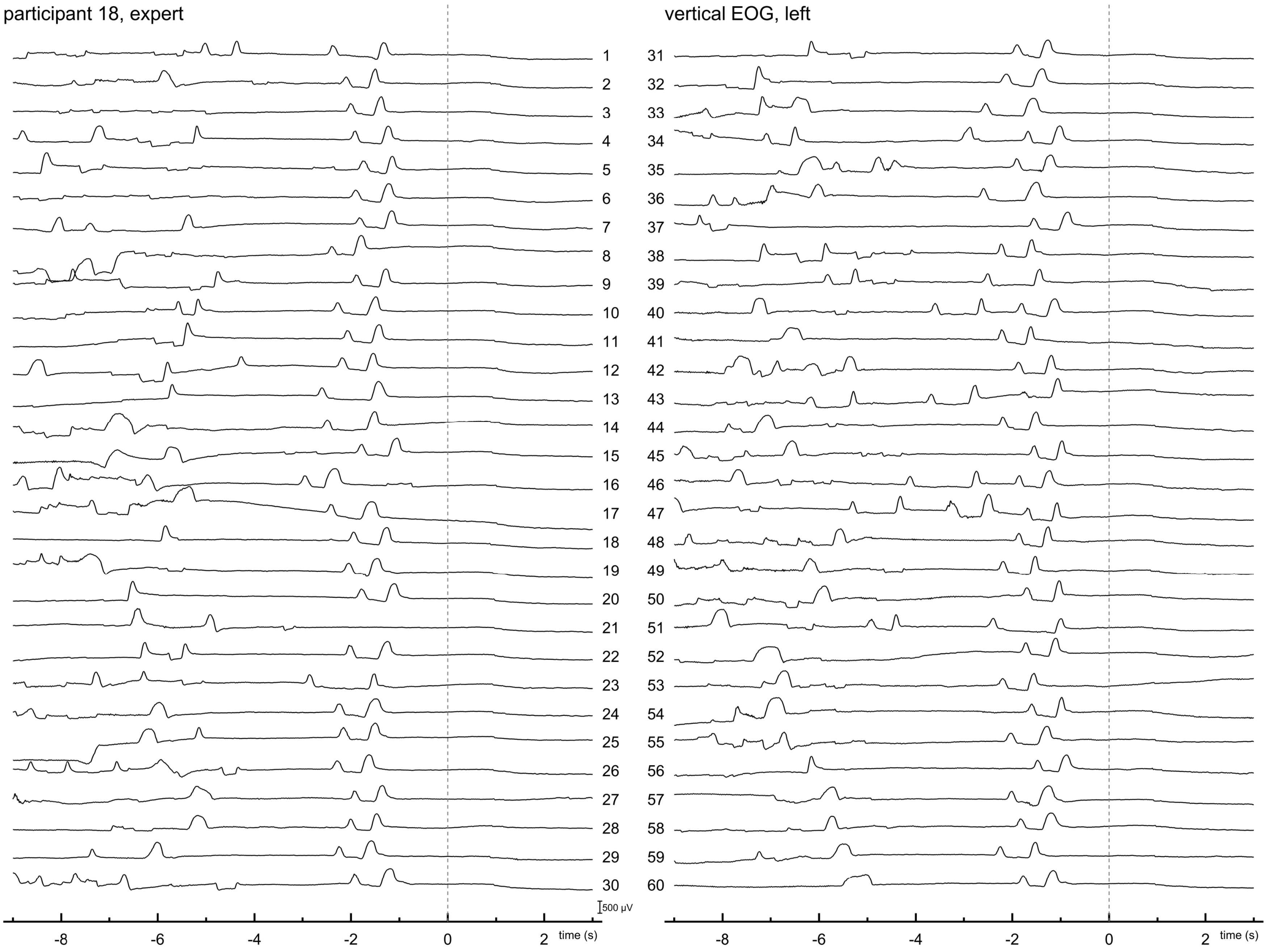
participant 18, expert
vertical EOG, left
1
2
3
4
5
6
7
8
9
10
11
12
13
14
15
16
17
18
19
20
21
22
23
24
25
26
27
28
29
30
31
32
33
34
35
36
37
38
39
40
41
42
43
44
45
46
47
48
49
50
51
52
53
54
55
56
57
58
59
60
500 µV
-8
-6
-4
-2
0
2
time (s)

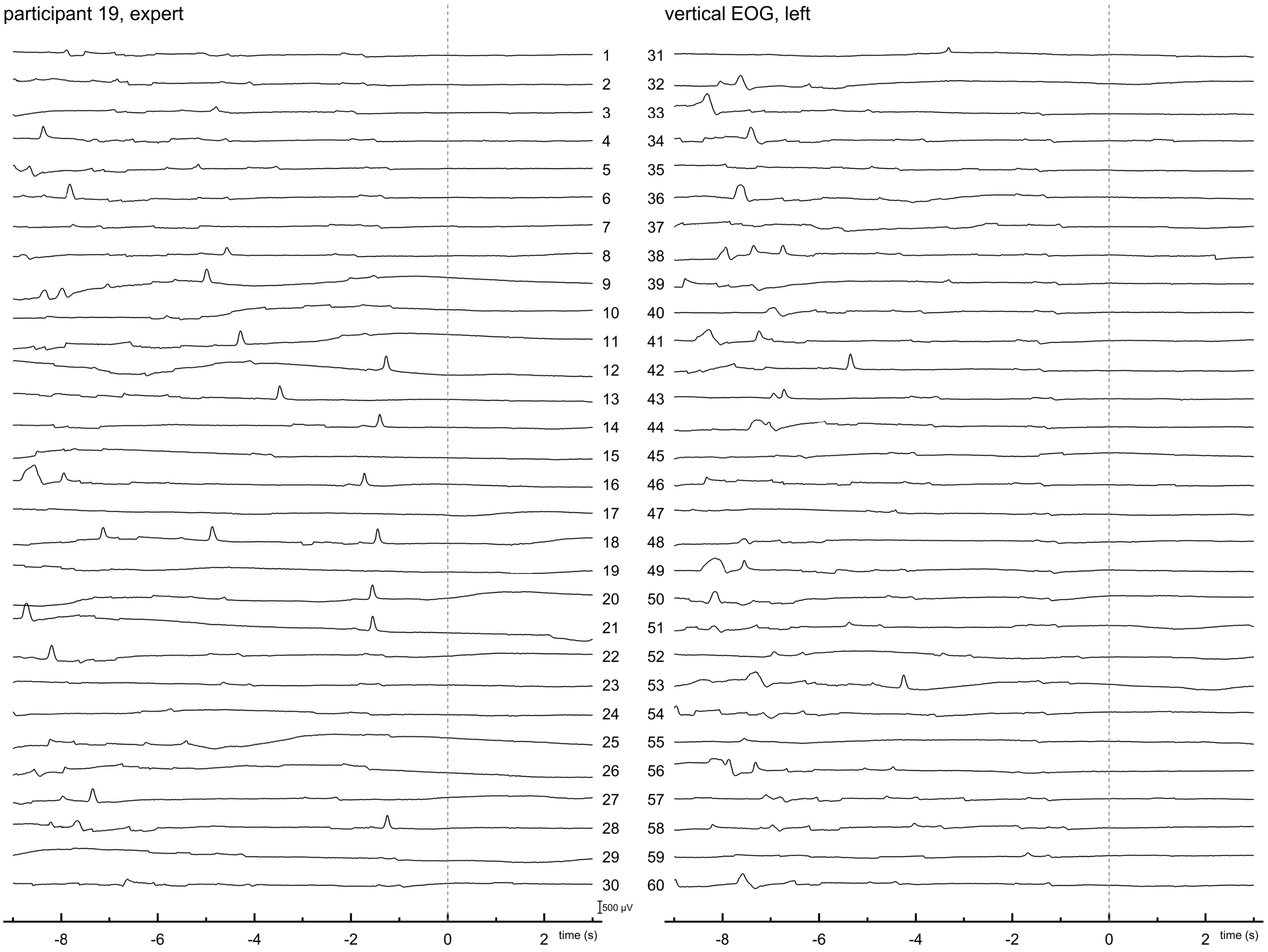
participant 19, expert
vertical EOG, left
1
2
3
4
5
6
7
8
9
10
11
12
13
14
15
16
17
18
19
20
21
22
23
24
25
26
27
28
29
30
31
32
33
34
35
36
37
38
39
40
41
42
43
44
45
46
47
48
49
50
51
52
53
54
55
56
57
58
59
60
500 µV
-8
-6
-4
-2
0
2
time (s)

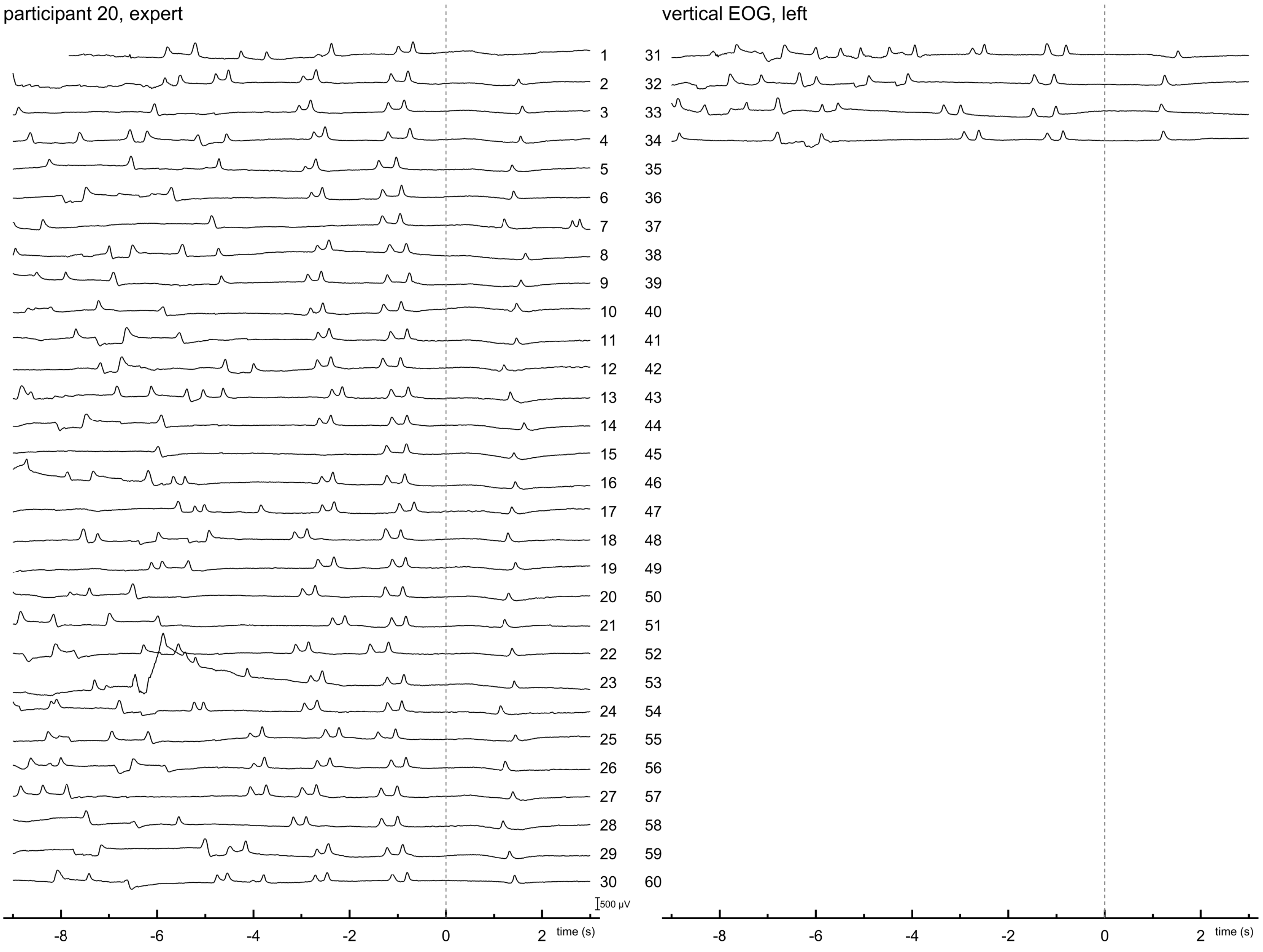
participant 20, expert
vertical EOG, left
1 2 3 4 5 6 7 8 9 10 11 12 13 14 15 16 17 18 19 20 21 22 23 24 25 26 27 28 29 30
31 32 33 34 35 36 37 38 39 40 41 42 43 44 45 46 47 48 49 50 51 52 53 54 55 56 57 58 59 60
500 µV
-8 -6 -4 -2 0 2
time (s)
-8 -6 -4 -2 0 2
time (s)

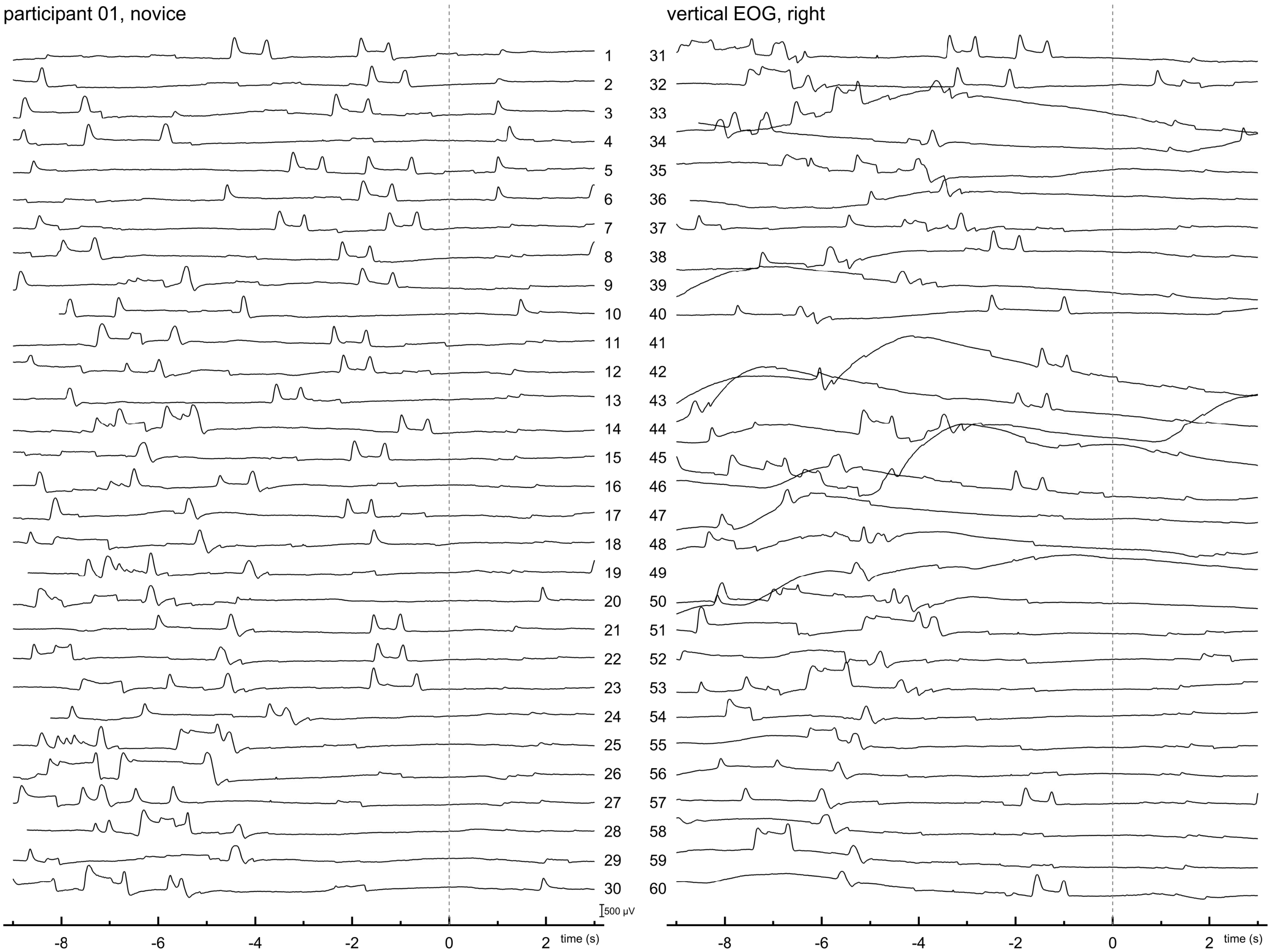

participant 01, novice
vertical EOG, right
500 µV
time (s)
-8
-6
-4
-2
0
2

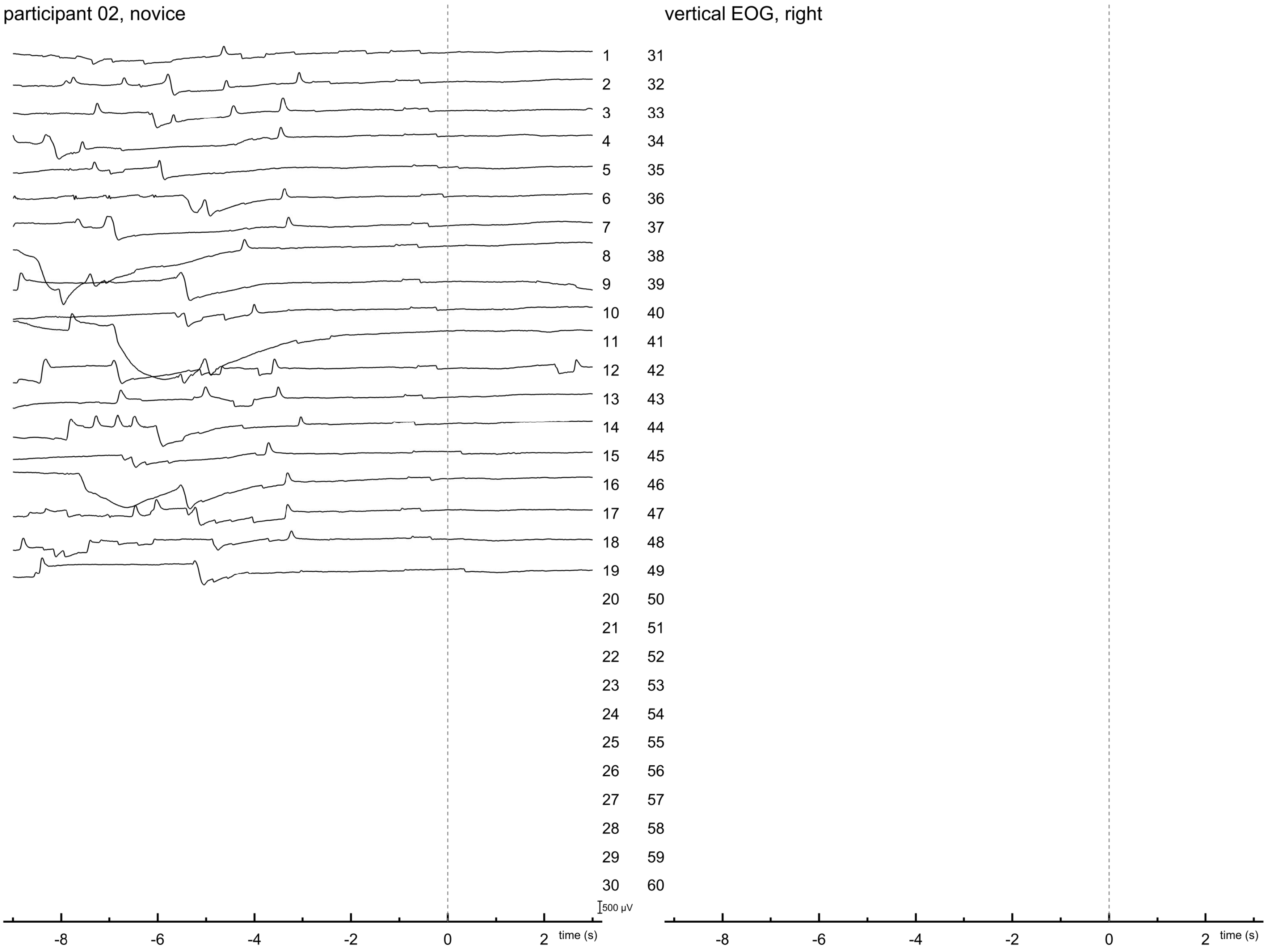
participant 02, novice
vertical EOG, right
1 31
2 32
3 33
4 34
5 35
6 36
7 37
8 38
9 39
10 40
11 41
12 42
13 43
14 44
15 45
16 46
17 47
18 48
19 49
20 50
21 51
22 52
23 53
24 54
25 55
26 56
27 57
28 58
29 59
30 60
500 µV
-8
-6
-4
-2
0
2
time (s)

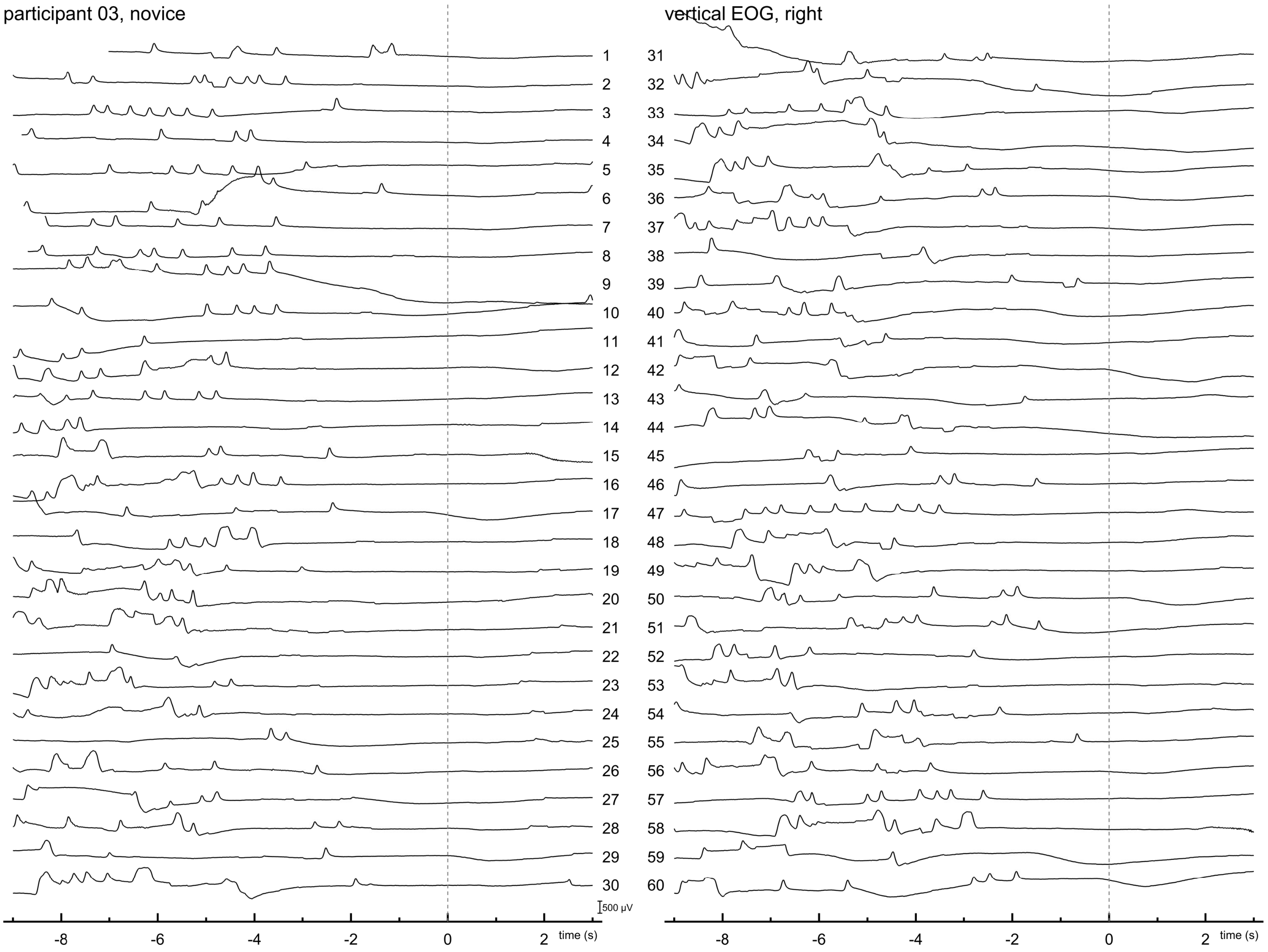

participant 03, novice
vertical EOG, right
1
2
3
4
5
6
7
8
9
10
11
12
13
14
15
16
17
18
19
20
21
22
23
24
25
26
27
28
29
30
31
32
33
34
35
36
37
38
39
40
41
42
43
44
45
46
47
48
49
50
51
52
53
54
55
56
57
58
59
60
500 µV
-8
-6
-4
-2
0
2
time (s)

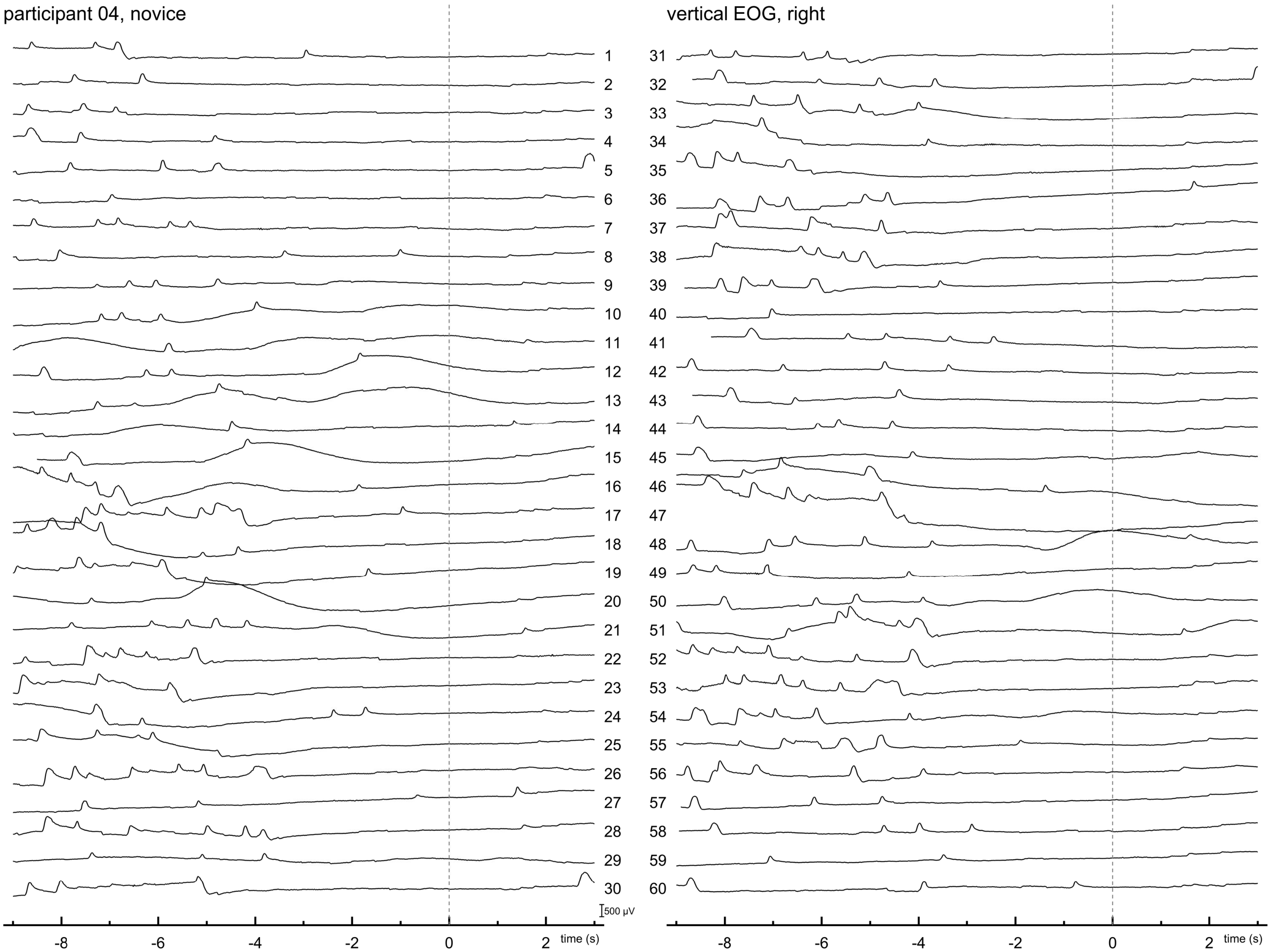
participant 04, novice
vertical EOG, right
500 µV
time (s)
-8
-6
-4
-2
0
2

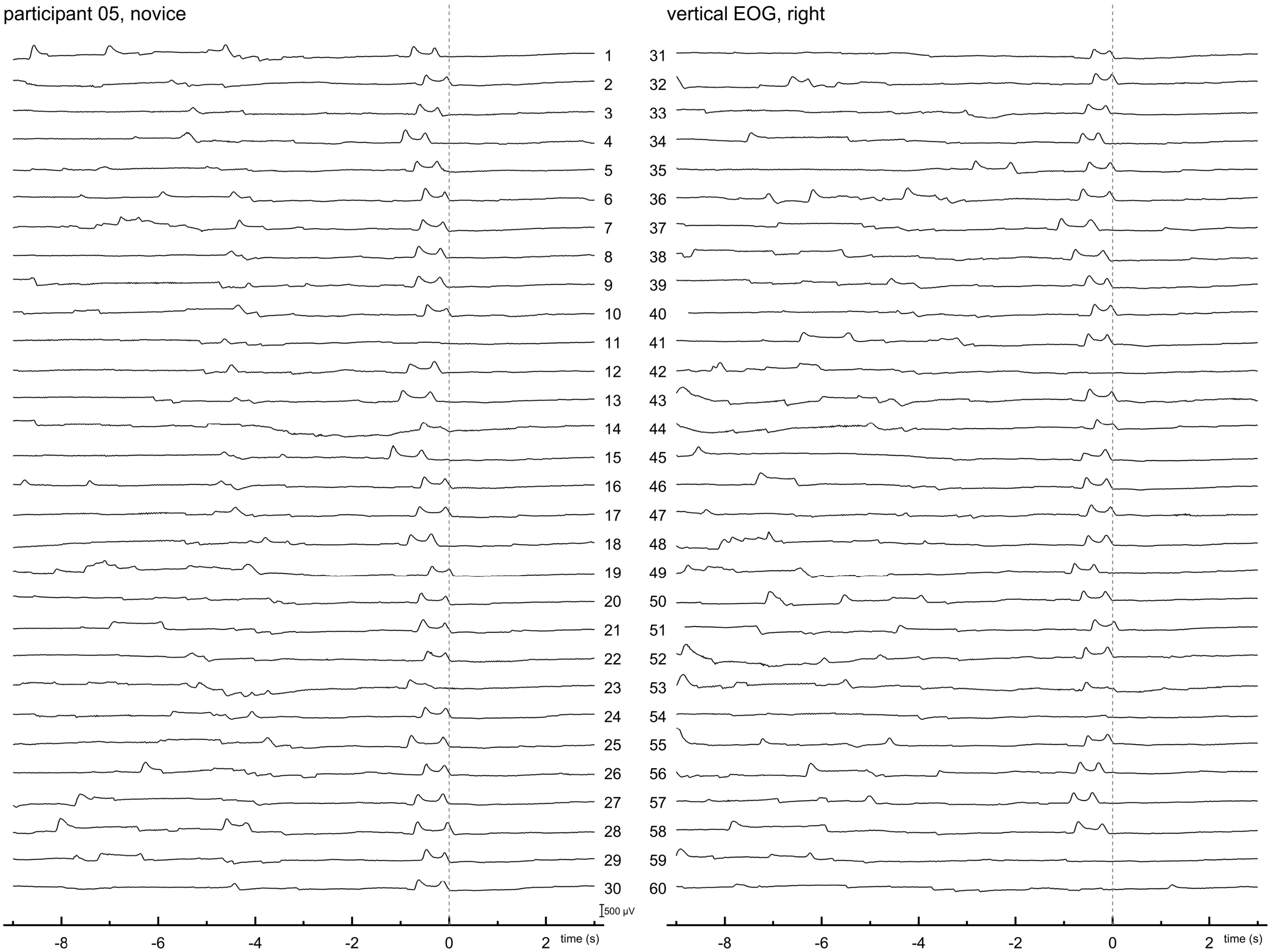
participant 05, novice
vertical EOG, right
1
2
3
4
5
6
7
8
9
10
11
12
13
14
15
16
17
18
19
20
21
22
23
24
25
26
27
28
29
30
31
32
33
34
35
36
37
38
39
40
41
42
43
44
45
46
47
48
49
50
51
52
53
54
55
56
57
58
59
60
500 µV
-8
-6
-4
-2
0
2
time (s)

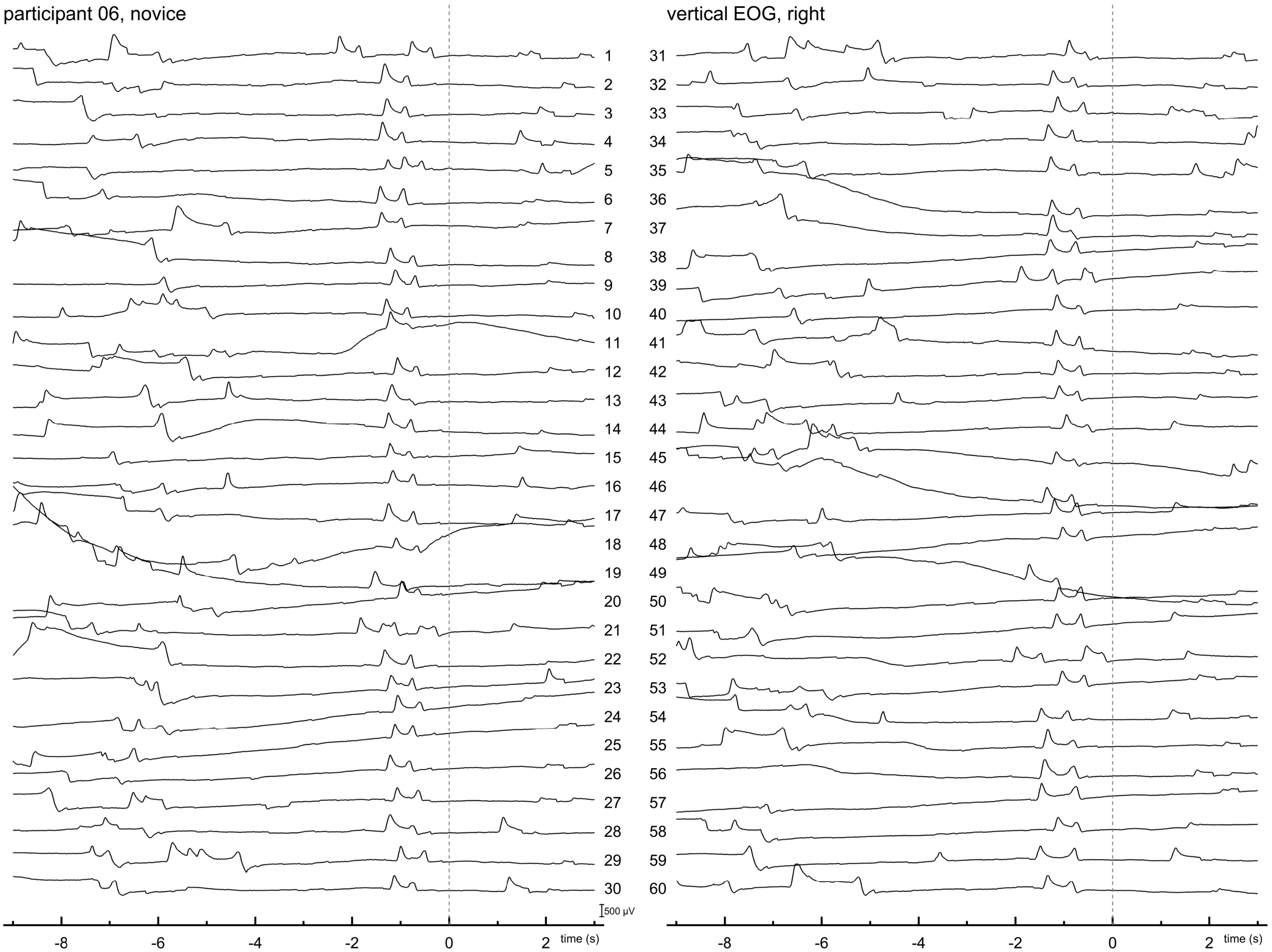
participant 06, novice
vertical EOG, right
1
2
3
4
5
6
7
8
9
10
11
12
13
14
15
16
17
18
19
20
21
22
23
24
25
26
27
28
29
30
31
32
33
34
35
36
37
38
39
40
41
42
43
44
45
46
47
48
49
50
51
52
53
54
55
56
57
58
59
60
500 µV
-8
-6
-4
-2
0
2
time (s)

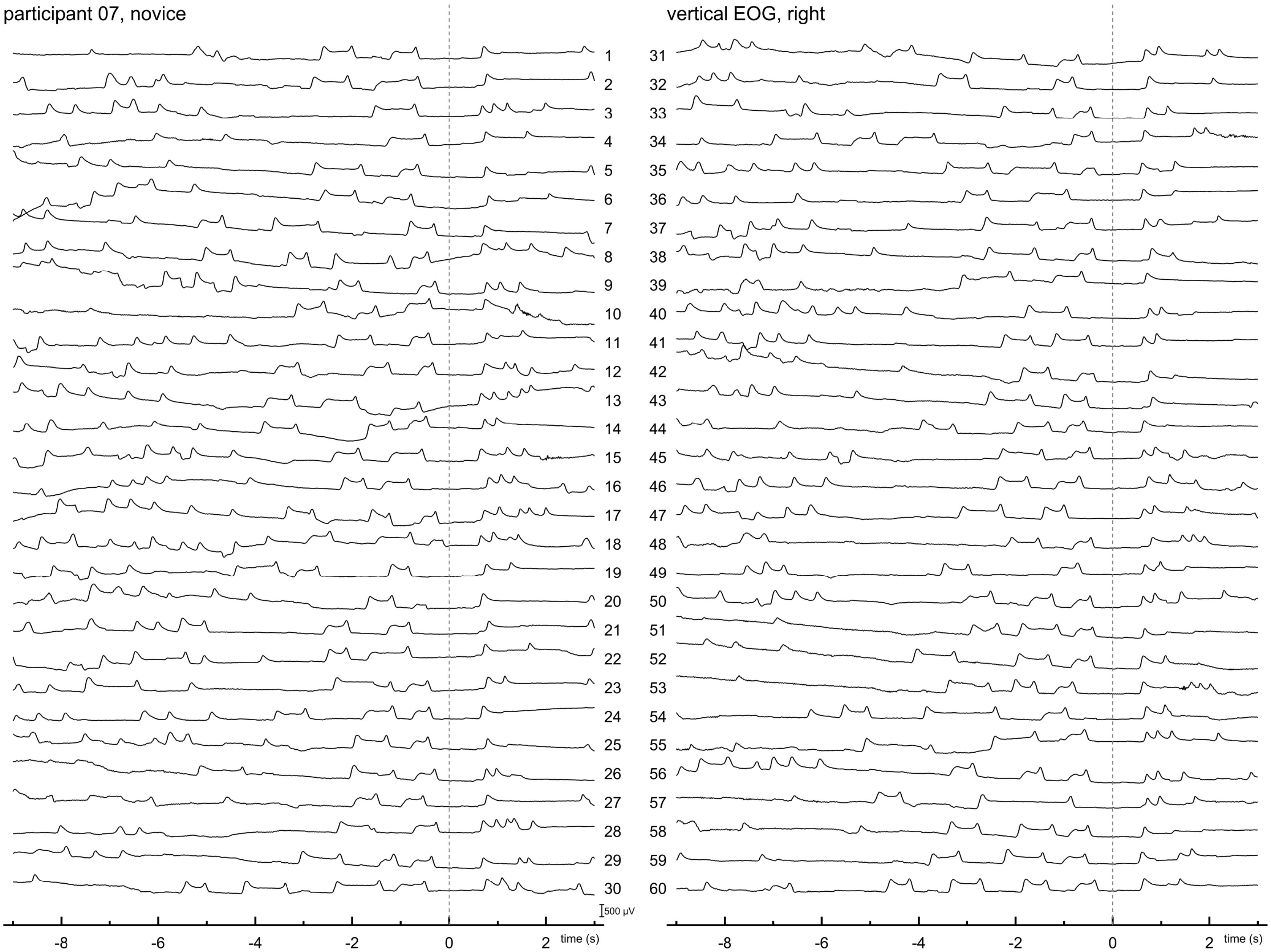

participant 07, novice
vertical EOG, right
1
2
3
4
5
6
7
8
9
10
11
12
13
14
15
16
17
18
19
20
21
22
23
24
25
26
27
28
29
30
31
32
33
34
35
36
37
38
39
40
41
42
43
44
45
46
47
48
49
50
51
52
53
54
55
56
57
58
59
60
500 µV
-8
-6
-4
-2
0
2
time (s)

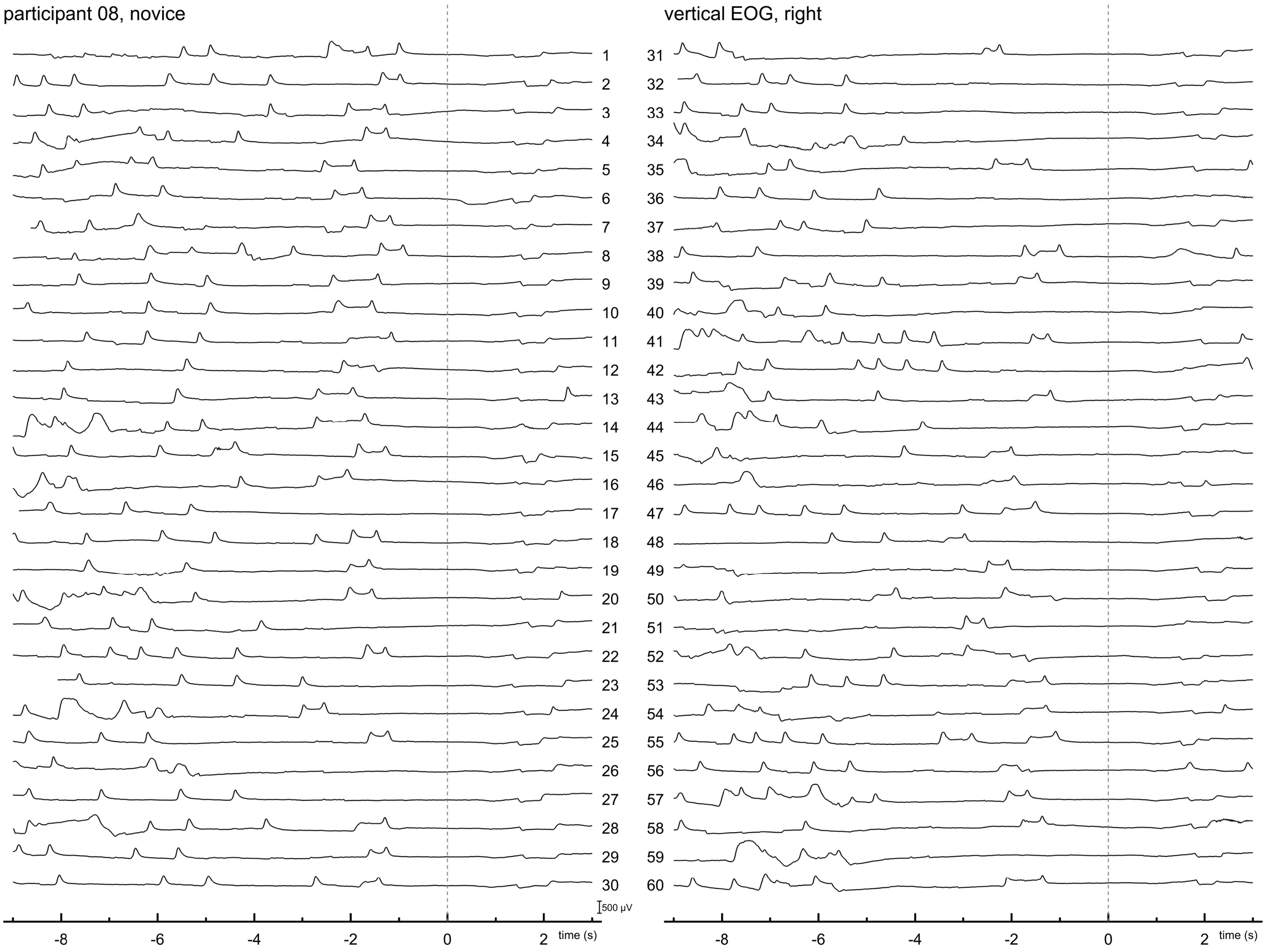
participant 08, novice
vertical EOG, right
500 µV
time (s)
-8
-6
-4
-2
0
2

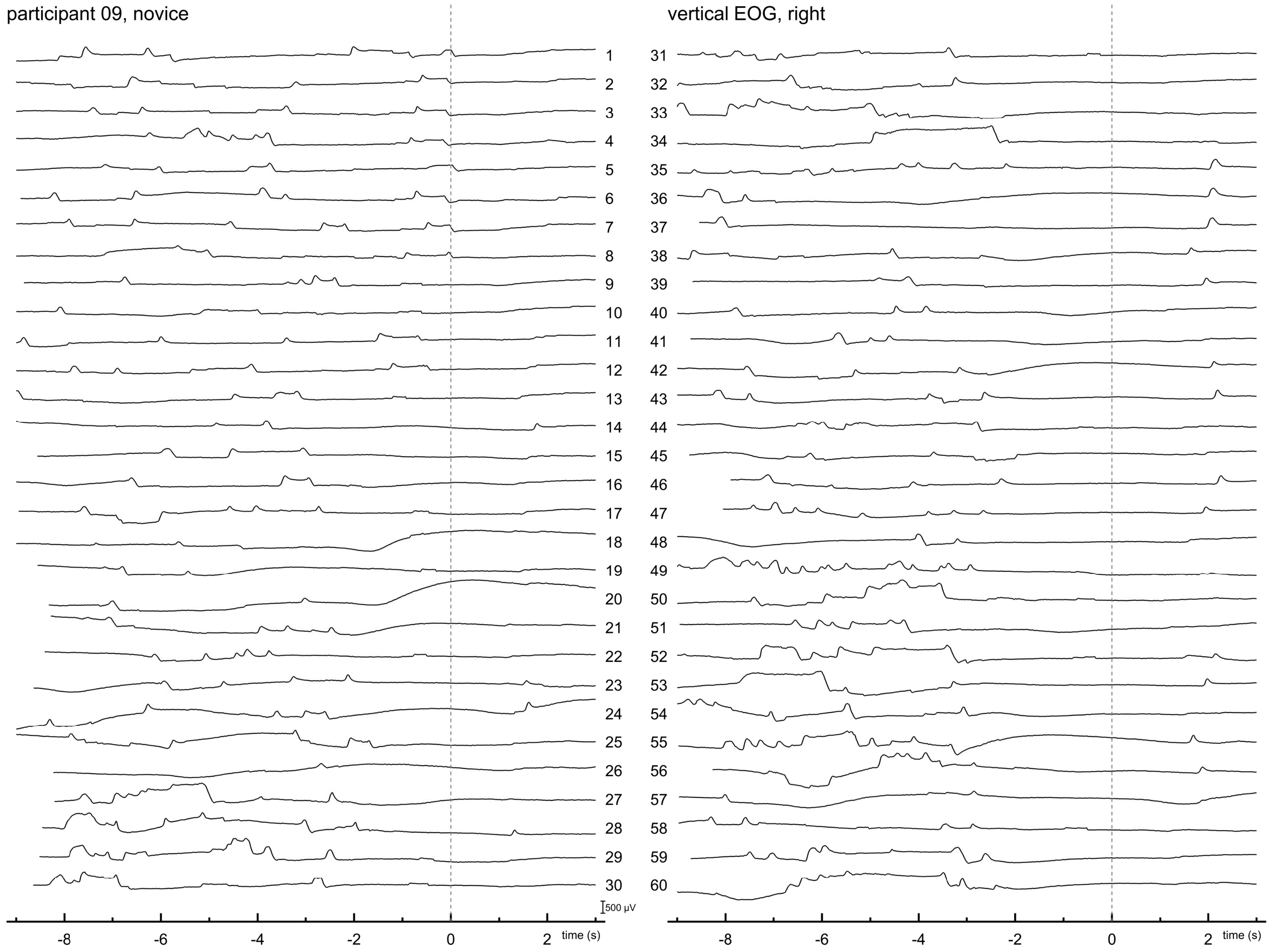
participant 09, novice
vertical EOG, right
1
2
3
4
5
6
7
8
9
10
11
12
13
14
15
16
17
18
19
20
21
22
23
24
25
26
27
28
29
30
31
32
33
34
35
36
37
38
39
40
41
42
43
44
45
46
47
48
49
50
51
52
53
54
55
56
57
58
59
60
500 µV
-8
-6
-4
-2
0
2
time (s)
-8
-6
-4
-2
0
2
time (s)

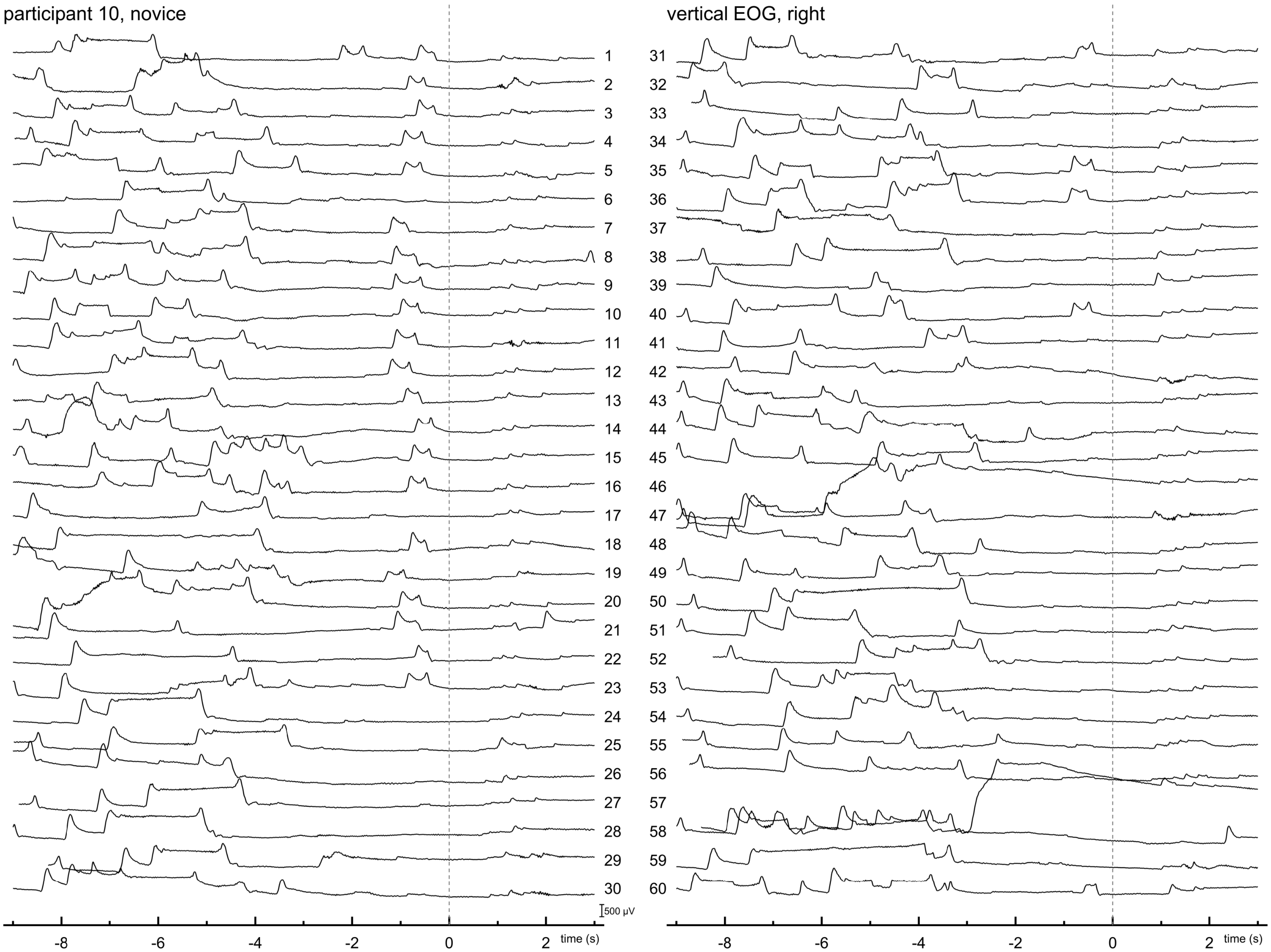

participant 10, novice
vertical EOG, right
500 µV
time (s)
-8
-6
-4
-2
0
2

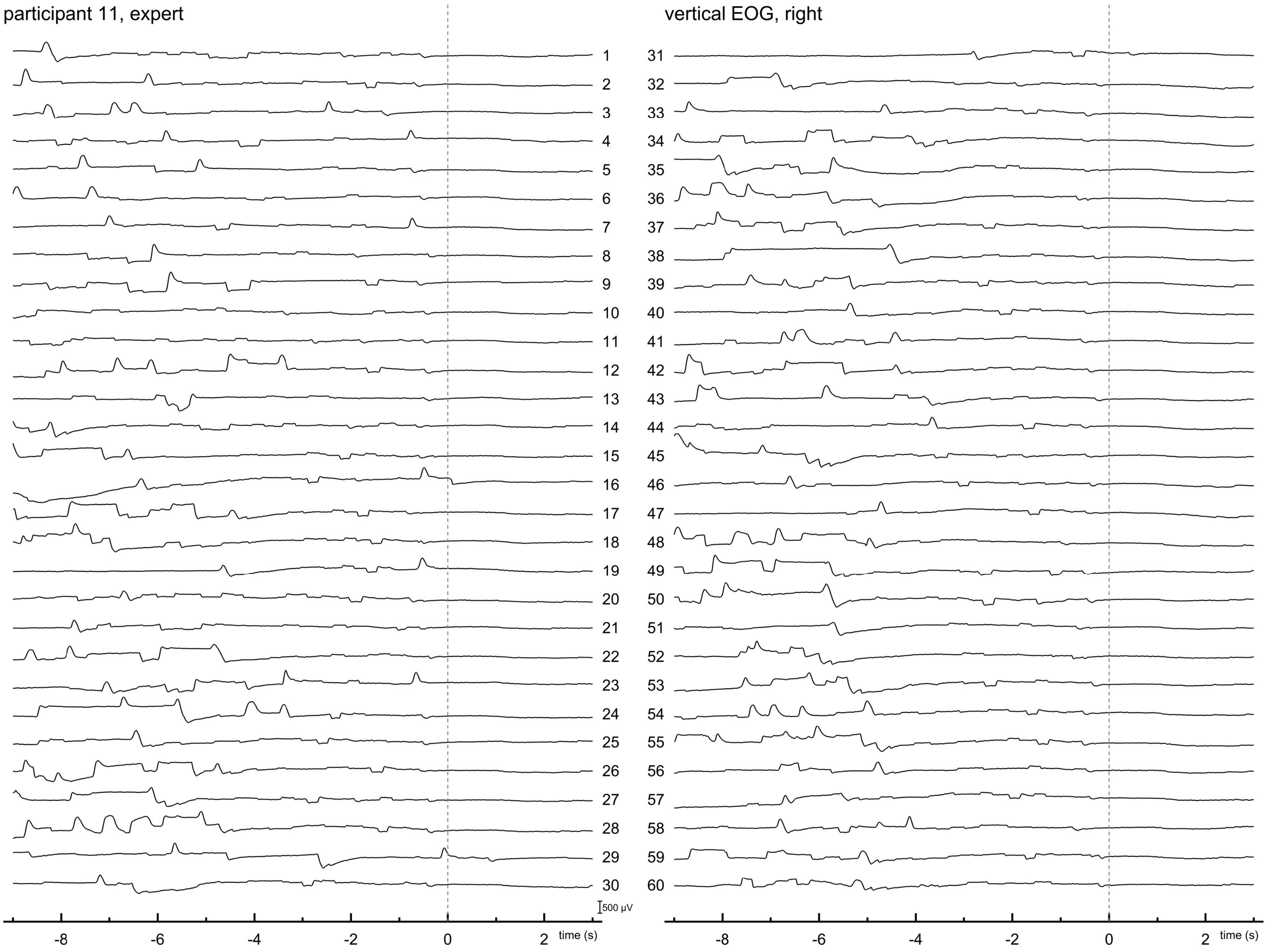
participant 11, expert
vertical EOG, right
1
2
3
4
5
6
7
8
9
10
11
12
13
14
15
16
17
18
19
20
21
22
23
24
25
26
27
28
29
30
31
32
33
34
35
36
37
38
39
40
41
42
43
44
45
46
47
48
49
50
51
52
53
54
55
56
57
58
59
60
500 µV
-8
-6
-4
-2
0
2
time (s)

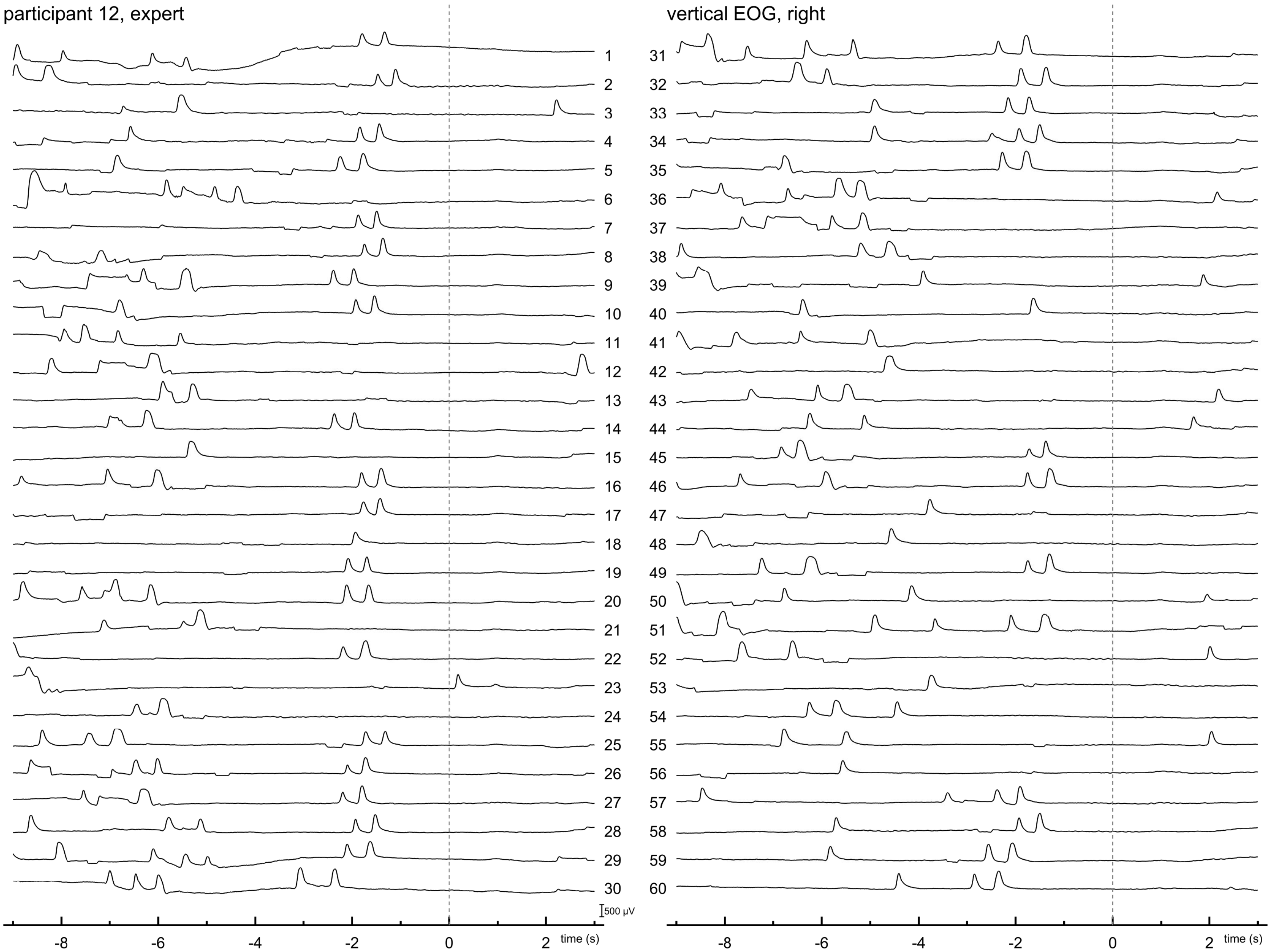
participant 12, expert
vertical EOG, right
1 2 3 4 5 6 7 8 9 10 11 12 13 14 15 16 17 18 19 20 21 22 23 24 25 26 27 28 29 30
31 32 33 34 35 36 37 38 39 40 41 42 43 44 45 46 47 48 49 50 51 52 53 54 55 56 57 58 59 60
500 µV
-8 -6 -4 -2 0 2 time (s)
-8 -6 -4 -2 0 2 time (s)

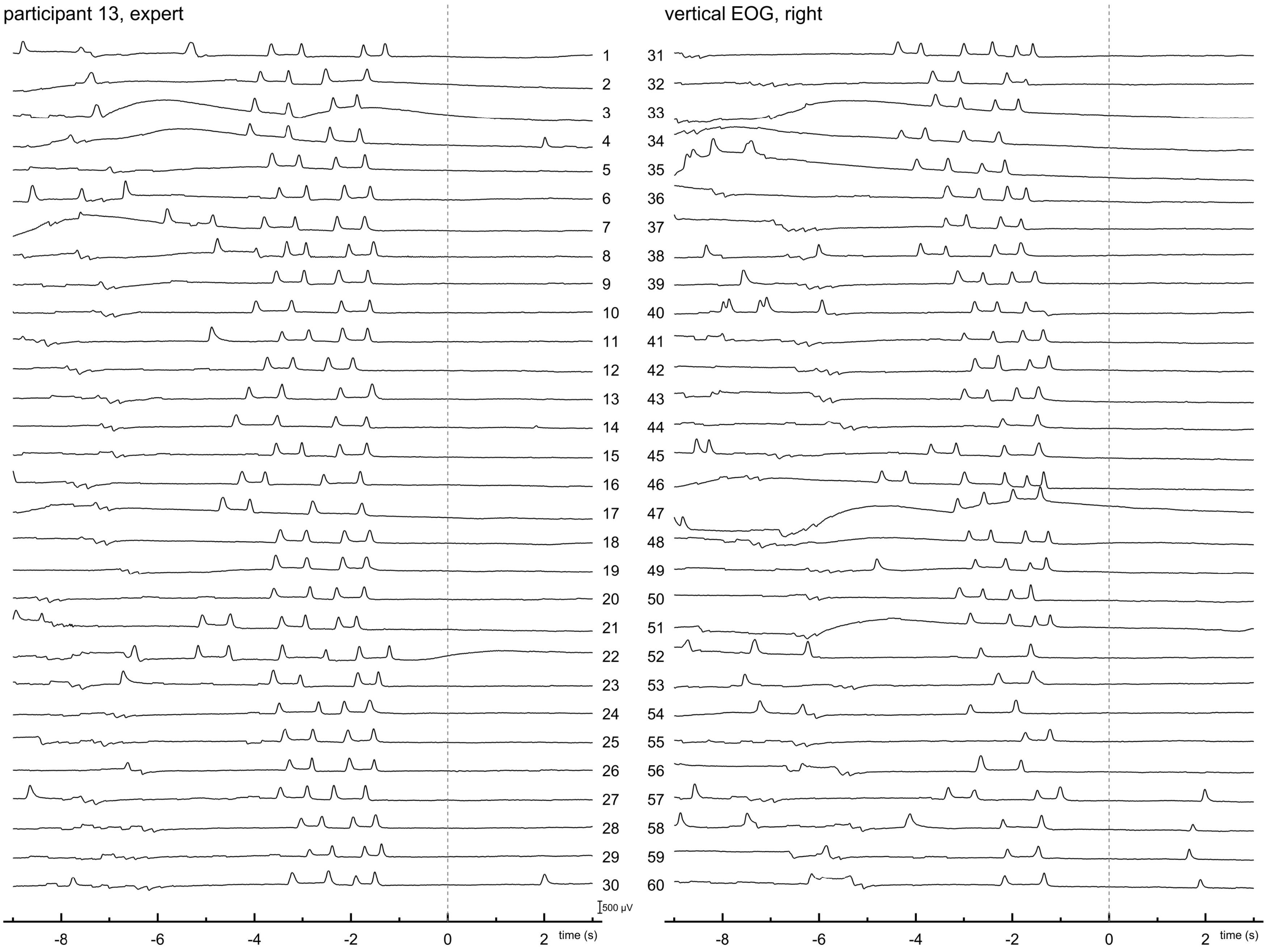
participant 13, expert
vertical EOG, right
1
2
3
4
5
6
7
8
9
10
11
12
13
14
15
16
17
18
19
20
21
22
23
24
25
26
27
28
29
30
31
32
33
34
35
36
37
38
39
40
41
42
43
44
45
46
47
48
49
50
51
52
53
54
55
56
57
58
59
60
500 µV
-8
-6
-4
-2
0
2
time (s)

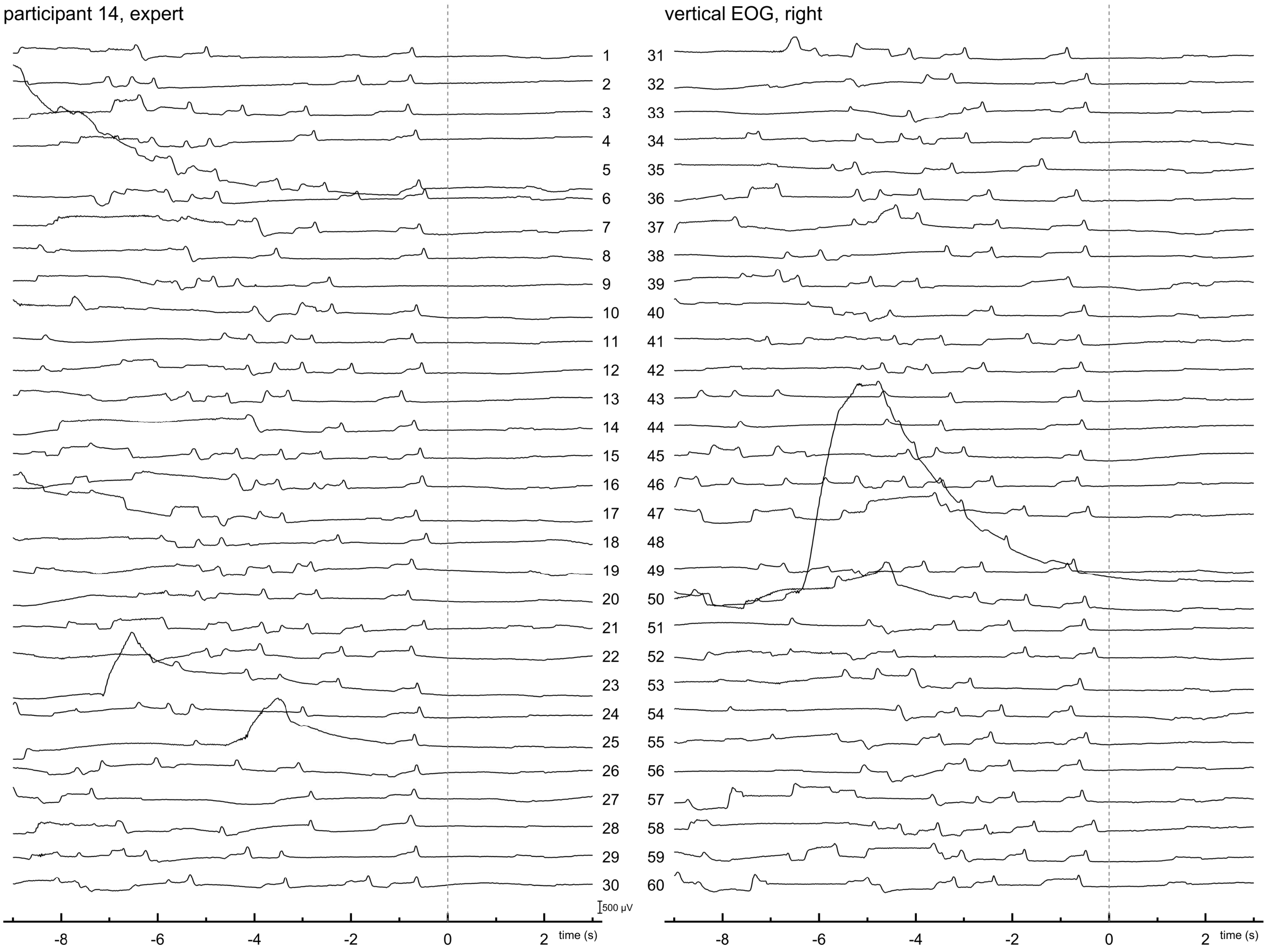
participant 14, expert
vertical EOG, right
1 2 3 4 5 6 7 8 9 10 11 12 13 14 15 16 17 18 19 20 21 22 23 24 25 26 27 28 29 30
31 32 33 34 35 36 37 38 39 40 41 42 43 44 45 46 47 48 49 50 51 52 53 54 55 56 57 58 59 60
500 µV
-8 -6 -4 -2 0 2
time (s)

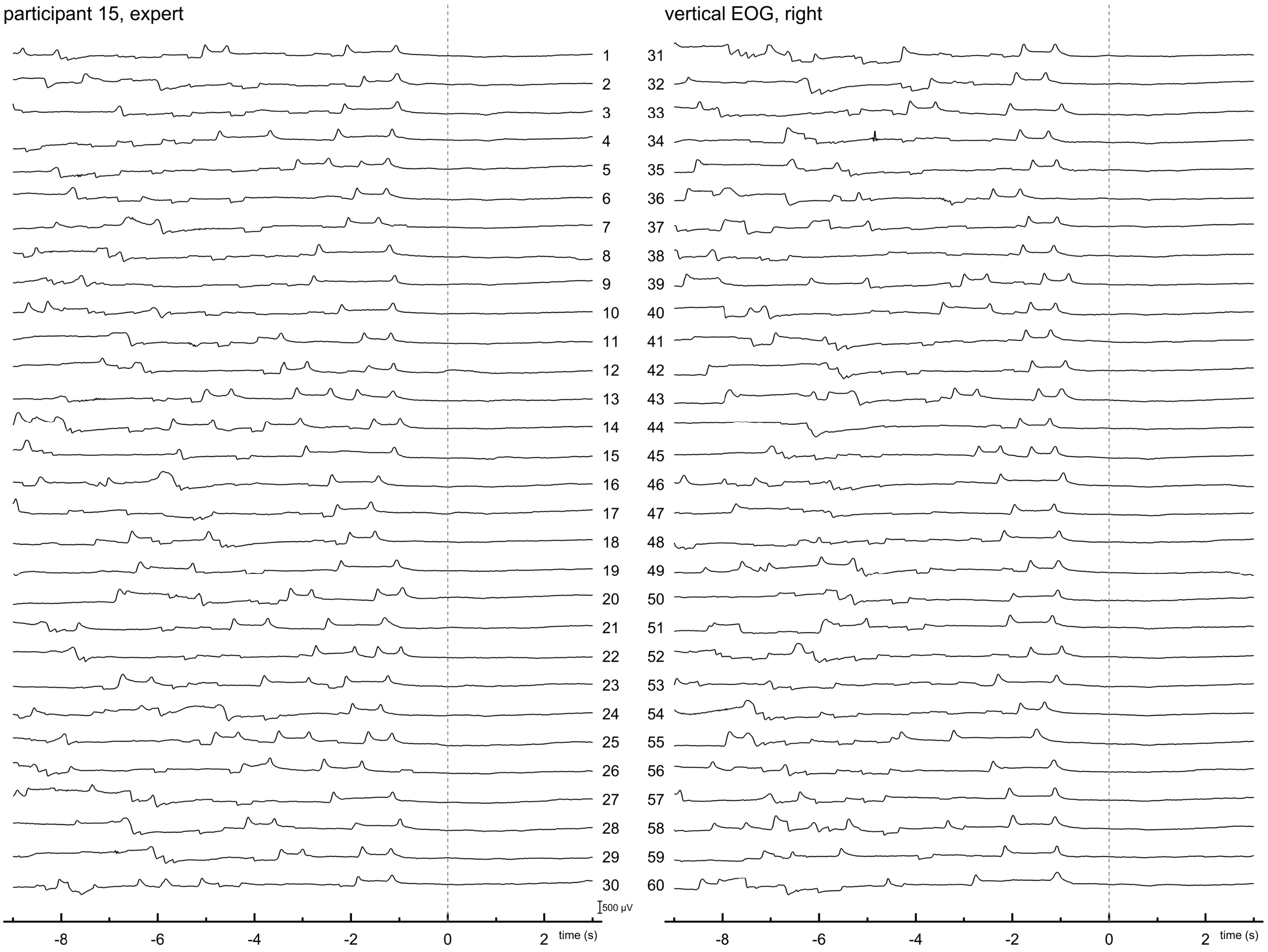

participant 15, expert
vertical EOG, right
500 µV
time (s)

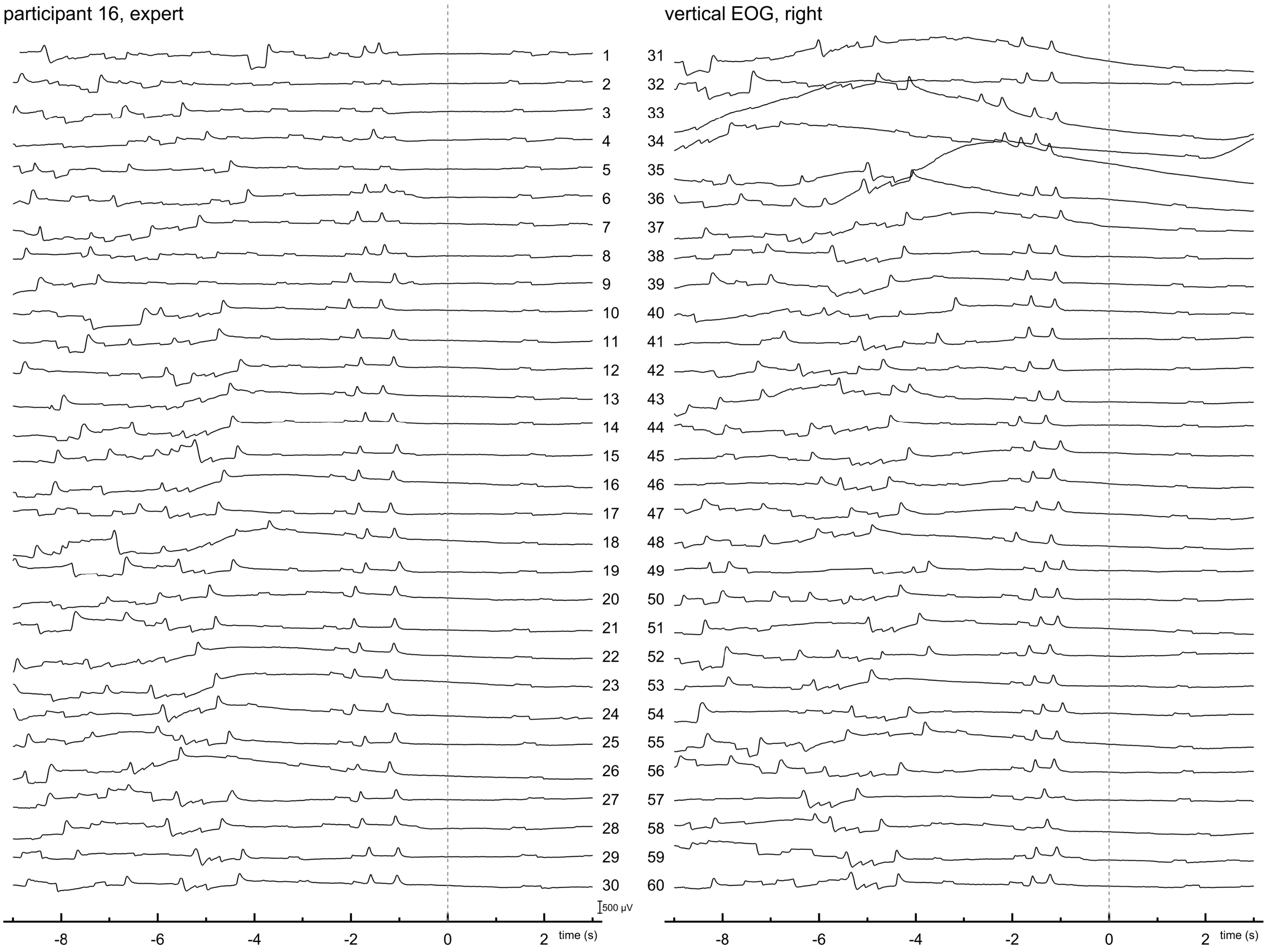
participant 16, expert
vertical EOG, right
1
2
3
4
5
6
7
8
9
10
11
12
13
14
15
16
17
18
19
20
21
22
23
24
25
26
27
28
29
30
31
32
33
34
35
36
37
38
39
40
41
42
43
44
45
46
47
48
49
50
51
52
53
54
55
56
57
58
59
60
500 µV
-8
-6
-4
-2
0
2
time (s)

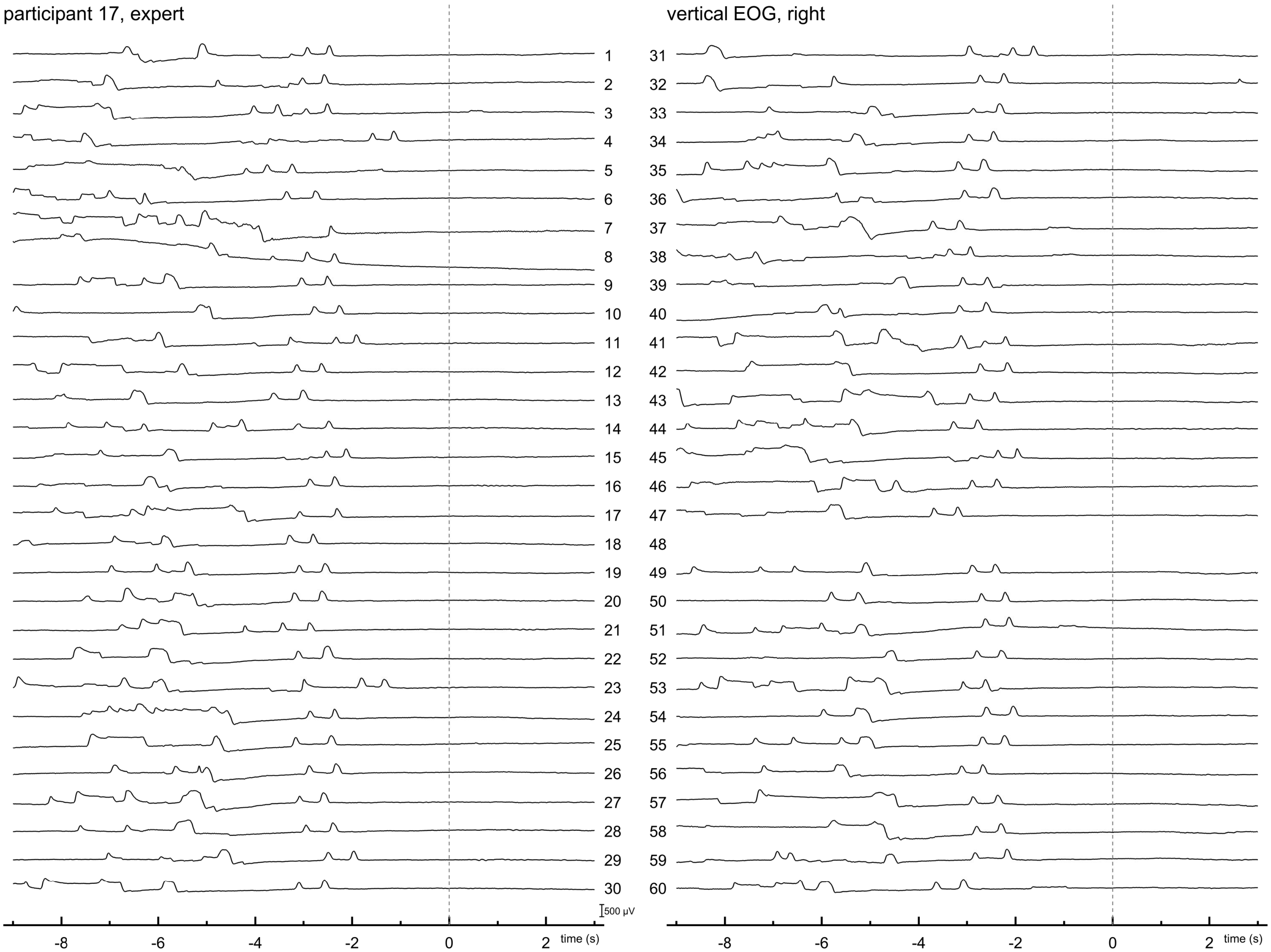
participant 17, expert
vertical EOG, right
1 2 3 4 5 6 7 8 9 10 11 12 13 14 15 16 17 18 19 20 21 22 23 24 25 26 27 28 29 30
31 32 33 34 35 36 37 38 39 40 41 42 43 44 45 46 47 48 49 50 51 52 53 54 55 56 57 58 59 60
500 µV
-8
-6
-4
-2
0
2
time (s)

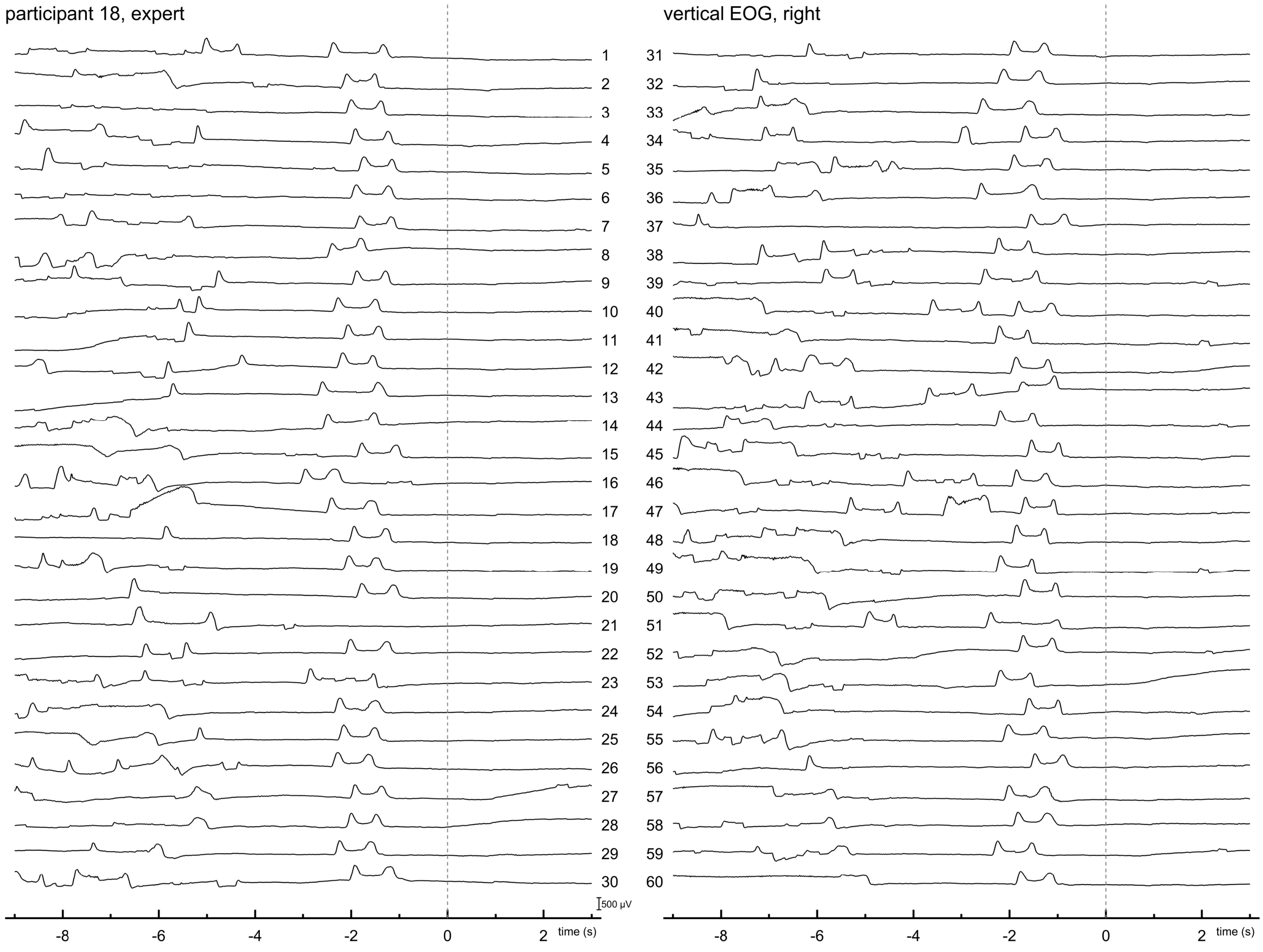

participant 18, expert
vertical EOG, right
500 µV
time (s)
-8
-6
-4
-2
0
2

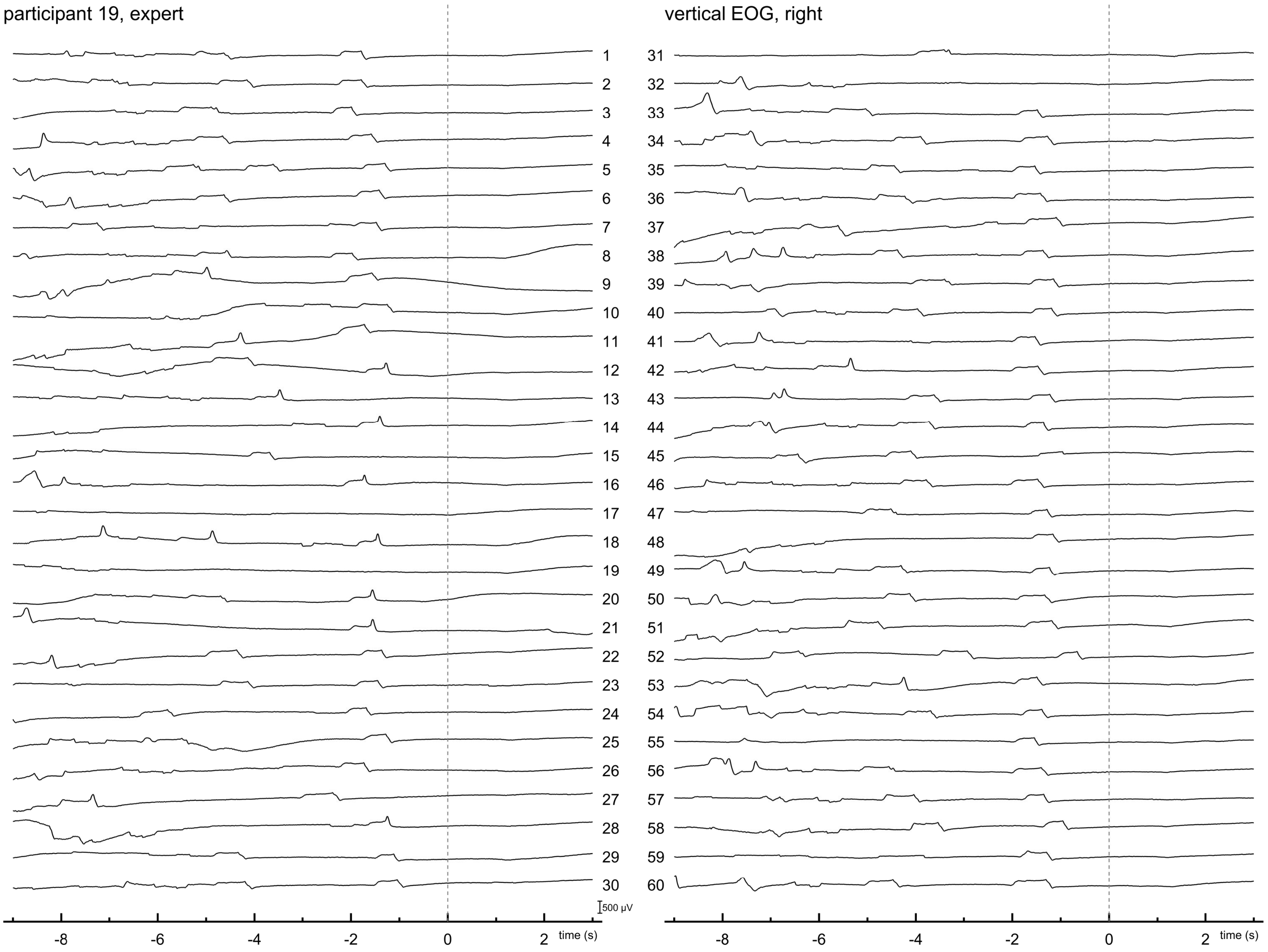

participant 19, expert
vertical EOG, right
500 µV
time (s)
-8
-6
-4
-2
0
2

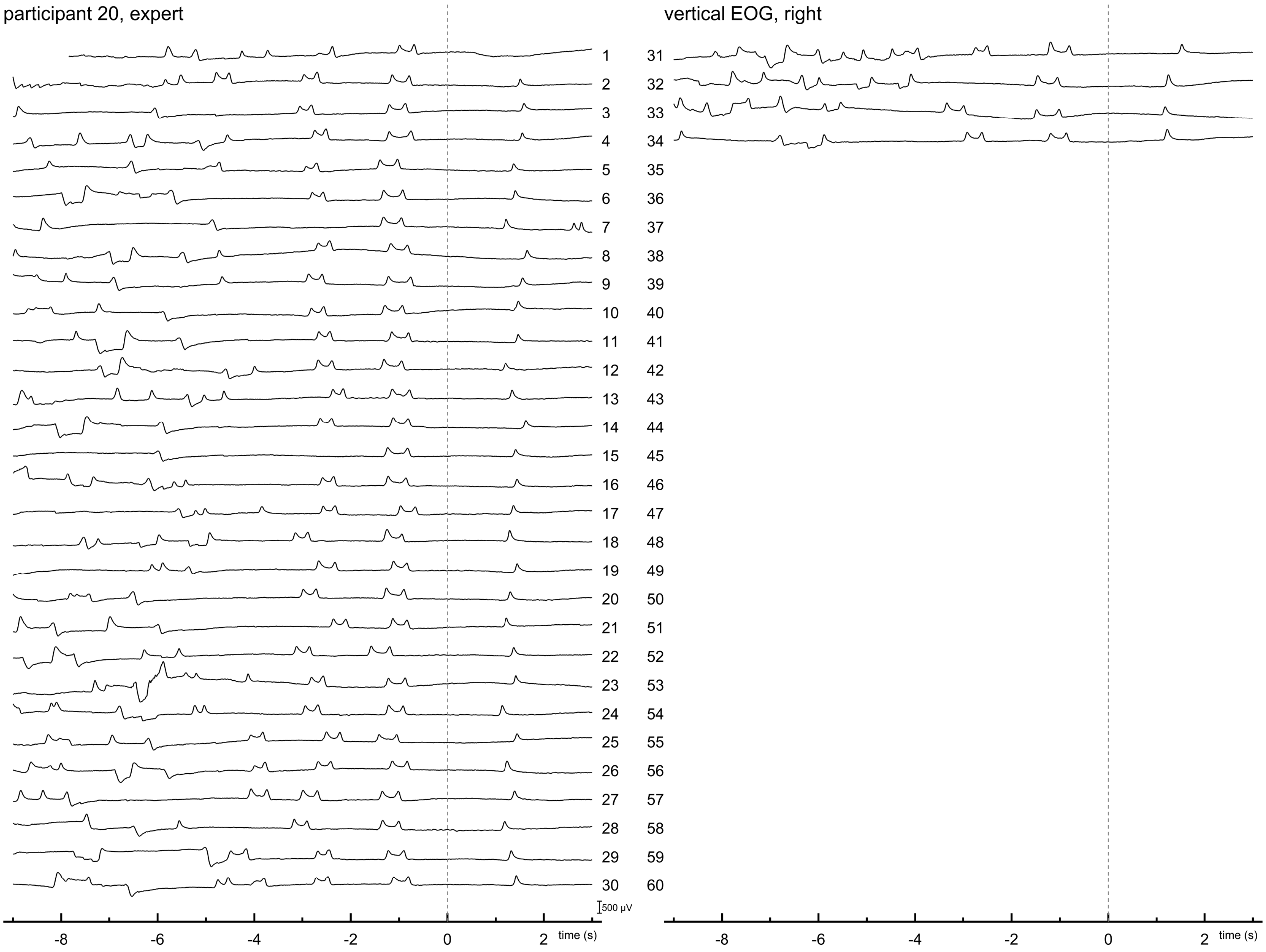
participant 20, expert
vertical EOG, right
1
2
3
4
5
6
7
8
9
10
11
12
13
14
15
16
17
18
19
20
21
22
23
24
25
26
27
28
29
30
31
32
33
34
35
36
37
38
39
40
41
42
43
44
45
46
47
48
49
50
51
52
53
54
55
56
57
58
59
60
500 µV
-8
-6
-4
-2
0
2
time (s)
-8
-6
-4
-2
0
2
time (s)
