# Supplementary material for: Assessing ocular activity during performance of motor skills using electrooculography
Source: Psychophysiology. 2018 Feb 9;55(7):e13070. doi: 10.1111/psyp.13070 (PMC6849535; doi:10.1111/psyp.13070)
Supplement: Supplementary file 2 — Appendix S2 [file PSYP-55-na-s002.pdf]

Gallicchio, G., Cooke, A., & Ring, C. Assessing ocular activity during performance of motor skills using electrooculography. *Psychophysiology*
Supplement S2

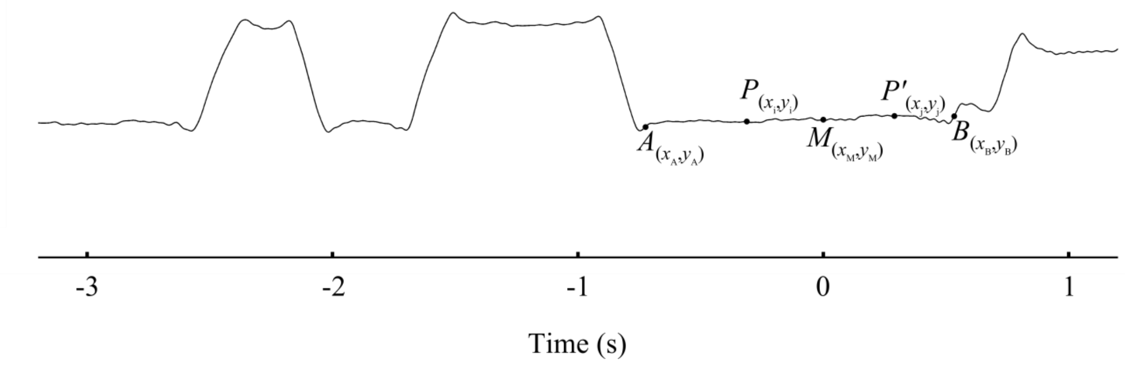

The figure shows the horizontal EOG signal relative to a golf putt. The point $M(x_M, y_M)$ identifies movement initiation (i.e., the beginning of the backswing): x_M identifies the time of movement initiation and y_M identifies the voltage corresponding to that time.

The voltage-threshold algorithm is composed of two parts. The first part identifies QE onset by comparing the voltage y_M with the voltages in each of the preceding time points. Specifically, a point $P(x_i, y_i)$ is identified as the time-point immediately preceding x_M (i.e., $x_i = x_M - 1$). The voltage y_i is compared with the reference voltage y_M. If their difference is within a certain voltage threshold th (i.e., $|y_i - y_M| \leq th$), then the point $P(x_i, y_i)$ is slid backwards (i.e., $x_i = x_i - 1$) and the new voltage y_i is compared with the reference voltage y_M. This procedure continues until the voltage difference exceeds the voltage threshold (i.e., $|y_i - y_M| > th$). The point $A(x_A, y_A)$ with $x_A = x_i + 1$, is the last point for which the voltage difference is within the threshold, and therefore is taken as the onset of the QE. The difference $x_M - x_A$ indicates QE_{pre}. If $x_M = x_A$ QE_{pre} is 0.

The second part identifies QE offset in the same way, but using time points that follow movement initiation. A point $P(x_j, y_j)$ is identified as the time-point immediately following x_M (i.e., $x_j = x_M + 1$). The voltage y_j is compared with the reference voltage y_M. If their difference is within a certain voltage threshold th (i.e., $|y_j - y_M| \leq th$), then the point $P(x_i, y_i)$ is slid forwards (i.e., $x_j = x_j + 1$) and the new voltage y_j is compared with the reference voltage y_M. This procedure continues until the voltage difference exceeds the voltage threshold (i.e., $|y_j - y_M| > th$). The point $B(x_B, y_B)$ with $x_B = x_j - 1$, is the last point for which the voltage difference is within the threshold, and therefore is taken as the offset of the QE. The difference $x_B - x_M$ indicates QE_{post}. If $x_M = x_B$ QE_{post} is 0.
