# Supplementary material for: Assessing ocular activity during performance of motor skills using electrooculography
Source: Psychophysiology. 2018 Feb 9;55(7):e13070. doi: 10.1111/psyp.13070 (PMC6849535; doi:10.1111/psyp.13070)
Supplement: Supplementary file 3 — Appendix S3 [file PSYP-55-na-s003.pdf]

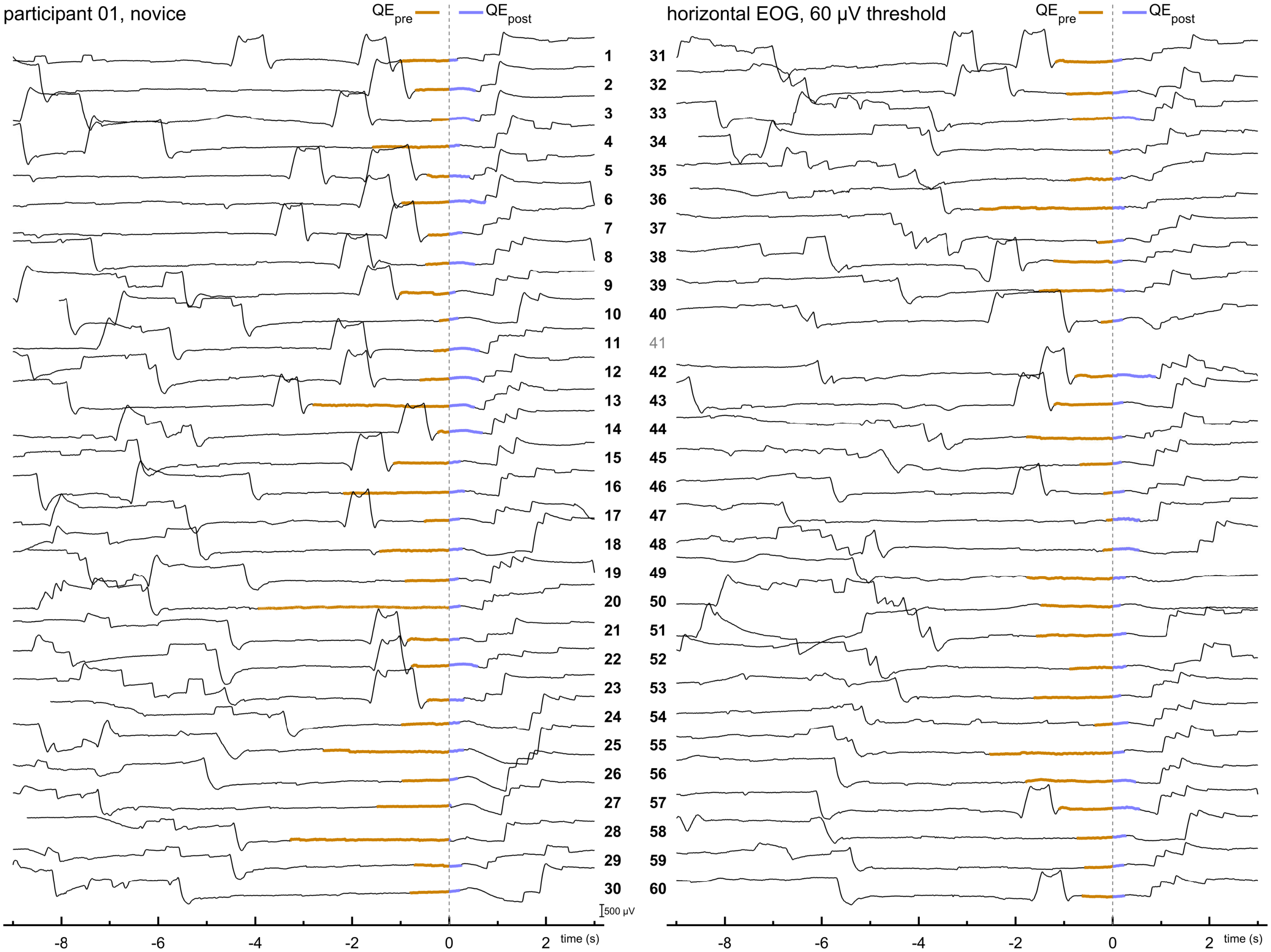

participant 01, novice
horizontal EOG, 60 µV threshold
QE pre
QE post
500 µV
time (s)
-8
-6
-4
-2
0
2
1
2
3
4
5
6
7
8
9
10
11
12
13
14
15
16
17
18
19
20
21
22
23
24
25
26
27
28
29
30
31
32
33
34
35
36
37
38
39
40
41
42
43
44
45
46
47
48
49
50
51
52
53
54
55
56
57
58
59
60

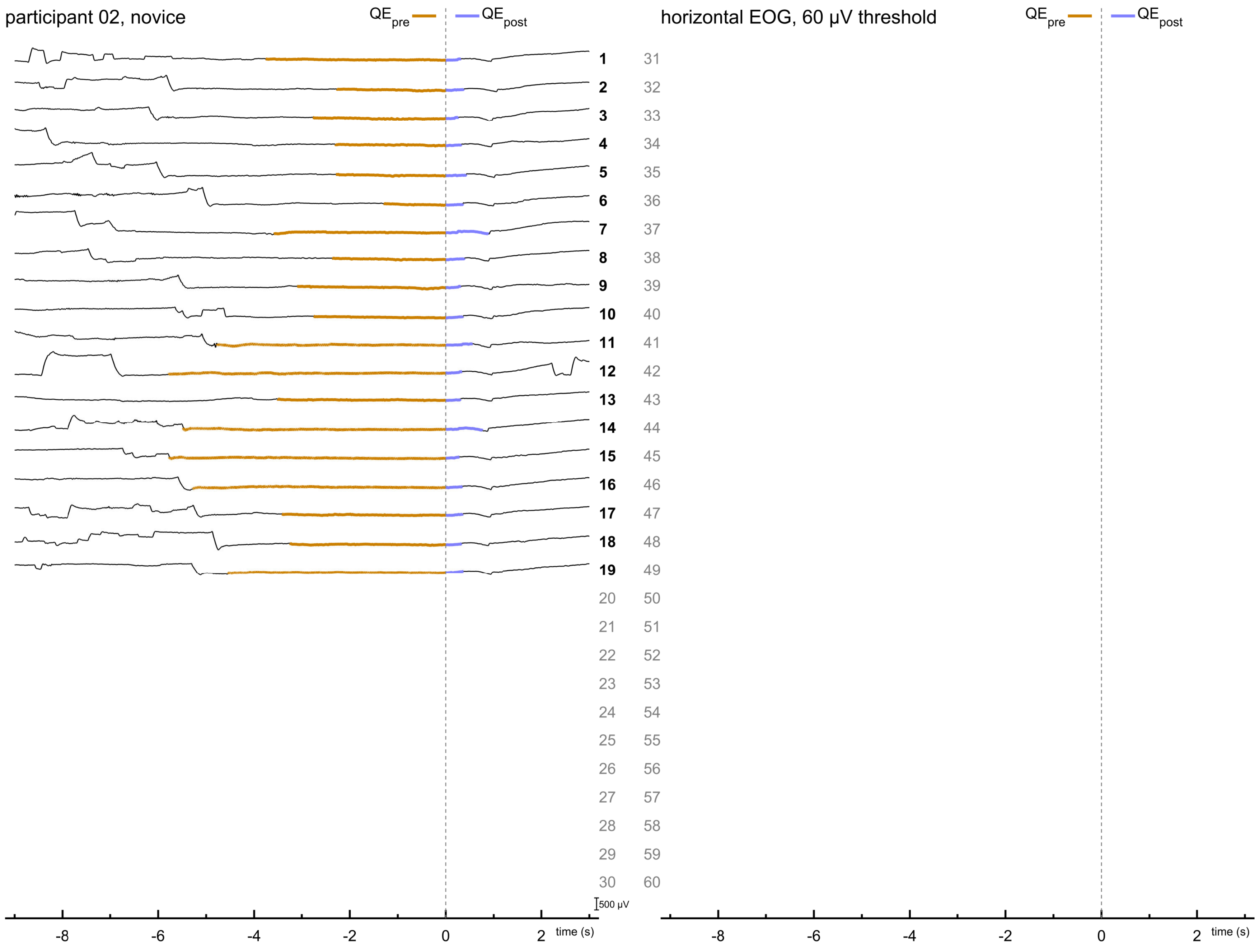
participant 02, novice
horizontal EOG, 60 μV threshold
QEpre
QEpost
500 μV
time (s)
-8
-6
-4
-2
0
2

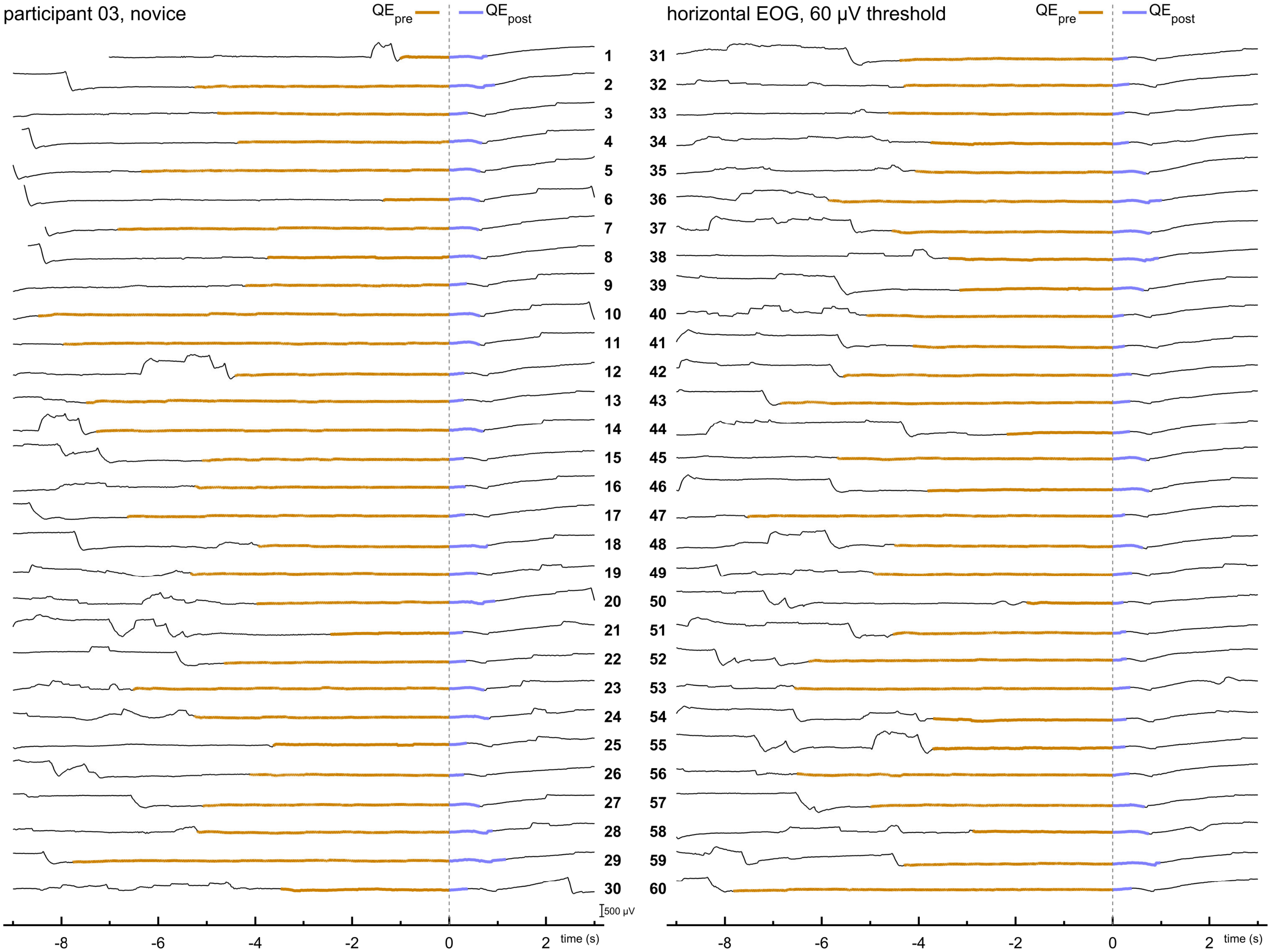

participant 03, novice
horizontal EOG, 60 µV threshold
QEpre
QEpost
500 µV
time (s)
-8
-6
-4
-2
0
2

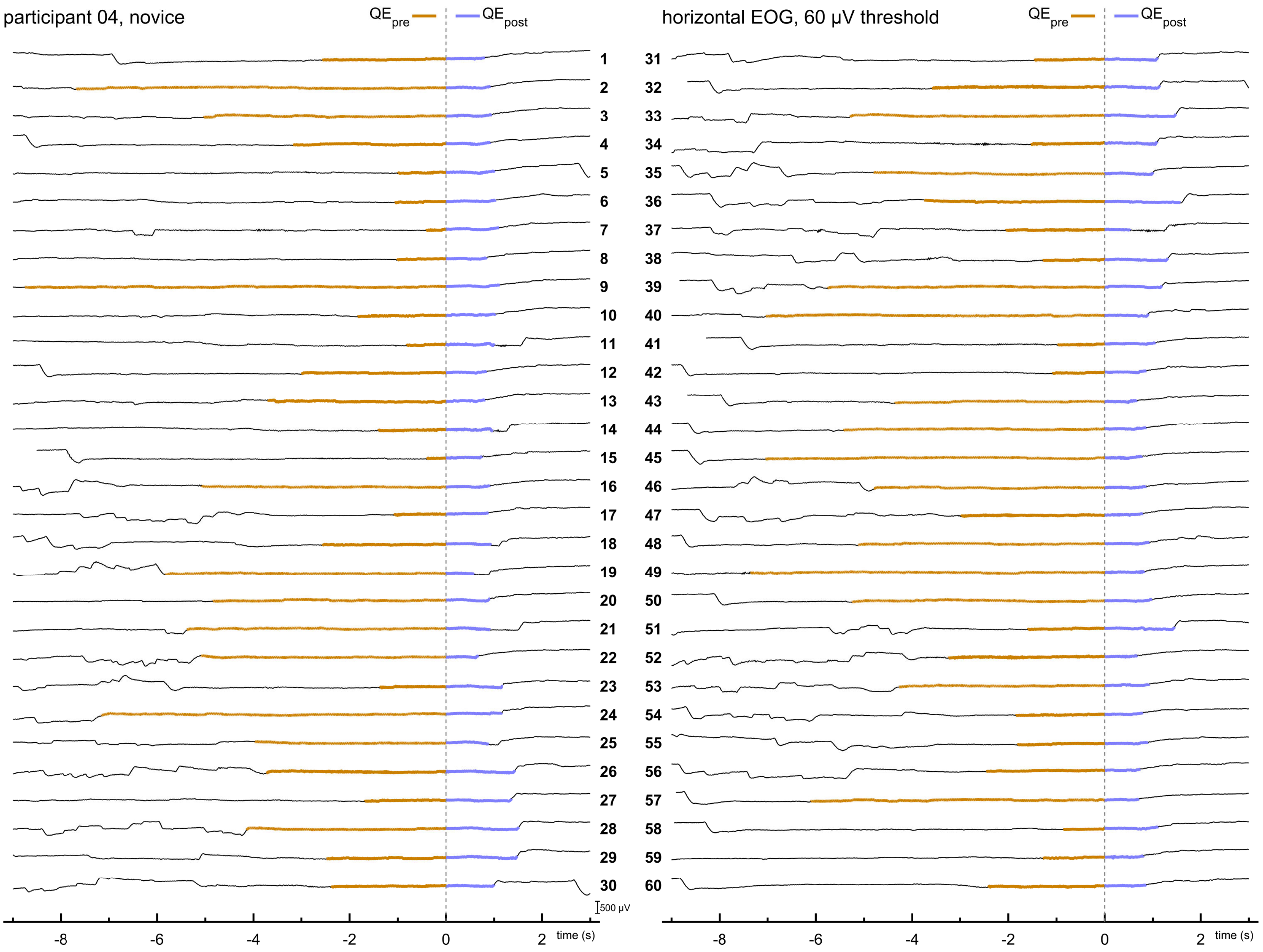

participant 04, novice
horizontal EOG, 60 µV threshold
QE_{pre}
QE_{post}
500 µV
time (s)
-8
-6
-4
-2
0
2

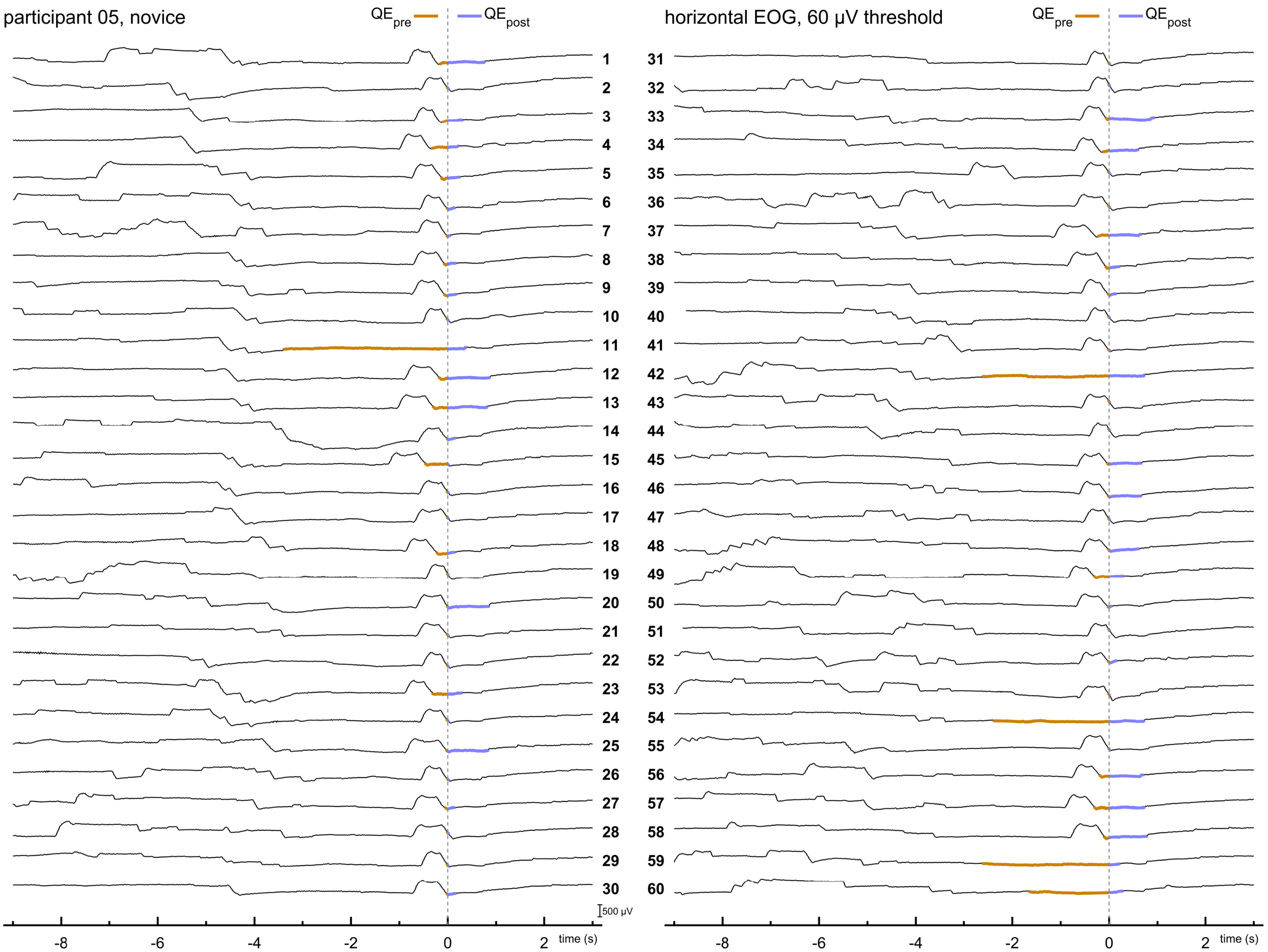
participant 05, novice
horizontal EOG, 60 µV threshold
QEpre
QEpost
500 µV
time (s)
-8
-6
-4
-2
0
2
1
2
3
4
5
6
7
8
9
10
11
12
13
14
15
16
17
18
19
20
21
22
23
24
25
26
27
28
29
30
31
32
33
34
35
36
37
38
39
40
41
42
43
44
45
46
47
48
49
50
51
52
53
54
55
56
57
58
59
60

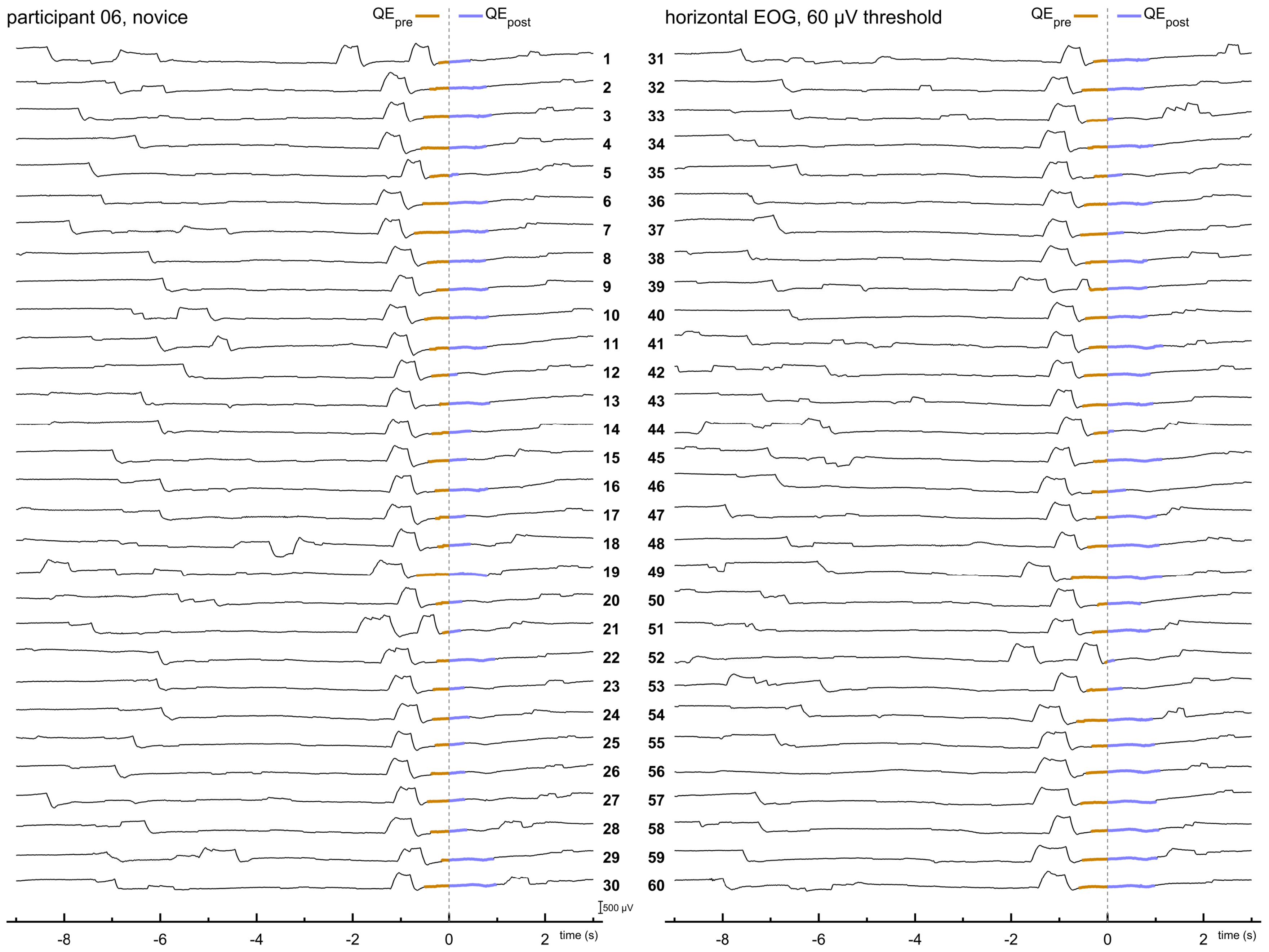

participant 06, novice
horizontal EOG, 60 µV threshold
QE pre
QE post
1 2 3 4 5 6 7 8 9 10 11 12 13 14 15 16 17 18 19 20 21 22 23 24 25 26 27 28 29 30
31 32 33 34 35 36 37 38 39 40 41 42 43 44 45 46 47 48 49 50 51 52 53 54 55 56 57 58 59 60
500 µV
-8 -6 -4 -2 0 2
time (s)

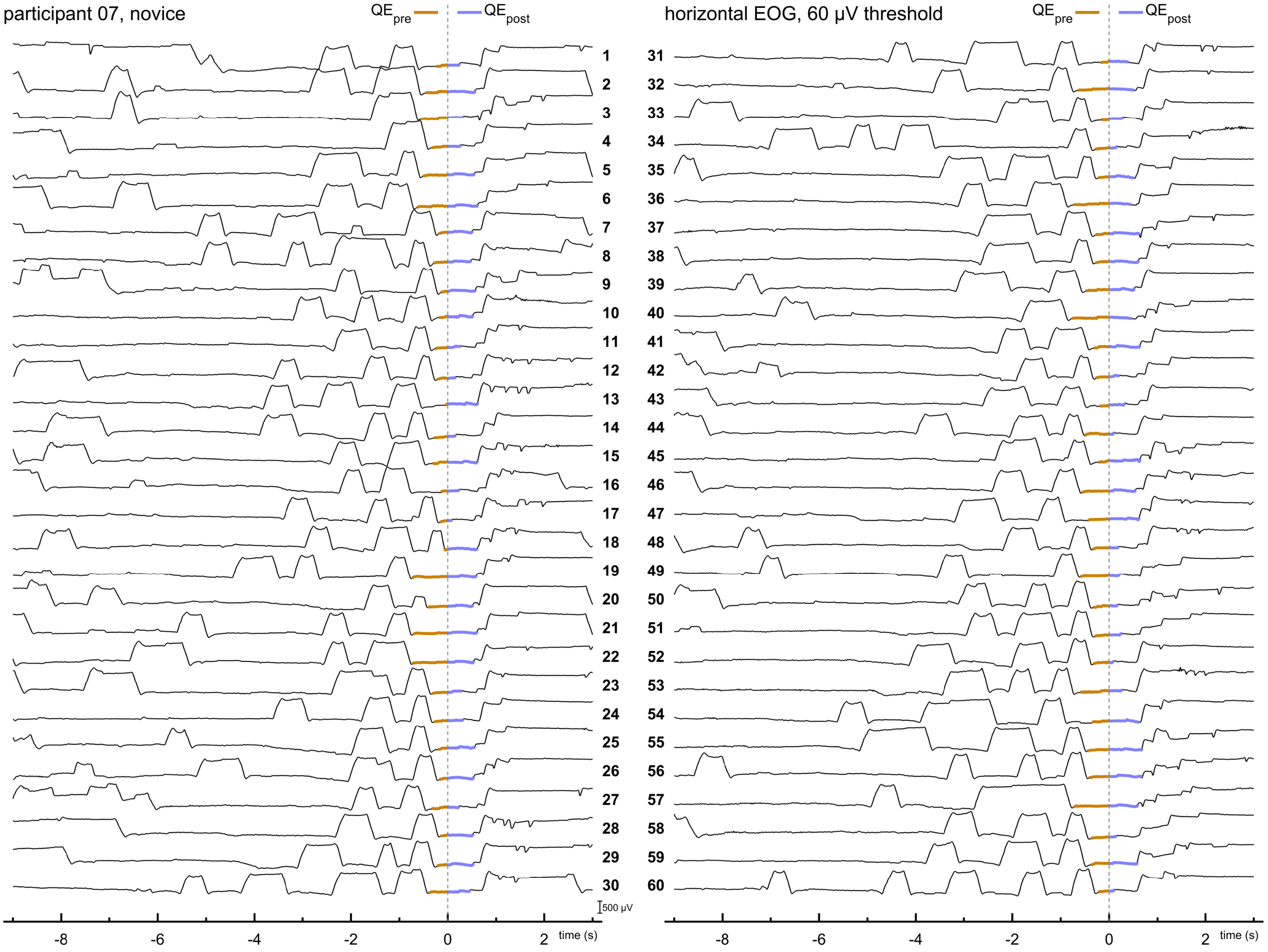

participant 07, novice
horizontal EOG, 60 µV threshold
QEpre
QEpost
1 2 3 4 5 6 7 8 9 10 11 12 13 14 15 16 17 18 19 20 21 22 23 24 25 26 27 28 29 30
31 32 33 34 35 36 37 38 39 40 41 42 43 44 45 46 47 48 49 50 51 52 53 54 55 56 57 58 59 60
500 µV
-8 -6 -4 -2 0 2
time (s)

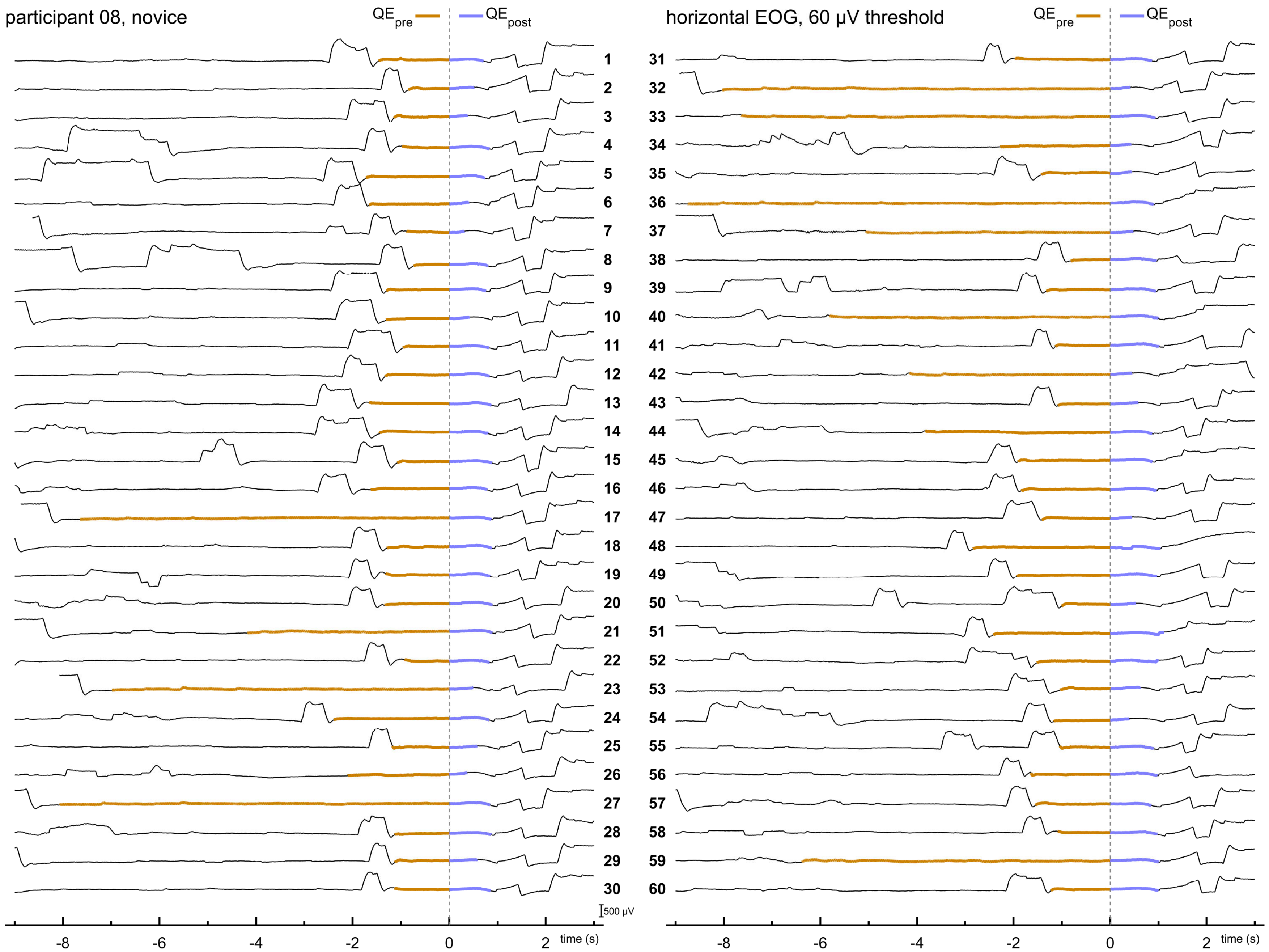
participant 08, novice
horizontal EOG, 60 µV threshold
QEpre
QEpost
1 2 3 4 5 6 7 8 9 10 11 12 13 14 15 16 17 18 19 20 21 22 23 24 25 26 27 28 29 30
31 32 33 34 35 36 37 38 39 40 41 42 43 44 45 46 47 48 49 50 51 52 53 54 55 56 57 58 59 60
500 µV
-8
-6
-4
-2
0
2
time (s)

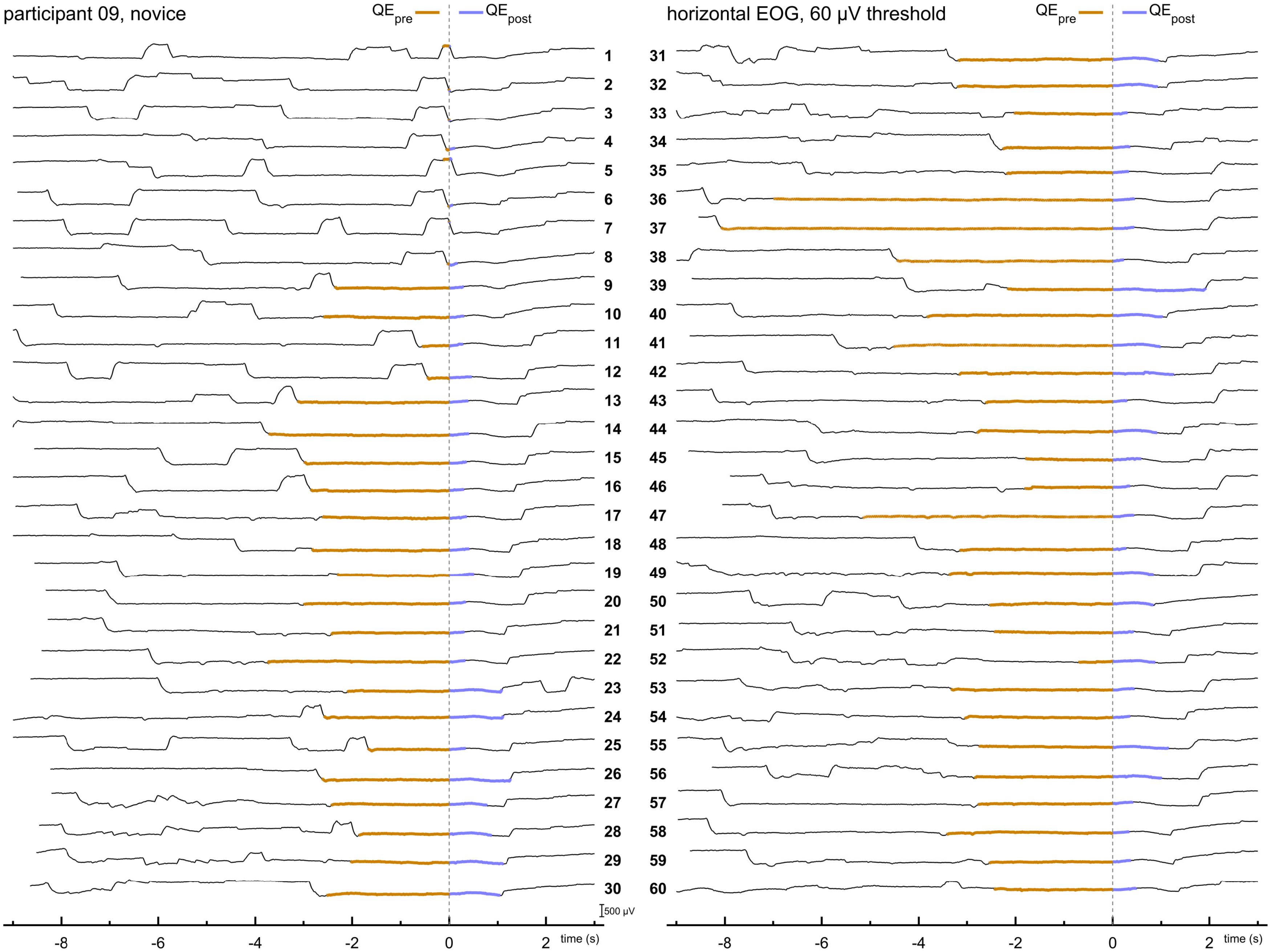

participant 09, novice
horizontal EOG, 60 µV threshold
QE pre
QE post
1
2
3
4
5
6
7
8
9
10
11
12
13
14
15
16
17
18
19
20
21
22
23
24
25
26
27
28
29
30
31
32
33
34
35
36
37
38
39
40
41
42
43
44
45
46
47
48
49
50
51
52
53
54
55
56
57
58
59
60
500 µV
-8
-6
-4
-2
0
2
time (s)

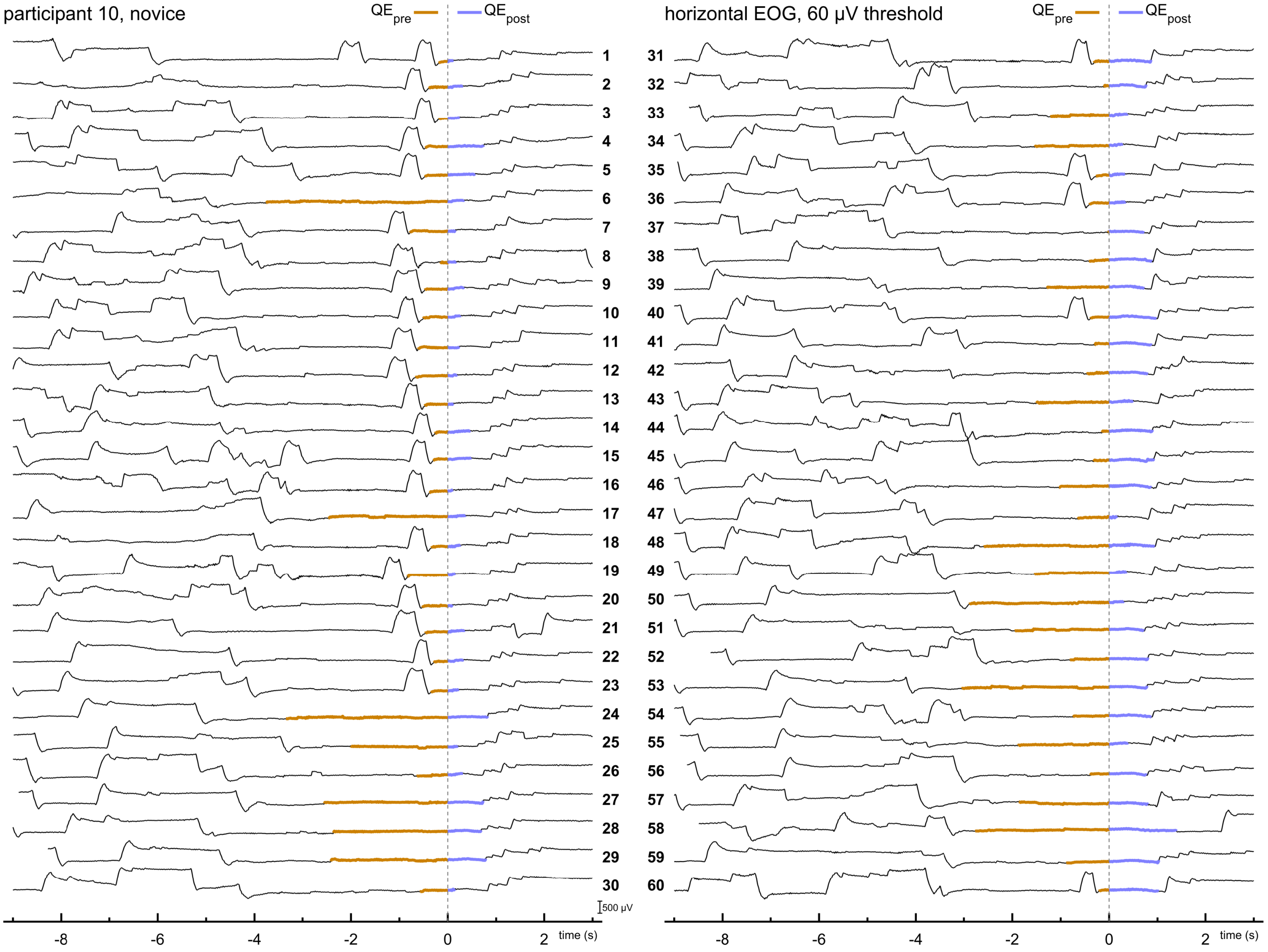
participant 10, novice
horizontal EOG, 60 µV threshold
QE$_{pre}$
QE$_{post}$
500 µV
time (s)
-8
-6
-4
-2
0
2
1 2 3 4 5 6 7 8 9 10 11 12 13 14 15 16 17 18 19 20 21 22 23 24 25 26 27 28 29 30
31 32 33 34 35 36 37 38 39 40 41 42 43 44 45 46 47 48 49 50 51 52 53 54 55 56 57 58 59 60

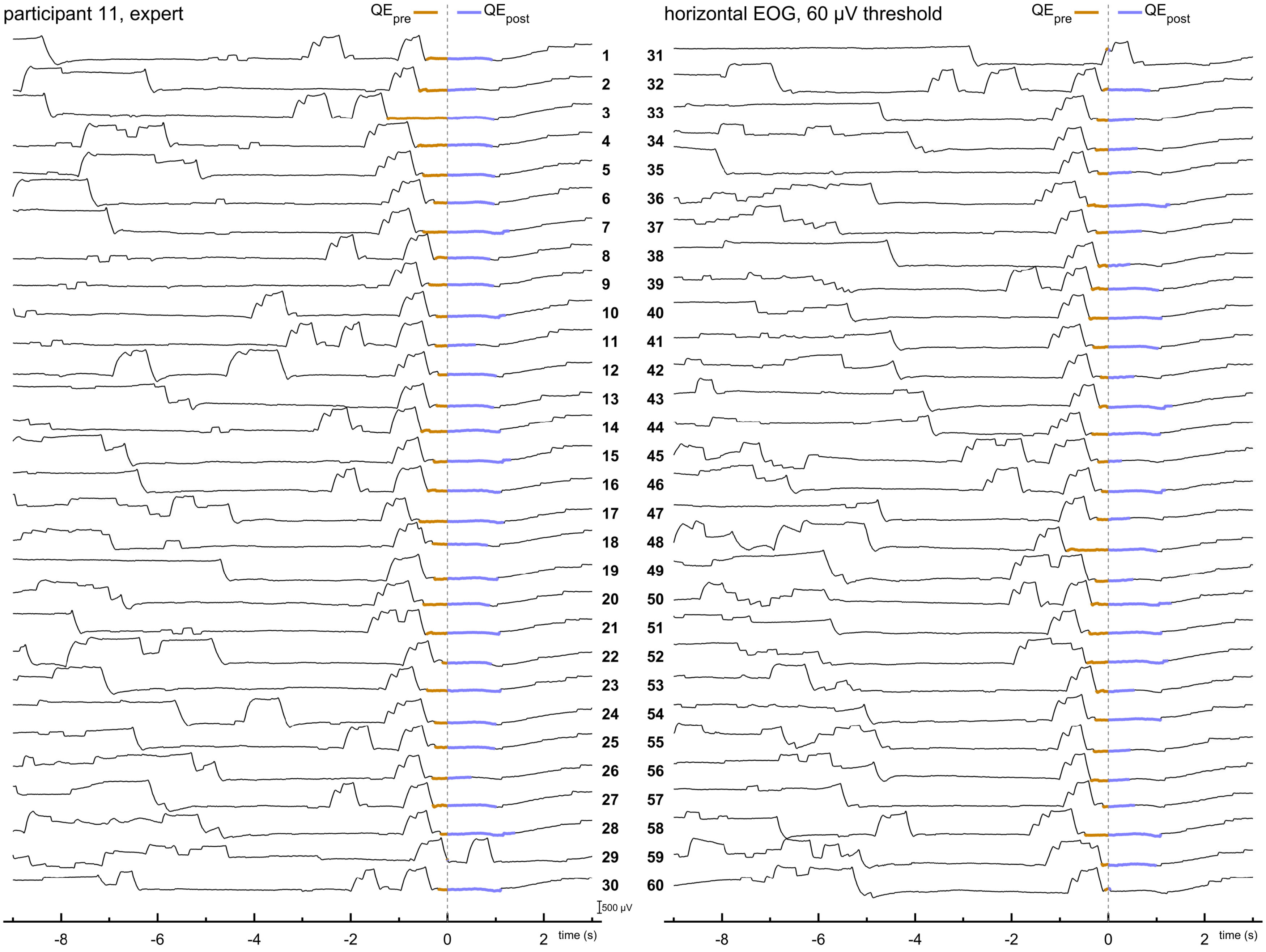

participant 11, expert
horizontal EOG, 60 μV threshold
QE_pre
QE_post
500 μV
time (s)
-8
-6
-4
-2
0
2

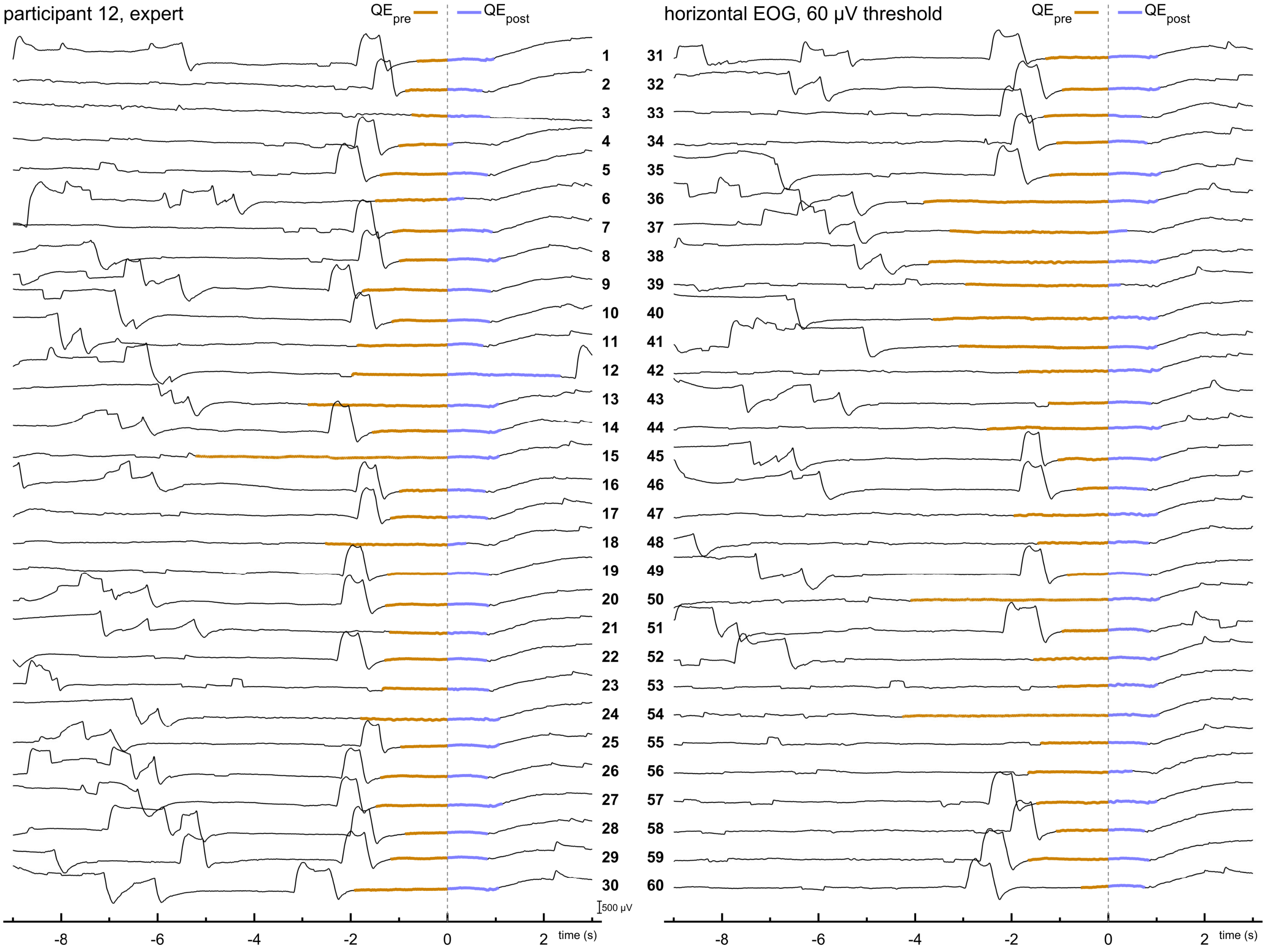
participant 12, expert
horizontal EOG, 60 μV threshold
QEpre
QEpost
500 μV
time (s)
-8
-6
-4
-2
0
2

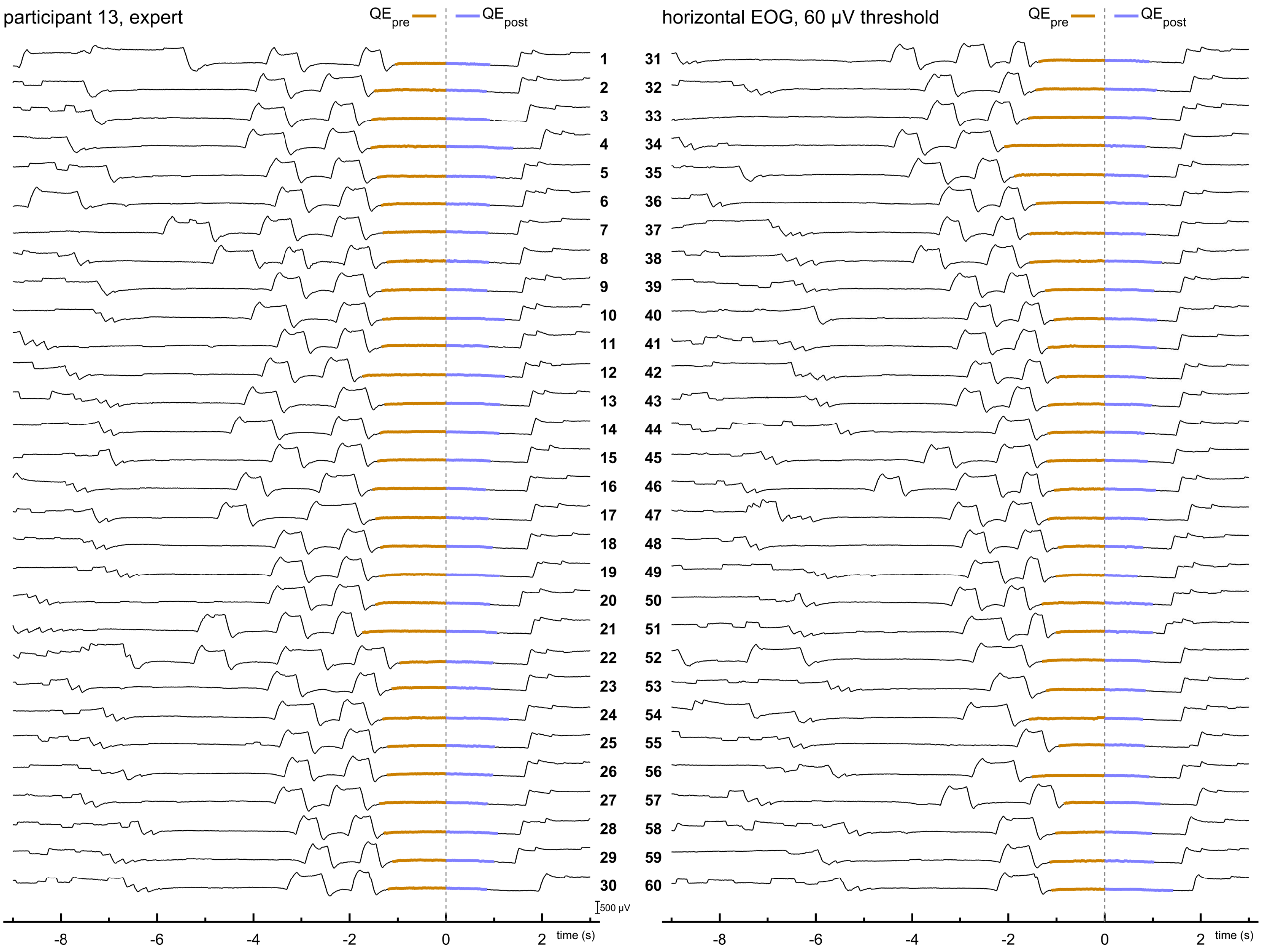
participant 13, expert
horizontal EOG, 60 µV threshold
QEpre
QEpost
1 2 3 4 5 6 7 8 9 10 11 12 13 14 15 16 17 18 19 20 21 22 23 24 25 26 27 28 29 30
31 32 33 34 35 36 37 38 39 40 41 42 43 44 45 46 47 48 49 50 51 52 53 54 55 56 57 58 59 60
500 µV
-8 -6 -4 -2 0 2
time (s)

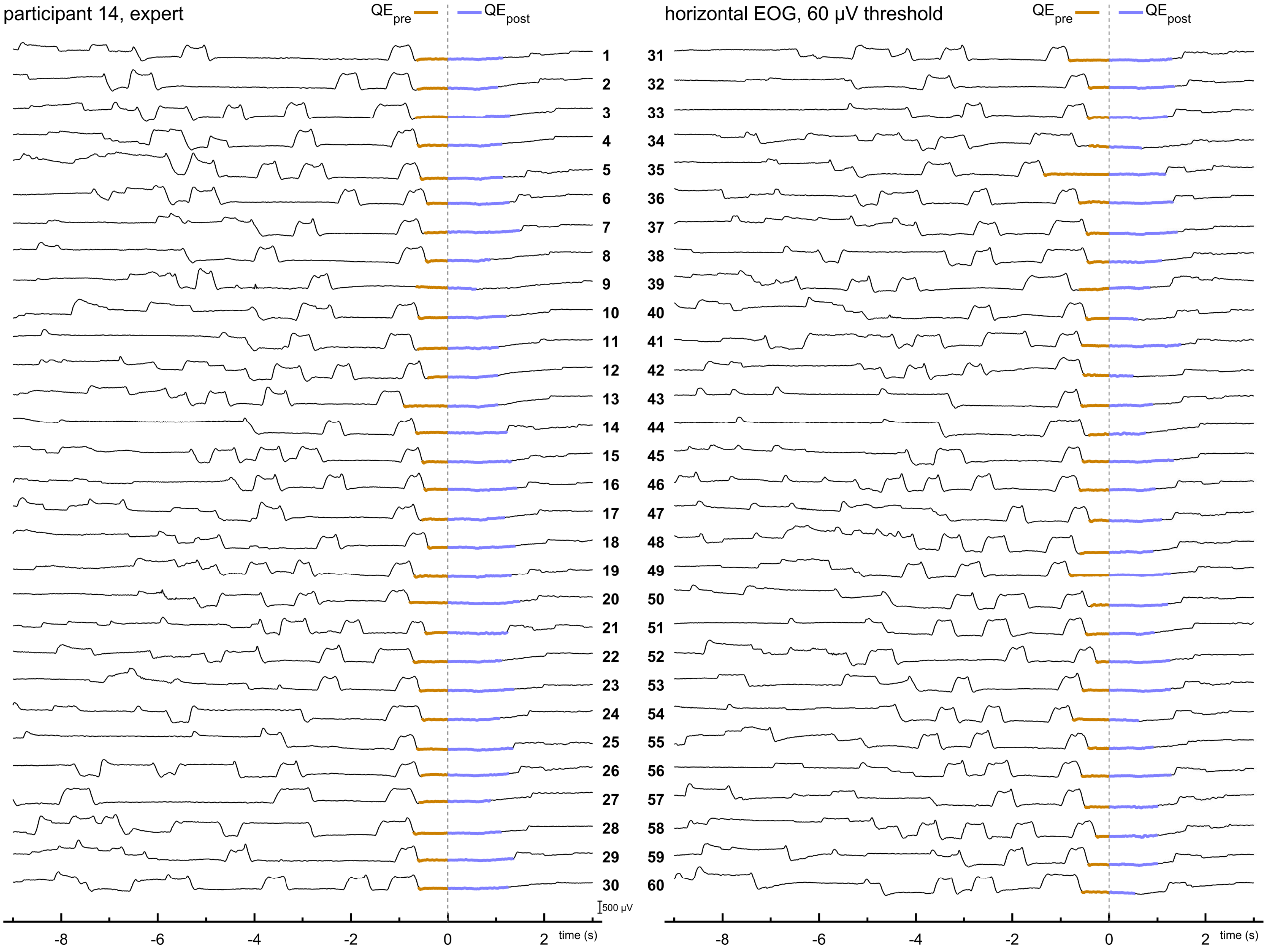

participant 14, expert
horizontal EOG, 60 µV threshold
QE pre
QE post
500 µV
time (s)
-8
-6
-4
-2
0
2

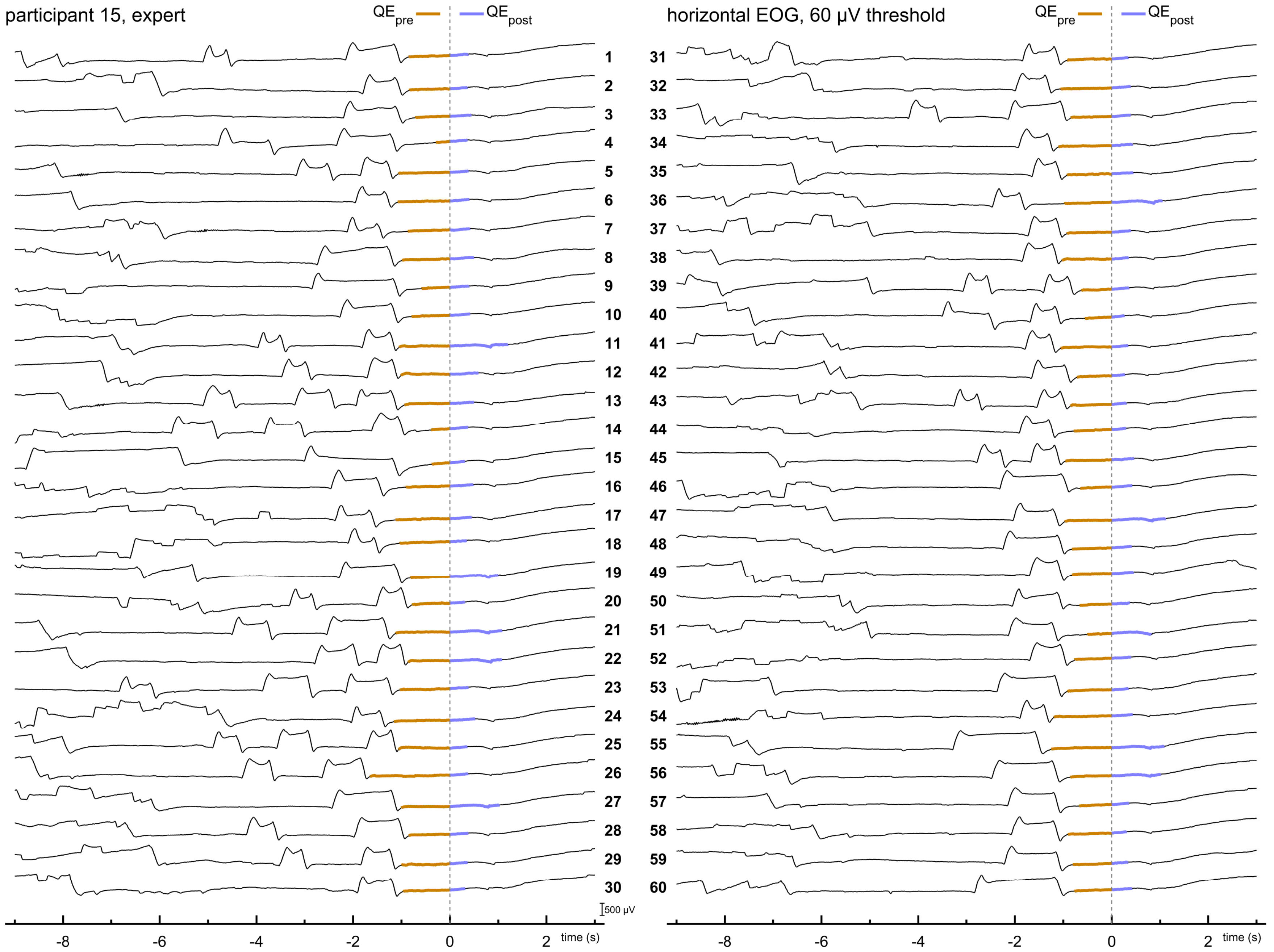
participant 15, expert
QEpre
QEpost
horizontal EOG, 60 μV threshold
QEpre
QEpost
1
2
3
4
5
6
7
8
9
10
11
12
13
14
15
16
17
18
19
20
21
22
23
24
25
26
27
28
29
30
31
32
33
34
35
36
37
38
39
40
41
42
43
44
45
46
47
48
49
50
51
52
53
54
55
56
57
58
59
60
500 μV
-8
-6
-4
-2
0
2
time (s)
-8
-6
-4
-2
0
2
time (s)

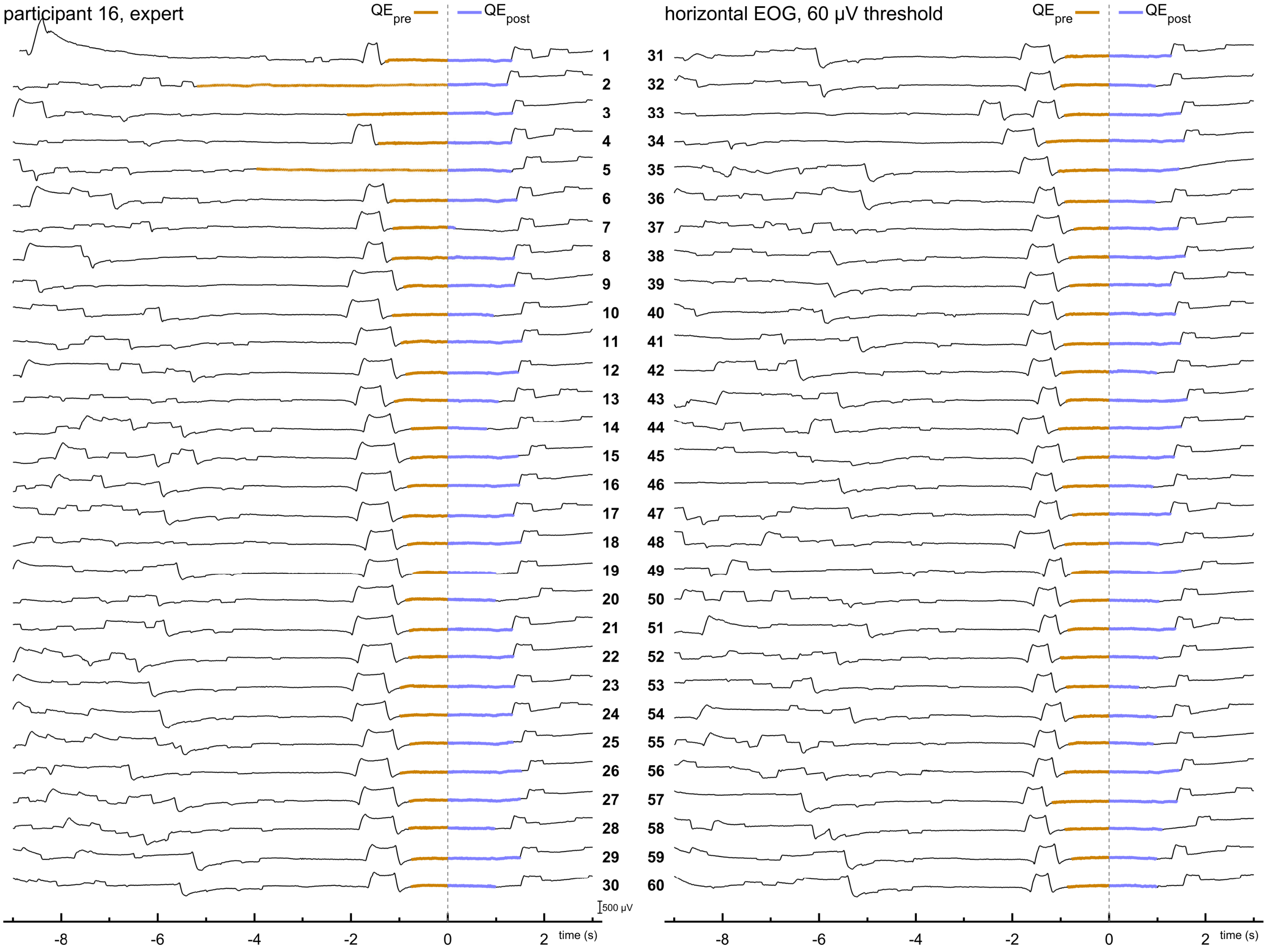

participant 16, expert
horizontal EOG, 60 µV threshold
QE pre
QE post
500 µV
time (s)
-8
-6
-4
-2
0
2
1 2 3 4 5 6 7 8 9 10 11 12 13 14 15 16 17 18 19 20 21 22 23 24 25 26 27 28 29 30
31 32 33 34 35 36 37 38 39 40 41 42 43 44 45 46 47 48 49 50 51 52 53 54 55 56 57 58 59 60

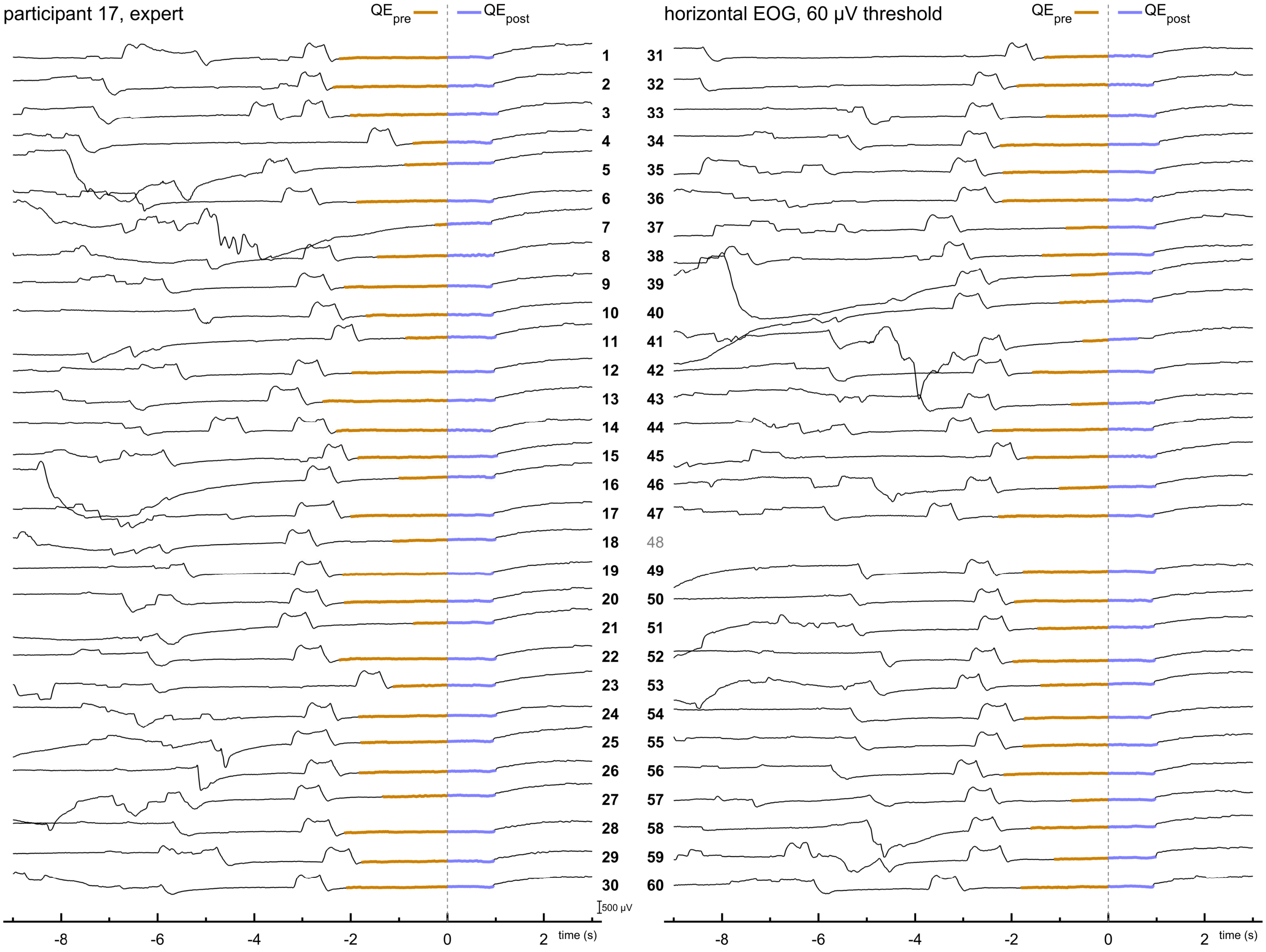
participant 17, expert
horizontal EOG, 60 µV threshold
QE pre
QE post
1
2
3
4
5
6
7
8
9
10
11
12
13
14
15
16
17
18
19
20
21
22
23
24
25
26
27
28
29
30
31
32
33
34
35
36
37
38
39
40
41
42
43
44
45
46
47
48
49
50
51
52
53
54
55
56
57
58
59
60
500 µV
-8
-6
-4
-2
0
2
time (s)

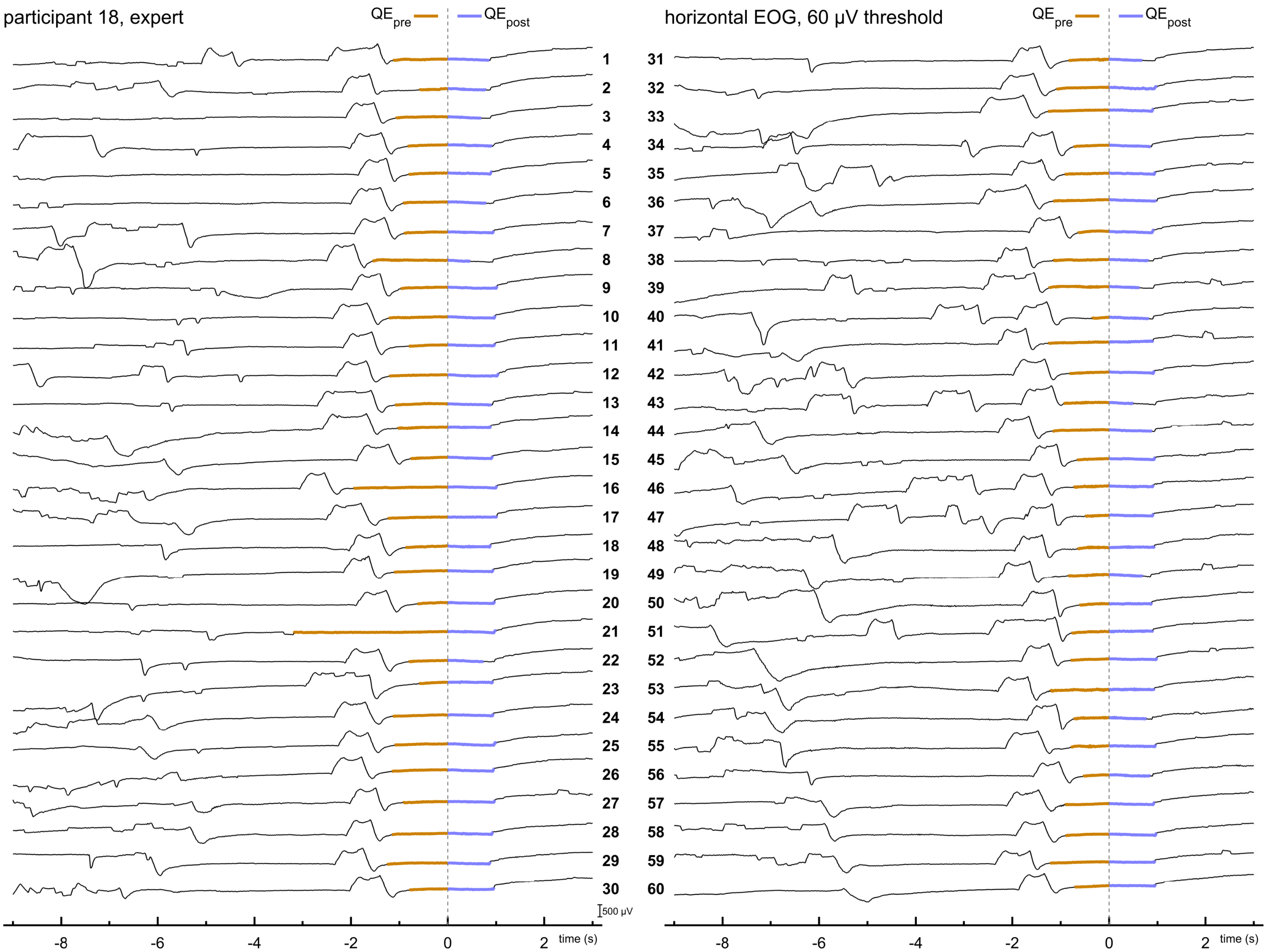
participant 18, expert
QEpre
QEpost
horizontal EOG, 60 µV threshold
1
2
3
4
5
6
7
8
9
10
11
12
13
14
15
16
17
18
19
20
21
22
23
24
25
26
27
28
29
30
31
32
33
34
35
36
37
38
39
40
41
42
43
44
45
46
47
48
49
50
51
52
53
54
55
56
57
58
59
60
500 µV
-8
-6
-4
-2
0
2
time (s)

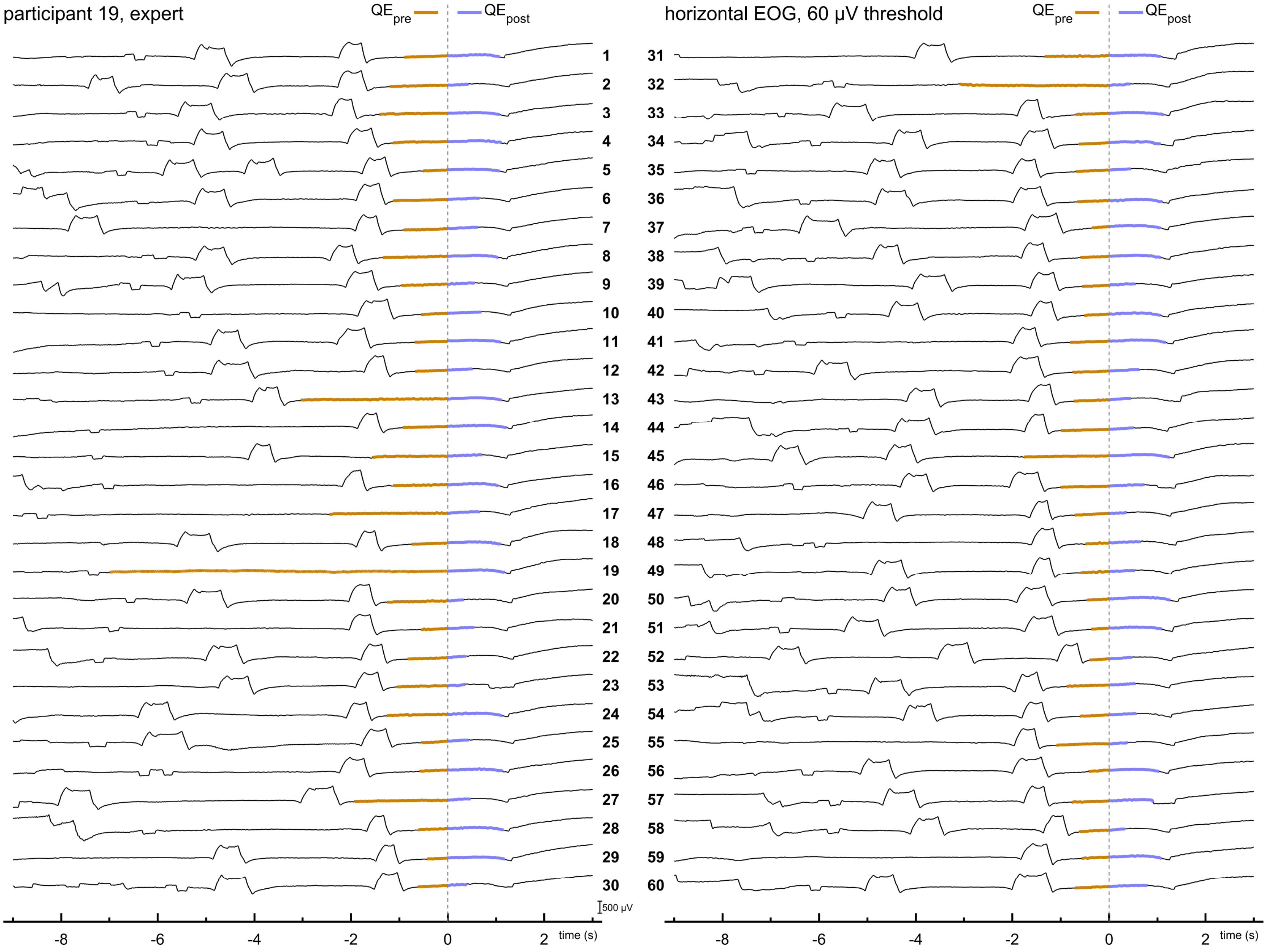

participant 19, expert
horizontal EOG, 60 μV threshold
QEpre
QEpost
500 μV
-8
-6
-4
-2
0
2
time (s)

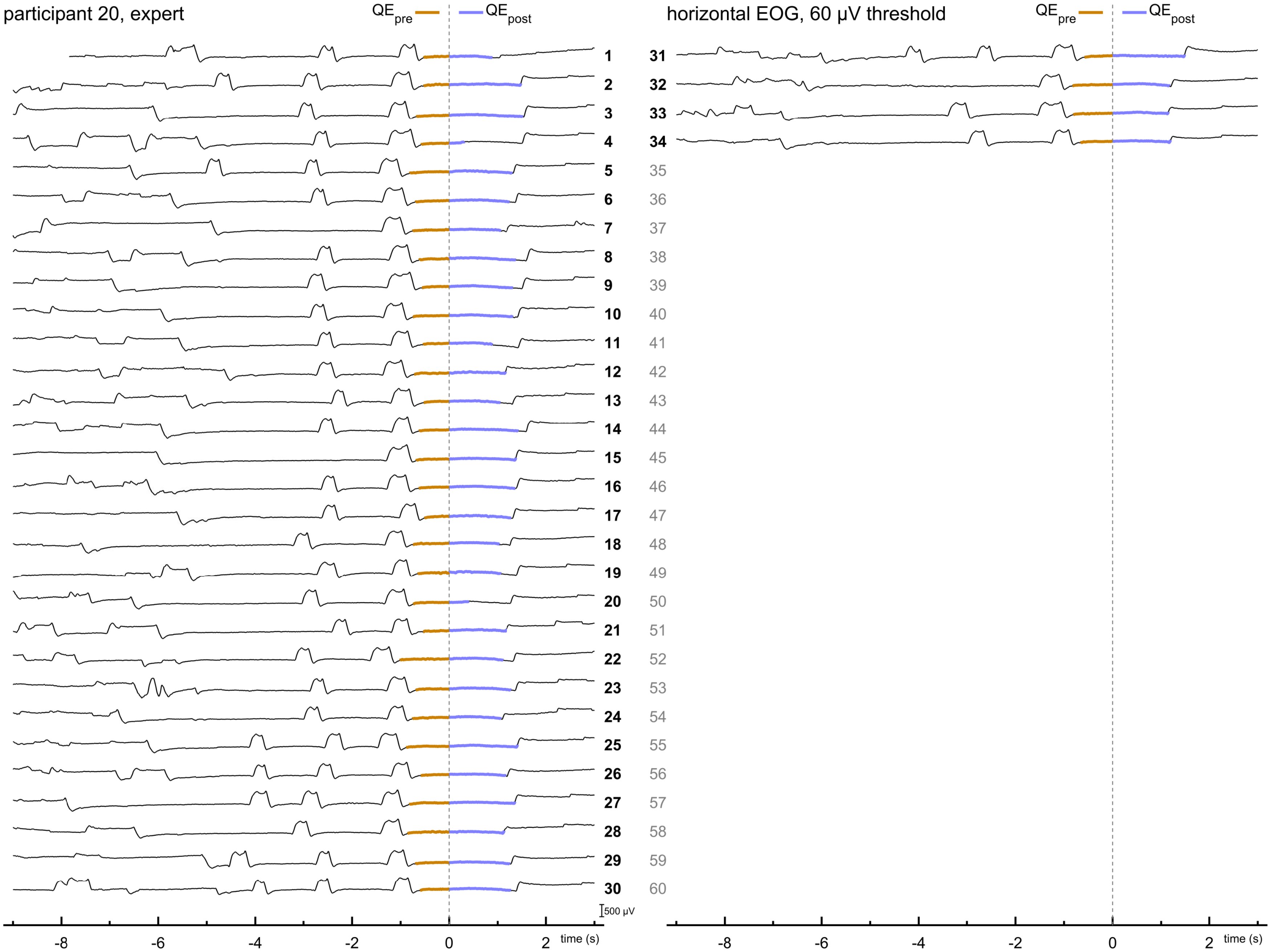

participant 20, expert
QE pre
QE post
horizontal EOG, 60 µV threshold
1
2
3
4
5
6
7
8
9
10
11
12
13
14
15
16
17
18
19
20
21
22
23
24
25
26
27
28
29
30
31
32
33
34
35
36
37
38
39
40
41
42
43
44
45
46
47
48
49
50
51
52
53
54
55
56
57
58
59
60
500 µV
-8
-6
-4
-2
0
2
time (s)

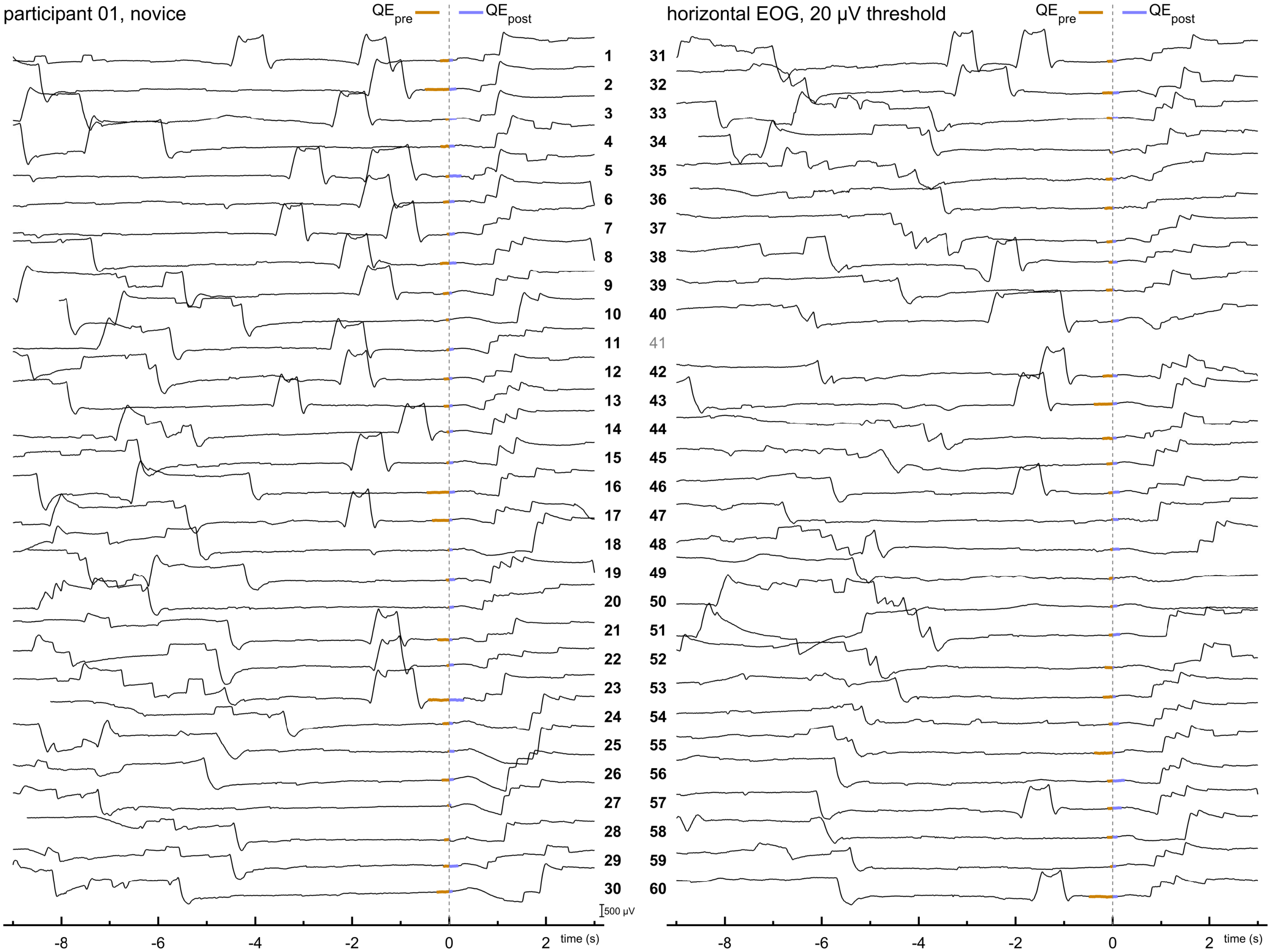

participant 01, novice
horizontal EOG, 20 μV threshold
QE pre
QE post
500 μV
time (s)
-8
-6
-4
-2
0
2

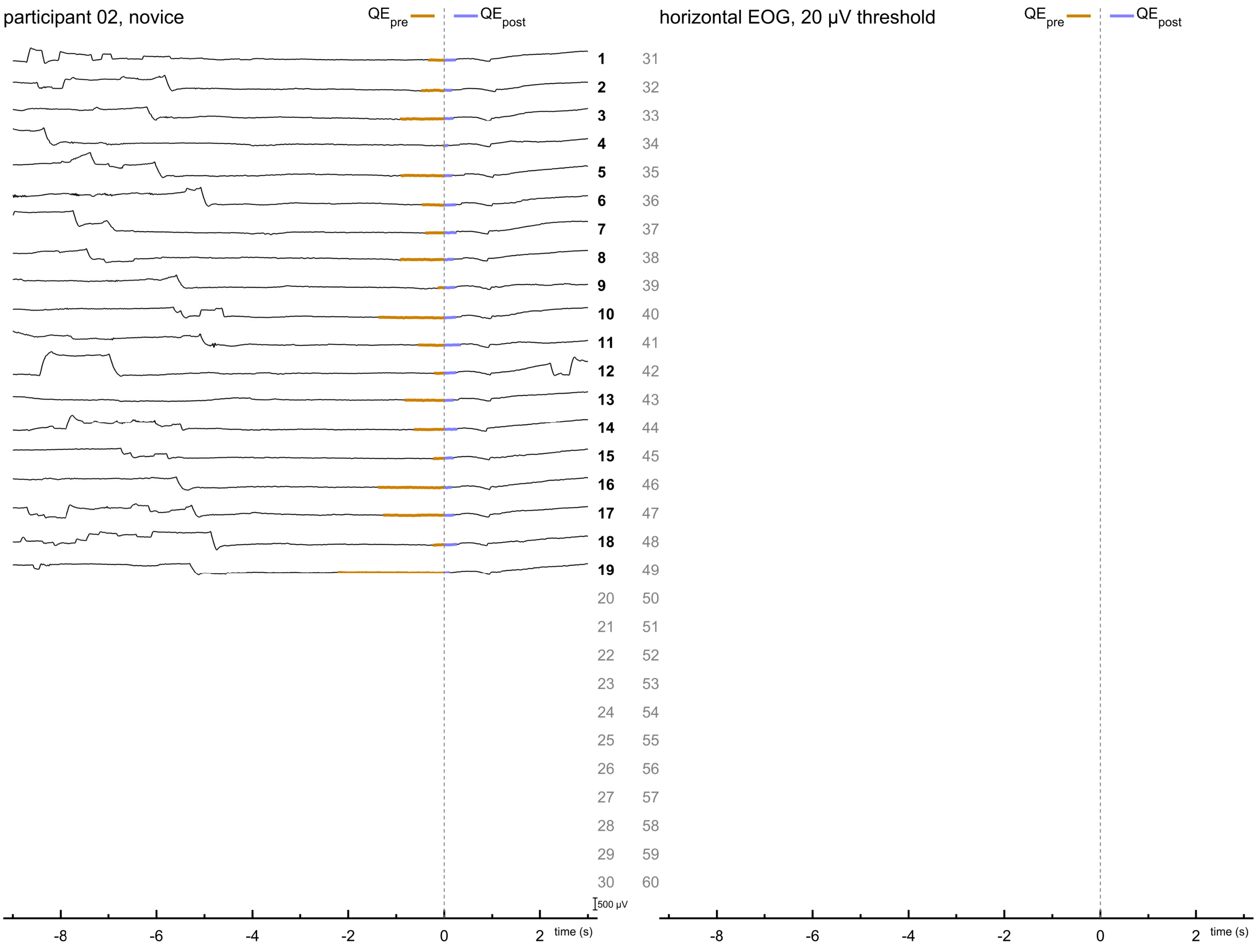
participant 02, novice
horizontal EOG, 20 μV threshold
QEpre
QEpost
1 2 3 4 5 6 7 8 9 10 11 12 13 14 15 16 17 18 19 20 21 22 23 24 25 26 27 28 29 30
31 32 33 34 35 36 37 38 39 40 41 42 43 44 45 46 47 48 49 50 51 52 53 54 55 56 57 58 59 60
500 μV
-8 -6 -4 -2 0 2
time (s)

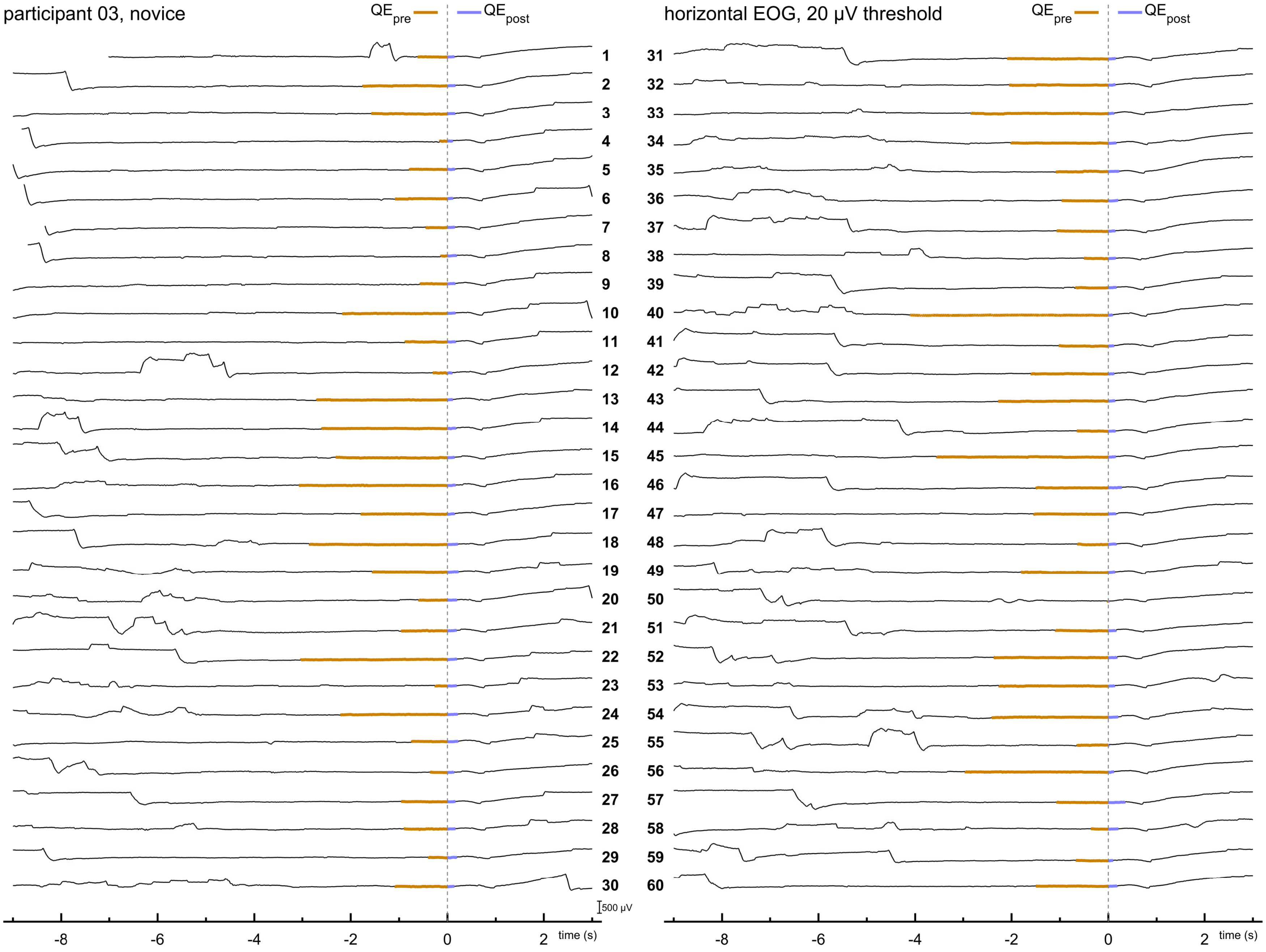
participant 03, novice
QE$_{pre}$
QE$_{post}$
horizontal EOG, 20 µV threshold
1 2 3 4 5 6 7 8 9 10 11 12 13 14 15 16 17 18 19 20 21 22 23 24 25 26 27 28 29 30
31 32 33 34 35 36 37 38 39 40 41 42 43 44 45 46 47 48 49 50 51 52 53 54 55 56 57 58 59 60
500 µV
-8 -6 -4 -2 0 2
time (s)

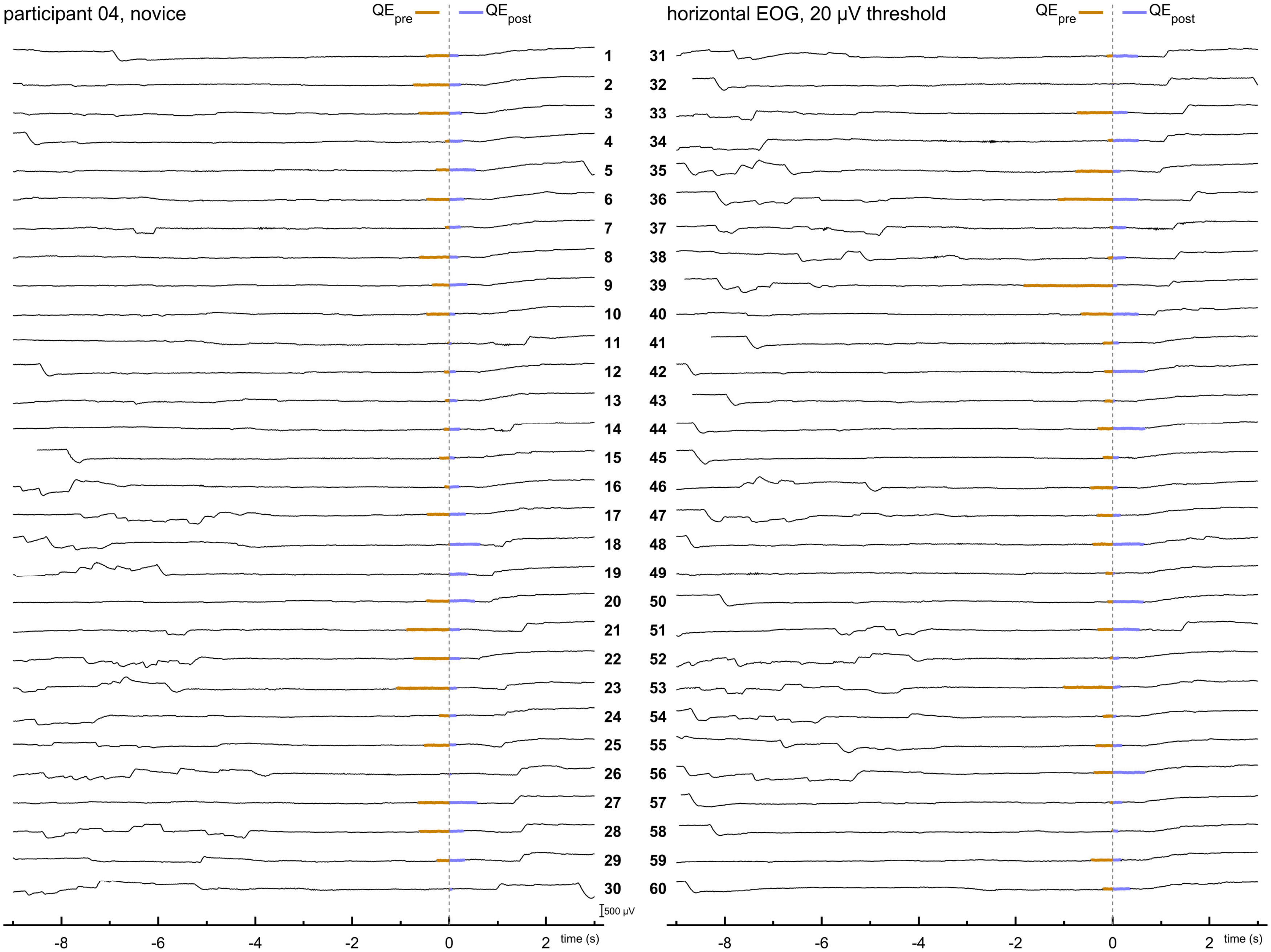
participant 04, novice
horizontal EOG, 20 µV threshold
QEpre
QEpost
500 µV
time (s)
-8
-6
-4
-2
0
2
1 2 3 4 5 6 7 8 9 10 11 12 13 14 15 16 17 18 19 20 21 22 23 24 25 26 27 28 29 30
31 32 33 34 35 36 37 38 39 40 41 42 43 44 45 46 47 48 49 50 51 52 53 54 55 56 57 58 59 60

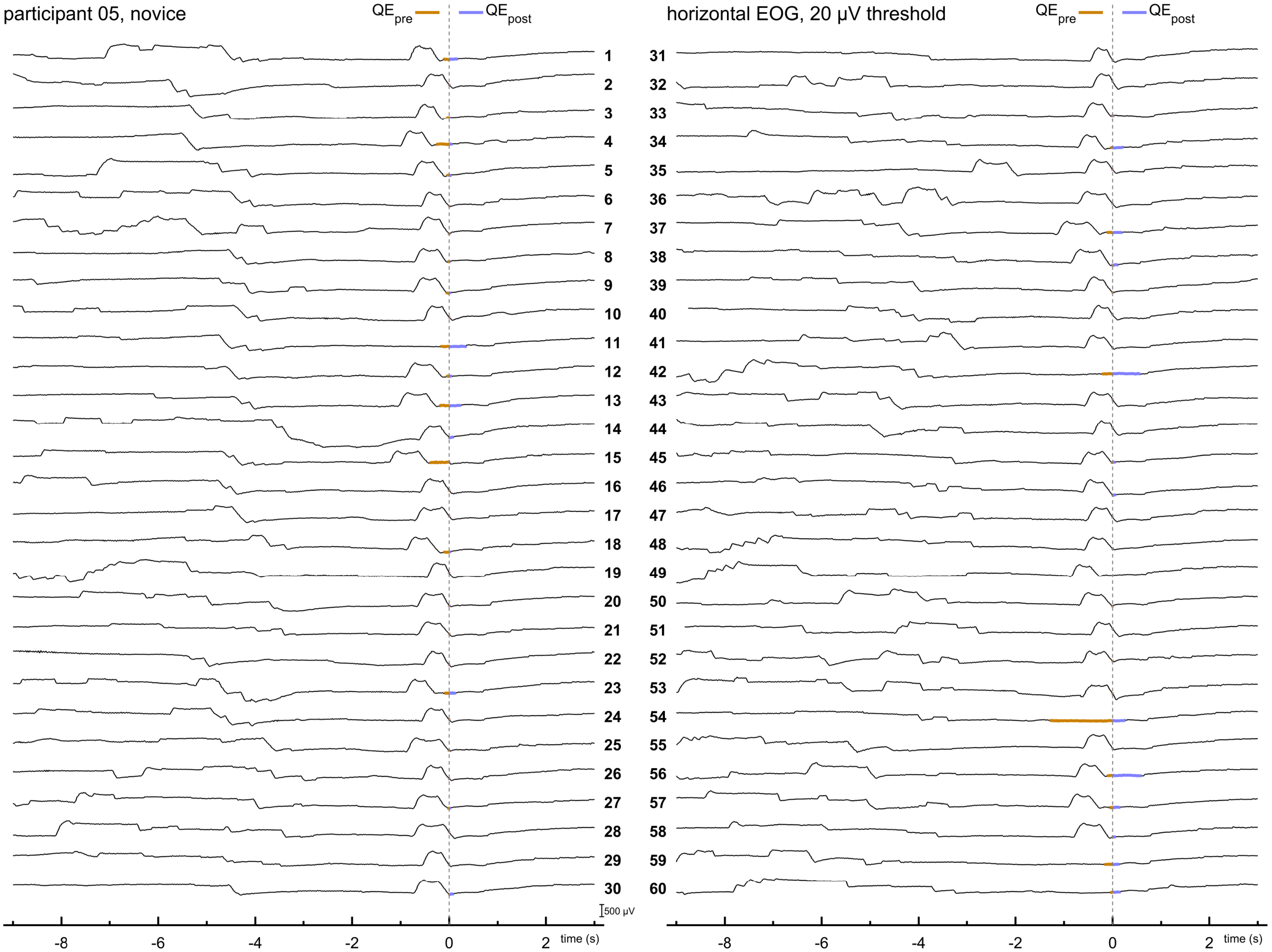

participant 05, novice
horizontal EOG, 20 µV threshold
QEpre
QEpost
1 2 3 4 5 6 7 8 9 10 11 12 13 14 15 16 17 18 19 20 21 22 23 24 25 26 27 28 29 30
31 32 33 34 35 36 37 38 39 40 41 42 43 44 45 46 47 48 49 50 51 52 53 54 55 56 57 58 59 60
500 µV
-8 -6 -4 -2 0 2
time (s)

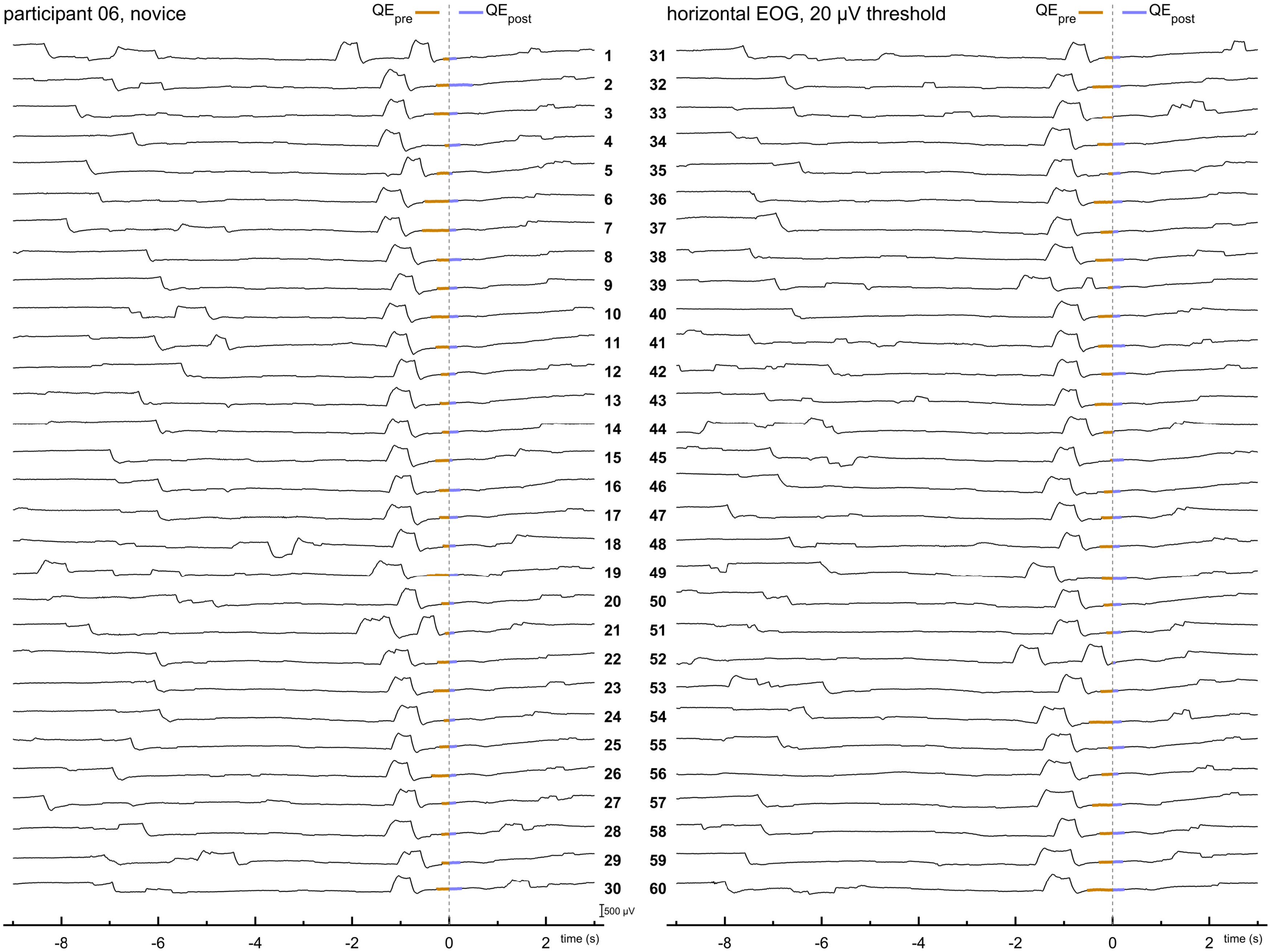

participant 06, novice
horizontal EOG, 20 µV threshold
QEpre
QEpost
500 µV
time (s)
-8
-6
-4
-2
0
2

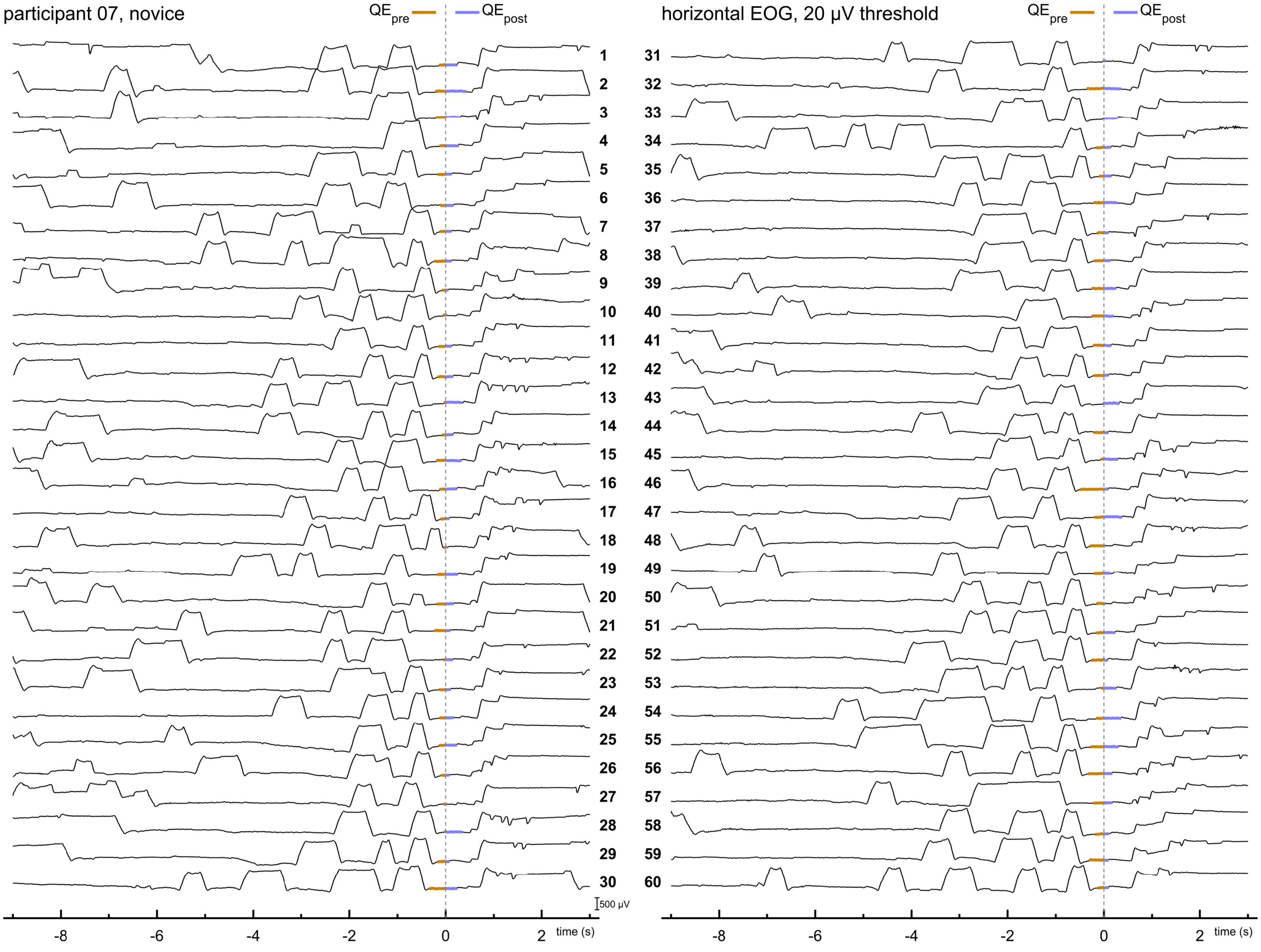

participant 07, novice
horizontal EOG, 20 μV threshold
QEpre
QEpost
500 μV
time (s)
-8
-6
-4
-2
0
2
1 2 3 4 5 6 7 8 9 10 11 12 13 14 15 16 17 18 19 20 21 22 23 24 25 26 27 28 29 30
31 32 33 34 35 36 37 38 39 40 41 42 43 44 45 46 47 48 49 50 51 52 53 54 55 56 57 58 59 60

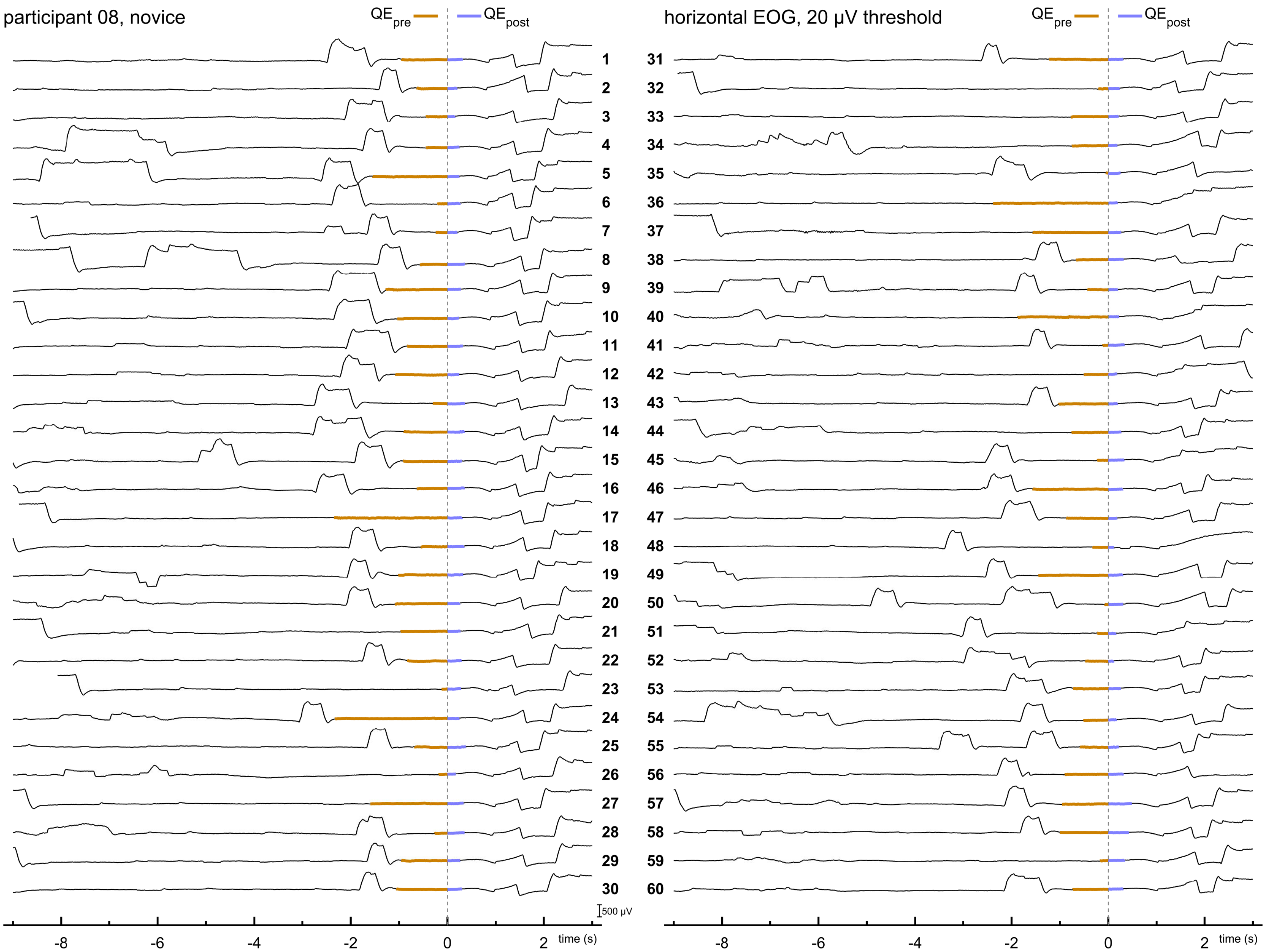

participant 08, novice
horizontal EOG, 20 μV threshold
QE pre
QE post
1 2 3 4 5 6 7 8 9 10 11 12 13 14 15 16 17 18 19 20 21 22 23 24 25 26 27 28 29 30
31 32 33 34 35 36 37 38 39 40 41 42 43 44 45 46 47 48 49 50 51 52 53 54 55 56 57 58 59 60
500 μV
-8 -6 -4 -2 0 2
time (s)

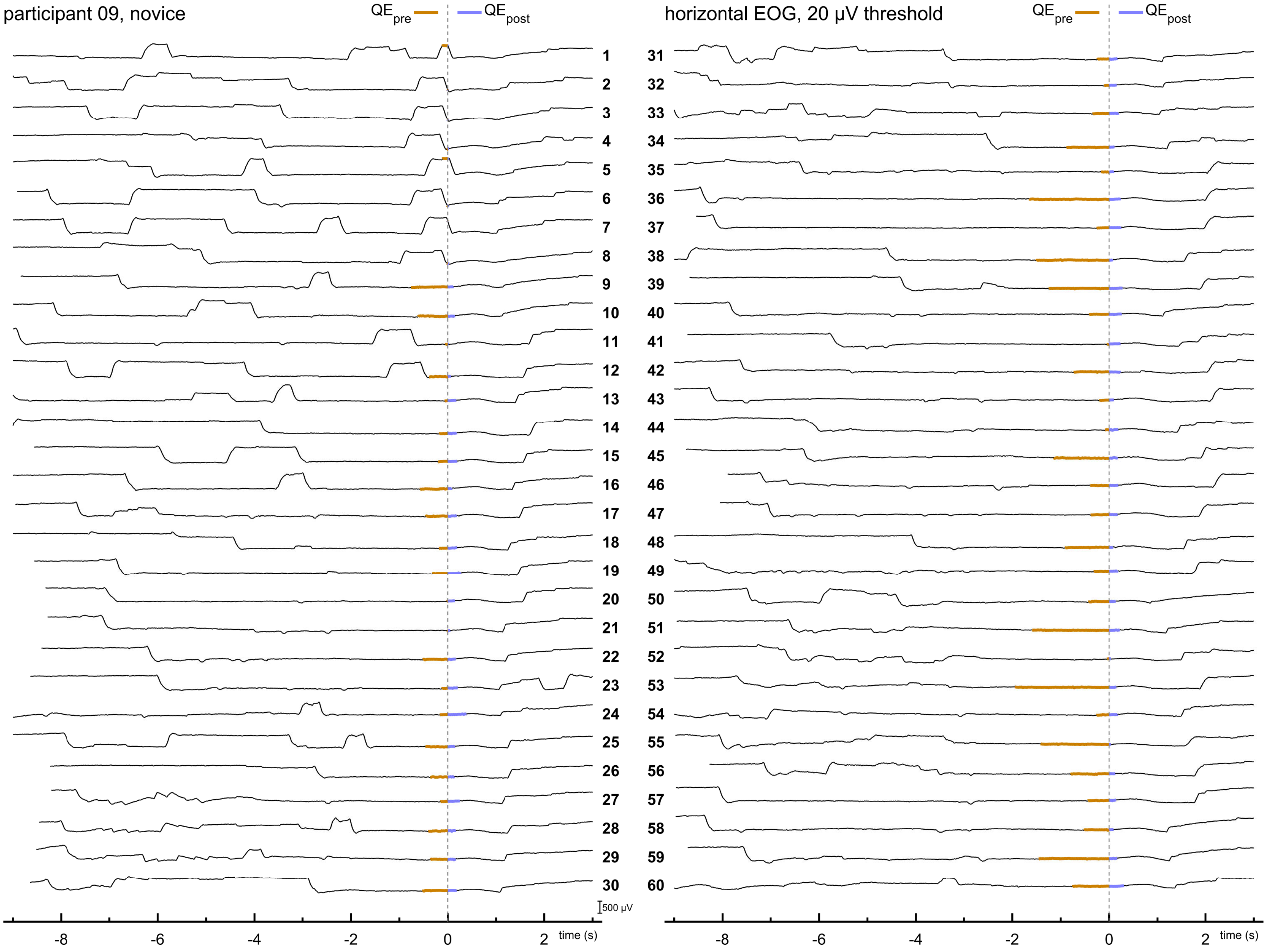

participant 09, novice
horizontal EOG, 20 µV threshold
QE pre
QE post
500 µV
time (s)
-8
-6
-4
-2
0
2

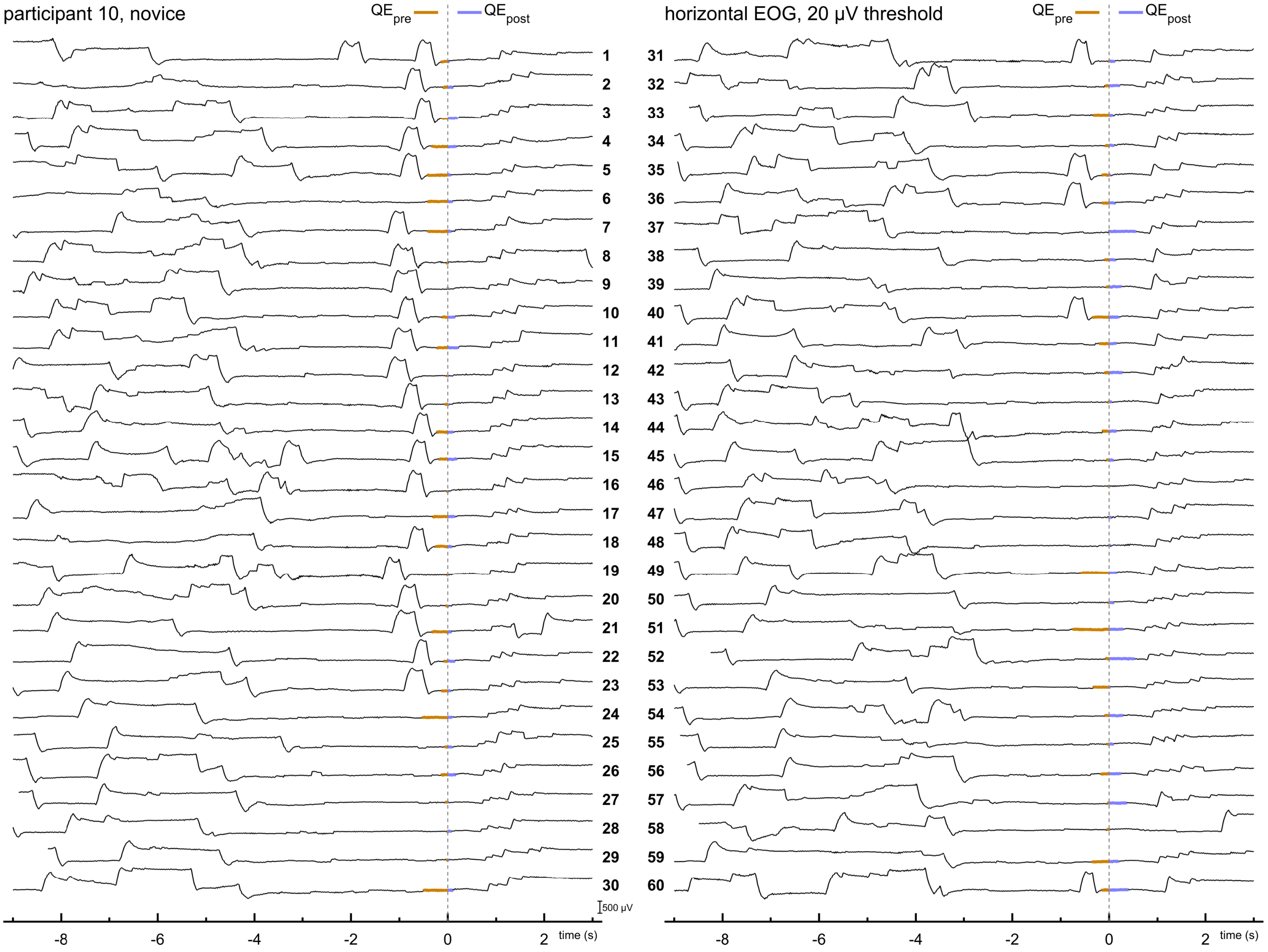

participant 10, novice
horizontal EOG, 20 µV threshold
QEpre
QEpost
500 µV
time (s)
-8
-6
-4
-2
0
2

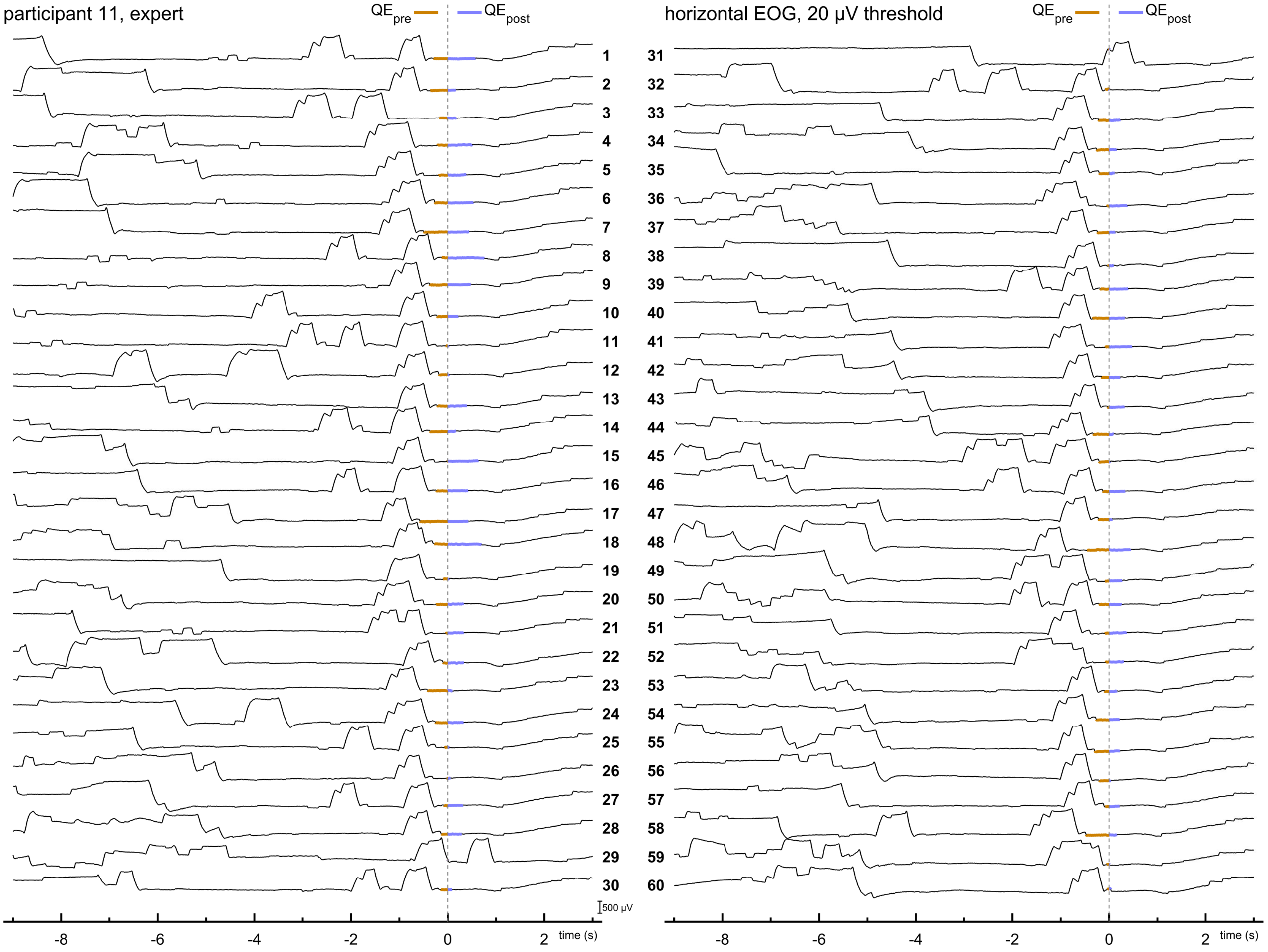

participant 11, expert
horizontal EOG, 20 µV threshold
QEpre
QEpost
1 2 3 4 5 6 7 8 9 10 11 12 13 14 15 16 17 18 19 20 21 22 23 24 25 26 27 28 29 30
31 32 33 34 35 36 37 38 39 40 41 42 43 44 45 46 47 48 49 50 51 52 53 54 55 56 57 58 59 60
500 µV
-8 -6 -4 -2 0 2
time (s)

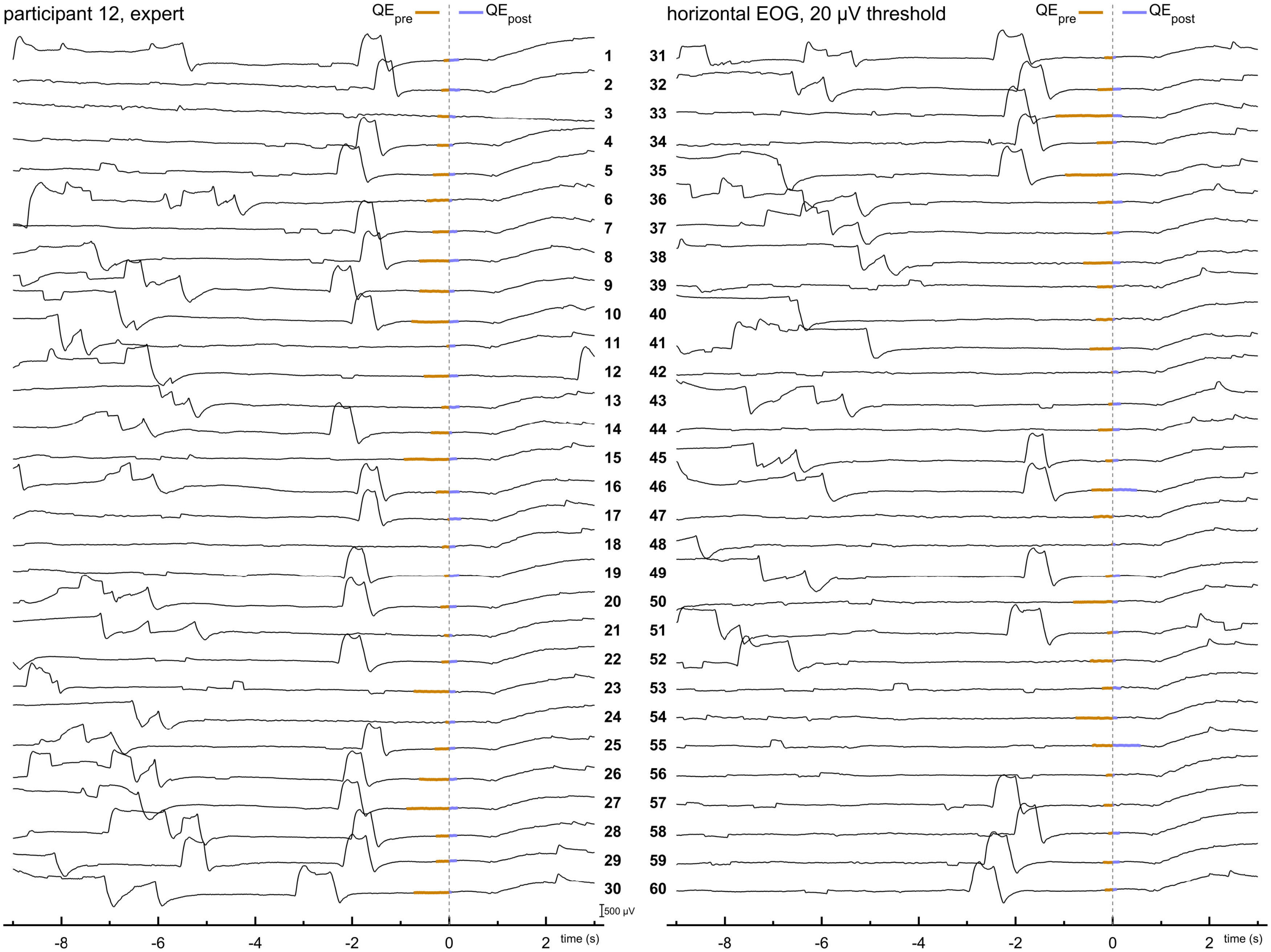
participant 12, expert
horizontal EOG, 20 μV threshold
QEpre
QEpost
500 μV
time (s)
-8
-6
-4
-2
0
2

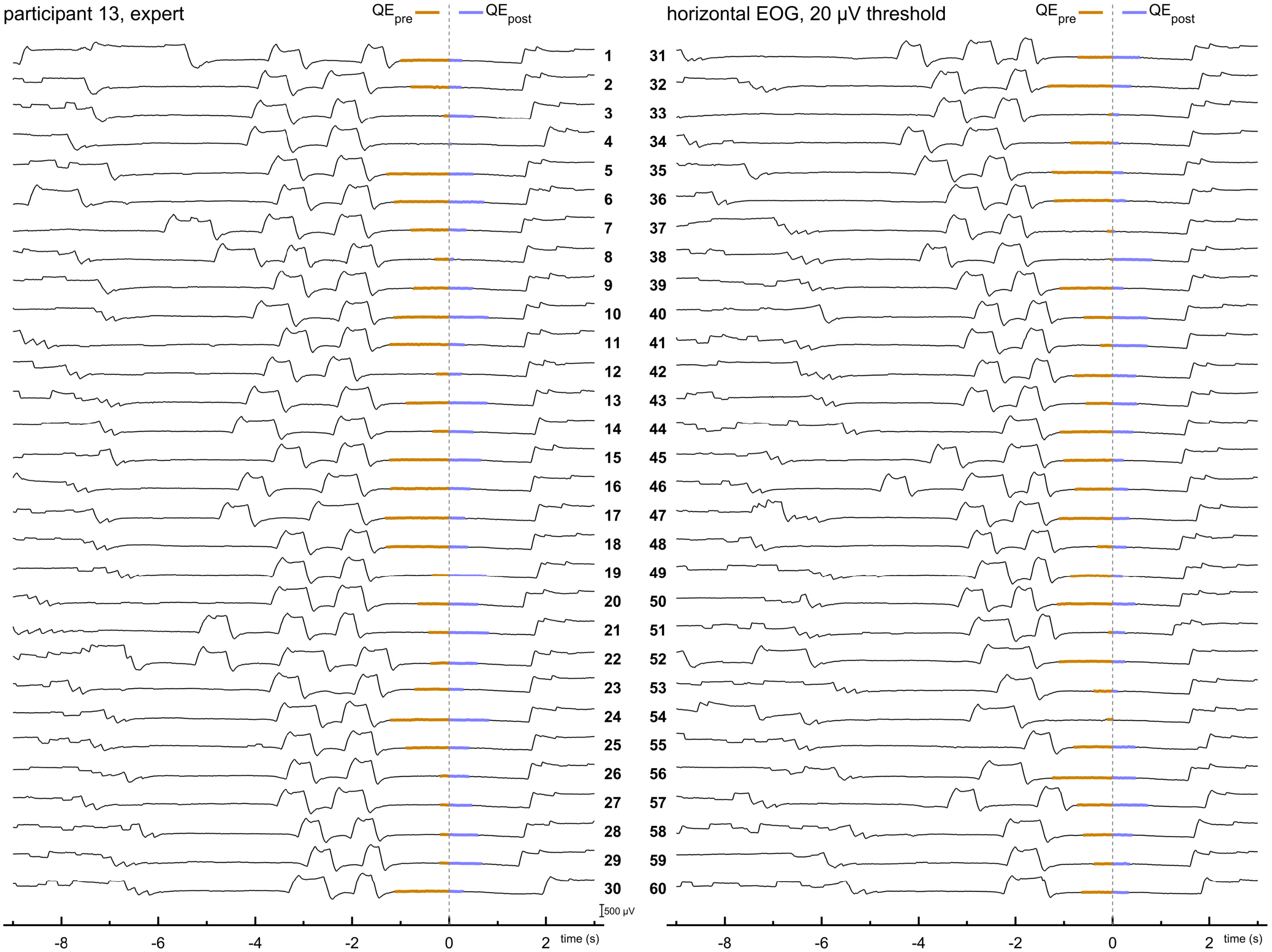
participant 13, expert
horizontal EOG, 20 µV threshold
QEpre
QEpost
500 µV
time (s)
-8
-6
-4
-2
0
2
1 2 3 4 5 6 7 8 9 10 11 12 13 14 15 16 17 18 19 20 21 22 23 24 25 26 27 28 29 30
31 32 33 34 35 36 37 38 39 40 41 42 43 44 45 46 47 48 49 50 51 52 53 54 55 56 57 58 59 60

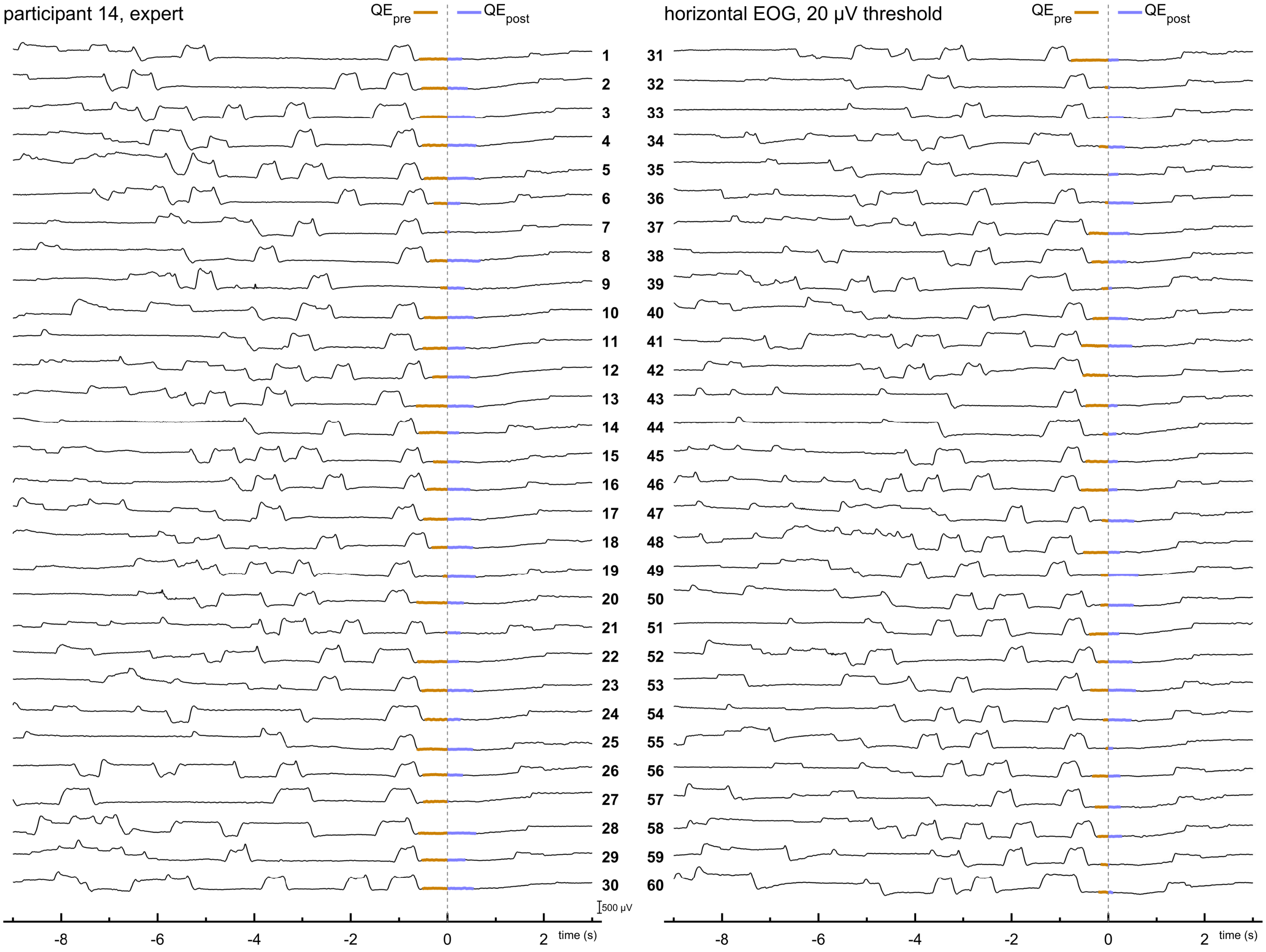
participant 14, expert
horizontal EOG, 20 µV threshold
QE pre
QE post
500 µV
time (s)
-8
-6
-4
-2
0
2
1
2
3
4
5
6
7
8
9
10
11
12
13
14
15
16
17
18
19
20
21
22
23
24
25
26
27
28
29
30
31
32
33
34
35
36
37
38
39
40
41
42
43
44
45
46
47
48
49
50
51
52
53
54
55
56
57
58
59
60

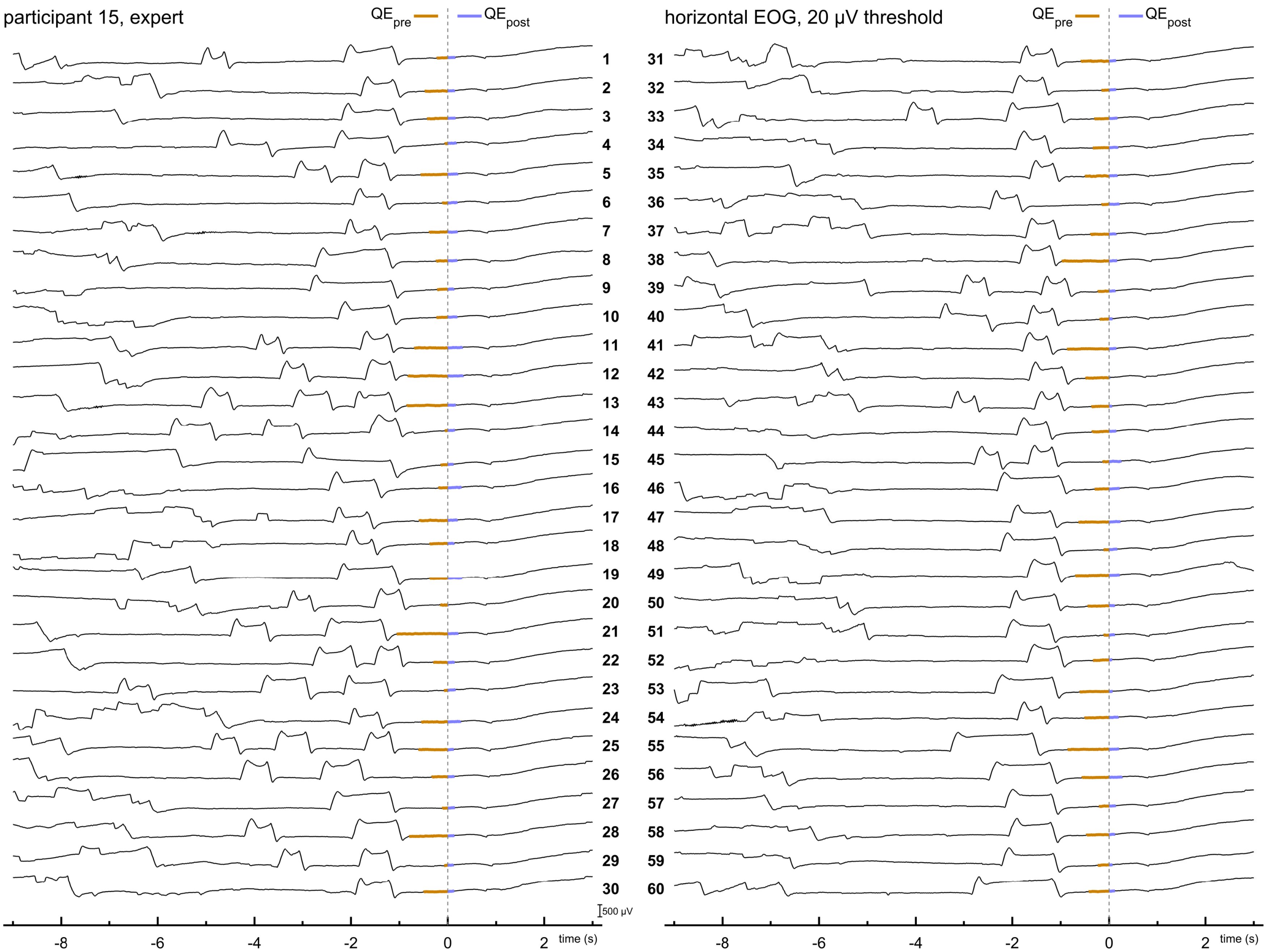
participant 15, expert
horizontal EOG, 20 µV threshold
QEpre
QEpost
500 µV
time (s)
-8
-6
-4
-2
0
2
1
2
3
4
5
6
7
8
9
10
11
12
13
14
15
16
17
18
19
20
21
22
23
24
25
26
27
28
29
30
31
32
33
34
35
36
37
38
39
40
41
42
43
44
45
46
47
48
49
50
51
52
53
54
55
56
57
58
59
60

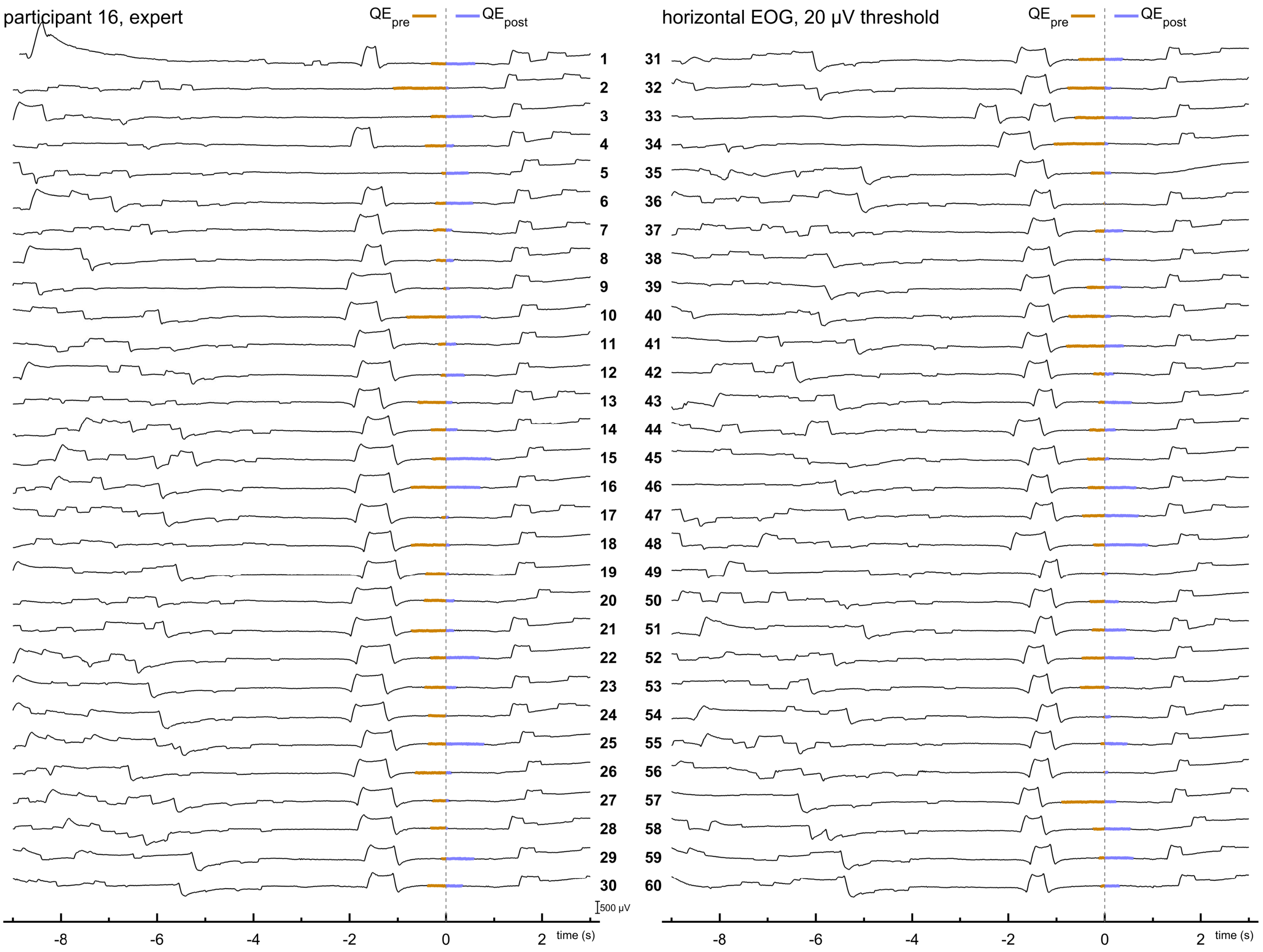

participant 16, expert
horizontal EOG, 20 µV threshold
QEpre
QEpost
500 µV
-8
-6
-4
-2
0
2
time (s)

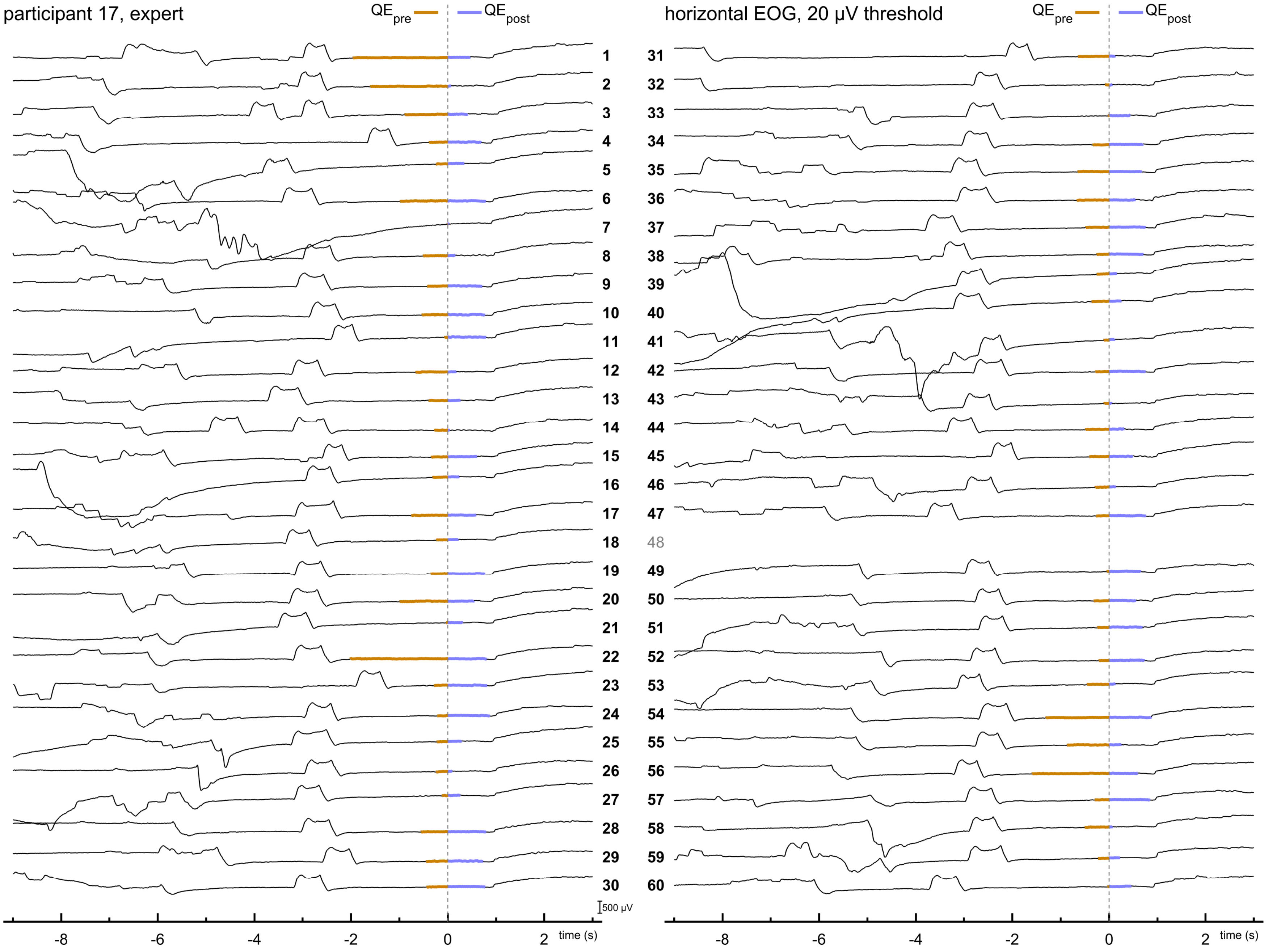

participant 17, expert
horizontal EOG, 20 µV threshold
QEpre
QEpost
500 µV
time (s)
-8
-6
-4
-2
0
2

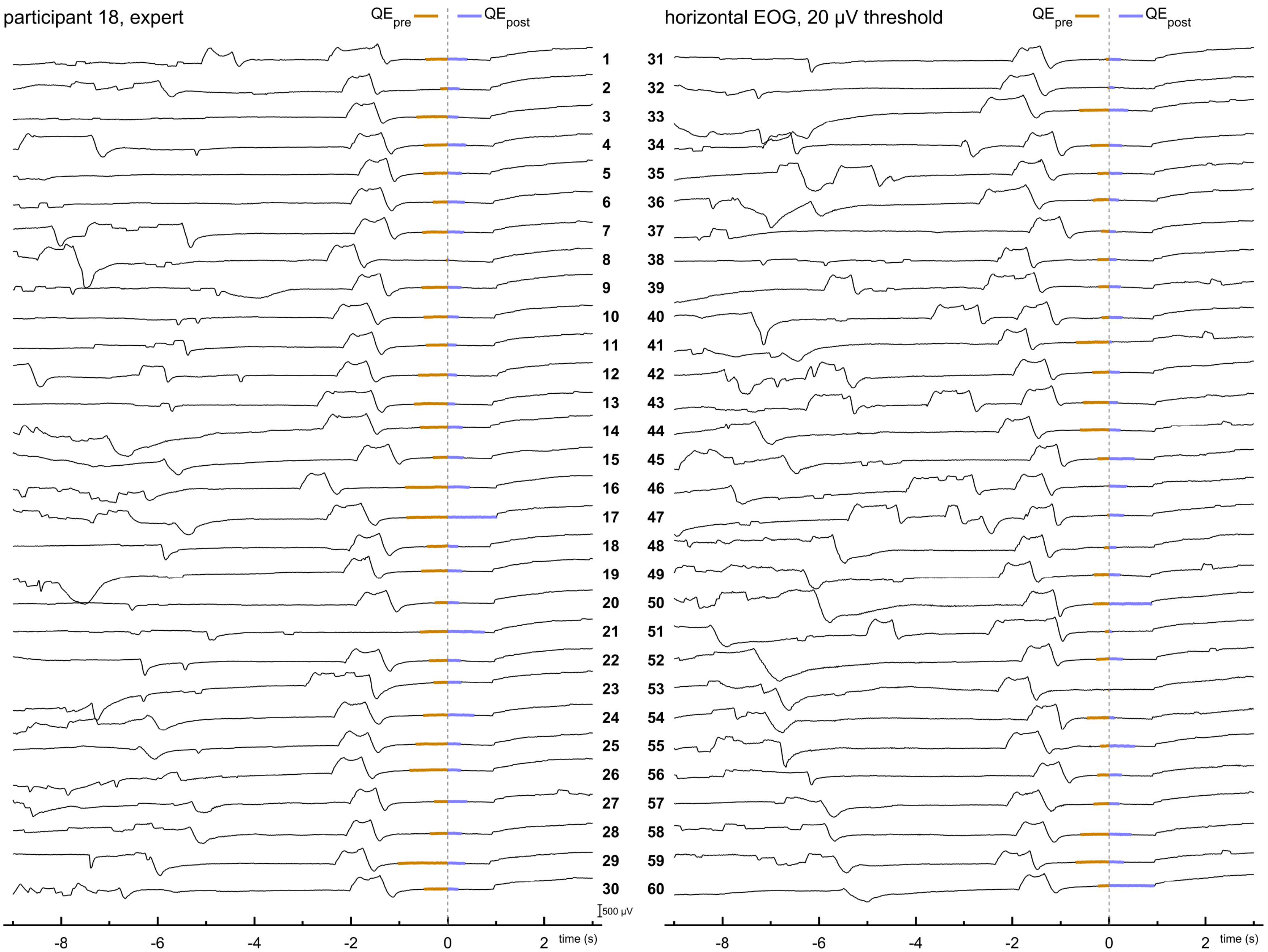
participant 18, expert
horizontal EOG, 20 µV threshold
QEpre
QEpost
1
2
3
4
5
6
7
8
9
10
11
12
13
14
15
16
17
18
19
20
21
22
23
24
25
26
27
28
29
30
31
32
33
34
35
36
37
38
39
40
41
42
43
44
45
46
47
48
49
50
51
52
53
54
55
56
57
58
59
60
500 µV
-8
-6
-4
-2
0
2
time (s)

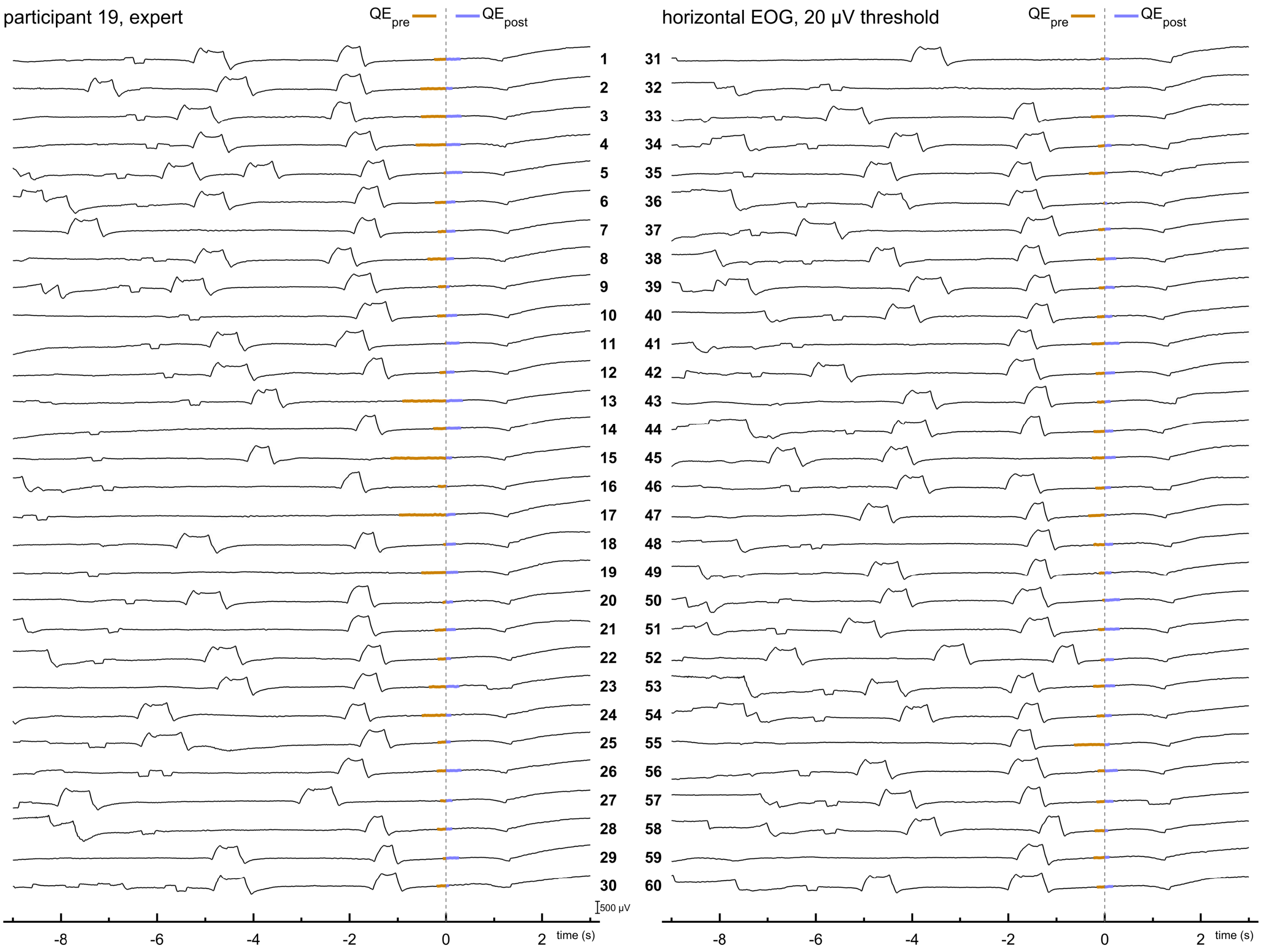

participant 19, expert
horizontal EOG, 20 µV threshold
QEpre
QEpost
500 µV
time (s)
-8
-6
-4
-2
0
2

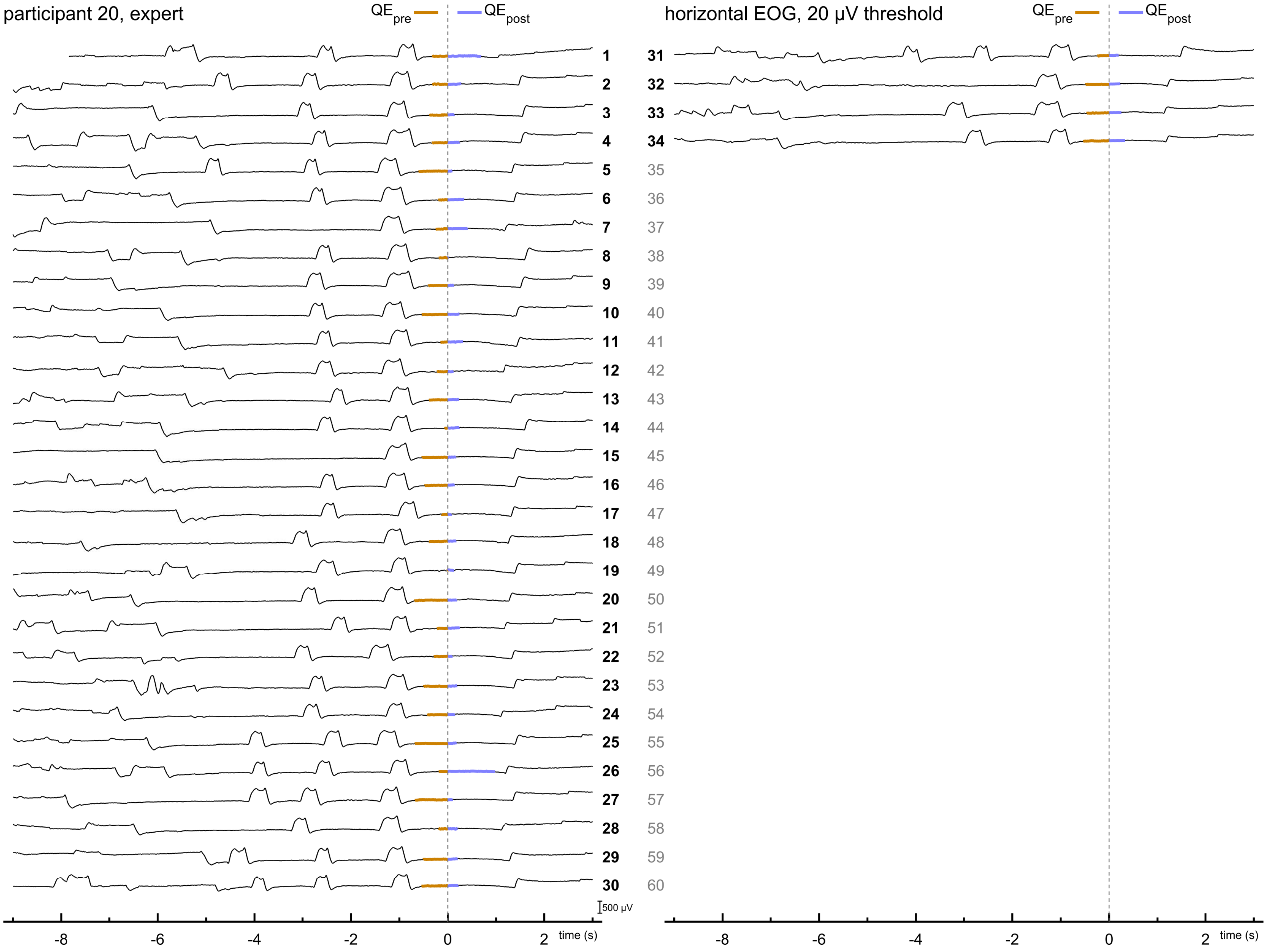
participant 20, expert
QE pre
QE post
horizontal EOG, 20 µV threshold
500 µV
time (s)
-8
-6
-4
-2
0
2
