# Supplementary material for: Assessing ocular activity during performance of motor skills using electrooculography
Source: Psychophysiology. 2018 Feb 9;55(7):e13070. doi: 10.1111/psyp.13070 (PMC6849535; doi:10.1111/psyp.13070)
Supplement: Supplementary file 4 — Appendix S4 [file PSYP-55-na-s004.pdf]

Gallicchio, G., Cooke, A., & Ring, C. Assessing ocular activity during performance of motor skills using electrooculography. *Psychophysiology*
Supplement S4

Pearson's correlations between putting performance (percentage of holed putts) and (i) quiet eye durations (QE_{total}, QE_{pre}, and QE_{post}), computed with 60 and 20 µV threshold levels, (ii) eye quietness (HEOG-SD), computed in different time intervals relative to backswing initiation, and (iii) putting times (address and swing times). Correlations were conducted separately for experts and novices.

Measures	Experts (n = 10)	Novices (n = 10)
QE_{total} (s)		
60 µV	–.17	**–.63***
20 µV	–.24	–.43
QE_{pre} (s)		
60 µV	–.08	**–.63***
20 µV	–.29	–.42
QE_{post} (s)		
60 µV	–.20	–.25
20 µV	–.12	–.26
HEOG-SD (µV)		
–4 to –3.5 s	–.15	.01
–3.5 to –3 s	–.03	.17
–3 to –2.5 s	.37	.30
–2.5 to –2 s	–.05	.24
–2 to –1.5 s	**–.76***	.18
–1.5 to –1 s	–.35	.48
–1 to –0.5 s	.31	.61
–0.5 to 0 s	.18	**.73***
0 to 0.5 s	.23	.19
0.5 to 1 s	.32	.23
1 to 1.5 s	–.11	–.43
1.5 to 2 s	**–.71***	–.25
Putting times (s)		
Address	–.26	–.47
Swing	–.20	–.33

* $p \leq .05$
