# Supplementary material for: Assessing ocular activity during performance of motor skills using electrooculography
Source: Psychophysiology. 2018 Feb 9;55(7):e13070. doi: 10.1111/psyp.13070 (PMC6849535; doi:10.1111/psyp.13070)
Supplement: Supplementary file 5 — Appendix S5 [file PSYP-55-na-s005.pdf]

Gallicchio, G., Cooke, A., & Ring, C. Assessing ocular activity during performance of motor skills using electrooculography. *Psychophysiology*

Supplement S5

Pearson's correlations between putting times (address and swing times) and indices of ocular activity (quiet eye durations and eye quietness). Quiet eye durations (QE_{pre} and QE_{post}) were computed with 60 and 20 µV threshold levels. Eye quietness (HEOG-SD) was computed in different time intervals relative to backswing initiation. The table shows relevant comparisons only, i.e., pre movement initiation ocular activity and address times, and post movement initiation ocular activity and swing times.

	Address times (s)	Swing times (s)
QE_{pre} (s)		
60 µV threshold	.21	-
20 µV threshold	.29	-
QE_{post} (s)		
60 µV threshold	-	**.52***
20 µV threshold	-	.30
HEOG-SD (µV)		
–4 to –3.5 s	.09	-
–3.5 to –3 s	.07	-
–3 to –2.5 s	.03	-
–2.5 to –2 s	.15	-
–2 to –1.5 s	.20	-
–1.5 to –1 s	.06	-
–1 to –0.5 s	.00	-
–0.5 to 0 s	–.05	-
0 to 0.5 s	-	–.34
0.5 to 1 s	-	**–.63****
1 to 1.5 s	-	–.03
1.5 to 2 s	-	.25

* $p \leq .05$; ** $p < .01$
